# Genetic diversity and molecular evolution of 3-carboxymuconate cyclase (Gp60–70), the major antigen in pathogenic *Sporothrix* species

**DOI:** 10.1080/21501203.2025.2467118

**Published:** 2025-03-08

**Authors:** Jamile Ambrósio de Carvalho, Thiago Costa Machado, Alexandre Augusto Sasaki, Fabian Glaser, Primavera Alvarado, Alexandro Bonifaz, Sarah Santos Gonçalves, Isabella Dib Gremião, Sandro Antonio Pereira, Olga Fischman Gompertz, Zoilo Pires de Camargo, Anderson Messias Rodrigues

**Affiliations:** a Laboratory of Emerging Fungal Pathogens, Department of Microbiology, Immunology, and Parasitology, Discipline of Cellular Biology, Federal University of São Paulo (UNIFESP), São Paulo, Brazil; b Department of Medicine, Discipline of Infectious Diseases, Federal University of São Paulo (UNIFESP), São Paulo, Brazil; c Bioinformatics Knowledge Unit, The Lorry I. Lokey Interdisciplinary Center for Life Sciences and Engineering, Technion—Israel Institute of Technology, Haifa, Israel; d Laboratorio de Micología, Instituto de Biomedicina “Dr. Jacinto Convit”, Apartado Postal Caracas Venezuela Apartado, Caracas, Venezuela; e Mycology Department, Hospital General de México “Dr. Eduardo Liceaga”, Mexico City, Mexico; f Center for Research in Medical Mycology, Department of Pathology, Federal University of Espírito Santo (UFES), Vitoria, Brazil; g Laboratory of Clinical Research on Dermatozoonoses in Domestic Animals, Evandro Chagas National Institute of Infectious Diseases (INI) – Oswaldo Cruz Foundation (Fiocruz), Rio de Janeiro, Brazil; h National Institute of Science and Technology in Human Pathogenic Fungi, São Paulo, Brazil

**Keywords:** Sporotrichosis, *Ophiostomatales*, 3-carboxymuconate cyclase, genetic diversity, evolution, virulence

## Abstract

Sporotrichosis, a neglected tropical disease caused by *Sporothrix* species, is a growing concern, particularly due to the emergence of highly virulent, cat-transmitted *S. brasiliensis*. Rapid diagnosis and surveillance are crucial for controlling sporotrichosis. This study investigated the 3-carboxymuconate cyclase (*CMC*) gene, which encodes the major *Sporothrix* antigen (Gp60–70), as a molecular marker to understand the genetic diversity and evolution of these fungi. Analysis of 104 isolates (*S. brasiliensis*, S. *schenckii*, *S. globosa*, and *S. luriei*) revealed 79 unique haplotypes, demonstrating superior discriminatory power over traditional molecular markers. High-*CMC* polymorphisms, especially in *S. brasiliensis* and *S. schenckii*, suggest recent population expansion or positive selection, potentially driven by environmental pressures such as polyaromatic hydrocarbon pollutants. The conserved chromosomal location of *CMC* in pathogenic *Sporothrix* and its absence in less virulent species suggest a role in virulence. Identifying conserved residues within predicted B-cell epitopes provides targets for diagnostics and therapeutics. Additionally, we identified *N*-linked glycosylation sequons (e.g. NGS at 62, NNT at 225, and NGT at 373/374) conserved in pathogenic *Sporothrix* but absent in environmental *Sordariomycetes*, possibly contributing to pathogenicity and niche adaptation. This study establishes *CMC* as a valuable marker for understanding *Sporothrix* evolution and virulence, aiding in sporotrichosis management.

## GRAPHICAL ABSTRACT

**Figure:**
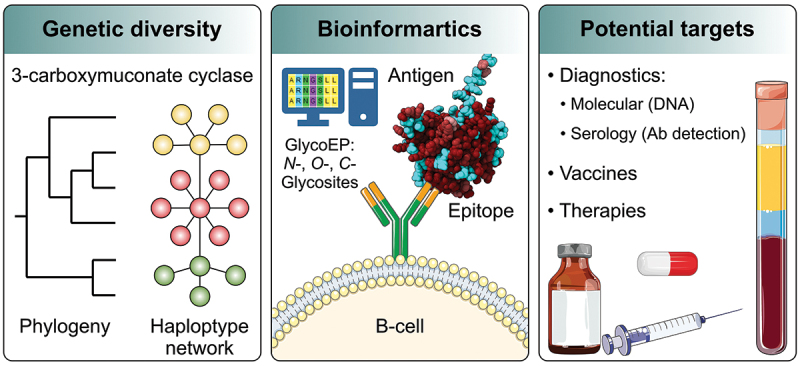

## 1. Introduction

*Sporothrix*, a diverse genus within the Ascomycete class *Sordariomycetes*, order *Ophiostomatales*, has emerged as a significant public health concern, particularly in tropical and subtropical regions (Bhunjun et al. 2024; Machado et al. 2025). While more than 70 *Sporothrix* species have been identified across diverse ecological niches, such as soil, plant debris, and insects (Rodrigues et al. 2020; de Beer et al. 2022), clinical clusters, including *S. brasiliensis*, *S. schenckii*, and *S. globosa*, exhibit heightened virulence and are primarily responsible for human and animal infections (Rodrigues et al. 2013a, 2013b, 2014, 2024). Sporotrichosis is an implantation mycosis involving transmission primarily through the traumatic inoculation of *Sporothrix* propagules from environmental sources (sapronosis), such as soil or plants, or via animal transmission, notably through scratches or bites from infected cats (de Carvalho et al. 2021; Rodrigues et al. 2022b).

Clinically, sporotrichosis presents with diverse manifestations, ranging from localised cutaneous and subcutaneous lesions to systemic dissemination. In humans, cutaneous lesions are common and often appear as nodules or ulcers arranged in cutaneous-lymphatic and cutaneous-fixed forms. Systemic dissemination is rare and can lead to severe complications such as meningitis and arthritis (Orofino-Costa et al. 2017, 2022; Ramírez-Soto 2025). In cats, sporotrichosis frequently presents with cutaneous lesions on the head, limbs, and tail, which can be ulcerative, nodular, or plaque like. Dissemination in this animal species can also occur, affecting various internal organs and potentially leading to severe systemic illness (Gremião et al. 2015, 2017, 2022).

Cat-transmitted sporotrichosis (CTS) is a significant public health issue in tropical and subtropical regions (Rodrigues et al. 2013a, 2013b, 2014). CTS is associated with sporadic outbreaks and is hyperendemic in areas such as Brazil, where *S. brasiliensis* spreads through epizooties and zoonoses (de Carvalho et al. 2021). Controlling the advancement of sporotrichosis necessitates tracking the spread of *Sporothrix* species via molecular markers. However, robust markers are lacking (de Carvalho et al. 2022). Traditionally, the evolutionary relationships of ribosomal genes, especially ITS1/2 + 5.8S, have been inferred (Zhou et al. 2014). While other markers, such as calmodulin (*CAL*) (Marimon et al. 2007), beta-tubulin (*BT2*) (Marimon et al. 2006), chitin synthase (*CHS*) (Kano et al. 2001), elongation factor 1-α (*EF1*-α) (Rodrigues et al. 2013b), aspartate aminotransferase (*AST*), glycerol-3-phosphate dehydrogenase fragment 2 (*GPD1*), cytosolic phospholipase A2 (*CPLA2*) and an uncharacterised protein (SPBR_05954) (de Souza Rabello et al. 2023), have been introduced, they are often limited to specific taxonomic groups or lack the discriminatory power needed for comprehensive epidemiological studies.

In medically relevant fungi, antigenic molecules can serve as informative molecular markers for studies on genetic diversity. Examples include *GP43* in *Paracoccidioides* spp. (Morais et al. 2000), *BAD1* for adhesin-1 in *Blastomyces* spp. (Maphanga et al. 2020), and the M and H antigens in *Histoplasma* spp. (Kasuga et al. 1999). Recently, we described the major antigen in *Sporothrix* species as 3-carboxymuconate cyclase (*CMC*), an exocellular glycoprotein containing multiple oligosaccharide chains that produces a protein with a molecular weight ranging between 60 and 70 kDa (Gp60–70) (Rodrigues et al. 2015c). The open reading frame of the *CMC* gene is within a 1,302–1,308-bp DNA fragment comprising two exons separated by a 63–66-bp intron (Rodrigues et al. 2015c). The *CMC* gene encodes a precursor protein of amino acids 412–413, which includes a signal peptide region of 21–22 residues (Rodrigues et al. 2015c). The processed Gp60–70 can be purified from *Sporothrix* culture medium (Nascimento and Almeida 2005) as a mixture of isoforms with six near but distinct isoelectric points (Rodrigues et al. 2015c).

Gp60–70 are recognised by antibodies in most sera from humans (Rodrigues et al. 2015c), cats (Rodrigues et al. 2015b), and experimentally infected rats (Fernandes et al. 2013), confirming the importance of this antigen in the *Sporothrix*-sporotrichosis system. In lymphocutaneous patients, Gp60–70 antibody titres are frequently elevated and tend to decrease as treatment progresses (Bernardes-Engemann et al. 2015). A favourable prognosis and clinical cure are often associated with reduced or absent antibody titres against *Sporothrix* antigens (Bernardes-Engemann et al. 2015). The capacity of Gp60–70 to bind laminin and fibronectin, in addition to its general presence in the *Sporothrix* cell wall, suggests a potential role in virulence (Teixeira et al. 2009; Castro et al. 2013). Therefore, vaccination with Gp70 alone or passive immunisation with anti-Gp70 antibodies can easily impact the development of sporotrichosis (Nascimento and Almeida 2005; Nascimento et al. 2008; Almeida 2012).

Given the importance of Gp60–70 in sporotrichosis and its versatile nature, this study investigated *CMC* gene polymorphisms in a diverse collection of medically relevant *Sporothrix* isolates from human and animal sporotrichosis cases. The complete precursor gene sequences of 104 *Sporothrix* isolates were determined and compared. We evaluated four critical aspects related to genetic diversity: 1) the potential of using *CMC* sequences to identify *Sporothrix* isolates in comparison with classical markers; 2) the genetic polymorphisms observed both within and between species; 3) the impact of these polymorphisms on the predicted amino acid sequence, revealing natural variants of Gp60–70; and 4) the influence of genetic diversity on the prediction of linear B-cell epitopes and the presence of putative *N*-, *O*-, and *C*-glycosylation sequons.

## 2. Materials and methods

### 2.1. Sporothrix *species isolates and culture conditions*

*Sporothrix* colonies were cultivated on Sabouraud dextrose agar (SDA) at 25 °C, with regular coculturing intervals of fourteen days (Brilhante et al. 2015). A group of 105 *Sporothrix* isolates was utilised, having undergone prior identification via multiplex qPCR (Della Terra et al. 2022) or phylogenetic analysis involving calmodulin and ITS1/2 + 5.8S. These isolates were classified as *S. brasiliensis* (*n* = 32), *S. schenckii* (*n* = 49), *S. globosa* (*n* = 22), *S. luriei* (*n* = 1), and *S. mexicana* (*n* = 1), thus encompassing members of the clinical and environmental clades (Marimon et al. 2008; Arrillaga-Moncrieff et al. 2009; Madrid et al. 2010; Rodrigues et al. 2014, 2015a; Zhou et al. 2014; Zhang et al. 2015; Moussa et al. 2017). *Sporothrix* isolates are maintained within the Laboratory of Emerging Fungal Pathogens Culture Collection at the Federal University of São Paulo (UNIFESP), São Paulo, Brazil. Moreover, our expanded dataset includes isolates from previously published studies with fully sequenced genomes (Cuomo et al. 2014; Teixeira et al. 2014; Huang et al. 2016; Gomez et al. 2018; New et al. 2019). These isolates have been obtained from various regions, including Australia, Brazil, Chile, China, Colombia, Italy, Mexico, Peru, Puerto Rico, South Africa, Spain, the USA, and Venezuela (Table S1).

### 2.2. DNA extraction

DNA was extracted from a 14-day-old monoconidial culture using a FastDNA kit (MP Biomedicals, Solon, OH, USA) following earlier established protocols (Rodrigues et al. 2014). The concentration and purity of the genomic DNA were evaluated via spectrophotometry (NanoDrop 2000; Thermo Fisher Scientific, Waltham, MA, USA). DNA extractions were deemed high quality when the *OD* 260/280 ratio fell within the range of 1.8–2.0. The absence of PCR inhibitors was confirmed by the successful amplification of an amplicon via the ITS1 and ITS4 primers in a PCR assay (White et al. 1990), as outlined in Table 1.

**Table 1.** Sequences of the primers used in this study.

Loci	Primer name	Orientation	Primer sequence (5‘−3’)	Tm (°C)	Reference
ITS1/2 + 5.8S	ITS1	Forward	TCC GTA GGT GAA CCT TGC GG	52	White et al. (1990)
	ITS4	Reverse	TCC TCC GCT TAT TGA TAT GC		
Calmodulin	CL1	Forward	GAR TWC AAG GAG GCC TTC TC	60	O’Donnell et al. (2000)
	CL2A	Reverse	TTT TTG CAT CAT GAG TTG GAC		
Ag70	Ag70_Up01	Forward	TCG CGC GAA TAT ACG GAA ATA CC	60	This study
	Ag70_Up02	Forward	GAT ATA AGA GGT CCC CTG GCT C	60	This study
	Ag70_Down	Reverse	CGT GAT TAA GCC GAG TAT AAA CCA GG	60	This study
	Ag70-F1	Forward	GCT GAA GTT TCT GGC TTT GGC	60	This study
	Ag70-R1	Reverse	GCA ACC CAG CAT GTA GCG TCT TGG	60	This study
	Ag70-F2	Forward	GTA TCA ACC CGA GAG GCA TGC	60	This study
	Ag70-R2	Reverse	CGT TAC GGT CCA GTC CCA GTG	60	This study
	Ag70-R (Probe)	Reverse	TTC GAC TGG GTA GGC AGC CA	60	This study
	Glo-gp70-F1	Forward	CAT CTT ACA ATC TAG TCA TCG CGC	60	This study
	Glo-gp70-F2	Forward	GTT CGA CTT CAT CTT GAA ACC ATT TG	60	This study
	Glo-gp70-Rv1	Reverse	GTT AAG CGC GAT ACG AAC CAG GC	60	This study
	Glo-gp70-Rv2	Reverse	CCT TGG TTA AGC GCG ATA CGA AC	60	This study

### 2.3. PCR amplification

The ITS1/2 + 5.8S region was utilised as the main barcoding marker, whereas the calmodulin gene was selected as a secondary polymorphic marker. PCR amplification and DNA sequencing of specific regions of the rRNA operon and the calmodulin gene (*CAL*) were achieved via the degenerate primers CL1 and CL2A (O’Donnell et al. 2000) and ITS1 and ITS4 (White et al. 1990), respectively (Table 1).

**Figure 1.**
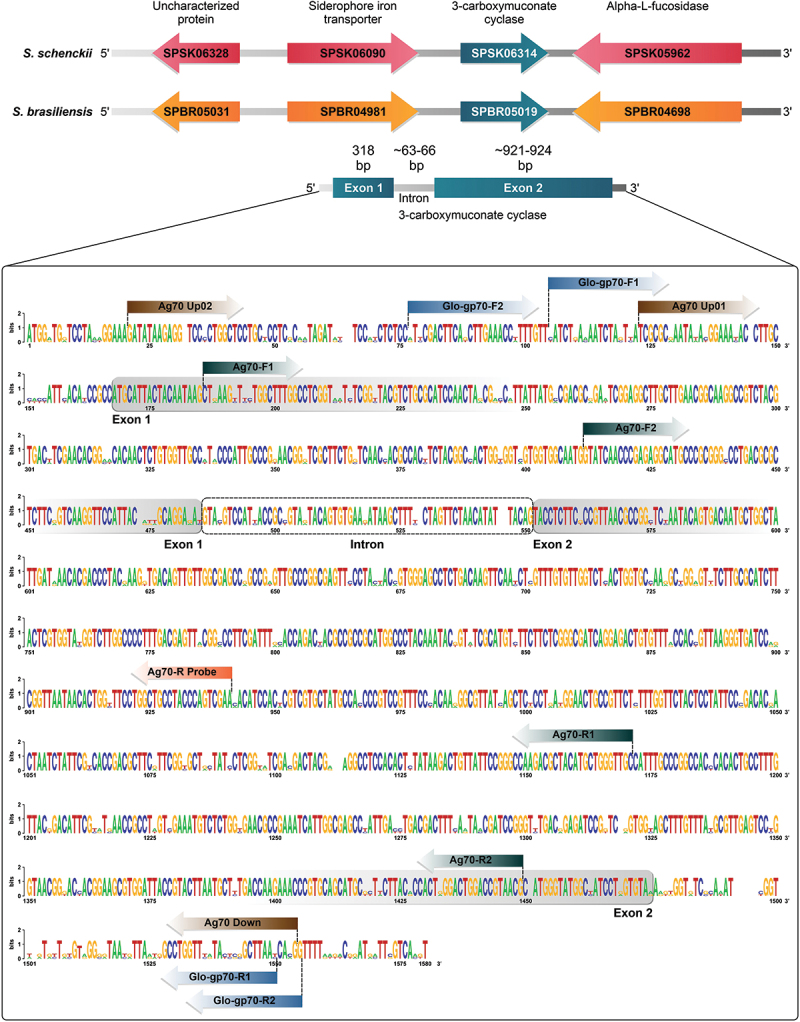
A WebLogo graphic representation was generated to illustrate polymorphism patterns within the alignment of three target sequences from *Sporothrix brasiliensis* (ATCC 4823; GeneID: 63678217), *S. schenckii* (ATCC 4821; GeneID: 27668297), and *S. globosa* (CBS 132922). The alignment revealed 12 primers recognising highly conserved regions shared among medically relevant *Sporothrix* species. The outermost forward primers (Ag70_Up01, Ag70_Up02, Glo-gp70-F1, and Glo-gp70-F2) and reverse primers (Ag70_Down, Glo-gp70-R1, and Glo-gp70-R2) are utilised for amplifying the entire gene, whereas the inner primers (Ag70-F1, Ag70-R1, Ag70-F2, and Ag70-R2) are employed for sequencing purposes.

The whole-genome sequences of *S. brasiliensis* (GenBank Accession No. AWTV01), *S. schenckii* (GenBank Accession No. AWEQ01 and AXCR01), and *S. globosa* (GenBank Accession No. LVYW01 and LVYX01) (Cuomo et al. 2014; Teixeira et al. 2014; Huang et al. 2016) were aligned via CLC Genomics Workbench version 9.0.1 (CLC Bio, Aarhus, Denmark). This alignment aimed to identify conserved regions in the 3-carboxymuconate cyclase gene sequences. Subsequently, pairs of oligonucleotides were designed via Primer3 (Untergasser et al. 2012), targeting upstream (Ag70_Up01 or Ag70_Up02) and downstream regions (Ag70_Down) and internal regions (*e.g*., Ag70-F1, Ag70-R1, Ag70-F2, Ag70-R2) to maximise sequencing coverage (Figure 1). Following primer design, the sequences were analysed via Mfold software (Zuker 2003) to predict potential secondary structures, guaranteeing minimal impact on amplification efficiency.

PCRs were set up in a total volume of 25 μL, containing 12.5 μL of PCR Master Mix buffer (2×), which included 3 mmol/L MgCl_2_, 400 mmol/L each dNTP, and 50 U/mL *Taq* DNA polymerase (Promega Corporation, Madison, WI, USA). Each reaction also included 1 μL of forward primer (10 pmol/μL; Integrated DNA Technologies, Coralville, IA, USA), 1 µL of reverse primer (10 pmol/μL), and 1 μL of target DNA (50 ng/μL). The amplifications were conducted using a Mastercycler X50 (Eppendorf, Hamburg, Germany). The thermal profile was initiated with a denaturation step at 95 °C for 5 min and then proceeded through 35 cycles consisting of denaturation at 95 °C for 1 min, annealing at 60 °C for 1 min, and extension at 72 °C for 1 min. A final extension stage of 10 min at 72 °C was included. Amplicons were resolved on a 1.2% agarose gel prepared with GelRed (Biotium, Hayward, CA, USA) in 1× TBE buffer and electrophoresed at 100 V for 1 h at room temperature (Sambrook and Russell 2001). Amplicons were visualised under UV light using the L-Pix Touch imaging system (Loccus Biotecnologia, São Paulo, Brazil), and fragment sizes were estimated with 100 bp GeneRuler DNA Ladders (Thermo Fisher Scientific, MA, USA).

After PCR amplification, the fragments were purified via the Wizard SV Gel and PCR Clean-Up System (Promega, Madison, WI, USA). The amplicons were sequenced in two reactions employing forward or reverse primers to increase sequence quality, ensuring a *Phred* score of ≥ 30. Sanger sequencing was performed with the BigDye Terminator v3.1 Cycle Sequencing Kit (Applied Biosystems), and the resulting data were analysed using the SeqStudio Genetic Analyser System (Applied Biosystems). The sequences obtained were submitted to the GenBank database and are listed in Table S1.

### 2.4. Phylogenetic reconstruction

The phylogenetic relationships of the DNA sequences were explored via neighbour-joining (NJ), maximum likelihood (ML), and maximum parsimony (MP) methodologies. Phylogenetic trees were constructed via MEGA11 (Tamura et al. 2021). Evolutionary distances were calculated via the Kimura 2-parameter model (Kimura 1980) or Tamura 3-parameter (Tamura and Kumar 2002), and the reliability of branches was ascertained through a bootstrap analysis involving 1,000 replicates (Felsenstein 1985).

A dataset of sixty-nine 3-carboxymuconate cyclase protein sequences from environmental *Sordariomycetes* was obtained from the NCBI database (Sayers et al. 2022). This dataset was selected for its diversity in sequence length, sample size, and sequence identity. These environmental sequences were aligned via MAFFT (Katoh and Standley 2013) with five *CMC* sequences from representatives from the clinical clade. We subsequently determined the best- and worst-fitting substitution models of protein evolution via the tools provided by MEGA11 (Tamura et al. 2021) based on the Bayesian information criterion (BIC) (Schwarz 1978) and corrected Akaike information criterion (AIC) (Hurvich and Tsai 1989). An ML phylogenetic tree was constructed, employing the selected best-fitting substitution model WAG+G+I (Whelan and Goldman 2001).

### 2.5. Nucleotide diversity, haplotype network, and recombination analysis

Nucleotide (π) and haplotype diversity (Hd) values (Nei 1987), Tajima’s D (neutrality test) (Tajima 1989), and the number of synonymous and nonsynonymous substitutions (Nei and Gojobori 1986) were calculated using DnaSP v6.12 (Rozas et al. 2017). Sites containing gaps and missing data were excluded from the analysis. Haplotype network analysis was performed via the median-joining method (Bandelt et al. 1999) and implemented in NETWORK 4.6.1.0 software (Fluxus-Technology, Colchester, United Kingdom). Potential recombination events were explored via the neighbour-net method (Bryant and Moulton 2004), which reveals reticulated relationships in the occurrence of recombination. This phenomenon was further elucidated through the Uncorrected-P distance or the split decomposition method (Bandelt and Dress 1992), which was integrated into SplitsTree v.4b06 (Huson and Bryant 2006). Further assessments of recombination were conducted via the PHI test, where a *p* value < 0.05 indicated a relevant sign of recombination.

### 2.6. Gene flow and genetic differentiation

Genetic differentiation and gene flow were estimated using DnaSP v6.12 (Rozas et al. 2017) for the aligned *CMC* nucleotide sequences. Genetic differentiation among populations was calculated based on Hudson’s statistics, including haplotype diversity (*Hs*), nucleotide diversity (*Ks*), and the fixation index (*Fst*), which evaluate the genetic structure of *Sporothrix* species (Hudson et al. 1992a, 1992b). Additionally, we calculated the haplotype-based statistic (*Hst*), nucleotide-based statistic (*Kst*), and neutrality tests (*Z* and Z*) to assess population differentiation. The significance was determined via permutation tests with 1,000 replicates (*p* < 0.001, 0.01 < *p* < 0.05, and not significant) (Hudson et al. 1992a). A chi-square test (*X*^*2*^) was also performed to evaluate overall genetic differentiation. Gene flow was estimated using the genetic differentiation statistic (*Gst*) and the number of migrants per generation (*Nm*) via Nei’s 1973 and 1982 methods (Nei 1973, 1982). *GammaSt*, based on allele frequency differences across populations, was computed to provide insights into the degree of population subdivision (Hudson et al. 1992a).

### 2.7. Pulsed-field gel electrophoresis and densitometric analysis

The electrophoretic karyotypes of the reference isolates were analysed to determine the chromosomal location of the 3-carboxymuconate cyclase gene in the *Sporothrix* genome. Pulsed-field gel electrophoresis (PFGE) was performed according to the protocol described by Sasaki et al. (2014), with modifications. Briefly, DNA plugs were resolved on 0.8% agarose gels using a Gene Navigator System (Amersham Pharmacia Biotech, USA) at 10 °C in 1× TAE buffer. The electrophoresis process spanned 168 h at 42 V, employing a stepwise variation of pulse times to optimise the resolution: Phase 1 (900 s for 24 h), Phase 2 (1,800 s for 24 h), Phase 3 (2,700 s for 48 h), Phase 4 (3,600 s for 48 h), and Phase 5 (4,500 s for 24 h). Chromosomal size markers from *Schizosaccharomyces pombe* (Bio-Rad, USA) were used for size estimation. Following electrophoresis, the gel was stained with ethidium bromide (0.5 µL/mL), rinsed with distilled water, and visualised using the L-Pix Touch image system (Loccus Biotecnologia, Brazil). Chromosome sizes were quantified by densitometric analysis with LabImage 1D software (Kapelan Bio-Imaging, Germany), and the results were calibrated against the *S. pombe* chromosomal markers.

### 2.8. Radiolabeling probes and southern blot hybridisation

Genomic fragments of the *CMC* gene were amplified from *S. schenckii* (Ss126) (100 ng/µL) via PCR using the Ag70-F2 and Ag70-R primers (Probe). The resulting amplicons were subsequently cloned and inserted into the pGEM-T Easy Vector System (Promega), adhering to the manufacturer’s guidelines, and transformed into DH5-α competent cells (Invitrogen) with subcloning efficiency according to the provided protocol. Plasmid purification was conducted via the NucleoSpin Plasmid Kit (Macherey-Nagel, Germany), followed by sequencing (Sasaki et al. 2014). Following the manufacturer’s protocol, probes were generated from plasmid inserts excised by the EcoRI restriction enzyme (Fermentas, USA) and purified via the NucleoSpin Extract II kit (Macherey-Nagel, Germany). The *CMC* probe was radiolabeled via a random primer labelling system (Invitrogen) and dCTP (α-P32) (Perkin Elmer, USA).

Following PFGE, chromosomal bands were blotted onto a Hybond-N nylon membrane (Amersham Bioscience, United Kingdom) via capillary transfer at 25 °C. The DNA was then crosslinked to the membrane under UV light (150 mJ) in a GS Gene Linker UV Chamber (Bio-Rad, USA). The membranes were subsequently stored at − 4 °C until further analysis (Sasaki et al. 2014).

For hybridisation, the membranes were incubated for 30 min in prehybridisation buffer at 65 °C. The prehybridisation buffer comprised 0.25 mol/L Na_2_PO_4_, 2 mL of phosphoric acid (85%), 7% SDS, 1% BSA, and 1 mmol/L EDTA. Radiolabeled probes were included, and hybridisation proceeded for 16 h at 65 °C. The membranes were subsequently washed first in 20 mL of Solution I (0.25 mol/L Na_2_PO_4_, 2 mL of phosphoric acid, 1% SDS, and 1 mmol/L EDTA) for 30 min and then in 20 mL of Solution II (0.125 mol/L Na_2_PO_4_, 1 mL of phosphoric acid [85%], 1% SDS, and 1 mmol/L EDTA) for another 30 min. After washing, the membranes were exposed to X-ray film (GE Healthcare, United Kingdom) at − 80 °C for 2 to 4 days to visualise hybridisation patterns, which were subsequently analysed following film development (Sasaki et al. 2014).

### 2.9. Bioinformatic analysis

For *in silico* analysis of protein antigenicity, B-cell epitopes were predicted and compared between *S. brasiliensis*, *S. schenckii*, *S. globosa*, and *S. luriei*. Protein sequences were subjected to ABCpred (Saha and Raghava 2006), BepiPred (Jespersen et al. 2017), and COBEpro (Magnan et al. 2010) using the default parameters. AntigenPRO (Magnan et al. 2010) and VaxiJen v2.0 (Doytchinova and Flower 2007), two sequence-based, alignment-free, and pathogen-independent predictors of protein antigenicity, were employed with a threshold of 0.5. Protein sequences with antigenic scores exceeding 0.5 were considered antigenic.

The Consurf server (Ashkenazy et al. 2016) was employed to estimate the evolutionary conservation of amino acid residue positions for each predicted B-cell epitope. Multiple sequence alignment was accomplished via MAFFT (Katoh and Standley 2013) for members of the *Sporothrix* clinical clade and members of the *Sordariomycetes*. The degree of amino acid position conservation was calculated via Rate4Site software (version 2.01) (Pupko et al. 2002), and the results were compared with those of predicted B-cell epitopes (Jespersen et al. 2017). The conservation scores were categorised into a discrete scale of 9 bins for visualisation, where bin 9 represented the most conserved positions and bin 1 represented the most mutable positions. Molecular graphics and analyses were conducted using UCSF ChimeraX (Meng et al. 2023), a tool created by the Resource for Biocomputing, Visualisation, and Informatics at the University of California, San Francisco (NIH grant R01-GM129325) (Pettersen et al. 2021).

We used the Pfam 33.1 database (Mistry et al. 2021) to annotate the *CMC* protein sequences. To investigate *in silico* the presence and population-level conservation of *N*-, *O*-, and *C*-linked glycosylation sequons in Gp60–70 of *S. brasiliensis*, *S. schenckii*, *S. globosa*, and *S. luriei*, we used GlycoEP (Chauhan et al. 2013). GlycoEP employs the binary profile of patterns (BPP) algorithm, which converts amino acid sequences into binary patterns to identify potential glycosylation sequons.

To assess the structural and functional importance of residues within the *CMC* protein, we employed the LERI web server (Cheung et al. 2021), a computational tool that uses evolutionary coupling analysis (ECA) to identify coevolving residues. The SAEC (spectrum analysis on evolutionary coupling) method was applied to multiple *CMC* protein sequence alignments (MSAs) from clinical *Sporothrix* species and the broader *Sordariomycetes* dataset. This analysis aimed to identify “residue communities”, which are networks of interconnected residues exhibiting strong evolutionary couplings, suggesting functional interdependence and evolutionary constraints. The degree of residue conservation across homologous sequences was visualised on a circular plot, highlighting the relationship between evolutionary couplings and conservation.

## 3. Results

### 3.1. CMC *gene structure and primer design*

The genomic analysis revealed that the *CMC* gene (encoding 3-carboxymuconate cyclase; GeneID: 27668297, SPSK06314; GeneID: 63678217, SPBR_05019) is flanked by genes encoding alpha-L-fucosidase (GeneID: 27667947, SPSK05962; GeneID: 63677896, SPBR04698) and a siderophore-iron transporter (GeneID: 27668074, SPSK06090; GeneID: 63678179, SPBR04981) in *S. brasiliensis* and *S. schenckii* (accessions AWTV01000010 and AXCR01000001). On this basis, a set of 12 primers spanning exonic and intronic sections of the *CMC* gene was designed (Figure 1). To amplify the whole gene from the *S. brasiliensis* and *S. schenckii* isolates, Ag70_Up01 or Ag70_Up02, in conjunction with Ag70_Down, were utilised. The primers Ag70-F1, Ag70-R1, Ag70-F2, and Ag70-R2 were also used for DNA sequencing. For *S. globosa*, amplification and sequencing were performed via the primers Glo-gp70-F1, Glo-gp70-F2, Glo-gp70-Rv1, and Glo-gp70-Rv2 (Figure 1).

### 3.2. CMC *gene diversity analysis*

Analysis of the complete *CMC* gene sequence from 104 isolates [94 from this study, 10 from previous work (Cuomo et al. 2014; Teixeira et al. 2014; Rodrigues et al. 2015c; Huang et al. 2016; Gomez et al. 2018; New et al. 2019)] revealed that the *CMC* gene spans between 1,302 and 1,308 base pairs (bp) in members of the clinical clade and comprises two exons (318 bp, 921–924 bp) separated by an intron (63–66 bp). A total of 79 haplotypes (*H*) with high haplotype diversity (*Hd* = 0.990) were observed. Strobeck’s S statistic (Strobeck 1987) returned a perfect value of 1.000, indicating a high probability that the observed number of haplotypes (79) is accurate. Fu’s Fs statistic (Fu 1997) revealed a negative value of −9.415, suggesting a substantial excess of rare alleles in the population, indicative of positive selection or recent population expansion.

*S*. *schenckii* (*n* = 49) presented the highest *CMC* nucleotide diversity (π = 0.01832) and haplotype diversity (*Hd* = 0.986), followed closely by *S. brasiliensis* (*n* = 32, π = 0.00802, *Hd* = 0.978). In contrast, *S. globosa* (*n* = 22) presented lower nucleotide diversity (π = 0.00350) and haplotype diversity (*Hd* = 0.896), suggesting a comparatively lower level of genetic diversity within this species. This observation is further supported by Fu’s Fs statistic, where *S. brasiliensis* and *S. schenckii CMCs* exhibit notably negative values (−12.606 and −10.264, respectively), indicative of population expansion or positive selection. In contrast, *S. globosa* presented a low value (−1.648), suggesting a different demographic history. Additionally, Strobeck’s S statistic indicates a high probability of the observed number of haplotypes being accurate for all three species (Table 2). The *CMC* gene shows high synonymous nucleotide diversity (π = 0.10995) but low nonsynonymous diversity (π = 0.02316), suggesting purifying selection. This is expected for a gene encoding a critical enzyme such as *CMC*, where nonsynonymous changes may be deleterious, as depicted by a Tajima’s D (NonSyn/Syn) ratio of −0.41730.

**Table 2.** Statistical values from the different phylogenetic analyses.

	n	N. of sites	C	V	Pi	Si	S	η	H	Hd	Fu’s Fs statistic	Strobeck’s S statistic	π	*k*
*Sporothrix brasiliensis CMC*	32	1,303	1,229	73	28	45	73	80	27	0.978	−12,606	1.000	0.00802	10.445
*S. schenckii CMC*	49	1,306	1,167	139	105	34	139	147	40	0.986	−10,264	1.000	0.01832	23.929
*S. globosa CMC*	22	1,308	1,281	27	18	9	27	28	11	0.896	−1,648	0.928	0.00350	4.584
Overall *CMC*^1^	104	1,308	941	367	222	145	361	434	79	0.990	−9,415	1.000	0.04789	61.400
*CMC*^2^	56	1,308	985	323	169	154	316	369	44	0.9857	−1,738	0.914	0.04850	62.180
ITS	56	413	393	15	10	5	12	12	14	0.8221	5.691	0.013	0.00952	3.771
*CAL*	56	585	465	109	60	49	95	111	22	0.9091	7.163	0.002	0.04219	22.025
MLSA^3^	56	2,306	1,843	447	239	208	423	492	50	0.9942	–	–	0.03999	87.977

^1^Expanded dataset #1 encompasses 104 isolates, including all sequences of *CMC* generated in this study. ^2^Dataset #2 comprises 56 isolates utilised for comparison purposes with ITS1/2 + 5.8S and calmodulin. ^3^MLSA: Multilocus sequence analysis, combining *CMC*, ITS1/2 + 5.8S, and *CAL* sequences. C = conserved characters; V = variable characters; Pi = parsimony-informative characters; Si = singletons; S = number of polymorphic (segregating) sites; η = total number of mutations; H = number of haplotypes; Hd = haplotype (gene) diversity; π = nucleotide diversity; *k* = average number of nucleotide differences.

The PHI test results revealed statistically significant evidence of recombination for *S. brasiliensis* (*p* = 0.03677) when a window size of 100 and *k* = 2 were used, with a mean of 0.2672, variance of 0.00193, and observed value of 0.1887. In contrast, no significant evidence of recombination was found for *S. schenckii* (*p* = 0.2857; *k* = 8) or *S. globosa* (*p* = 1.0; *k* = 1), with mean and observed values of 0.0727 and 0.0684 for *S. schenckii* and 0.0 for *S. globosa*. These findings suggest differences in recombination activity among the species.

### 3.3. Gene flow and genetic differentiation

The genetic differentiation and gene flow among three *Sporothrix* species (*S. brasiliensis*, *S. schenckii*, and *S. globosa*) were evaluated based on 103 sequences across 1,242 sites. The average number of nucleotide differences (*K*) was highest in *S. schenckii* (23.929) and lowest in *S. globosa* (4.584), with an overall mean of 61.400 (Table 2). Genetic differentiation was significant, with a chi-square value of 206.000 (*p* = 0.0023, df = 152). The HBK 1992 parameters showed low differentiation within populations (*Hs* = 0.96450) but high differentiation between populations (*Hst* = 0.02585, *p* < 0.001). Similarly, the *Kst* value was 0.60670 (*p* < 0.001), indicating substantial genetic differentiation across populations. On the basis of Nei’s 1973 *Gst*, gene flow estimates suggest a high level of gene flow (*Nm* = 8.90). However, the sequence-based estimate (*GammaSt* = 0.61250, *Nm* = 0.16) and Hudson, Slatkin, and Maddison’s 1992 *Fst* (0.74852, *Nm* = 0.08) revealed limited gene flow (Table S2). The pairwise *Fst* values between the populations ranged from 0.66201 (between *S. brasiliensis* and *S. schenckii*) to 0.84368 (between *S. brasiliensis* and *S. globosa*), further confirming the significant genetic structure among the *Sporothrix* species (Table 3). These results indicate strong genetic differentiation and limited gene flow between the populations, particularly between *S. brasiliensis* and *S. globosa*.

**Table 3.** Genetic differentiation among medically relevant *Sporothrix* species on the basis of the *CMC* gene.

Population 1	Population 2	*Hs*	*Ks*	*Kxy*	*Gst*	*GammaSt*	*Fst*
*S. brasiliensis*	*S. schenckii*	0.98227	17.55418	48.01339	0.00967	0.47269	0.66201
*S. brasiliensis*	*S. globosa*	0.94393	7.73989	46.35938	0.03236	0.71787	0.84368
*S. schenckii*	*S. globosa*	0.95944	16.98097	52.91837	0.0278	0.50559	0.74365

*Hs*: Haplotype diversity; *Ks*: Nucleotide diversity within populations; *Kxy*: Average number of nucleotide differences between populations; *Gst*: Genetic differentiation based on haplotypes; *GammaSt:* Genetic differentiation based on nucleotide sequences; *Fst*: Fixation index.

### 3.4. Comparison with traditional markers (ITS and calmodulin)

**Figure 2.**
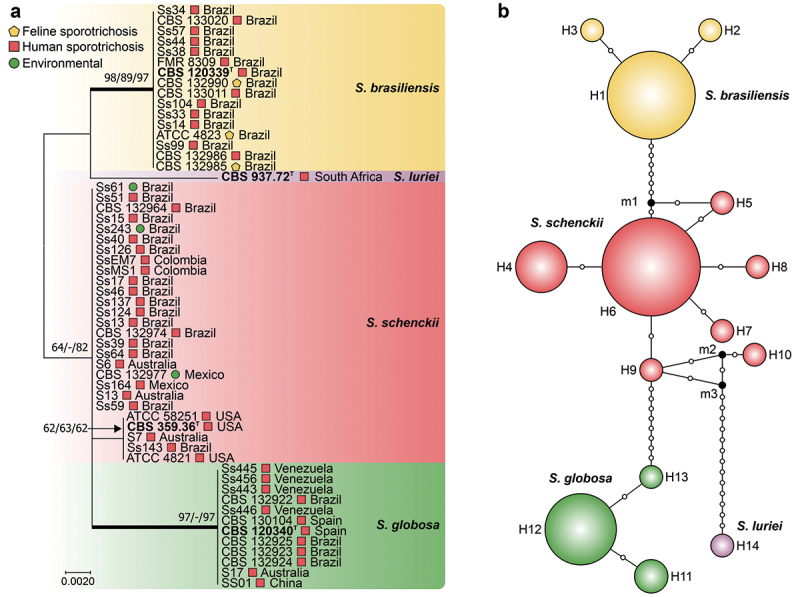
Phylogenetic relationships and haplotype network of *Sporothrix* species based on ITS sequences. (a) Phylogenetic tree derived from maximum likelihood (ML) analyses of rDNA operon sequences (ITS1, 5.8S, and ITS2). Bootstrap values (1,000 replicates) are annotated on branches (ML/MP/NJ), with species indicated. The bar denotes nucleotide differences. (b) Median-joining haplotype network based on ITS sequences, with circumference size proportional to haplotype frequency. Isolates are coded by species, and mutational steps are depicted by white dots, while black dots (median vectors) represent unsampled or extinct haplotypes. “T” indicates the type strain.

**Figure 3.**
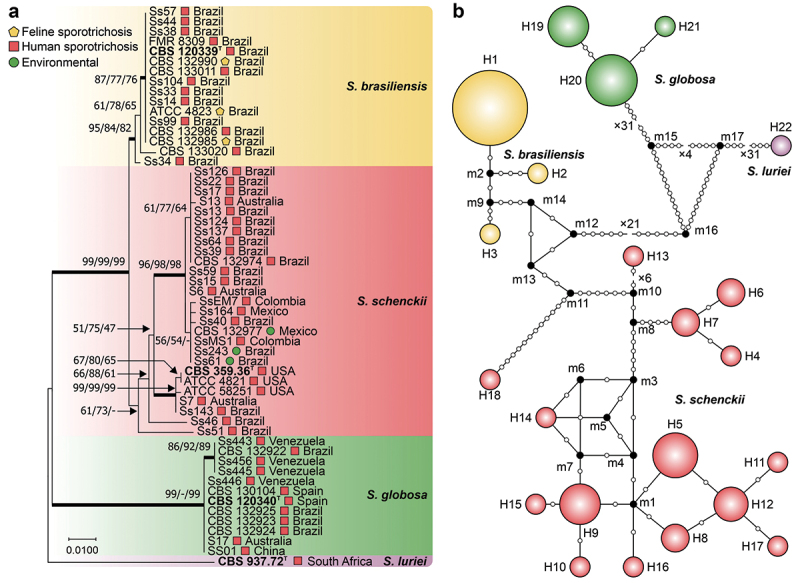
Phylogenetic relationships and haplotype network of *Sporothrix* species based on calmodulin sequences. (a) Phylogenetic tree derived from maximum likelihood (ML) analyses of calmodulin sequences (exons 3–5). Bootstrap values (1,000 replicates) are annotated on branches (ML/MP/NJ), with species indicated. The bar denotes nucleotide differences. (b) The median-joining haplotype network is based on calmodulin sequences, with the circumference size proportional to the haplotype frequency. Isolates are coded by species, and mutational steps are depicted by white dots, while black dots (median vectors) represent unsampled or extinct haplotypes. “T” indicates the type strain.

Using ITS1/2 + 5.8S and calmodulin as control markers, we observed lower haplotype and nucleotide diversity than the *CMC* gene did (Table 2, Figures 2 and 3). The ITS dataset comprised 413 characters (ITS1/2 + 5.8S), revealing 15 (3.63%) variable characters, 10 (2.42%) parsimony-informative characters, and 5 (1.21%) singletons. The phylogenetic reconstruction revealed well-defined groups, with *S. luriei* appearing closer to *S. brasiliensis*, whereas *S. schenckii* and *S. globosa* occupied more external positions. Haplotype analysis revealed an *Hd* of 0.8221 and a π of 0.00952, indicating moderate diversity within the ITS region, with an average number of nucleotide differences (*k*) of 3.771 (Figure 2; Table 2). In contrast, the calmodulin dataset contained 585 characters (exons 3–5), exhibiting more significant variability, with 109 (18.64%) variable characters, 60 (10.26%) parsimony-informative characters, and 49 (8.38%) singletons. Compared with those in the ITS dataset, *Hd* (0.9091) and π (0.04219) were greater, indicating increased diversity within the calmodulin region (Figure 3; Table 2).

**Figure 4.**
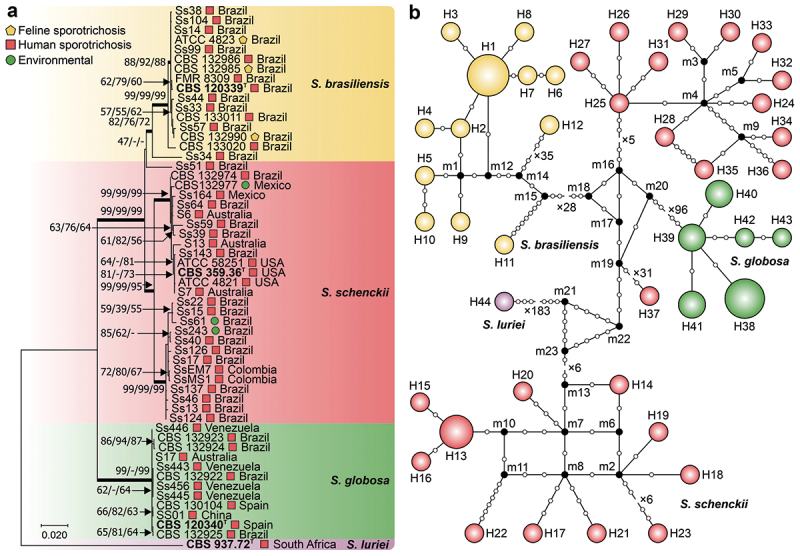
Phylogenetic relationships and haplotype network of *Sporothrix* species based on 3-carboxymuconate cyclase sequences. (a) Phylogenetic tree derived from maximum likelihood (ML) analyses of 3-carboxymuconate cyclase (*CMC*) sequences. Bootstrap values (1,000 replicates) are annotated on branches (ML/MP/NJ), with species indicated. The bar denotes nucleotide differences. (b) Median-joining haplotype network based on 3-carboxymuconate cyclase sequences, with the circumference size proportional to the haplotype frequency. Isolates are coded by species, and mutational steps are depicted by white dots, while black dots (median vectors) represent unsampled or extinct haplotypes. “T” indicates the type strain.

We analysed a smaller subset of 56 diverse *Sporothrix* isolates matching the ITS1/2 + 5.8S and calmodulin datasets to further test the effectiveness of the *CMC* gene in estimating species relationships. Within this subset, the *CMC* gene comprised 56 sequences and 1,308 characters, of which 323 (24.66%) were variable, 169 (12.91%) were parsimony informative, and 154 (11.76%) were singletons (Table 2). Phylogenetic analysis of the *S. schenckii s. str*. isolates revealed two distinct genetic clusters with contrasting geographic distributions. The first cluster consists primarily of isolates from South America, mainly Brazil (*e.g*., Ss40 and Ss61) and Colombia (*e.g*., SSEM7 and SsMS1). The second cluster demonstrates greater diversity, encompassing representatives from Brazil, the United States (*e.g*., CBS 359.36 and ATCC58251), Australia (*e.g*., S6, S13, and S7), and Mexico (*e.g*., CBS 132977, Ss164). Notably, isolate Ss51, a classical genetic variant, occupied a position external to the other *S. schenckii s. str*. isolates. A haplotype network analysis identified 44 unique haplotypes among the isolates. The calculated haplotype diversity of 0.9857 underscores the potential of the *CMC* gene as a valuable marker for understanding *Sporothrix* diversity and speciation (Figure 4).

**Figure 5.**
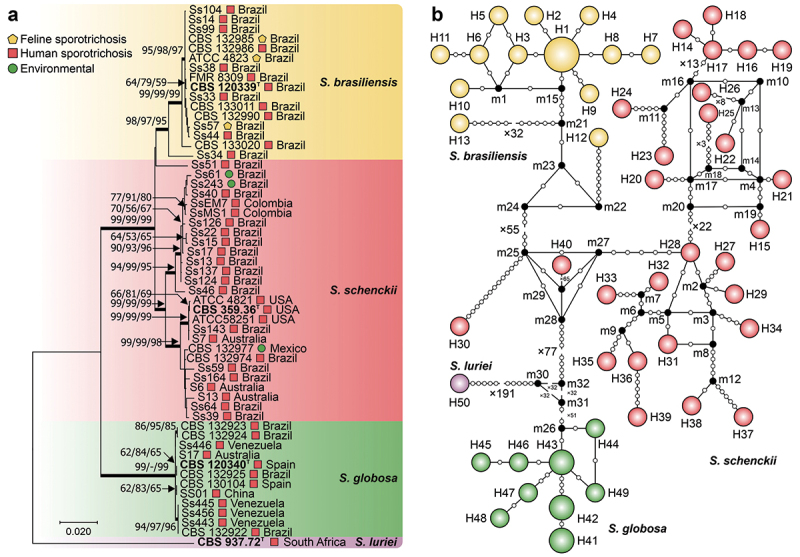
Phylogenetic relationships and haplotype network of *Sporothrix* species based on multilocus sequence analysis of ITS1/2 + 5.8S, calmodulin, and 3-carboxymuconate cyclase. (a) Phylogenetic tree derived from maximum likelihood (ML) analyses of concatenated *CMCs*, calmodulin, and ITS1/2 + 5.8S. Bootstrap values (1,000 replicates) are annotated on branches (ML/MP/NJ), with species indicated. The bar denotes nucleotide differences. (b) Median-joining haplotype network based on concatenated *CMC*, calmodulin, and ITS1/2 + 5.8S sequences, with the circumference size proportional to the haplotype frequency. Isolates are coded by species, and mutational steps are depicted by white dots, while black dots (median vectors) represent unsampled or extinct haplotypes. “T” indicates the type strain.

A multilocus sequence analysis was conducted using a subset of 56 isolates with available ITS1/2 + 5.8S and calmodulin sequences to assess the combined power of the *CMC* alongside established markers. Individually, ITS and calmodulin exhibited moderate haplotype and nucleotide diversity (Table 2; Figures 2 and 3). However, when all three loci were incorporated, the concatenated dataset demonstrated remarkably high haplotype diversity (*Hd* = 0.9942) and revealed 50 unique haplotypes (Figure 5). Compared with individual markers, this MLSA approach significantly enhanced the resolution of species boundaries and revealed a finer-scale genetic structure within the *Sporothrix* clinical clade, underscoring the synergistic value of combining *CMC* with established loci for a more comprehensive understanding of *Sporothrix* diversity.

### 3.5. *Chromosomal location and evolutionary conservation of* CMC

**Figure 6.**
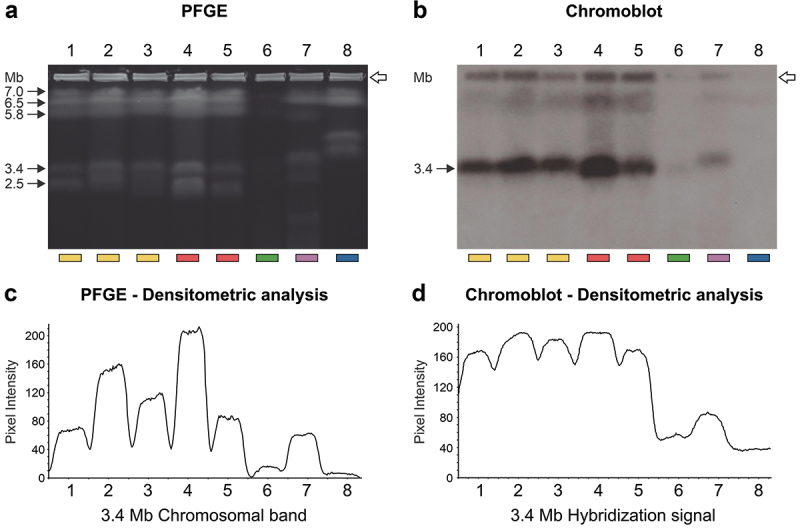
Chromosomal localisation of the *CMC* gene in *Sporothrix* species. (a) Ethidium bromide-stained gel after pulsed-field gel electrophoresis (PFGE) separation of chromosomes. (b) Southern blots showing the hybridisation of a radiolabeled *CMC* probe to *Sporothrix* chromosomes. The probe spans the region between the primers Ag70-F2 and the Ag70-R probe. (c) Densitometric analysis of the PFGE gel, highlighting a 3.4 Mb chromosomal band. (d) Densitometric analysis of the hybridisation pattern of the *CMC* probe, corresponding to the 3.4 Mb band. Chromosomal band sizes (Mb) are indicated on the left and above the peaks in the densitometric analysis. The open arrows indicate the sample loading positions. Lanes 1–8 contain CBS 132985 (*S. brasiliensis*, yellow bar), CBS 120339 (*S. brasiliensis*, yellow bar), IPEC 16919 (*S. brasiliensis*, yellow bar), CBS 359.36 (*S. schenckii*, red bar), Ss126 (*S. schenckii*, red bar), CBS 120340 (*S. globosa*, green bar), CBS 937.72 (*S. luriei*, purple bar), and CBS 120341 (*S. mexicana*, blue bar), respectively.

**Figure 7.**
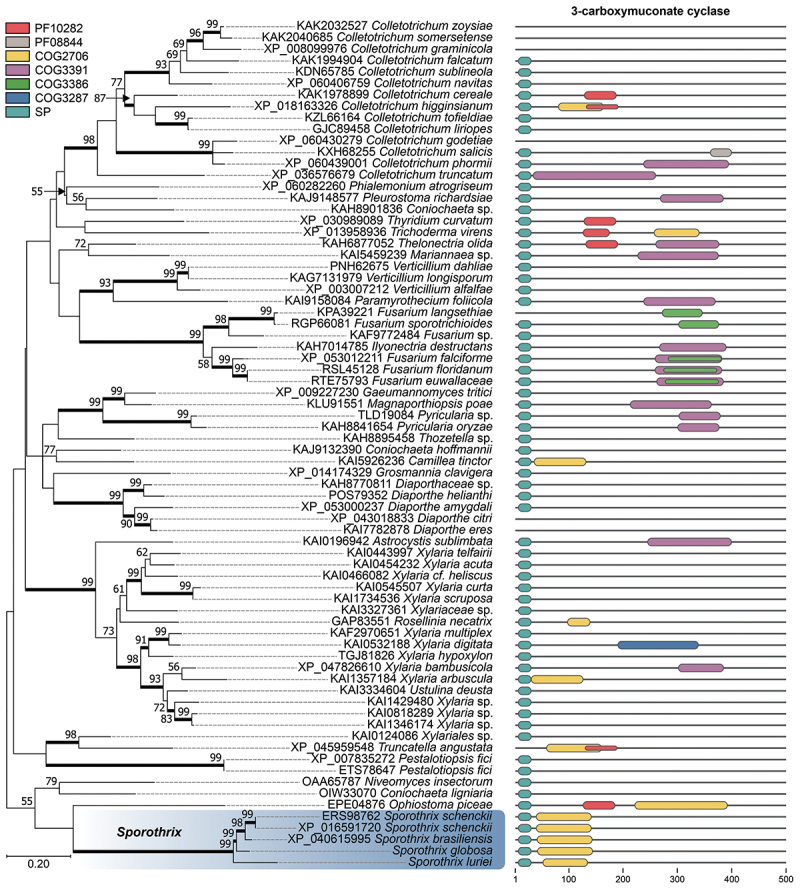
Phylogenetic analysis and domain architecture of 3-carboxymuconate cyclase (*CMC*) homologs in *Sordariomycetes*. The phylogenetic tree illustrates the evolutionary relationships of the *CMC* protein sequences from various *Sordariomycetes*, with *Ophiostoma piceae* identified as the closest relative to the pathogenic *Sporothrix* species. The tree was constructed via a maximum likelihood approach with the WAG+G+I substitution model. Branch support values (bootstrap percentages) are indicated at the nodes. The presence of specific protein domains within the 3-carboxymuconate cyclase (*CMC*) homologs is colour coded. Red: PF10282 (Lactonase, 7-bladed beta-propeller); yellow: COG2706 (3-carboxymuconate cyclase); purple: COG3391 (Uncharacterized conserved protein); green: COG3386 (Gluconolactonase); grey: PF08844 (Domain of unknown function, DUF1815); blue: COG3287 (Uncharacterized conserved protein); and teal: signal peptide (SignalP-6.0). Notably, COG2706 (3-carboxymuconate cyclase) is consistently present in pathogenic *Sporothrix* species, whereas environmental *Sordariomycetes* exhibit greater domain diversity. Most *CMC* homologs contain a signal peptide (SP), suggesting a secreted function.

We conducted Southern blot analysis to determine the genomic location of the *CMC* gene within *Sporothrix* species. This analysis localised the *CMC* gene to a 3.4 Mb chromosomal band in *S. brasiliensis*, *S. schenckii*, *S. globosa*, and *S. luriei*, but notably, it was absent in *S. mexicana* (Figure 6). This absence was further confirmed by *CMC*-BLASTn searches, which revealed no significant homology in the reference genomes of *S. pallida* (CBS 131.56; GCA_021396235.1), *S. humicola* (CBS 118129; GCA_021396245.1), *S. inflata* (CBS 239.68; GCA_021396225), *S. mexicana* (CBS 120341; GCA_021396375.1), *S. curviconia* (CBS 959.73; GCA_016097085.2), *S. variecibatus* (CBS 121960; GCA_016097105.2), *S. protearum* (CBS 116654; GCA_016097115.2), *S. phasma* (CBS 119588; GCA_016097075.2), *S. pseudoabietina* (VPRI43531; GCA_019925295.1), *S. euskadiensis* (VPRI43754; GCA_019925375.1), *S. dimorphospora* (CBS 553.74; GCA_021397985.1), *S. nigrograna* (VPRI43755; GCA_019925305.1), and *S. brunneoviolacea* (CBS 124561; GCA_021396205.1). This unexpected finding broadened our investigation to all *Sordariomycetes*. A comprehensive search of protein databases revealed the presence of *CMC* homologs in other members of this class, with the closest relative identified as *Ophiostoma piceae* (Figure 7).

Further analysis of protein domains revealed distinct patterns in *CMC* homologs across different fungal groups. In all clinical *Sporothrix* species, the COG2706 domain, which is characteristic of 3-carboxymuconate cyclase, was consistently identified. In contrast, the COG2706 domain was present sporadically in environmental fungi such as *Colletotrichum*, *Trichoderma*, *Camillea*, *Rosellinia*, *Xylaria*, *Truncatella*, and *Ophiostoma*. Moreover, in environmental *Sordariomycetes*, *CMC* homologs exhibited greater diversity, with the presence of other domains, such as COG3391 (uncharacterised conserved protein) and PF10282 (lactonase, 7-bladed beta-propeller), in addition to COG2706. Notably, 66 out of the 74 evaluated *CMC* sequences contained a signal peptide, strongly suggesting that *CMC* is a secreted protein (likelihood = 0.9). This consistent presence of COG2706 in pathogenic *Sporothrix*, along with the protein secretion pathway, provides further insight into the likely role of *CMC* in the virulence of these species, while the domain variation in environmental *Sordariomycetes* suggests potential functional diversification in these fungi.

### 3.6. Antigenicity and epitope prediction

*CMC* (Gp60–70), a key serological marker for sporotrichosis, was further investigated for its role in linear B-cell epitopes. Previous research has revealed cross-reactivity among clinical *Sporothrix* species, suggesting that conserved epitopes are highly specific, indicating that these epitopes are absent in other fungal antigens. ConSurf analysis of the *CMC* protein revealed high conservation of amino acid residues among pathogenic species and significant divergence within the broader *Sordariomycetes* dataset.

**Figure 8.**
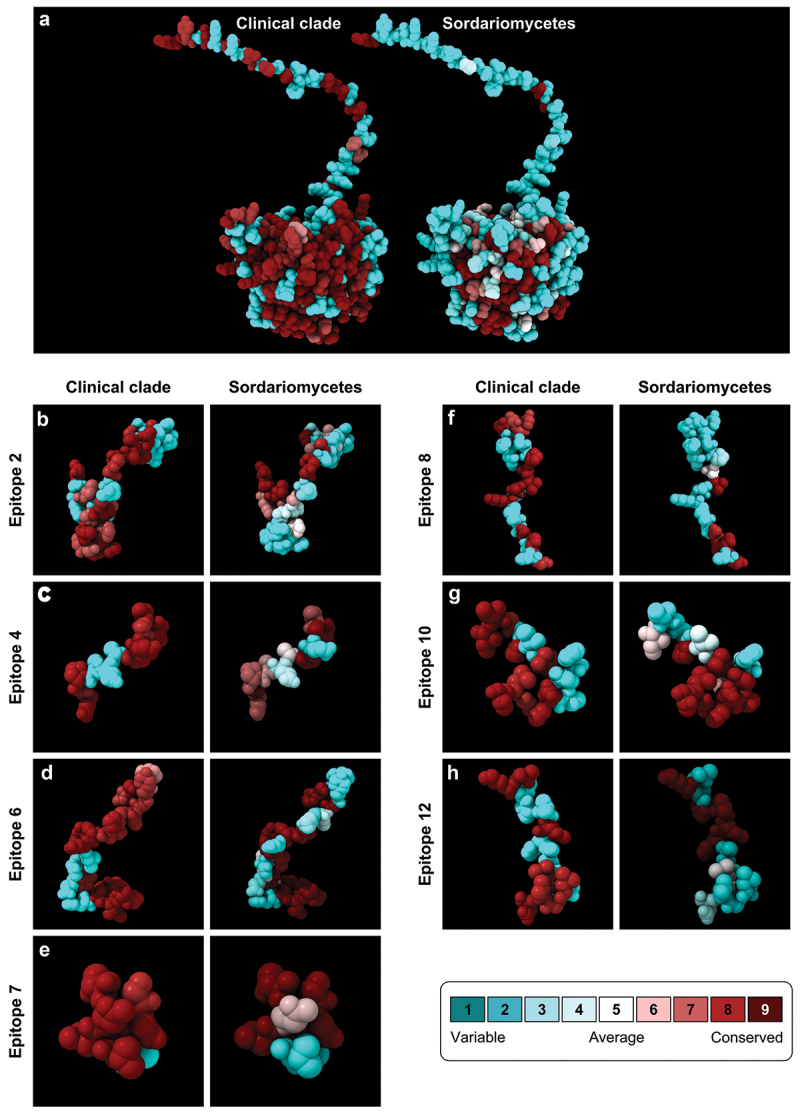
Conservation and predicted epitopes of the *CMC* protein in *Sporothrix* and *Sordariomycetes*. (a) The figure illustrates the predicted structure of the *CMC*, the conservation patterns, and the predicted B-cell epitopes within the *CMC* protein across the *Sporothrix* clinical clade (left panels) and the broader *Sordariomycetes* (right panels). (b–h) Each panel depicts a linear B-cell epitope sequence with corresponding positions in the *CMC* protein. Amino acid conservation scores are visualized via a color gradient, where dark red indicates the highest conservation, and dark blue indicates the lowest conservation. This reveals the extent to which each epitope is conserved across the *Sporothrix* clinical clade (b–d) or the wider *Sordariomycetes* (f–h). Additional information is provided in Table S3.

Importantly, *S. brasiliensis* presented the highest antigenicity scores in both the Vaxijen (0.720 ± 0.016) and AntigenPro (0.927 ± 0.009) analyses. This finding was consistent across *Sporothrix* samples from various geographic locations (Table S1). The combined analysis of all *Sporothrix* species in both Vaxijen (0.702 ± 0.034) and AntigenPro (0.898 ± 0.023) revealed minor diversity in antigenic potential within the genus, supporting cross-reactivity among closely related members of the clinical clade. The most frequently predicted epitopes, depicted in Figure 8a, highlight *CMC*‘s unique profile as an excellent serological marker for sporotrichosis, demonstrating both cross-reactivity within the clinical clade and high specificity owing to the absence of these epitopes in other *Sordariomycetes* (Figure 8b-h).

**Figure 9.**
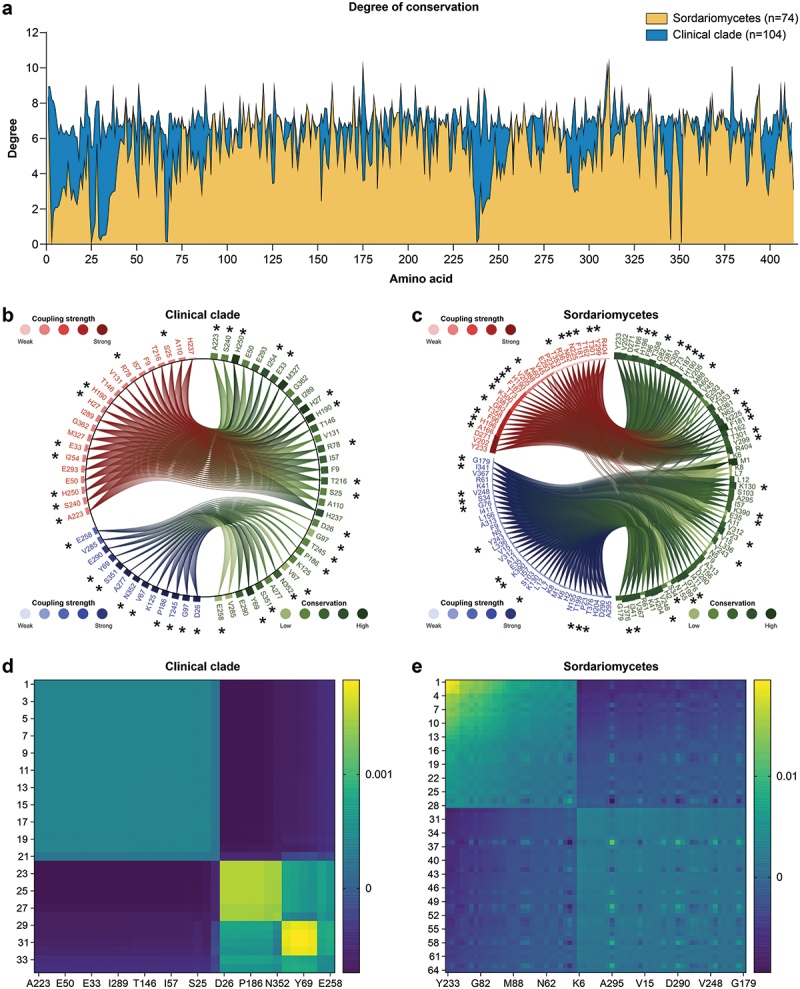
Evolutionary coupling analysis (ECA) and residue conservation in the *CMC* protein. Interconnected networks of coevolving amino acids, termed residue communities, are crucial for understanding protein structure, function, and interactions. (a) Degree of conservation at each amino acid position in the *CMC* protein, comparing the *Sporothrix* clinical clade (blue) with the broader *Sordariomycetes* (yellow). High peaks indicate highly conserved residues. (b, c) Residue communities identified within the *CMC* protein in the *Sporothrix* clinical clade (b) and the broader *Sordariomycetes* (c). Each line represents an evolutionary coupling between two residues, with thicker lines indicating stronger couplings. The color of the line indicates the coupling strength. (d, e) Spectral analysis of coupled amino acid interactions within the protein reveals both positive (yellow) and negative (blue) interactions within residue communities in the *Sporothrix* clinical clade (d) and broader *Sordariomycetes* (e). Positive interactions suggest coevolution that maintains interactions, whereas negative interactions suggest coevolution that avoids interaction. *residue communities present in B-cell epitopes.

A comparison of multiple sequence alignments revealed higher conservation levels across medically relevant *Sporothrix* species than across the broader *Sordariomycetes* throughout the *CMC* protein core (Figure 9a). Notably, the greater conservation observed in the clinical *Sporothrix* species may be partially attributed to the smaller sample size compared with the broad *Sordariomycetes*, as the number of sequences included in rate site calculations can influence conservation estimates (Figures 8b-h and 9a).

Analysis of the predicted linear B-cell epitopes across multiple *Sporothrix* strains revealed several residues also identified within the residue communities of the *CMC* protein (Figure 9b-e). These residues, including serine (S25, S240, S351), aspartic acid (D26), glutamic acid (E33, E258), glycine (G97), threonine (T245), alanine (A223), tyrosine (Y69), histidine (H27, H190, H250), arginine (R78), proline (P186), isoleucine (I254), asparagine (N352), and valine (V67), are present in two networks of coevolving residues with strong evolutionary couplings. Identifying these residues within the predicted epitopes and the residue communities underscores their potential importance as targets for immune recognition and therapeutic intervention. Interestingly, the distribution of these residues differed between medically relevant *Sporothrix* species (Figure 9b,d) and the broader *Sordariomycetes* (Figure 9c,e), suggesting *Sporothrix*-specific adaptations. Remarkably, whereas ABCpred analysis using artificial neural networks is limited to predicting linear epitopes, the residues highlighted by LERI analysis may play a role in forming conformational epitopes that cannot be identified by linear epitope prediction tools or even critical structural and functional domains within Gp60–70.

### 3.7. Analysis of potential N-, O-, and C-linked glycosylation sequons in Gp60–70

Given the known influence of *N*-linked carbohydrates on antigenicity, we predicted potential *N*-glycosylation sequons in Gp60–70. Our analysis via GlycoEP identified three highly conserved *N*-glycosylation sequons across *S. brasiliensis*, *S. schenckii*, *S. globosa*, and *S. luriei*, specifically at position 62 (NGS, 98.08% conserved; 102/104), 225 (NNT, 100.00% conserved; 104/104), and 373/374 (NGT, 99.04%, conserved; 103/104). These conservancy analysis results underscore the close evolutionary relationships among medically relevant *Sporothrix* and suggest that these sequons may be relevant for protein function. Additionally, *S. globosa* and *S. luriei* presented two additional sequons at positions 190/168 (NQT, 22.12% conserved; 23/104) and 258/236 (NGT, 22.12% conserved; 23/104) (Table S4). Notably, all putative glycosylation sequons in members of the clinical clade were found within or bordering the predicted linear B-cell epitopes, for example, in epitopes #2 (pos. 62: NGS), #7 (pos. 225: NNT), and #11 (pos. 373/374: NGT) (Table S3). Moreover, GlycoEP analysis of 104 medically relevant *Sporothrix* sequences revealed no potential *O*-linked or *C*-linked glycosylation sequons (SVM threshold: 0.0). In environmental *Sordariomycetes*, minimum conservation was noted at position 62 (NGS, 14.49%; 10/69), whereas partial conservation was noted at positions 225 (NNT, 42.03%; 29/69) and 373/374 (NGT, 86.96%; 60/69). However, most potential *N*-glycosylation sequons were unique to these environmental species (Table S5), suggesting a distinct evolutionary trajectory, adaptations to specific environments, or specialised interactions with hosts. Furthermore, GlycoEP analysis revealed no putative *O*-linked or *C*-linked glycosylation sequons in the *Sordariomycetes* dataset, except for a single sequence from *Xylaria curta* (accession KAI0545507) that exhibited a putative *C*-linked glycosylation sequon at position 299 W, with a score of 0.0034187883.

## 4. Discussion

The β-ketoadipate pathway is a sequence of biochemical reactions that certain bacteria and fungi use to break down complex aromatic structures, such as lignin (a component of plant cell walls) and polyaromatic hydrocarbons (Stainer and Ornston 1973; Michielse et al. 2012). This degradation pathway not only serves as a source of carbon and energy for these organisms but also plays a crucial ecological role in breaking down harmful aromatic pollutants (*e.g*., nitrophenols, organophosphates, polychlorinated biphenyls, polycyclic aromatic hydrocarbons, benzene, toluene, ethylbenzene, and xylene compounds) (Wells and Ragauskas 2012).

A comprehensive genetic investigation of *Sporothrix*, focusing on the *CMC* gene – a key enzyme within the β-ketoadipate pathway – reveals its potential as a marker for understanding the diversity and evolution of these *Ophiostomatales*. In medically relevant *Sporothrix*, *CMC* functions as an exocellular glycoprotein (Gp60–70), acting as a significant adhesin or antigen that triggers immune responses in humans and animals (Nascimento et al. 2008; Teixeira et al. 2009; Castro et al. 2013; Rodrigues et al. 2015b, 2015c; Martínez-Álvarez et al. 2019). *CMC*‘s dual functionality – environmental degradation and pathogenic interaction – has led researchers to hypothesise that the current habitat shifts within *S. brasiliensis* from plants to cats in southeastern Brazil, potentially explaining its emergence as a pathogen (Rodrigues et al. 2013b, 2014). This adaptation to virulence mirrors a similar pattern observed in *Blastomyces* spp. (Finkel-Jimenez et al. 2002; McBride et al. 2019). The multifaceted role of *CMC* underscores its importance for future research and potential applications in disease surveillance, diagnostics, and therapeutic development.

The elevated polymorphism observed in the *CMC* gene, notably within *S. brasiliensis* and *S. schenckii*, as evidenced by high haplotype and nucleotide diversity (Table 2) and a negative Fu’s Fs statistic, suggests the potential influence of positive selection or recent population expansion in these species (Rangel-Gamboa et al. 2016). This observation is further supported by the high levels of genetic differentiation (*Gst*, *GammaSt*, and *Fst*) between *S. brasiliensis* and *S. schenckii* or *S. globosa* (Table 3), implying limited gene flow and a shared evolutionary history. This finding aligns chronologically with the emergence of sporotrichosis in Rio de Janeiro during the mid-1990s (de Lima Barros et al. 2001; Barros et al. 2004; Schubach et al. 2005a, 2005b; Rodrigues et al. 2013b). This first wave of CTS emergence predominantly occurred in areas characterised by deforestation, poor sanitation, and high pollution levels (Fernandes et al. 2002; Gutierrez-Galhardo et al. 2015; Freire et al. 2020; Rodrigues et al. 2022a), reflecting the socioecological conditions of the city’s urban environment at that time (IBGE 1991).

We hypothesise that the abundance of aromatic pollutants in urban environments may have exerted selective pressure on *Sporothrix* populations, favouring strains with increased *CMC* activity. This hypothesis is supported by the findings of Martínez-Álvarez et al. (2019), who successfully expressed a recombinant, nonglycosylated version of Gp70 (rGp70) in *E. coli* and confirmed its enzymatic activity as a *CMC*, further highlighting the potential of *CMC* as a selective advantage in polluted environments. *CMC* activity is known to benefit saprophytic fungi such as white rot (*e.g*., *Phanerochaete chrysosporium*) and brown-rot fungi (*e.g*., *Gloeophyllum trabeum*), as well as soil fungi such as *Aspergillus* and *Penicillium* (Cameron et al. 2000; Haritash and Kaushik 2009; Harms et al. 2011). This selective advantage could explain the genetic diversity and fast expansion of *S. brasiliensis* and *S. schenckii* populations (de Carvalho et al. 2022; Losada et al. 2023), particularly in polluted urban environments. In contrast, the greater genetic differentiation observed between *S. globosa* and the other two species (Table 3), coupled with the lower intraspecific diversity observed in *S. globosa* (Table 2), may reflect distinct evolutionary trajectories or ecological niches (Zhang et al. 2015; Moussa et al. 2017; Zhao et al. 2017), potentially with less exposure to or reliance on aromatic pollutants for survival and growth.

Furthermore, we propose that epizootic (animal-to-animal) and zoonotic (animal-to-human) spread events involving cats are significant factors in the emergence of *S. brasiliensis* (Rodrigues et al. 2014). CTS events allow for rapid expansion of the *Sporothrix* population, particularly among susceptible individuals (Rodrigues et al. 2016b; Losada et al. 2023). The high density of cat populations and socioeconomic challenges in affected areas likely contribute to the spread of the disease (Alzuguir et al. 2020; Rodrigues et al. 2022a; de Oliveira et al. 2024). Consequently, the convergence of environmentally selective pressures and cat-mediated population expansion appear to be crucial triggers for *S. brasiliensis* outbreaks (Rodrigues et al. 2022a; Pinheiro et al. 2024). This genetic signature of positive selection (high-frequency derived alleles) or recent population expansion (excess of rare alleles), often associated with the emergence of virulent strains or adaptation to new environments or hosts, is not exclusive to *Sporothrix*. It has been observed in various other fungal pathogens, such as the wheat pathogen *Zymoseptoria tritici*, where positive selection has been detected in genes involved in virulence and fungicide resistance (Stukenbrock and McDonald 2008), and the human pathogen *Candidozyma auris* (formerly *Candida auris*), whose recent population expansion has been linked to its global emergence and increased drug resistance (Chow et al. 2020).

We demonstrated the great diversity of the *CMC* gene, with 79 unique haplotypes identified across 104 isolates (Table 2), establishing it as a highly informative marker for species delineation and intraspecific differentiation. While the ITS1/2 + 5.8S region is the official barcoding marker for fungi (Schoch et al. 2012), including *Sporothrix* (Zhou et al. 2014), its low polymorphism can sometimes hinder accurate species discrimination, necessitating the use of secondary barcodes (Rodrigues et al. 2016a). The *CMC* gene outperforms traditional markers such as ITS and calmodulin (Table 2; Figures 2–4) (Zhang et al. 2015), thus addressing the “barcoding gap” – the discrepancy between recognised and reliably identifiable species (Meyer and Paulay 2005). This diversity underscores the value of incorporating the *CMC* gene into the existing panel of molecular markers used for *Sporothrix* characterisation, including *BT2* (Marimon et al. 2006), *CHS* (Kano et al. 2001), *EF1*-α (Rodrigues et al. 2013b), *AST*, *GPD1*, *CPLA2* and SPBR_05954 (de Souza Rabello et al. 2023).

The conserved chromosomal location of *CMC* in pathogenic *Sporothrix* species (Figure 6) and the identification of homologous genes within the broader *Sordariomycetes* (Figure 7) highlight its potential importance in fungal biology and pathogenesis. Intriguingly, *CMC* is absent in *S. mexicana* and a few related members of the environmental clade (Figure 7), which usually exhibit attenuated virulence in murine models (Arrillaga-Moncrieff et al. 2009; Rodrigues et al. 2016a). This absence, previously noted in the immunoproteomic profile of *S. mexicana* by Rodrigues et al. (2015c), is further supported by our Southern blot and BLAST search analyses. This absence in *S. mexicana* might contribute to the reduced virulence observed in the environmental clade, potentially mirroring the attenuated disease profile of *Blastomyces* strains lacking the *BAD-1* gene (Baily et al. 1991; Klein et al. 1997; Meece et al. 2010). In *Blastomyces*, the *BAD-1* gene has been shown to increase virulence in a mouse model, leading to faster mortality in infected animals (Brandhorst et al. 1999).

Interestingly, Gp70 abundance appears to be inversely correlated with virulence in *Sporothrix* isolates, with higher expression in less virulent strains (Castro et al. 2013; Fernandes et al. 2013). This observation and its role as the primary cell wall antigen suggest a dual role in pathogenesis: adhesion and immunogenicity (Nascimento and Almeida 2005; Nascimento et al. 2008; Teixeira et al. 2009). While the adhesive properties of this glycoconjugate are evident in its ability to bind to extracellular matrix proteins and contribute to fungal attachment to host tissues (Nascimento and Almeida 2005; Nascimento et al. 2008; Ruiz-Baca et al. 2009; Castro et al. 2013), its lower expression in virulent strains and recognition by antibodies from infected patients point to its importance in triggering an immune response (Nascimento and Almeida 2005; Nascimento et al. 2008; Rodrigues et al. 2015b, 2015c). Notably, passive immunisation with anti-Gp70 antibodies has been shown to confer protection against infection in a mouse model, further supporting its role in modulating host-pathogen interactions (Nascimento et al. 2008; Almeida 2012; Chen et al. 2019; Martínez-Álvarez et al. 2019).

From a serological diagnostic perspective, we investigated the impact of the observed high genetic diversity on the prediction of linear B-cell epitopes. Our data revealed that *Sporothrix CMC* contains a signal peptide across all the evaluated clinical isolates. Furthermore, the prediction of conserved linear B-cell epitopes, along with the high antigenicity scores calculated by Vaxijen and AntigenPro for *S. brasiliensis*, underscores the strong immunogenic potential of *CMC* and its relevance for developing diagnostics and vaccines for sporotrichosis (Figure 8). It also supports the cross-reactivity reported in humans (Rodrigues et al. 2015c), cats (Rodrigues et al. 2015b), and murine models (Fernandes et al. 2013; Della Terra et al. 2017). Moreover, our data revealed significant differences in the prediction of linear B-cell epitopes between members of the *Sporothrix* clinical clade and members of the class *Sordariomycetes* (Figure 8), supporting the high specificity of serological assays for the diagnosis of human and feline sporotrichosis (Bernardes-Engemann et al. 2005, 2009, 2015; Fernandes et al. 2011; Rodrigues et al. 2015b, 2015c).

We used the SAEC method to analyse coevolving amino acids in Gp60–70 via multiple sequence alignments of their *CMC* protein regions. These positions may be potentially important for immune recognition, therapeutic targeting, and, as suggested by conserved residues within B-cell epitopes and coevolving communities, protein structure and function (Cheung et al. 2021). Differences in the occurrence of these residue communities between the medically relevant *Sporothrix* and the broader *Sordariomycetes* highlight the need to study Gp60–70 *Sporothrix*-specific adaptations to mammalian pathogenicity (Figure 9b-e). These findings are consistent with recent advances in protein function prediction, which emphasise the importance of incorporating evolutionary information, such as residue communities, to enhance our understanding of protein function and identify potential therapeutic targets (Granata et al. 2017; Jang et al. 2024; Roy and Ray 2025).

In *S. schenckii*, *N*-linked glycosylation, which is characterised by key glycosidases, is vital for cell wall integrity and virulence (Lopes-Bezerra et al. 2015). This process significantly impacts the antigenicity of Gp60–70, affecting its function and host immune interactions (Ruiz-Baca et al. 2009, 2011; Félix-Contreras et al. 2020; López-Ramírez et al. 2024; Padró-Villegas et al. 2025). This modification influences immunogenicity by providing glycan epitopes and generating antigenic variability that can hinder immune recognition (Ruiz-Baca et al. 2009, 2011; Alba-Fierro et al. 2014, 2016). Additionally, glycosylation modulates host responses by affecting immune cell activation and cytokine production and may influence *Sporothrix* adhesion to host tissues (Nascimento and Almeida 2005; Teixeira et al. 2009; de Lima Franco et al. 2012; Castro et al. 2013; Martínez-Álvarez et al. 2019; López-Ramírez et al. 2024).

Our study highlights the striking diversity in the number of predicted *N*-glycosylation sequons across *Sporothrix* species, whereas no potential *O*- or *C*-linked sequons were predicted in Gp60–70. These findings are consistent with previous experimental data, which revealed that approximately 5.7% of the molecular mass of Gp70 is attributed to *N*-linked glycans, with no evidence of *O*-linked oligosaccharides (Ruiz-Baca et al. 2009). From a population-level perspective, this result suggests an evolutionary trade-off, where selective pressures shape a delicate balance between the advantage of evading the host immune response and the necessity of preserving essential functions critical for *Sporothrix* survival and dissemination (Cui et al. 2009; Gagneux et al. 2022). The shared pattern of three highly conserved *N*-glycosylation sequons within the clinical clade underscores their close evolutionary relationship and suggests that these sites may play critical roles in Gp60–70 function. In contrast, the additional glycosylation sequons found in *S. globosa* and *S. luriei* raise intriguing questions about the evolutionary forces driving these differences, as changes in glycosylation can significantly impact pathogen virulence (Marín-Menguiano et al. 2019; Liu et al. 2021).

While the presence of a sequon is a key determinant of *N*-glycosylation, it may not be sufficient to ensure that glycosylation occurs (Knauer and Lehle 1999; Dutta et al. 2017). Not all sequons are glycosylated, and not all glycosylated sites are essential for proper folding (He et al. 2024). However, in certain cases, productive protein folding and functionality rely on *N*-glycosylation at specific sites (Petrescu et al. 2006). For example, in *Ustilago maydis*, *N*-glycosylation of the protein disulphide isomerase Pdi1 plays a critical role in the folding and secretion of virulence factors, which are essential for pathogenicity (Marín-Menguiano et al. 2019). This case highlights the broader importance of *N*-glycosylation in protein function and host-pathogen interactions (Gómez-Gaviria et al. 2021).

The potential of Gp60–70 as a therapeutic target is strongly supported by its immunogenic conservation patterns across *Sporothrix* species. For example, a peptide derived from Gp70, KPVQHALLTPLGLDR, was identified and shown to elicit robust immune responses in murine models when displayed on recombinant phages, providing significant protection against *S. globosa* infections. Additionally, antibodies targeting this peptide effectively reduce fungal burden, mitigate inflammation, and improve survival rates (Chen et al. 2017, 2019). Similarly, the ZR8 peptide (LKFLALASVISATSA), derived from *S. brasiliensis* Gp60, induces a potent cellular immune response in murine models characterised by increased CD4+ T-cell populations and elevated levels of IFN-γ, IL-17A, and IL-1β (de Almeida et al. 2018).

Our population-level sequence analysis demonstrated a distinct conservancy pattern between these two peptides. The Gp70-derived peptide (Chen et al. 2017), which matches epitope 12 predicted in our analysis (Table S3), exhibits a high degree of conservation across medically relevant *Sporothrix* species, with a conservancy of 82.69% (86/104), underscoring its potential for broad-spectrum application. In contrast, the ZR8 peptide shows high conservation within *S. brasiliensis* (conservation of 87.50%; 28/32) but exhibits significant residue modifications in closely related *Sporothrix*, resulting in an overall conservancy of only 26.92% (28/104). The population-level sequences generated in our study offer a crucial framework for assessing the conservation of Gp60–70-derived peptides. This variability has significant implications for the broad-spectrum and species-specific therapeutic potential of these peptides.

The importance of Gp60–70 in *Sporothrix* pathogenesis is underscored by its multifaceted role in adhesion, cell wall integrity, and immune modulation. Silencing the *CMC* gene, which encodes Gp60–70, leads to pleiotropic effects, including alterations in adhesion, cell wall composition, virulence, the immune response, and phagocytosis (López-Ramírez et al. 2024; Padró-Villegas et al. 2025). Our findings and those of previous studies emphasise the complex relationships among evolutionary domains, coevolving residues, *N*-glycosylation sequons, B-cell epitope diversity, and the immune evasion strategies *Sporothrix* employs. This intricate interplay highlights the sophistication of the pathogen’s mechanisms for survival and dissemination (Fernandes et al. 2013; Rodrigues et al. 2015b, 2015c; Chen et al. 2017, 2019; Della Terra et al. 2017; de Almeida et al. 2018; López-Ramírez et al. 2024).

In conclusion, we demonstrate the significance of the *CMC* gene as a versatile marker for investigating the genetic diversity, evolution, and immunogenicity of *Sporothrix* species. The high polymorphism of the *CMC* gene, particularly in *S. brasiliensis* and *S. schenckii*, its conserved epitopes, *N*-glycosylation sequons, and *Sporothrix*-specific antigenic properties make it a promising candidate for developing diagnostics, vaccines, and potentially even therapeutic targets to address the emergence of sporotrichosis.

## Supplementary Material

accept-0211-Mycology_Supplementary_Files_Reviewed_v2-han.docx
