# Supplementary material for: Genetic diversity and molecular evolution of 3-carboxymuconate cyclase (Gp60–70), the major antigen in pathogenic Sporothrix species
Source: Mycology. 2025 Mar 8;16(4):1754–80. doi: 10.1080/21501203.2025.2467118 (PMC12667341; doi:10.1080/21501203.2025.2467118)
Supplement: accept-0211-Mycology_Supplementary_Files_Reviewed_v2-han.docx [file TMYC_A_2467118_SM9039.docx]

**Supplementary Table S1.** *Sporothrix* species isolates and sequences used in this study.

| **Isolate** | **Other code** | **Species** | **Source** | **Origin** | **GenBank Accession codes** | | | **Antigenicity prediction** | | **Ref.** |
| --- | --- | --- | --- | --- | --- | --- | --- | --- | --- | --- |
|  |  |  |  |  | ***CAL*** | ***ITS*** | ***GP70*** | **VaxiJen** | **AntigenPro** |  |
| Ss05 | CBS 132985 | *S. brasiliensis* | Feline sporotrichosis | Minas Gerais, Brazil | KC693830 | KF961142 | PP826558 | 0.7244 | 0.936199 | [1-3] |
| Ss07 | CBS 132986 | *S. brasiliensis* | Human sporotrichosis | Minas Gerais, Brazil | KC693831 | KF961143 | PP826559 | 0.7244 | 0.936199 | [1-3] |
| Ss99 | - | *S. brasiliensis* | Human sporotrichosis | Rio de Janeiro, Brazil | KF574460 | KF574442 | PP826560 | 0.7244 | 0.936199 | [1-3] |
| 5110 | ATCC 4823 | *S. brasiliensis* | Feline sporotrichosis | Rio de Janeiro, Brazil | JF313351 | JQ070114 | AWTV00000000 | 0.7405 | 0.936199 | [4] |
| Ss14 | - | *S. brasiliensis* | Human sporotrichosis | Minas Gerais, Brazil | KF943632 | KF961145 | PP826561 | 0.7244 | 0.936199 | [1-3] |
| Ss33 | - | *S. brasiliensis* | Human sporotrichosis | Paraná, Brazil | KF943637 | KF961146 | PP826562 | 0.7244 | 0.936199 | [1-3] |
| Ss354 | - | *S. brasiliensis* | Feline sporotrichosis | São Paulo, Brazil | N/A | N/A | PP826563 | 0.7244 | 0.936199 | [1-3] |
| Ss104 | - | *S. brasiliensis* | Human sporotrichosis | Mato Grosso, Brazil | KF574461 | KF574443 | PP826564 | 0.7259 | 0.930274 | [1-3] |
| Ss245 | CBS 133005 | *S. brasiliensis* | Feline sporotrichosis | Rio de Janeiro, Brazil | KC693878 | N/A | PP826565 | 0.7244 | 0.936199 | [1-3] |
| Ss250 | CBS 133009 | *S. brasiliensis* | Feline sporotrichosis | Rio de Janeiro, Brazil | KC693883 | N/A | PP826566 | 0.7244 | 0.936199 | [1-3] |
| Ss52 | - | *S. brasiliensis* | Human sporotrichosis | São Paulo, Brazil | KC693845 | KF574444 | PP826567 | 0.7244 | 0.936199 | [1-3] |
| Ss54 | CBS 132990 | *S. brasiliensis* | Feline sporotrichosis | Rio Grande do Sul, Brazil | JQ041903 | JN885580 | PP826568 | 0.7244 | 0.936199 | [1-3] |
| Ss178 | CBS 120339 | *S. brasiliensis* | Human sporotrichosis | Rio de Janeiro, Brazil | AM116898 | KF574440 | PP826569 | 0.7215 | 0.929872 | [1-3] |
| Ss177 | FMR8309 | *S. brasiliensis* | Human sporotrichosis | Rio de Janeiro, Brazil | AM116899 | KF574441 | PP826570 | 0.7215 | 0.929872 | [1-3] |
| Ss252 | CBS 133011 | *S. brasiliensis* | Feline sporotrichosis | Rio de Janeiro, Brazil | KC693885 | N/A | PP826571 | 0.7171 | 0.924127 | [1-3] |
| Ss38 | - | *S. brasiliensis* | Human sporotrichosis | Paraná, Brazil | KC693844 | KF961149 | PP826572 | 0.7388 | 0.915264 | [1-3] |
| Ss333 | - | *S. brasiliensis* | Feline sporotrichosis | São Paulo, Brazil | N/A | N/A | PP826573 | 0.7244 | 0.936199 | [1-3] |
| Ss248 | CBS 133007 | *S. brasiliensis* | Feline sporotrichosis | Rio de Janeiro, Brazil | KC693881 | N/A | PP826574 | 0.7162 | 0.921583 | [1-3] |
| Ss246 | CBS 133002 | *S. brasiliensis* | Feline sporotrichosis | Rio de Janeiro, Brazil | KC693879 | N/A | PP826575 | 0.7380 | 0.917191 | [1-3] |
| Ss254 | CBS 133013 | *S. brasiliensis* | Feline sporotrichosis | Rio de Janeiro, Brazil | KJ020534 | N/A | PP826576 | 0.7277 | 0.917703 | [1-3] |
| Ss44 | - | *S. brasiliensis* | Human sporotrichosis | Ceará, Brazil | KF943641 | KF961151 | PP826577 | 0.7277 | 0.917703 | [1-3] |
| Ss57 | - | *S. brasiliensis* | Human sporotrichosis | Rio Grande do Sul, Brazil | KF943645 | KF961154 | PP826578 | 0.7204 | 0.900662 | [1-3] |
| Ss360 | - | *S. brasiliensis* | Feline sporotrichosis | São Paulo, Brazil | N/A | N/A | PP826579 | 0.7244 | 0.936199 | [1-3] |
| Ss338 | - | *S. brasiliensis* | Feline sporotrichosis | São Paulo, Brazil | N/A | N/A | PP826580 | 0.7019 | 0.930353 | [1-3] |
| Ss10 | CBS 132987 | *S. brasiliensis* | Human sporotrichosis | Minas Gerais, Brazil | KC693834 | N/A | PP826581 | 0.7290 | 0.923423 | [1-3, 5] |
| Ss332 | - | *S. brasiliensis* | Feline sporotrichosis | São Paulo, Brazil | N/A | N/A | PP826582 | 0.7345 | 0.920563 | [1-3, 5] |
| Ss265 | CBS 133020 | *S. brasiliensis* | Human sporotrichosis | Minas Gerais, Brazil | JN204360 | KF574445 | PP826583 | 0.7346 | 0.918801 | [1-3, 5] |
| Ss226 | CBS 133003 | *S. brasiliensis* | Human sporotrichosis | São Paulo, Brazil | KC693875 | N/A | PP826584 | 0.6993 | 0.919196 | [1-3, 5] |
| Ss172 | CBS 133000 | *S. brasiliensis* | Feline sporotrichosis | Paraná, Brazil | KC693872 | N/A | PP826585 | 0.7051 | 0.914054 | [1-3, 5] |
| Ss174 | CBS 133002 | *S. brasiliensis* | Feline sporotrichosis | Paraná, Brazil | KC693874 | N/A | PP826586 | 0.7018 | 0.921969 | [1-3, 5] |
| Ss171 | CBS 132999 | *S. brasiliensis* | Feline sporotrichosis | Paraná, Brazil | KC693871 | N/A | PP826587 | 0.7018 | 0.921969 | [1-3, 5] |
| Ss34 | - | *S. brasiliensis* | Human sporotrichosis | Paraná, Brazil | KF943638 | KF961147 | PP826588 | 0.6580 | 0.924786 | [1-3, 5] |
| ATCC58251 | - | *S. schenckii* | Human sporotrichosis | Puerto Rico | AWEQ01 | AWEQ01 | AWEQ01 | 0.6768 | 0.882802 | [6] |
| Ss478 | - | *S. schenckii* | Human sporotrichosis | Mexico | N/A | N/A | PP826589 | 0.6640 | 0.891889 | [1-3, 5] |
| Ss530 | - | *S. schenckii* | Human sporotrichosis | Mexico | N/A | N/A | PP826590 | 0.6616 | 0.906531 | [1-3, 5] |
| Ss118 | CBS 132974 | *S. schenckii* | Human sporotrichosis | São Paulo, Brazil | JX077126 | KF961174 | PP826591 | 0.6644 | 0.886361 | [1-3, 5] |
| Ss234 | - | *S. schenckii* | Human sporotrichosis | Peru | KF943716 | N/A | PP826592 | 0.6612 | 0.882802 | [1-3, 5] |
| Ss577 | - | *S. schenckii* | Human sporotrichosis | Mexico | N/A | N/A | PP826593 | 0.6290 | 0.893712 | [1-3, 5] |
| Ss24 | - | *S. schenckii* | Human sporotrichosis | Paraná, Brazil | KC693839 | N/A | PP826594 | 0.6624 | 0.906135 | [1-3, 5] |
| Ss185 | CBS 359.36 | *S. schenckii* | Human sporotrichosis | USA | AM117437 | FJ545232 | PP826595 | 0.6612 | 0.882802 | [1-3, 5] |
| S7 | PWQ1508 | *S. schenckii* | Human sporotrichosis | Australia | SRR6655911 | SRR6655911 | SRR6655911 | 0.6702 | 0.882802 | [7] |
| Ss143 | - | *S. schenckii* | Human sporotrichosis | Pará, Brazil | JQ041906 | JN885583 | PP826596 | 0.6625 | 0.880497 | [1-3, 5] |
| 1099-18 | ATCC 4821 | *S. schenckii* | Human sporotrichosis | USA | KF574470 | JQ070112 | AXCR00000000 | 0.6769 | 0.882802 | [4] |
| Ss476 | - | *S. schenckii* | Human sporotrichosis | Mexico | N/A | N/A | PP826597 | 0.6490 | 0.882264 | [1-3, 5] |
| Ss477 | - | *S. schenckii* | Human sporotrichosis | Mexico | N/A | N/A | PP826598 | 0.6640 | 0.891889 | [1-3, 5] |
| Ss176 | CDM18 | *S. schenckii* | Human sporotrichosis | Italy | N/A | N/A | PP826599 | 0.6490 | 0.882264 | [1-3, 5] |
| Ss529 | - | *S. schenckii* | Human sporotrichosis | Mexico | N/A | N/A | PP826600 | 0.6722 | 0.886566 | [1-3, 5] |
| Ss39 | - | *S. schenckii* | Human sporotrichosis | Paraná, Brazil | JQ041899 | JN885576 | PP826601 | 0.6593 | 0.883244 | [1-3, 5] |
| Ss64 | - | *S. schenckii* | Human sporotrichosis | Espírito Santo, Brazil | JX077124 | KF961169 | PP826602 | 0.6646 | 0.887126 | [1-3, 5] |
| S6 | PWQ3030 | *S. schenckii* | Human sporotrichosis | Australia | SRR6655907 | SRR6655907 | SRR6655907 | 0.6686 | 0.883177 | [7] |
| Ss527 | - | *S. schenckii* | Human sporotrichosis | Mexico | N/A | N/A | PP826603 | 0.6450 | 0.897495 | [1-3, 5] |
| Ss162 | CBS 132977 | *S. schenckii* | Vegetal | México | KF574467 | KF574453 | PP826604 | 0.6475 | 0.906837 | [1-3, 5] |
| Ss164 | - | *S. schenckii* | Human sporotrichosis | Peru | KF574469 | KF574455 | PP826605 | 0.6612 | 0.882802 | [1-3, 5] |
| Ss229 | - | *S. schenckii* | Human sporotrichosis | Peru | KF943712 | N/A | PP826606 | 0.6612 | 0.882802 | [1-3, 5] |
| Ss231 | - | *S. schenckii* | Human sporotrichosis | Peru | KF943714 | N/A | PP826607 | 0.6612 | 0.882802 | [1-3, 5] |
| Ss228 | - | *S. schenckii* | Human sporotrichosis | Peru | KF943711 | N/A | PP826608 | 0.6612 | 0.882802 | [1-3, 5] |
| Ss554 | - | *S. schenckii* | Human sporotrichosis | Mexico | N/A | N/A | PP826609 | 0.6534 | 0.897967 | [1-3, 5] |
| Ss447 | - | *S. schenckii* | Human sporotrichosis | Venezuela | N/A | N/A | PP826610 | 0.6625 | 0.883948 | [1-3, 5] |
| Ss175 | SPO1 | *S. schenckii* | Human sporotrichosis | Italy | N/A | N/A | PP826611 | 0.6909 | 0.893634 | [1-3, 5] |
| S13 | PWQ3048 | *S. schenckii* | Human sporotrichosis | Australia | SRR6655897 | SRR6655897 | SRR6655897 | 0.6714 | 0.906135 | [7] |
| Ss564 | - | *S. schenckii* | Human sporotrichosis | Mexico | N/A | N/A | PP826612 | 0.6516 | 0.884193 | [1-3, 5] |
| Ss567 | - | *S. schenckii* | Human sporotrichosis | Mexico | N/A | N/A | PP826613 | 0.6516 | 0.884193 | [1-3, 5] |
| Ss59 | - | *S. schenckii* | Human sporotrichosis | São Paulo, Brazil | KF943647 | KF961168 | PP826614 | 0.6680 | 0.869443 | [1-3, 5] |
| Ss551 | - | *S. schenckii* | Human sporotrichosis | Mexico | N/A | N/A | PP826615 | 0.6462 | 0.920492 | [1-3, 5] |
| Ss13 | - | *S. schenckii* | Human sporotrichosis | Minas Gerais, Brazil | KC693836 | KF961157 | PP826616 | 0.7058 | 0.877922 | [1-3, 5] |
| Ss124 | - | *S. schenckii* | Human sporotrichosis | São Paulo, Brazil | KF943687 | KF961176 | PP826617 | 0.6913 | 0.869754 | [1-3, 5] |
| Ss137 | - | *S. schenckii* | Human sporotrichosis | Pernambuco, Brazil | KF574462 | KF574448 | PP826618 | 0.7018 | 0.871437 | [1-3, 5] |
| Ss46 | - | *S. schenckii* | Human sporotrichosis | Goiás, Brazil | KF943642 | KF961166 | PP826619 | 0.6914 | 0.879955 | [1-3, 5] |
| Ss17 | - | *S. schenckii* | Human sporotrichosis | Paraná, Brazil | KC693838 | KF961160 | PP826620 | 0.6951 | 0.871764 | [1-3, 5] |
| SsMS1 | - | *S. schenckii* | Human sporotrichosis | Colombia | PGUU01 | PGUU01 | PGUU01 | 0.7088 | 0.888532 | [8] |
| SsEM7 | - | *S. schenckii* | Human sporotrichosis | Colombia | NTMI01 | NTMI01 | NTMI01 | 0.7038 | 0.871764 | [8] |
| Ss126 | - | *S. schenckii* | Human sporotrichosis | São Paulo, Brazil | JQ041904 | JN885581 | PP826621 | 0.6855 | 0.884715 | [1-3, 5] |
| Ss241 | - | *S. schenckii* | Human sporotrichosis | São Paulo, Brazil | KF943705 | N/A | PP826622 | 0.6951 | 0.871764 | [1-3, 5] |
| Ss40 | - | *S. schenckii* | Human sporotrichosis | Ceará, Brazil | JQ041900 | JN885577 | PP826623 | 0.6973 | 0.866486 | [1-3, 5] |
| Ss243 | - | *S. schenckii* | Soil | São Paulo, Brazil | KJ020359 | KF961179 | PP826624 | 0.6973 | 0.866486 | [1-3, 5] |
| Ss15 | - | *S. schenckii* | Human sporotrichosis | Minas Gerais, Brazil | KC693837 | KF961158 | PP826625 | 0.6620 | 0.875799 | [1-3, 5] |
| Ss205 | - | *S. schenckii* | Human sporotrichosis | São Paulo, Brazil | KJ542535 | N/A | PP826626 | 0.6595 | 0.886380 | [1-3, 5] |
| Ss22 | CBS 132964 | *S. schenckii* | Human sporotrichosis | Paraná, Brazil | KF943634 | KF961162 | PP826627 | 0.6620 | 0.875799 | [1-3, 5] |
| Ss61 | - | *S. schenckii* | Soil | São Paulo, Brazil | KF561244 | KF574447 | PP826628 | 0.6642 | 0.866392 | [1-3, 5] |
| Ss468 | - | *S. schenckii* | Human sporotrichosis | São Paulo, Brazil | N/A | N/A | PP826629 | 0.7237 | 0.907029 | [1-3, 5] |
| Ss51 | - | *S. schenckii* | Human sporotrichosis | Pará, Brazil | JQ041902 | JN885579 | PP826630 | 0.7253 | 0.917174 | [1-3, 5] |
| Ss467 | - | *S. globosa* | Human sporotrichosis | São Paulo, Brazil | N/A | N/A | PP826631 | 0.7417 | 0.828427 | [1-3, 5] |
| Ss449 | C199 | *S. globosa* | Human sporotrichosis | Venezuela | N/A | N/A | PP826632 | 0.7335 | 0.883552 | [1-3, 5] |
| Ss445 | C8775 | *S. globosa* | Human sporotrichosis | Venezuela | KF478901 | KJ999896 | PP826633 | 0.7335 | 0.883552 | [1-3, 5] |
| Ss456 | C8981 | *S. globosa* | Human sporotrichosis | Venezuela | KF478905 | KJ999900 | PP826634 | 0.7335 | 0.883552 | [1-3, 5] |
| Ss457 | 3676 | *S. globosa* | Human sporotrichosis | Venezuela | N/A | N/A | PP826635 | 0.7335 | 0.883552 | [1-3, 5] |
| Ss443 | C9887 | *S. globosa* | Human sporotrichosis | Venezuela | KF478908 | KJ999905 | PP826636 | 0.7335 | 0.883552 | [1-3, 5] |
| Ss06 | CBS 132922 | *S. globosa* | Human sporotrichosis | Minas Gerais, Brazil | JF811336 | JN885574 | PP826637 | 0.7335 | 0.883552 | [1-3, 5] |
| Ss446 | C0329 | *S. globosa* | Human sporotrichosis | Venezuela | KF478892 | KJ999878 | PP826638 | 0.7335 | 0.883552 | [1-3, 5] |
| Ss180 | CBS 130104 | *S. globosa* | Human sporotrichosis | Spain | AM116905 | KC113225 | PP826639 | 0.7477 | 0.883552 | [1-3, 5] |
| CBS120340 | FMR 8600 | *S. globosa* | Human sporotrichosis | Spain | AM116908 | FN549905 | PP826640 | 0.7418 | 0.883552 | [1-3, 5] |
| Ss471 | - | *S. globosa* | Human sporotrichosis | Chile | N/A | N/A | PP826641 | 0.7335 | 0.883552 | [1-3, 5] |
| Ss472 | - | *S. globosa* | Soil | Chile | N/A | N/A | PP826642 | 0.7335 | 0.883552 | [1-3, 5] |
| Ss236 | CBS 132925 | *S. globosa* | Human sporotrichosis | Minas Gerais, Brazil | KC693877 | KF961181 | PP826643 | 0.7335 | 0.883552 | [1-3, 5] |
| Ss520 | - | *S. globosa* | Human sporotrichosis | Rio de Janeiro, Brazil | N/A | N/A | PP826644 | 0.7348 | 0.888016 | [1-3, 5] |
| 5659 | - | *S. globosa* | Human sporotrichosis | Venezuela | N/A | N/A | PP826645 | 0.7475 | 0.892476 | [1-3, 5] |
| Ss41 | CBS 132923 | *S. globosa* | Human sporotrichosis | Minas Gerais, Brazil | JF811337 | KF574456 | PP826646 | 0.7348 | 0.881595 | [1-3, 5] |
| Ss334 | - | *S. globosa* | Feline sporotrichosis | São Paulo, Brazil | N/A | N/A | PP826647 | 0.7348 | 0.881595 | [1-3, 5] |
| Ss49 | CBS 132924 | *S. globosa* | Human sporotrichosis | Minas Gerais, Brazil | JF811338 | KF961180 | PP826648 | 0.7319 | 0.887450 | [1-3, 5] |
| S17 | PWQ3063 | *S. globosa* | Human sporotrichosis | Australia | SRR6655893 | SRR6655893 | SRR6655893 | 0.7427 | 0.883552 | [7] |
| SS01 | - | *S. globosa* | Human sporotrichosis | China | LVYX01 | LVYX01 | LVYX01 | 0.7335 | 0.883552 | [9] |
| Ss534 | - | *S. globosa* | Human sporotrichosis | Mexico | N/A | N/A | PP826649 | 0.7680 | 0.902811 | [1-3, 5] |
| Ss545 | - | *S. globosa* | Human sporotrichosis | Mexico | N/A | N/A | PP826650 | 0.7720 | 0.910828 | [1-3, 5] |
| Ss187 | CBS 937.72 | *S. luriei* | Human sporotrichosis | South Africa | AM747302 | AB128012 | PP826651 | 0.7538 | 0.856939 | [1-3, 5] |

**Supplementary Table S2.** Genetic differentiation and gene flow estimates for populations *Sporothrix brasiliensis*, *S. schenckii*, and *S. globosa*.

| **Estimate** | **Value** | ***P*-value** | **Notes** |
| --- | --- | --- | --- |
| Chi-square (Chi^2^) | 206.000 | 0.0023 ** | df = 152 |
| *Hs* | 0.96450 | 0.0000 *** | HBK 1992 |
| *Hst* | 0.02585 | 0.0000 *** | HBK 1992 |
| *Ks* | 14.78394 | 0.0000 *** | HBK 1992 |
| *Kst* | 0.60670 | 0.0000 *** | HBK 1992 |
| *Ks** | 2.24882 | 0.0000 *** | HBK 1992 |
| *Kst** | 0.33305 | 0.0000 *** | HBK 1992 |
| *Z* | 951.39517 | 0.0000 *** | HBK 1992 |
| *Z** | 6.44400 | 0.0000 *** | HBK 1992 |
| *Gst* | 0.02732 | - | Nei 1973 |
| *Nm (Gst)* | 8.90 | - | Gene Flow (Haplotype Data) |
| *GammaSt* | 0.61250 | - | Nei 1982 |
| *Nm (GammaSt)* | 0.16 | - | Gene Flow (Sequence Data) |
| *Fst* | 0.74852 | - | Hudson, Slatkin & Maddison 1992 |
| *Nm (Fst)* | 0.08 | - | Gene Flow (Sequence Data) |

*P*-values are represented as **: 0.001 < *P* < 0.01, and ***: *P* < 0.001.

**Supplementary Table S3.** BepiPred 2.0 results.

| **Protein name: 3-carboxymuconate cyclase (Gp60–70)**  **Predicted peptides: Ss05**   | **No.** | **Start** | **End** | **Peptide** | **Length** | | --- | --- | --- | --- | --- | | 1 | 23 | 38 | PTSDHYYADAESEACL | 16 | | 2 | 62 | 98 | NGSLLVNYATSTATGGRGGNGINPRGMPAGPDALFGQ | 37 | | 3 | 127 | 129 | DPT | 3 | | 4 | 133 | 143 | VVGEPAELPGE | 11 | | 5 | 165 | 167 | KAG | 3 | | 6 | 173 | 199 | YSWYGLGPFDELRPFDLHQTTPPHGPT | 27 | | 7 | 220 | 227 | GDPAVNNT | 8 | | 8 | 238 | 259 | IHSSCYATPSVSHKGVISSPEG | 22 | | 9 | 297 | 305 | LYKTVIPGQ | 9 | | 10 | 340 | 357 | IIGEPIDLTTFSNDPGLT | 18 | | 11 | 374 | 375 | GT | 2 | | 12 | 387 | 402 | TKKPVQHALLTPLGLD | 16 | | **BepiPred Linear Epitope Prediction 2.0**  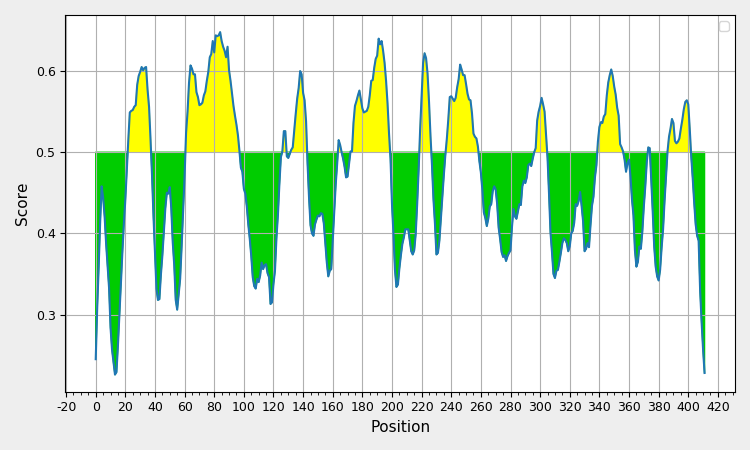 |
| --- | --- | --- | --- | --- | --- | --- | --- | --- | --- | --- | --- | --- | --- | --- | --- | --- | --- | --- | --- | --- | --- | --- | --- | --- | --- | --- | --- | --- | --- | --- | --- | --- | --- | --- | --- | --- | --- | --- | --- | --- | --- | --- | --- | --- | --- | --- | --- | --- | --- | --- | --- | --- | --- | --- | --- | --- | --- | --- | --- | --- | --- | --- | --- | --- | --- | --- |

| **Protein name: 3-carboxymuconate cyclase (Gp60–70)**  **Predicted peptides: Ss07**   | **No.** | **Start** | **End** | **Peptide** | **Length** | | --- | --- | --- | --- | --- | | 1 | 23 | 38 | PTSDHYYADAESEACL | 16 | | 2 | 62 | 98 | NGSLLVNYATSTATGGRGGNGINPRGMPAGPDALFGQ | 37 | | 3 | 127 | 129 | DPT | 3 | | 4 | 133 | 143 | VVGEPAELPGE | 11 | | 5 | 165 | 167 | KAG | 3 | | 6 | 173 | 199 | YSWYGLGPFDELRPFDLHQTTPPHGPT | 27 | | 7 | 220 | 227 | GDPAVNNT | 8 | | 8 | 238 | 259 | IHSSCYATPSVSHKGVISSPEG | 22 | | 9 | 297 | 305 | LYKTVIPGQ | 9 | | 10 | 340 | 357 | IIGEPIDLTTFSNDPGLT | 18 | | 11 | 374 | 375 | GT | 2 | | 12 | 387 | 402 | TKKPVQHALLTPLGLD | 16 | | **BepiPred Linear Epitope Prediction 2.0**  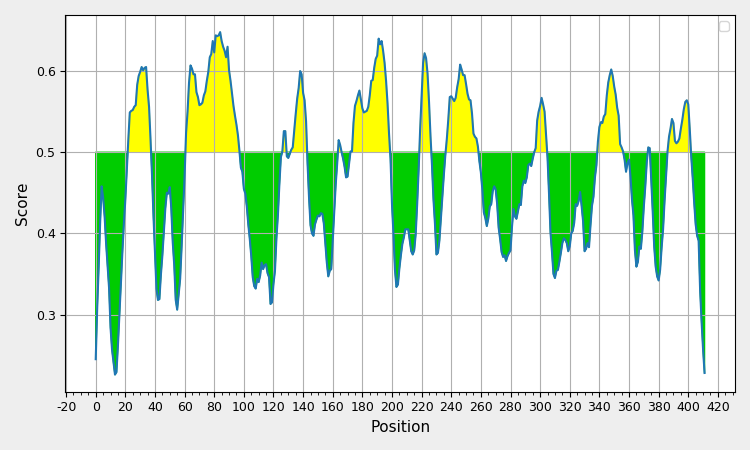 |
| --- | --- | --- | --- | --- | --- | --- | --- | --- | --- | --- | --- | --- | --- | --- | --- | --- | --- | --- | --- | --- | --- | --- | --- | --- | --- | --- | --- | --- | --- | --- | --- | --- | --- | --- | --- | --- | --- | --- | --- | --- | --- | --- | --- | --- | --- | --- | --- | --- | --- | --- | --- | --- | --- | --- | --- | --- | --- | --- | --- | --- | --- | --- | --- | --- | --- | --- |

| **Protein name: 3-carboxymuconate cyclase (Gp60–70)**  **Predicted peptides: Ss99**   | **No.** | **Start** | **End** | **Peptide** | **Length** | | --- | --- | --- | --- | --- | | 1 | 23 | 38 | PTSDHYYADAESEACL | 16 | | 2 | 62 | 98 | NGSLLVNYATSTATGGRGGNGINPRGMPAGPDALFGQ | 37 | | 3 | 127 | 129 | DPT | 3 | | 4 | 133 | 143 | VVGEPAELPGE | 11 | | 5 | 165 | 167 | KAG | 3 | | 6 | 173 | 199 | YSWYGLGPFDELRPFDLHQTTPPHGPT | 27 | | 7 | 220 | 227 | GDPAVNNT | 8 | | 8 | 238 | 259 | IHSSCYATPSVSHKGVISSPEG | 22 | | 9 | 297 | 305 | LYKTVIPGQ | 9 | | 10 | 340 | 357 | IIGEPIDLTTFSNDPGLT | 18 | | 11 | 374 | 375 | GT | 2 | | 12 | 387 | 402 | TKKPVQHALLTPLGLD | 16 | | **BepiPred Linear Epitope Prediction 2.0**  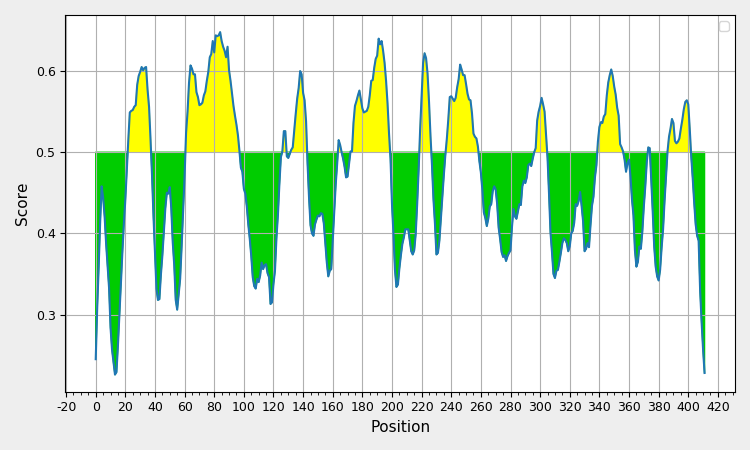 |
| --- | --- | --- | --- | --- | --- | --- | --- | --- | --- | --- | --- | --- | --- | --- | --- | --- | --- | --- | --- | --- | --- | --- | --- | --- | --- | --- | --- | --- | --- | --- | --- | --- | --- | --- | --- | --- | --- | --- | --- | --- | --- | --- | --- | --- | --- | --- | --- | --- | --- | --- | --- | --- | --- | --- | --- | --- | --- | --- | --- | --- | --- | --- | --- | --- | --- | --- |

| **Protein name: 3-carboxymuconate cyclase (Gp60–70)**  **Predicted peptides: 5110**   | **No.** | **Start** | **End** | **Peptide** | **Length** | | --- | --- | --- | --- | --- | | 1 | 23 | 38 | PTSDHYYADAESEACL | 16 | | 2 | 62 | 98 | NGSLLVNYATSTATGGRGGNGINPRGMPAGPDALFGQ | 37 | | 3 | 127 | 129 | DPT | 3 | | 4 | 133 | 143 | VVGEPAELPGE | 11 | | 5 | 165 | 167 | KAG | 3 | | 6 | 173 | 199 | YSWYGLGPFDELRPFDLHQTTPPHGPT | 27 | | 7 | 220 | 227 | GDPAVNNT | 8 | | 8 | 238 | 259 | IHSSCYATPSVSHKGVISSPEG | 22 | | 9 | 297 | 305 | LYKTVIPGQ | 9 | | 10 | 340 | 357 | IIGEPIDLTTFSNDPGLT | 18 | | 11 | 374 | 375 | GT | 2 | | 12 | 387 | 402 | TKKPVQHALLTPLGLD | 16 | | **BepiPred Linear Epitope Prediction 2.0**  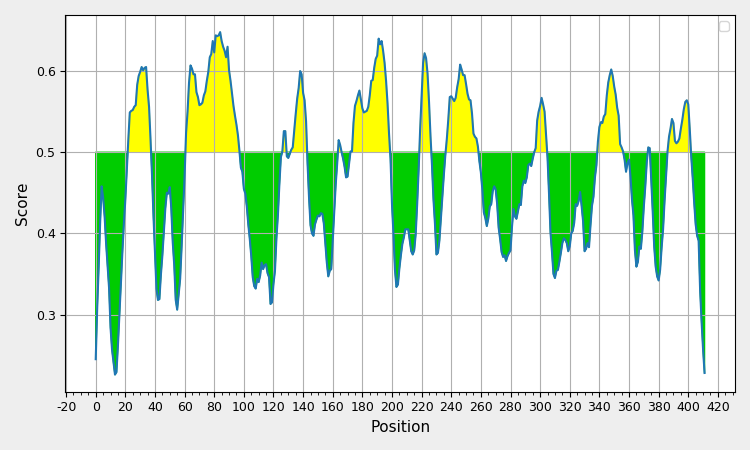 |
| --- | --- | --- | --- | --- | --- | --- | --- | --- | --- | --- | --- | --- | --- | --- | --- | --- | --- | --- | --- | --- | --- | --- | --- | --- | --- | --- | --- | --- | --- | --- | --- | --- | --- | --- | --- | --- | --- | --- | --- | --- | --- | --- | --- | --- | --- | --- | --- | --- | --- | --- | --- | --- | --- | --- | --- | --- | --- | --- | --- | --- | --- | --- | --- | --- | --- | --- |

| **Protein name: 3-carboxymuconate cyclase (Gp60–70)**  **Predicted peptides: Ss14**   | **No.** | **Start** | **End** | **Peptide** | **Length** | | --- | --- | --- | --- | --- | | 1 | 23 | 38 | PTSDHYYADAESEACL | 16 | | 2 | 62 | 98 | NGSLLVNYATSTATGGRGGNGINPRGMPAGPDALFGQ | 37 | | 3 | 127 | 129 | DPT | 3 | | 4 | 133 | 143 | VVGEPAELPGE | 11 | | 5 | 165 | 167 | KAG | 3 | | 6 | 173 | 199 | YSWYGLGPFDELRPFDLHQTTPPHGPT | 27 | | 7 | 220 | 227 | GDPAVNNT | 8 | | 8 | 238 | 259 | IHSSCYATPSVSHKGVISSPEG | 22 | | 9 | 297 | 305 | LYKTVIPGQ | 9 | | 10 | 340 | 357 | IIGEPIDLTTFSNDPGLT | 18 | | 11 | 374 | 375 | GT | 2 | | 12 | 387 | 402 | TKKPVQHALLTPLGLD | 16 | | **BepiPred Linear Epitope Prediction 2.0**  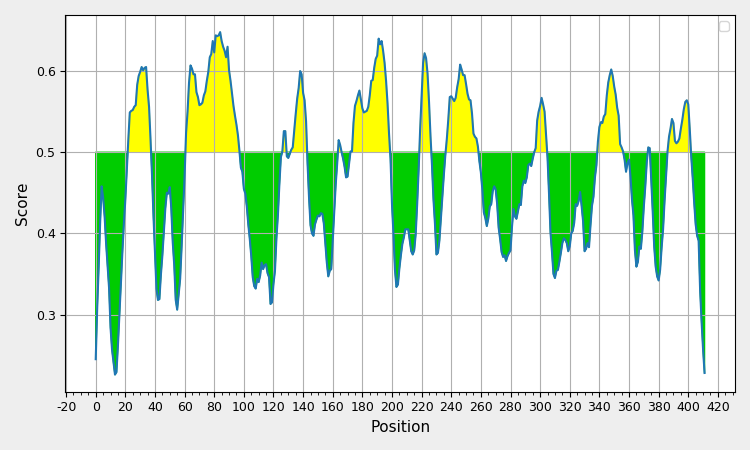 |
| --- | --- | --- | --- | --- | --- | --- | --- | --- | --- | --- | --- | --- | --- | --- | --- | --- | --- | --- | --- | --- | --- | --- | --- | --- | --- | --- | --- | --- | --- | --- | --- | --- | --- | --- | --- | --- | --- | --- | --- | --- | --- | --- | --- | --- | --- | --- | --- | --- | --- | --- | --- | --- | --- | --- | --- | --- | --- | --- | --- | --- | --- | --- | --- | --- | --- | --- |

| **Protein name: 3-carboxymuconate cyclase (Gp60–70)**  **Predicted peptides: Ss33**   | **No.** | **Start** | **End** | **Peptide** | **Length** | | --- | --- | --- | --- | --- | | 1 | 23 | 38 | PTSDHYYADAESEACL | 16 | | 2 | 62 | 98 | NGSLLVNYATSTATGGRGGNGINPRGMPAGPDALFGQ | 37 | | 3 | 127 | 129 | DPT | 3 | | 4 | 133 | 143 | VVGEPAELPGE | 11 | | 5 | 165 | 167 | KAG | 3 | | 6 | 173 | 199 | YSWYGLGPFDELRPFDLHQTTPPHGPT | 27 | | 7 | 220 | 227 | GDPAVNNT | 8 | | 8 | 238 | 259 | IHSSCYATPSVSHKGVISSPEG | 22 | | 9 | 297 | 305 | LYKTVIPGQ | 9 | | 10 | 340 | 357 | IIGEPIDLTTFSNDPGLT | 18 | | 11 | 374 | 375 | GT | 2 | | 12 | 387 | 402 | TKKPVQHALLTPLGLD | 16 | | **BepiPred Linear Epitope Prediction 2.0**  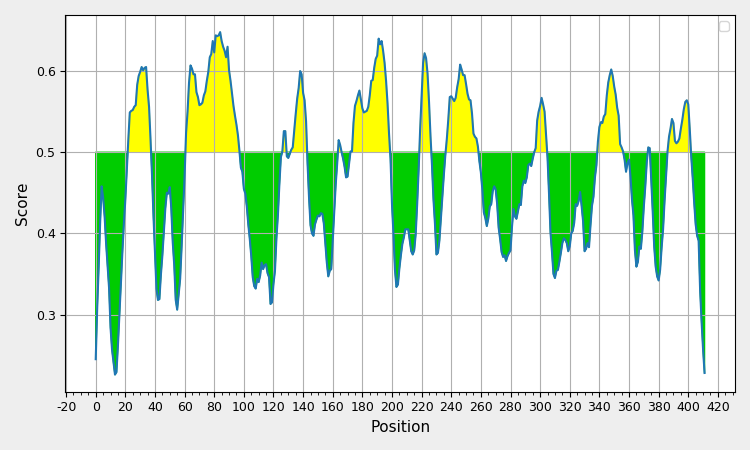 |
| --- | --- | --- | --- | --- | --- | --- | --- | --- | --- | --- | --- | --- | --- | --- | --- | --- | --- | --- | --- | --- | --- | --- | --- | --- | --- | --- | --- | --- | --- | --- | --- | --- | --- | --- | --- | --- | --- | --- | --- | --- | --- | --- | --- | --- | --- | --- | --- | --- | --- | --- | --- | --- | --- | --- | --- | --- | --- | --- | --- | --- | --- | --- | --- | --- | --- | --- |

| **Protein name: 3-carboxymuconate cyclase (Gp60–70)**  **Predicted peptides: Ss354**   | **No.** | **Start** | **End** | **Peptide** | **Length** | | --- | --- | --- | --- | --- | | 1 | 23 | 38 | PTSDHYYADAESEACL | 16 | | 2 | 62 | 98 | NGSLLVNYATSTATGGRGGNGINPRGMPAGPDALFGQ | 37 | | 3 | 127 | 129 | DPT | 3 | | 4 | 133 | 143 | VVGEPAELPGE | 11 | | 5 | 165 | 167 | KAG | 3 | | 6 | 173 | 199 | YSWYGLGPFDELRPFDLHQTTPPHGPT | 27 | | 7 | 220 | 227 | GDPAVNNT | 8 | | 8 | 238 | 259 | IHSSCYATPSVSHKGVISSPEG | 22 | | 9 | 297 | 305 | LYKTVIPGQ | 9 | | 10 | 340 | 357 | IIGEPIDLTTFSNDPGLT | 18 | | 11 | 374 | 375 | GT | 2 | | 12 | 387 | 402 | TKKPVQHALLTPLGLD | 16 | | **BepiPred Linear Epitope Prediction 2.0**  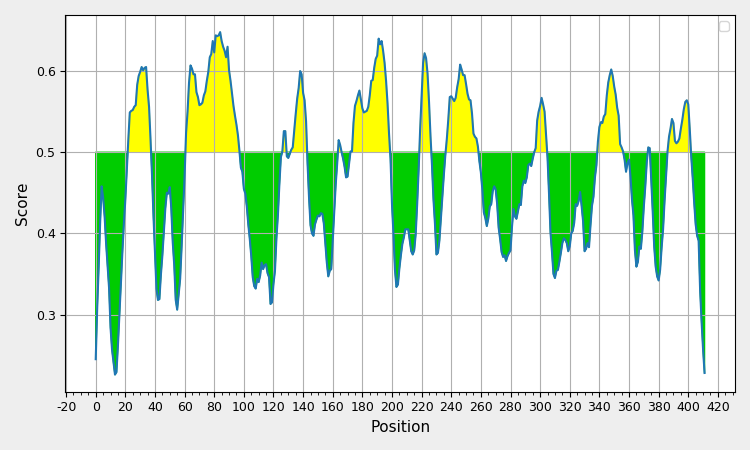 |
| --- | --- | --- | --- | --- | --- | --- | --- | --- | --- | --- | --- | --- | --- | --- | --- | --- | --- | --- | --- | --- | --- | --- | --- | --- | --- | --- | --- | --- | --- | --- | --- | --- | --- | --- | --- | --- | --- | --- | --- | --- | --- | --- | --- | --- | --- | --- | --- | --- | --- | --- | --- | --- | --- | --- | --- | --- | --- | --- | --- | --- | --- | --- | --- | --- | --- | --- |

| **Protein name: 3-carboxymuconate cyclase (Gp60–70)**  **Predicted peptides: Ss104**   | **No.** | **Start** | **End** | **Peptide** | **Length** | | --- | --- | --- | --- | --- | | 1 | 23 | 38 | PTSDHYYADAESEACL | 16 | | 2 | 62 | 98 | NGSLLVNYATSTATGGRGGNGINPRGMPAGPDALFGQ | 37 | | 3 | 127 | 129 | DPT | 3 | | 4 | 133 | 143 | VVGEPAELPGE | 11 | | 5 | 165 | 167 | KAG | 3 | | 6 | 173 | 173 | Y | 1 | | 7 | 175 | 199 | WYGLGPFDELRPFDLHQTTPPHGPT | 25 | | 8 | 220 | 227 | GDPAVNNT | 8 | | 9 | 238 | 259 | IHSSCYATPSVSHKGVISSPEG | 22 | | 10 | 297 | 305 | LYKTVIPGQ | 9 | | 11 | 340 | 357 | IIGEPIDLTTFSNDPGLT | 18 | | 12 | 374 | 375 | GT | 2 | | 13 | 387 | 402 | TKKPVQHALLTPLGLD | 16 | | **BepiPred Linear Epitope Prediction 2.0**  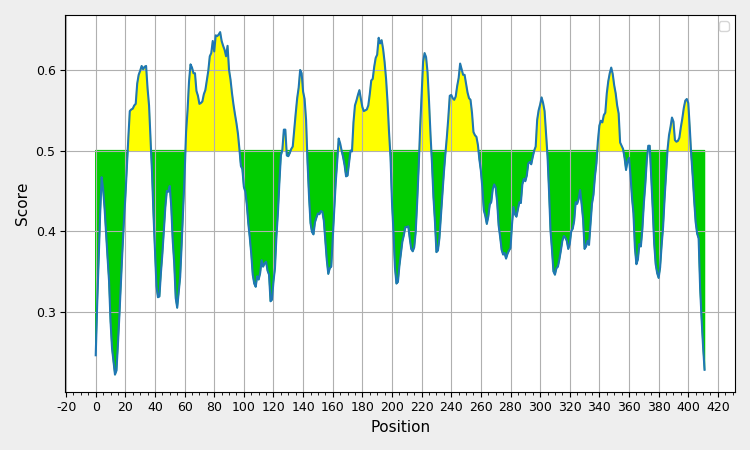 |
| --- | --- | --- | --- | --- | --- | --- | --- | --- | --- | --- | --- | --- | --- | --- | --- | --- | --- | --- | --- | --- | --- | --- | --- | --- | --- | --- | --- | --- | --- | --- | --- | --- | --- | --- | --- | --- | --- | --- | --- | --- | --- | --- | --- | --- | --- | --- | --- | --- | --- | --- | --- | --- | --- | --- | --- | --- | --- | --- | --- | --- | --- | --- | --- | --- | --- | --- | --- | --- | --- | --- | --- |

| **Protein name: 3-carboxymuconate cyclase (Gp60–70)**  **Predicted peptides: Ss245**   | **No.** | **Start** | **End** | **Peptide** | **Length** | | --- | --- | --- | --- | --- | | 1 | 23 | 38 | PTSDHYYADAESEACL | 16 | | 2 | 62 | 98 | NGSLLVNYATSTATGGRGGNGINPRGMPAGPDALFGQ | 37 | | 3 | 127 | 129 | DPT | 3 | | 4 | 133 | 143 | VVGEPAELPGE | 11 | | 5 | 165 | 167 | KAG | 3 | | 6 | 173 | 199 | YSWYGLGPFDELRPFDLHQTTPPHGPT | 27 | | 7 | 220 | 227 | GDPAVNNT | 8 | | 8 | 238 | 259 | IHSSCYATPSVSHKGVISSPEG | 22 | | 9 | 297 | 305 | LYKTVIPGQ | 9 | | 10 | 340 | 357 | IIGEPIDLTTFSNDPGLT | 18 | | 11 | 374 | 375 | GT | 2 | | 12 | 387 | 402 | TKKPVQHALLTPLGLD | 16 | | **BepiPred Linear Epitope Prediction 2.0**  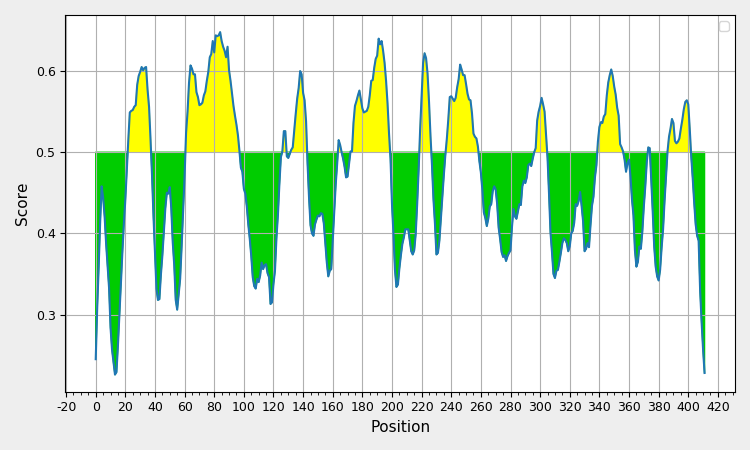 |
| --- | --- | --- | --- | --- | --- | --- | --- | --- | --- | --- | --- | --- | --- | --- | --- | --- | --- | --- | --- | --- | --- | --- | --- | --- | --- | --- | --- | --- | --- | --- | --- | --- | --- | --- | --- | --- | --- | --- | --- | --- | --- | --- | --- | --- | --- | --- | --- | --- | --- | --- | --- | --- | --- | --- | --- | --- | --- | --- | --- | --- | --- | --- | --- | --- | --- | --- |

| **Protein name: 3-carboxymuconate cyclase (Gp60–70)**  **Predicted peptides: Ss250**   | **No.** | **Start** | **End** | **Peptide** | **Length** | | --- | --- | --- | --- | --- | | 1 | 23 | 38 | PTSDHYYADAESEACL | 16 | | 2 | 62 | 98 | NGSLLVNYATSTATGGRGGNGINPRGMPAGPDALFGQ | 37 | | 3 | 127 | 129 | DPT | 3 | | 4 | 133 | 143 | VVGEPAELPGE | 11 | | 5 | 165 | 167 | KAG | 3 | | 6 | 173 | 199 | YSWYGLGPFDELRPFDLHQTTPPHGPT | 27 | | 7 | 220 | 227 | GDPAVNNT | 8 | | 8 | 238 | 259 | IHSSCYATPSVSHKGVISSPEG | 22 | | 9 | 297 | 305 | LYKTVIPGQ | 9 | | 10 | 340 | 357 | IIGEPIDLTTFSNDPGLT | 18 | | 11 | 374 | 375 | GT | 2 | | 12 | 387 | 402 | TKKPVQHALLTPLGLD | 16 | | **BepiPred Linear Epitope Prediction 2.0**  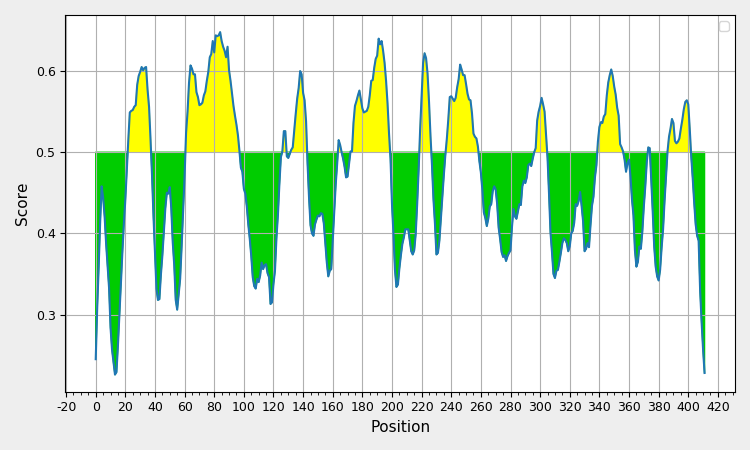 |
| --- | --- | --- | --- | --- | --- | --- | --- | --- | --- | --- | --- | --- | --- | --- | --- | --- | --- | --- | --- | --- | --- | --- | --- | --- | --- | --- | --- | --- | --- | --- | --- | --- | --- | --- | --- | --- | --- | --- | --- | --- | --- | --- | --- | --- | --- | --- | --- | --- | --- | --- | --- | --- | --- | --- | --- | --- | --- | --- | --- | --- | --- | --- | --- | --- | --- | --- |

| **Protein name: 3-carboxymuconate cyclase (Gp60–70)**  **Predicted peptides: Ss52**   | **No.** | **Start** | **End** | **Peptide** | **Length** | | --- | --- | --- | --- | --- | | 1 | 23 | 38 | PTSDHYYADAESEACL | 16 | | 2 | 62 | 98 | NGSLLVNYATSTATGGRGGNGINPRGMPAGPDALFGQ | 37 | | 3 | 127 | 129 | DPT | 3 | | 4 | 133 | 143 | VVGEPAELPGE | 11 | | 5 | 165 | 167 | KAG | 3 | | 6 | 173 | 199 | YSWYGLGPFDELRPFDLHQTTPPHGPT | 27 | | 7 | 220 | 227 | GDPAVNNT | 8 | | 8 | 238 | 259 | IHSSCYATPSVSHKGVISSPEG | 22 | | 9 | 297 | 305 | LYKTVIPGQ | 9 | | 10 | 340 | 357 | IIGEPIDLTTFSNDPGLT | 18 | | 11 | 374 | 375 | GT | 2 | | 12 | 387 | 402 | TKKPVQHALLTPLGLD | 16 | | **BepiPred Linear Epitope Prediction 2.0**  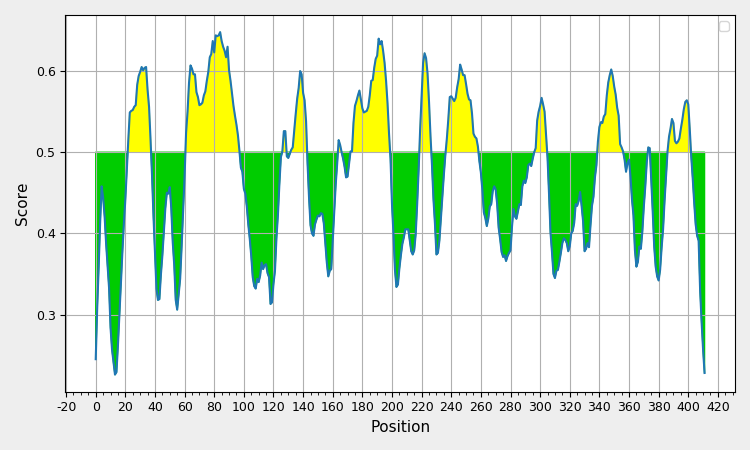 |
| --- | --- | --- | --- | --- | --- | --- | --- | --- | --- | --- | --- | --- | --- | --- | --- | --- | --- | --- | --- | --- | --- | --- | --- | --- | --- | --- | --- | --- | --- | --- | --- | --- | --- | --- | --- | --- | --- | --- | --- | --- | --- | --- | --- | --- | --- | --- | --- | --- | --- | --- | --- | --- | --- | --- | --- | --- | --- | --- | --- | --- | --- | --- | --- | --- | --- | --- |

| **Protein name: 3-carboxymuconate cyclase (Gp60–70)**  **Predicted peptides: Ss54**   | **No.** | **Start** | **End** | **Peptide** | **Length** | | --- | --- | --- | --- | --- | | 1 | 23 | 38 | PTSDHYYADAESEACL | 16 | | 2 | 62 | 98 | NGSLLVNYATSTATGGRGGNGINPRGMPAGPDALFGQ | 37 | | 3 | 127 | 129 | DPT | 3 | | 4 | 133 | 143 | VVGEPAELPGE | 11 | | 5 | 165 | 167 | KAG | 3 | | 6 | 173 | 199 | YSWYGLGPFDELRPFDLHQTTPPHGPT | 27 | | 7 | 220 | 227 | GDPAVNNT | 8 | | 8 | 238 | 259 | IHSSCYATPSVSHKGVISSPEG | 22 | | 9 | 297 | 305 | LYKTVIPGQ | 9 | | 10 | 340 | 357 | IIGEPIDLTTFSNDPGLT | 18 | | 11 | 374 | 375 | GT | 2 | | 12 | 387 | 402 | TKKPVQHALLTPLGLD | 16 | | **BepiPred Linear Epitope Prediction 2.0**  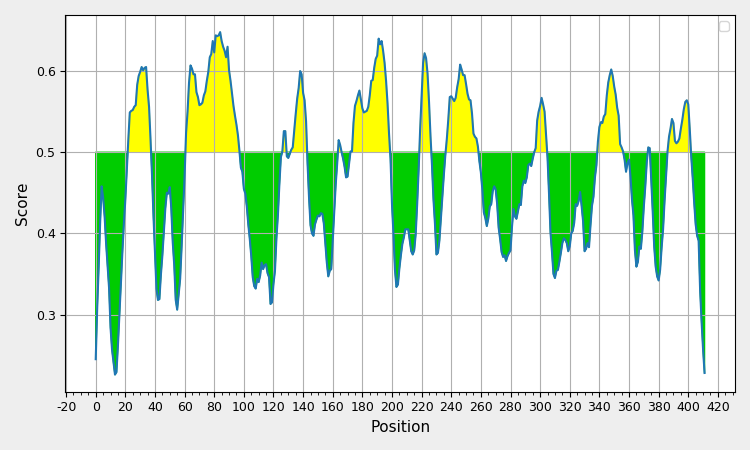 |
| --- | --- | --- | --- | --- | --- | --- | --- | --- | --- | --- | --- | --- | --- | --- | --- | --- | --- | --- | --- | --- | --- | --- | --- | --- | --- | --- | --- | --- | --- | --- | --- | --- | --- | --- | --- | --- | --- | --- | --- | --- | --- | --- | --- | --- | --- | --- | --- | --- | --- | --- | --- | --- | --- | --- | --- | --- | --- | --- | --- | --- | --- | --- | --- | --- | --- | --- |

| **Protein name: 3-carboxymuconate cyclase (Gp60–70)**  **Predicted peptides: Ss178**   | **No.** | **Start** | **End** | **Peptide** | **Length** | | --- | --- | --- | --- | --- | | 1 | 23 | 38 | PTSDHYYADAESEACL | 16 | | 2 | 62 | 97 | NGSLLVNYATSTATGGRGGNGINPRGMPAGPDALFG | 36 | | 3 | 128 | 143 | PTKVTVVGEPAELPGE | 16 | | 4 | 165 | 167 | KAG | 3 | | 5 | 174 | 199 | SWYGLGPFDELRPFDLHQTTPPHGPT | 26 | | 6 | 220 | 227 | GDPAVNNT | 8 | | 7 | 237 | 262 | HIHSSCYATPSVSHKGVISSPEGTAV | 26 | | 8 | 299 | 305 | KTVIPGQ | 7 | | 9 | 340 | 357 | IIGEPIDLTTFSNDPGLT | 18 | | 10 | 375 | 375 | T | 1 | | 11 | 387 | 402 | TKKPVQHALLTPLGLD | 16 | | **BepiPred Linear Epitope Prediction 2.0**  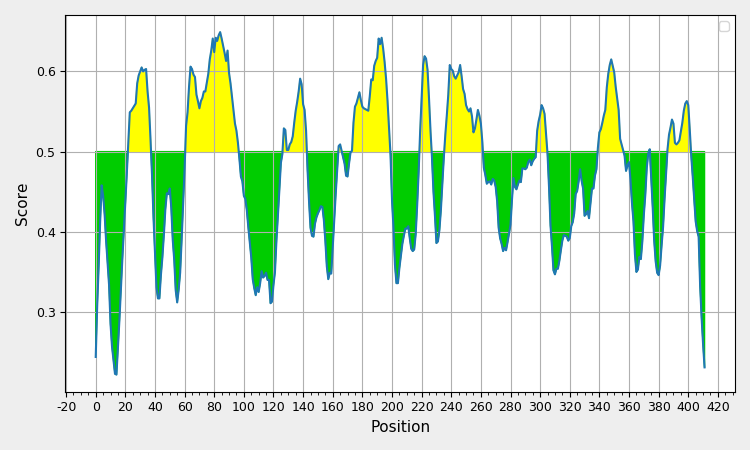 |
| --- | --- | --- | --- | --- | --- | --- | --- | --- | --- | --- | --- | --- | --- | --- | --- | --- | --- | --- | --- | --- | --- | --- | --- | --- | --- | --- | --- | --- | --- | --- | --- | --- | --- | --- | --- | --- | --- | --- | --- | --- | --- | --- | --- | --- | --- | --- | --- | --- | --- | --- | --- | --- | --- | --- | --- | --- | --- | --- | --- | --- | --- |

| **Protein name: 3-carboxymuconate cyclase (Gp60–70)**  **Predicted peptides: Ss177**   | **No.** | **Start** | **End** | **Peptide** | **Length** | | --- | --- | --- | --- | --- | | 1 | 23 | 38 | PTSDHYYADAESEACL | 16 | | 2 | 62 | 97 | NGSLLVNYATSTATGGRGGNGINPRGMPAGPDALFG | 36 | | 3 | 128 | 143 | PTKVTVVGEPAELPGE | 16 | | 4 | 165 | 167 | KAG | 3 | | 5 | 174 | 199 | SWYGLGPFDELRPFDLHQTTPPHGPT | 26 | | 6 | 220 | 227 | GDPAVNNT | 8 | | 7 | 237 | 262 | HIHSSCYATPSVSHKGVISSPEGTAV | 26 | | 8 | 299 | 305 | KTVIPGQ | 7 | | 9 | 340 | 357 | IIGEPIDLTTFSNDPGLT | 18 | | 10 | 375 | 375 | T | 1 | | 11 | 387 | 402 | TKKPVQHALLTPLGLD | 16 | | **BepiPred Linear Epitope Prediction 2.0**  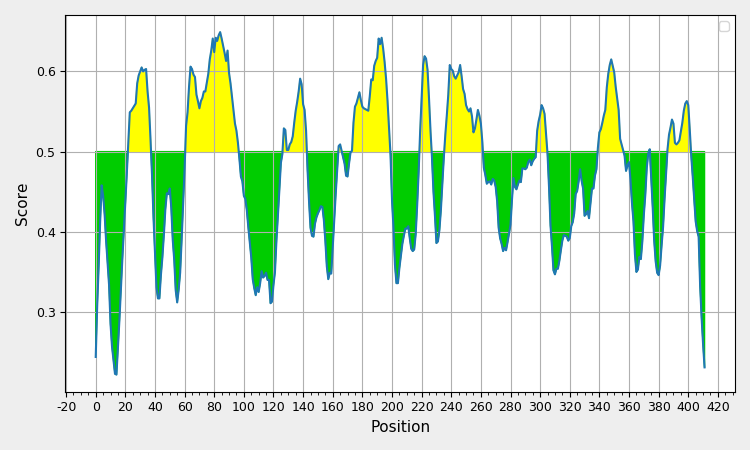 |
| --- | --- | --- | --- | --- | --- | --- | --- | --- | --- | --- | --- | --- | --- | --- | --- | --- | --- | --- | --- | --- | --- | --- | --- | --- | --- | --- | --- | --- | --- | --- | --- | --- | --- | --- | --- | --- | --- | --- | --- | --- | --- | --- | --- | --- | --- | --- | --- | --- | --- | --- | --- | --- | --- | --- | --- | --- | --- | --- | --- | --- | --- |

| **Protein name: 3-carboxymuconate cyclase (Gp60–70)**  **Predicted peptides: Ss252**   | **No.** | **Start** | **End** | **Peptide** | **Length** | | --- | --- | --- | --- | --- | | 1 | 23 | 38 | PTSDHYYADAESEACL | 16 | | 2 | 62 | 97 | NGSLLVNYATSTATGGRGGNGINPRGMPAGPDALFG | 36 | | 3 | 128 | 129 | PT | 2 | | 4 | 135 | 143 | GEPAELPGE | 9 | | 5 | 165 | 167 | KAG | 3 | | 6 | 173 | 200 | YSWYGLGPFDELRPFDLHQTTPPHGPTN | 28 | | 7 | 220 | 227 | GDPAVNNT | 8 | | 8 | 237 | 261 | HIHSSCYATPSVSHKGVISSPEGTG | 25 | | 9 | 299 | 305 | KTVIPGQ | 7 | | 10 | 340 | 354 | IIGEPIDLTTFSNDP | 15 | | 11 | 387 | 402 | TKKPVQHALLTPLGLD | 16 | | **BepiPred Linear Epitope Prediction 2.0**  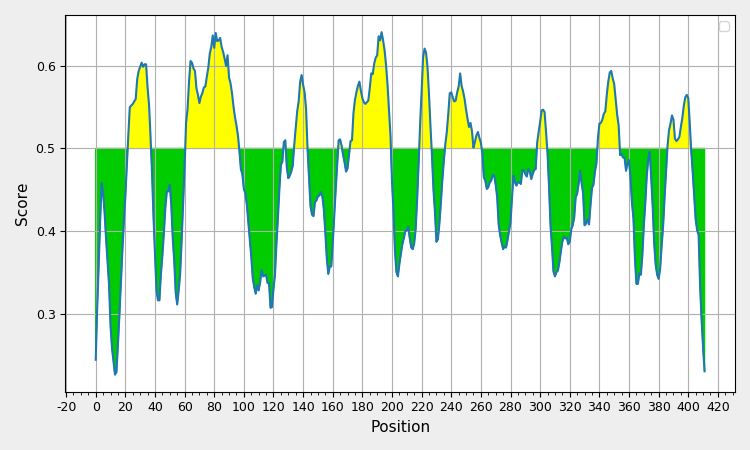 |
| --- | --- | --- | --- | --- | --- | --- | --- | --- | --- | --- | --- | --- | --- | --- | --- | --- | --- | --- | --- | --- | --- | --- | --- | --- | --- | --- | --- | --- | --- | --- | --- | --- | --- | --- | --- | --- | --- | --- | --- | --- | --- | --- | --- | --- | --- | --- | --- | --- | --- | --- | --- | --- | --- | --- | --- | --- | --- | --- | --- | --- | --- |

| **Protein name: 3-carboxymuconate cyclase (Gp60–70)**  **Predicted peptides: Ss38**   | **No.** | **Start** | **End** | **Peptide** | **Length** | | --- | --- | --- | --- | --- | | 1 | 23 | 38 | PTSDHYYADAESEACL | 16 | | 2 | 62 | 97 | NGSLLVNYATSTATGGRGGNGINPRGMPAGPDALFG | 36 | | 3 | 128 | 129 | PT | 2 | | 4 | 136 | 144 | EPAELPGEF | 9 | | 5 | 175 | 199 | WYGLGPFDELRPFDLHQTTPPHGPT | 25 | | 6 | 220 | 227 | GDPAVNNT | 8 | | 7 | 237 | 260 | HIHSSCYATPSVSHKGVISSPEGT | 24 | | 8 | 297 | 305 | LYKTVIPGQ | 9 | | 9 | 340 | 361 | IIGEPIDLTTFSNDPGLTEIRS | 22 | | 10 | 387 | 402 | TKKPVQHALLTPLGLD | 16 | | **BepiPred Linear Epitope Prediction 2.0**  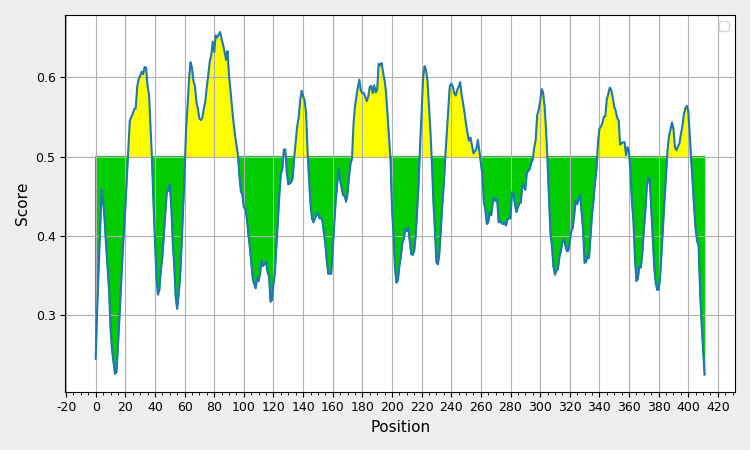 |
| --- | --- | --- | --- | --- | --- | --- | --- | --- | --- | --- | --- | --- | --- | --- | --- | --- | --- | --- | --- | --- | --- | --- | --- | --- | --- | --- | --- | --- | --- | --- | --- | --- | --- | --- | --- | --- | --- | --- | --- | --- | --- | --- | --- | --- | --- | --- | --- | --- | --- | --- | --- | --- | --- | --- | --- | --- |

| **Protein name: 3-carboxymuconate cyclase (Gp60–70)**  **Predicted peptides: Ss333**   | **No.** | **Start** | **End** | **Peptide** | **Length** | | --- | --- | --- | --- | --- | | 1 | 23 | 38 | PTSDHYYADAESEACL | 16 | | 2 | 62 | 98 | NGSLLVNYATSTATGGRGGNGINPRGMPAGPDALFGQ | 37 | | 3 | 127 | 129 | DPT | 3 | | 4 | 133 | 143 | VVGEPAELPGE | 11 | | 5 | 165 | 167 | KAG | 3 | | 6 | 173 | 199 | YSWYGLGPFDELRPFDLHQTTPPHGPT | 27 | | 7 | 220 | 227 | GDPAVNNT | 8 | | 8 | 238 | 259 | IHSSCYATPSVSHKGVISSPEG | 22 | | 9 | 297 | 305 | LYKTVIPGQ | 9 | | 10 | 340 | 357 | IIGEPIDLTTFSNDPGLT | 18 | | 11 | 374 | 375 | GT | 2 | | 12 | 387 | 402 | TKKPVQHALLTPLGLD | 16 | | **BepiPred Linear Epitope Prediction 2.0**  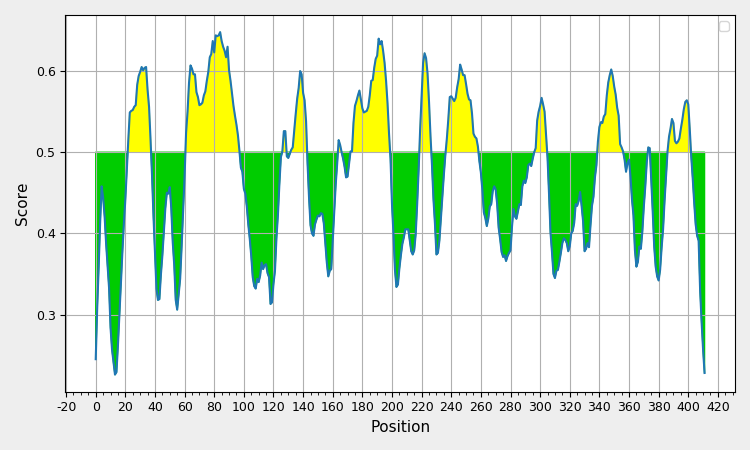 |
| --- | --- | --- | --- | --- | --- | --- | --- | --- | --- | --- | --- | --- | --- | --- | --- | --- | --- | --- | --- | --- | --- | --- | --- | --- | --- | --- | --- | --- | --- | --- | --- | --- | --- | --- | --- | --- | --- | --- | --- | --- | --- | --- | --- | --- | --- | --- | --- | --- | --- | --- | --- | --- | --- | --- | --- | --- | --- | --- | --- | --- | --- | --- | --- | --- | --- | --- |

| **Protein name: 3-carboxymuconate cyclase (Gp60–70)**  **Predicted peptides: Ss248**   | **No.** | **Start** | **End** | **Peptide** | **Length** | | --- | --- | --- | --- | --- | | 1 | 23 | 38 | PTSDHYYADAESEACL | 16 | | 2 | 62 | 100 | NGSLLVNYATSTATGGRGGNGINPRGMPAGPDALFGQGS | 39 | | 3 | 128 | 129 | PT | 2 | | 4 | 135 | 143 | GEPAELPGE | 9 | | 5 | 175 | 200 | WYGLGPFDELRPFDLHQTTPPHGPTN | 26 | | 6 | 220 | 227 | GDPAVNNT | 8 | | 7 | 237 | 255 | HIHSSCYATPSVSHKGVIS | 19 | | 8 | 257 | 261 | PEGTA | 5 | | 9 | 299 | 305 | KTVIPGQ | 7 | | 10 | 340 | 361 | IIGEPIDLTTFSNDPGLTEIRS | 22 | | 11 | 386 | 403 | LTKNPVQHGLLTPLGLDR | 18 | | **BepiPred Linear Epitope Prediction 2.0**  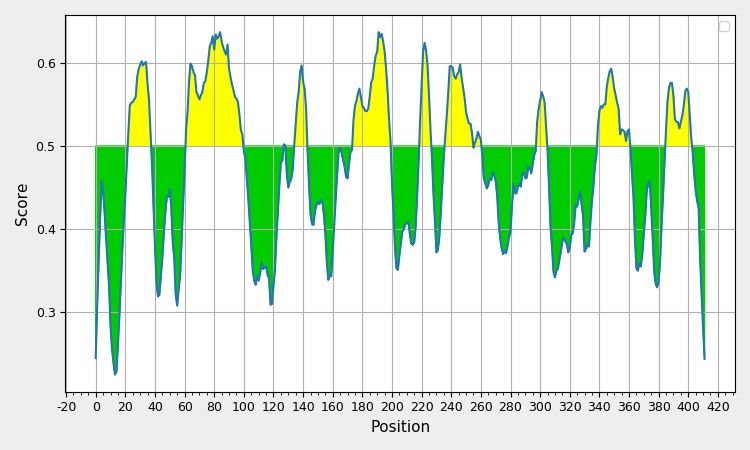 |
| --- | --- | --- | --- | --- | --- | --- | --- | --- | --- | --- | --- | --- | --- | --- | --- | --- | --- | --- | --- | --- | --- | --- | --- | --- | --- | --- | --- | --- | --- | --- | --- | --- | --- | --- | --- | --- | --- | --- | --- | --- | --- | --- | --- | --- | --- | --- | --- | --- | --- | --- | --- | --- | --- | --- | --- | --- | --- | --- | --- | --- | --- |

| **Protein name: 3-carboxymuconate cyclase (Gp60–70)**  **Predicted peptides: Ss246**   | **No.** | **Start** | **End** | **Peptide** | **Length** | | --- | --- | --- | --- | --- | | 1 | 23 | 38 | PTSDHYYADAESEACL | 16 | | 2 | 62 | 98 | NGSLLVNYATSTATGGRGGNGINPRGMPAGPDALFGQ | 37 | | 3 | 128 | 129 | PT | 2 | | 4 | 136 | 143 | EPAELPGE | 8 | | 5 | 175 | 199 | WYGLGPFDELRPFDLHQTTPPHGPT | 25 | | 6 | 220 | 227 | GDPAVNNT | 8 | | 7 | 238 | 255 | IHSSCYATPSVSHKGVIS | 18 | | 8 | 259 | 259 | G | 1 | | 9 | 299 | 306 | KTVIPGQD | 8 | | 10 | 340 | 354 | IIGEPIDLTTFSNDP | 15 | | 11 | 387 | 403 | TKNPAQHALLTPLGLDR | 17 | | **BepiPred Linear Epitope Prediction 2.0**  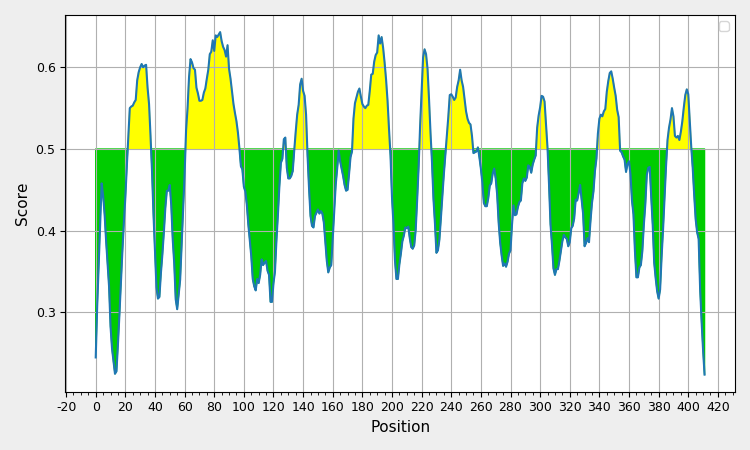 |
| --- | --- | --- | --- | --- | --- | --- | --- | --- | --- | --- | --- | --- | --- | --- | --- | --- | --- | --- | --- | --- | --- | --- | --- | --- | --- | --- | --- | --- | --- | --- | --- | --- | --- | --- | --- | --- | --- | --- | --- | --- | --- | --- | --- | --- | --- | --- | --- | --- | --- | --- | --- | --- | --- | --- | --- | --- | --- | --- | --- | --- | --- |

| **Protein name: 3-carboxymuconate cyclase (Gp60–70)**  **Predicted peptides: Ss254**   | **No.** | **Start** | **End** | **Peptide** | **Length** | | --- | --- | --- | --- | --- | | 1 | 23 | 38 | PTSDHYYADAESEACL | 16 | | 2 | 62 | 96 | NGSLLVNYATSTATGGRGGNGINPRGMPAGPDALF | 35 | | 3 | 128 | 129 | PT | 2 | | 4 | 135 | 143 | GEPAELPGE | 9 | | 5 | 165 | 165 | K | 1 | | 6 | 175 | 200 | WYGLGPFDELRPFDLHQTTPPHGPTN | 26 | | 7 | 220 | 227 | GDPAVNNT | 8 | | 8 | 237 | 262 | HIHSSCYATPSVSHKGVISSPEGTAV | 26 | | 9 | 297 | 305 | LYKTVIPGQ | 9 | | 10 | 340 | 354 | IIGEPIDLTTFSNDP | 15 | | 11 | 387 | 402 | TKNPVQHALLTPLGLD | 16 | | **BepiPred Linear Epitope Prediction 2.0**  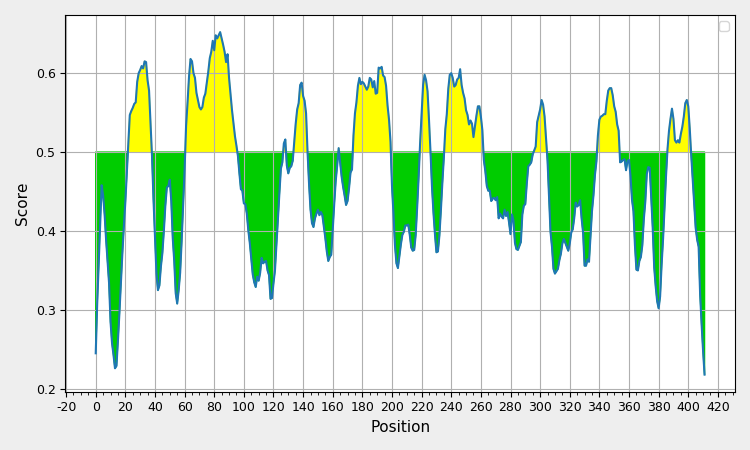 |
| --- | --- | --- | --- | --- | --- | --- | --- | --- | --- | --- | --- | --- | --- | --- | --- | --- | --- | --- | --- | --- | --- | --- | --- | --- | --- | --- | --- | --- | --- | --- | --- | --- | --- | --- | --- | --- | --- | --- | --- | --- | --- | --- | --- | --- | --- | --- | --- | --- | --- | --- | --- | --- | --- | --- | --- | --- | --- | --- | --- | --- | --- |

| **Protein name: 3-carboxymuconate cyclase (Gp60–70)**  **Predicted peptides: Ss44**   | **No.** | **Start** | **End** | **Peptide** | **Length** | | --- | --- | --- | --- | --- | | 1 | 23 | 38 | PTSDHYYADAESEACL | 16 | | 2 | 62 | 96 | NGSLLVNYATSTATGGRGGNGINPRGMPAGPDALF | 35 | | 3 | 128 | 129 | PT | 2 | | 4 | 135 | 143 | GEPAELPGE | 9 | | 5 | 165 | 165 | K | 1 | | 6 | 175 | 200 | WYGLGPFDELRPFDLHQTTPPHGPTN | 26 | | 7 | 220 | 227 | GDPAVNNT | 8 | | 8 | 237 | 262 | HIHSSCYATPSVSHKGVISSPEGTAV | 26 | | 9 | 297 | 305 | LYKTVIPGQ | 9 | | 10 | 340 | 354 | IIGEPIDLTTFSNDP | 15 | | 11 | 387 | 402 | TKNPVQHALLTPLGLD | 16 | | **BepiPred Linear Epitope Prediction 2.0**  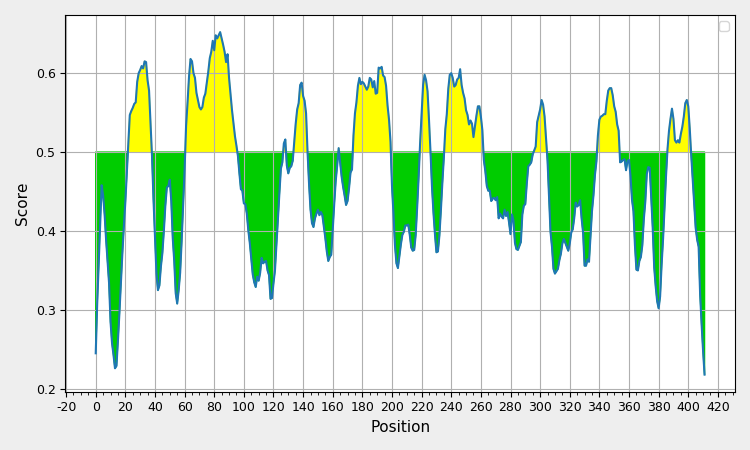 |
| --- | --- | --- | --- | --- | --- | --- | --- | --- | --- | --- | --- | --- | --- | --- | --- | --- | --- | --- | --- | --- | --- | --- | --- | --- | --- | --- | --- | --- | --- | --- | --- | --- | --- | --- | --- | --- | --- | --- | --- | --- | --- | --- | --- | --- | --- | --- | --- | --- | --- | --- | --- | --- | --- | --- | --- | --- | --- | --- | --- | --- | --- |

| **Protein name: 3-carboxymuconate cyclase (Gp60–70)**  **Predicted peptides: Ss57**   | **No.** | **Start** | **End** | **Peptide** | **Length** | | --- | --- | --- | --- | --- | | 1 | 23 | 38 | PTSDHYYADAESEACL | 16 | | 2 | 62 | 98 | NGSLLVNYATSTATGGRGGNGINPRGMPAGPDALFGQ | 37 | | 3 | 127 | 129 | DPT | 3 | | 4 | 133 | 143 | VVGEPAELPGE | 11 | | 5 | 165 | 167 | KAG | 3 | | 6 | 173 | 199 | YSWYGLGPFDELRPFDLHQTTPPHGPT | 27 | | 7 | 220 | 227 | GDPAVNNT | 8 | | 8 | 238 | 259 | IHSSCYATPSVSHKGVISSPEG | 22 | | 9 | 297 | 305 | LYKTVIPGQ | 9 | | 10 | 340 | 357 | IIGEPIDLTTFSNDPGLT | 18 | | 11 | 374 | 375 | GT | 2 | | 12 | 389 | 402 | NLVQHALLTPLGLD | 14 | | **BepiPred Linear Epitope Prediction 2.0**  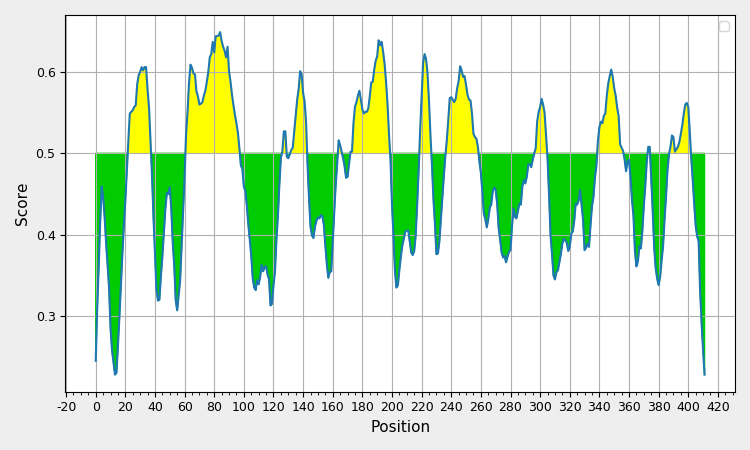 |
| --- | --- | --- | --- | --- | --- | --- | --- | --- | --- | --- | --- | --- | --- | --- | --- | --- | --- | --- | --- | --- | --- | --- | --- | --- | --- | --- | --- | --- | --- | --- | --- | --- | --- | --- | --- | --- | --- | --- | --- | --- | --- | --- | --- | --- | --- | --- | --- | --- | --- | --- | --- | --- | --- | --- | --- | --- | --- | --- | --- | --- | --- | --- | --- | --- | --- | --- |

| **Protein name: 3-carboxymuconate cyclase (Gp60–70)**  **Predicted peptides: Ss360**   | **No.** | **Start** | **End** | **Peptide** | **Length** | | --- | --- | --- | --- | --- | | 1 | 23 | 38 | PTSDHYYADAESEACL | 16 | | 2 | 62 | 98 | NGSLLVNYATSTATGGRGGNGINPRGMPAGPDALFGQ | 37 | | 3 | 127 | 129 | DPT | 3 | | 4 | 133 | 143 | VVGEPAELPGE | 11 | | 5 | 165 | 167 | KAG | 3 | | 6 | 173 | 199 | YSWYGLGPFDELRPFDLHQTTPPHGPT | 27 | | 7 | 220 | 227 | GDPAVNNT | 8 | | 8 | 238 | 259 | IHSSCYATPSVSHKGVISSPEG | 22 | | 9 | 297 | 305 | LYKTVIPGQ | 9 | | 10 | 340 | 357 | IIGEPIDLTTFSNDPGLT | 18 | | 11 | 374 | 375 | GT | 2 | | 12 | 387 | 402 | TKKPVQHALLTPLGLD | 16 | | **BepiPred Linear Epitope Prediction 2.0**  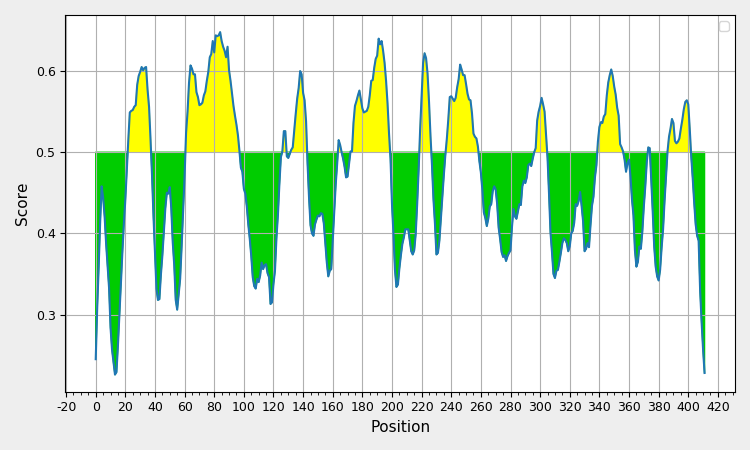 |
| --- | --- | --- | --- | --- | --- | --- | --- | --- | --- | --- | --- | --- | --- | --- | --- | --- | --- | --- | --- | --- | --- | --- | --- | --- | --- | --- | --- | --- | --- | --- | --- | --- | --- | --- | --- | --- | --- | --- | --- | --- | --- | --- | --- | --- | --- | --- | --- | --- | --- | --- | --- | --- | --- | --- | --- | --- | --- | --- | --- | --- | --- | --- | --- | --- | --- | --- |

| **Protein name: 3-carboxymuconate cyclase (Gp60–70)**  **Predicted peptides: Ss338**   | **No.** | **Start** | **End** | **Peptide** | **Length** | | --- | --- | --- | --- | --- | | 1 | 23 | 38 | PTSDHYYADAESEACL | 16 | | 2 | 62 | 98 | NGSLLVNYATSTATGGRGGNGINPRGMPAGPDALFGQ | 37 | | 3 | 128 | 129 | PT | 2 | | 4 | 135 | 143 | GEPAELPGE | 9 | | 5 | 165 | 165 | K | 1 | | 6 | 175 | 199 | WYGLGPFDELRPFDLHQTTPPHGPT | 25 | | 7 | 220 | 227 | GDPAVNNT | 8 | | 8 | 238 | 255 | IHSSCYATPSVSHKGVIS | 18 | | 9 | 259 | 259 | G | 1 | | 10 | 299 | 306 | KTVIPGQD | 8 | | 11 | 340 | 354 | IIGEPIDLTTFSNDP | 15 | | 12 | 387 | 392 | TKKPVQ | 6 | | 13 | 394 | 401 | ALLTPLGL | 8 | | **BepiPred Linear Epitope Prediction 2.0**  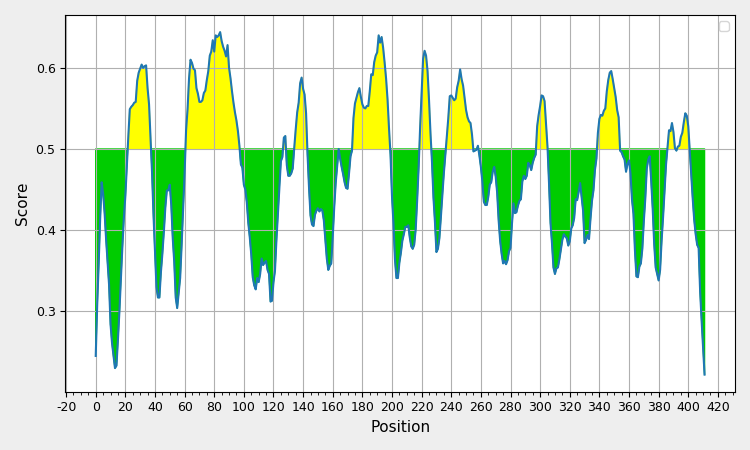 |
| --- | --- | --- | --- | --- | --- | --- | --- | --- | --- | --- | --- | --- | --- | --- | --- | --- | --- | --- | --- | --- | --- | --- | --- | --- | --- | --- | --- | --- | --- | --- | --- | --- | --- | --- | --- | --- | --- | --- | --- | --- | --- | --- | --- | --- | --- | --- | --- | --- | --- | --- | --- | --- | --- | --- | --- | --- | --- | --- | --- | --- | --- | --- | --- | --- | --- | --- | --- | --- | --- | --- | --- |

| **Protein name: 3-carboxymuconate cyclase (Gp60–70)**  **Predicted peptides: Ss10**   | **No.** | **Start** | **End** | **Peptide** | **Length** | | --- | --- | --- | --- | --- | | 1 | 23 | 38 | PTSDHYYADAESEACL | 16 | | 2 | 62 | 97 | NGSLLVNYATSTATGGRGGNGINPRGMPAGPDALFG | 36 | | 3 | 128 | 129 | PT | 2 | | 4 | 134 | 143 | VGEPPELPGE | 10 | | 5 | 165 | 165 | K | 1 | | 6 | 175 | 199 | WYGLGPFDELRPFDLHQTTPPHGPT | 25 | | 7 | 220 | 226 | GDPAVNN | 7 | | 8 | 237 | 259 | HIHSSCYATPSVSHKAVISSPEG | 23 | | 9 | 297 | 306 | LYKTVIPGQD | 10 | | 10 | 340 | 354 | IIGEPIDLTTFSNDP | 15 | | 11 | 386 | 402 | LTKKPLQHALLTPLGLD | 17 | | **BepiPred Linear Epitope Prediction 2.0**  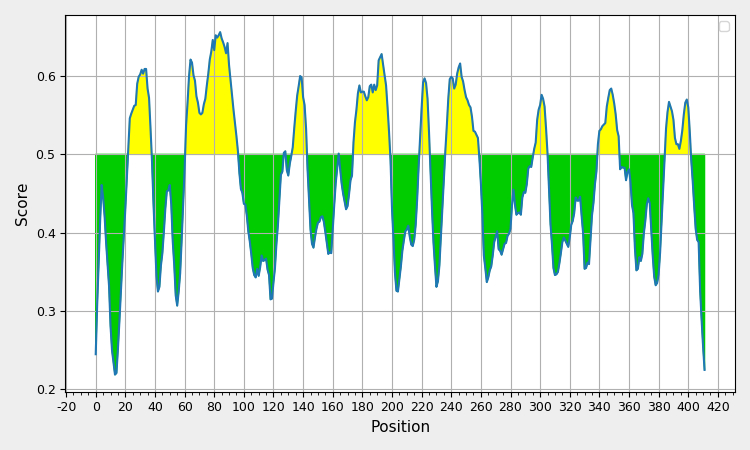 |
| --- | --- | --- | --- | --- | --- | --- | --- | --- | --- | --- | --- | --- | --- | --- | --- | --- | --- | --- | --- | --- | --- | --- | --- | --- | --- | --- | --- | --- | --- | --- | --- | --- | --- | --- | --- | --- | --- | --- | --- | --- | --- | --- | --- | --- | --- | --- | --- | --- | --- | --- | --- | --- | --- | --- | --- | --- | --- | --- | --- | --- | --- |

| **Protein name: 3-carboxymuconate cyclase (Gp60–70)**  **Predicted peptides: Ss332**   | **No.** | **Start** | **End** | **Peptide** | **Length** | | --- | --- | --- | --- | --- | | 1 | 23 | 38 | PTSDHYYADAESEACL | 16 | | 2 | 62 | 97 | NGSLLVNYATSTATGGRGGNGINPRGMPAGPDALFG | 36 | | 3 | 128 | 129 | PT | 2 | | 4 | 135 | 143 | GEPAELPGE | 9 | | 5 | 165 | 165 | K | 1 | | 6 | 173 | 200 | YSWYGLGPFDELRPFDLHQTTPPHGPTN | 28 | | 7 | 220 | 227 | GDPAVNNT | 8 | | 8 | 238 | 261 | IHSSCYATPSVSHKGVISSPEGTA | 24 | | 9 | 299 | 305 | KTVIPGQ | 7 | | 10 | 340 | 354 | IIGEPIDLTTFSNDP | 15 | | 11 | 386 | 402 | LTKNPQQHALLTPLGLD | 17 | | **BepiPred Linear Epitope Prediction 2.0**  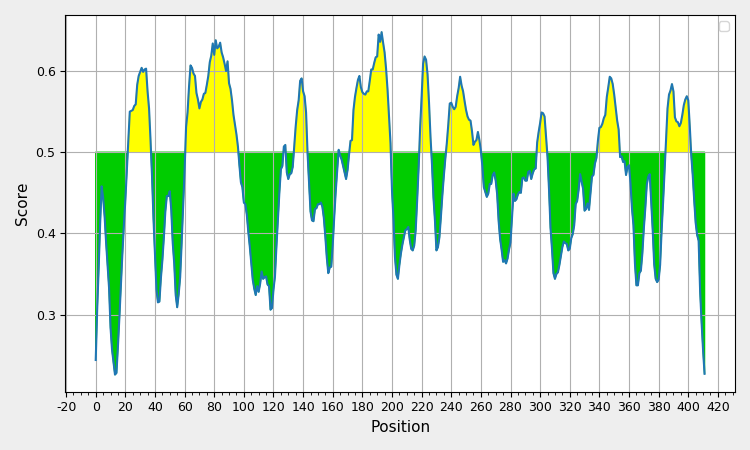 |
| --- | --- | --- | --- | --- | --- | --- | --- | --- | --- | --- | --- | --- | --- | --- | --- | --- | --- | --- | --- | --- | --- | --- | --- | --- | --- | --- | --- | --- | --- | --- | --- | --- | --- | --- | --- | --- | --- | --- | --- | --- | --- | --- | --- | --- | --- | --- | --- | --- | --- | --- | --- | --- | --- | --- | --- | --- | --- | --- | --- | --- | --- |

| **Protein name: 3-carboxymuconate cyclase (Gp60–70)**  **Predicted peptides: Ss265**   | **No.** | **Start** | **End** | **Peptide** | **Length** | | --- | --- | --- | --- | --- | | 1 | 23 | 38 | PTSDHYYADAESEACL | 16 | | 2 | 62 | 100 | NGSLLVNYATSTATGGRGGNGINPRGMPAGPDALFGQGS | 39 | | 3 | 128 | 129 | PT | 2 | | 4 | 133 | 143 | VVGEPAELPGE | 11 | | 5 | 165 | 167 | KAG | 3 | | 6 | 175 | 199 | WYGLGPFDELRPFDLHQTTPPHGPT | 25 | | 7 | 220 | 227 | GDPAVNNT | 8 | | 8 | 237 | 261 | HIHSSCYATPSVSHKGVISSPEGTA | 25 | | 9 | 298 | 305 | YKTVIPGQ | 8 | | 10 | 340 | 357 | IIGEPIDLTTFSNDPGLT | 18 | | 11 | 374 | 375 | GT | 2 | | 12 | 388 | 402 | KKPVQHALLTPLGLD | 15 | | **BepiPred Linear Epitope Prediction 2.0**  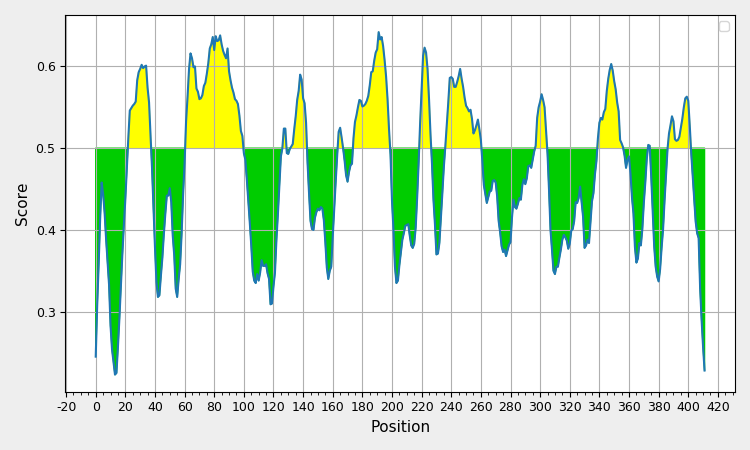 |
| --- | --- | --- | --- | --- | --- | --- | --- | --- | --- | --- | --- | --- | --- | --- | --- | --- | --- | --- | --- | --- | --- | --- | --- | --- | --- | --- | --- | --- | --- | --- | --- | --- | --- | --- | --- | --- | --- | --- | --- | --- | --- | --- | --- | --- | --- | --- | --- | --- | --- | --- | --- | --- | --- | --- | --- | --- | --- | --- | --- | --- | --- | --- | --- | --- | --- | --- |

| **Protein name: 3-carboxymuconate cyclase (Gp60–70)**  **Predicted peptides: Ss226**   | **No.** | **Start** | **End** | **Peptide** | **Length** | | --- | --- | --- | --- | --- | | 1 | 23 | 38 | PTSDHYYADAESEACL | 16 | | 2 | 62 | 97 | NGSLLVNYATSTATGGRGGNGINPRGMPAGPDALFG | 36 | | 3 | 128 | 129 | PT | 2 | | 4 | 132 | 143 | TVVGEPAELPGE | 12 | | 5 | 165 | 166 | KA | 2 | | 6 | 175 | 200 | WYGLGPFDELRPFDLHQTTPPHGPTN | 26 | | 7 | 220 | 227 | GDPAVNNT | 8 | | 8 | 237 | 261 | HIHSSCYATPSVSHKGVISSPEGTA | 25 | | 9 | 299 | 305 | KTVIPGQ | 7 | | 10 | 340 | 354 | IIGEPIDLTTFSNDP | 15 | | 11 | 374 | 375 | GT | 2 | | 12 | 387 | 402 | TKKPVQHALLTPLGLD | 16 | | **BepiPred Linear Epitope Prediction 2.0**  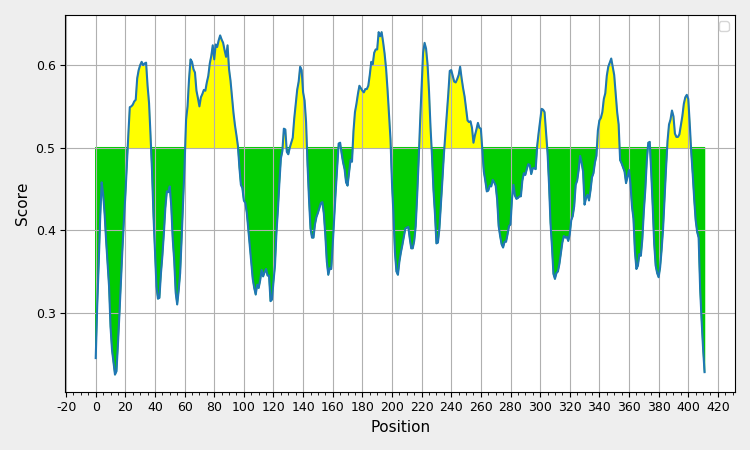 |
| --- | --- | --- | --- | --- | --- | --- | --- | --- | --- | --- | --- | --- | --- | --- | --- | --- | --- | --- | --- | --- | --- | --- | --- | --- | --- | --- | --- | --- | --- | --- | --- | --- | --- | --- | --- | --- | --- | --- | --- | --- | --- | --- | --- | --- | --- | --- | --- | --- | --- | --- | --- | --- | --- | --- | --- | --- | --- | --- | --- | --- | --- | --- | --- | --- | --- | --- |

| **Protein name: 3-carboxymuconate cyclase (Gp60–70)**  **Predicted peptides: Ss172**   | **No.** | **Start** | **End** | **Peptide** | **Length** | | --- | --- | --- | --- | --- | | 1 | 23 | 38 | PTSDHYYADAESEACL | 16 | | 2 | 62 | 98 | NGSLLVNHATSTATGGRGGNGINPRGMPAGPDALFGQ | 37 | | 3 | 128 | 129 | PT | 2 | | 4 | 135 | 143 | GEPAELPGE | 9 | | 5 | 175 | 199 | WYGLGPFDELRPFDLHQTTPPHGPT | 25 | | 6 | 220 | 227 | GDPAVNNT | 8 | | 7 | 237 | 259 | HIHSSCYATPSVSHKGVISSPEG | 23 | | 8 | 299 | 306 | KTVIPGQD | 8 | | 9 | 340 | 355 | IIGEPIDLTTFSNDPG | 16 | | 10 | 388 | 402 | KNPVQHALLTPLGLD | 15 | | **BepiPred Linear Epitope Prediction 2.0**  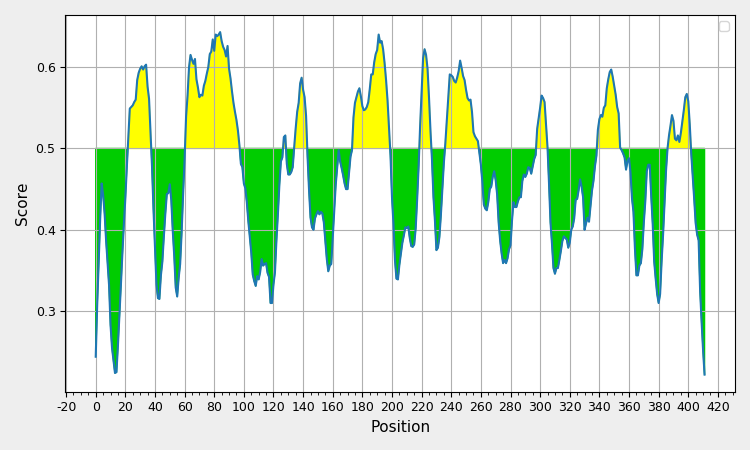 |
| --- | --- | --- | --- | --- | --- | --- | --- | --- | --- | --- | --- | --- | --- | --- | --- | --- | --- | --- | --- | --- | --- | --- | --- | --- | --- | --- | --- | --- | --- | --- | --- | --- | --- | --- | --- | --- | --- | --- | --- | --- | --- | --- | --- | --- | --- | --- | --- | --- | --- | --- | --- | --- | --- | --- | --- | --- |

| **Protein name: 3-carboxymuconate cyclase (Gp60–70)**  **Predicted peptides: Ss174**   | **No.** | **Start** | **End** | **Peptide** | **Length** | | --- | --- | --- | --- | --- | | 1 | 23 | 38 | PTSDHYYADAESEACL | 16 | | 2 | 62 | 100 | NGSLLVNHATSTATGGRGGNGINPRGMPAGPDALFGQGS | 39 | | 3 | 128 | 129 | PT | 2 | | 4 | 133 | 143 | VVGEPAELPGE | 11 | | 5 | 165 | 167 | KAG | 3 | | 6 | 175 | 199 | WYGLGPFDELRPFDLHQTTPPHGPT | 25 | | 7 | 220 | 227 | GDPAVNNT | 8 | | 8 | 237 | 261 | HIHSSCYATPSVSHKGVISSPEGTA | 25 | | 9 | 299 | 305 | KTVIPGQ | 7 | | 10 | 340 | 357 | IIGEPIDLTTFSNDPGLT | 18 | | 11 | 374 | 375 | GT | 2 | | 12 | 388 | 402 | KKPVQHALLTPLGLD | 15 | | **BepiPred Linear Epitope Prediction 2.0**  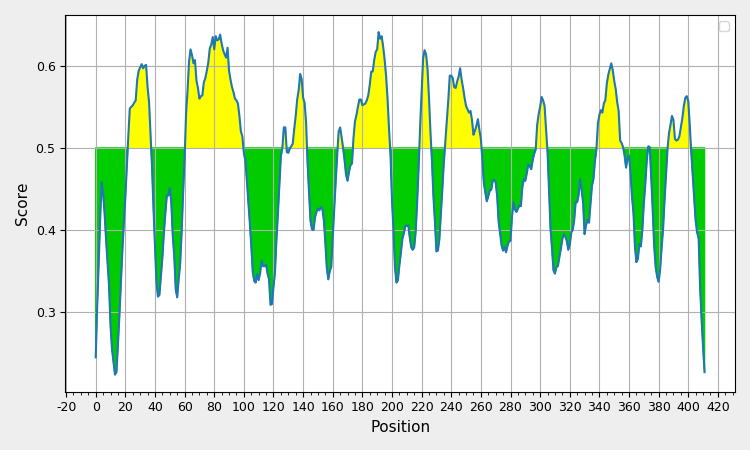 |
| --- | --- | --- | --- | --- | --- | --- | --- | --- | --- | --- | --- | --- | --- | --- | --- | --- | --- | --- | --- | --- | --- | --- | --- | --- | --- | --- | --- | --- | --- | --- | --- | --- | --- | --- | --- | --- | --- | --- | --- | --- | --- | --- | --- | --- | --- | --- | --- | --- | --- | --- | --- | --- | --- | --- | --- | --- | --- | --- | --- | --- | --- | --- | --- | --- | --- | --- |

| **Protein name: 3-carboxymuconate cyclase (Gp60–70)**  **Predicted peptides: Ss171**   | **No.** | **Start** | **End** | **Peptide** | **Length** | | --- | --- | --- | --- | --- | | 1 | 23 | 38 | PTSDHYYADAESEACL | 16 | | 2 | 62 | 100 | NGSLLVNHATSTATGGRGGNGINPRGMPAGPDALFGQGS | 39 | | 3 | 128 | 129 | PT | 2 | | 4 | 133 | 143 | VVGEPAELPGE | 11 | | 5 | 165 | 167 | KAG | 3 | | 6 | 175 | 199 | WYGLGPFDELRPFDLHQTTPPHGPT | 25 | | 7 | 220 | 227 | GDPAVNNT | 8 | | 8 | 237 | 261 | HIHSSCYATPSVSHKGVISSPEGTA | 25 | | 9 | 299 | 305 | KTVIPGQ | 7 | | 10 | 340 | 357 | IIGEPIDLTTFSNDPGLT | 18 | | 11 | 374 | 375 | GT | 2 | | 12 | 388 | 402 | KKPVQHALLTPLGLD | 15 | | **BepiPred Linear Epitope Prediction 2.0**  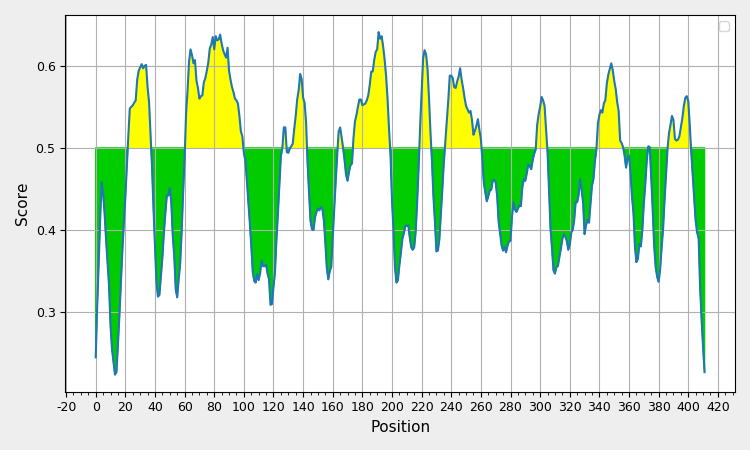 |
| --- | --- | --- | --- | --- | --- | --- | --- | --- | --- | --- | --- | --- | --- | --- | --- | --- | --- | --- | --- | --- | --- | --- | --- | --- | --- | --- | --- | --- | --- | --- | --- | --- | --- | --- | --- | --- | --- | --- | --- | --- | --- | --- | --- | --- | --- | --- | --- | --- | --- | --- | --- | --- | --- | --- | --- | --- | --- | --- | --- | --- | --- | --- | --- | --- | --- | --- |

| **Protein name: 3-carboxymuconate cyclase (Gp60–70)**  **Predicted peptides: Ss34**   | **No.** | **Start** | **End** | **Peptide** | **Length** | | --- | --- | --- | --- | --- | | 1 | 25 | 38 | SDHYYADAESEACL | 14 | | 2 | 62 | 97 | NGSLLVNHATSTATGGRGGNGINPRGMPAGPDALFG | 36 | | 3 | 128 | 129 | PT | 2 | | 4 | 133 | 143 | VVGEPAELPGE | 11 | | 5 | 173 | 200 | YSWYGLGPFDELRPFDLHQTTPPHGPTN | 28 | | 6 | 220 | 225 | GDPAVN | 6 | | 7 | 237 | 258 | HIHSSCYATPSVSHKGVISSPD | 22 | | 8 | 296 | 305 | TLYKTVIPGQ | 10 | | 9 | 340 | 356 | IIGEPFDLTTFSNDPGL | 17 | | 10 | 374 | 374 | G | 1 | | 11 | 387 | 404 | TKKPVQHALLTPLGLDRN | 18 | | **BepiPred Linear Epitope Prediction 2.0**  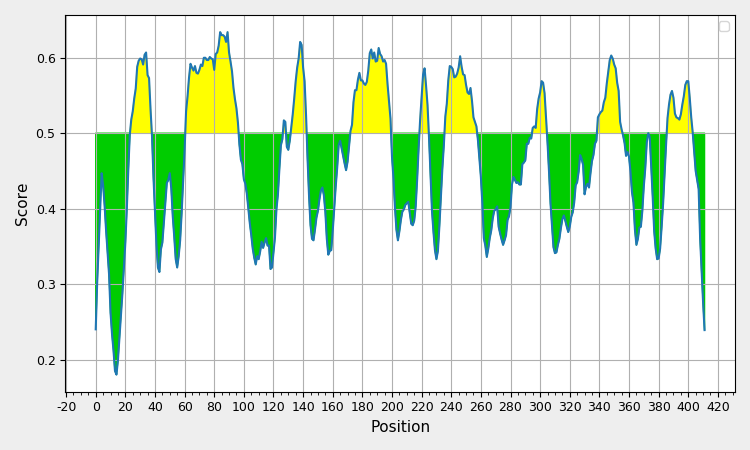 |
| --- | --- | --- | --- | --- | --- | --- | --- | --- | --- | --- | --- | --- | --- | --- | --- | --- | --- | --- | --- | --- | --- | --- | --- | --- | --- | --- | --- | --- | --- | --- | --- | --- | --- | --- | --- | --- | --- | --- | --- | --- | --- | --- | --- | --- | --- | --- | --- | --- | --- | --- | --- | --- | --- | --- | --- | --- | --- | --- | --- | --- | --- |

| **Protein name: 3-carboxymuconate cyclase (Gp60–70)**  **Predicted peptides: Ss185**   | **No.** | **Start** | **End** | **Peptide** | **Length** | | --- | --- | --- | --- | --- | | 1 | 23 | 38 | PTSGHYYDDAESEACL | 16 | | 2 | 64 | 98 | SLLLNHATSTATGGRGGNGINPRGMPAGPDALFSQ | 35 | | 3 | 128 | 129 | PT | 2 | | 4 | 133 | 143 | VVGEPAELPGE | 11 | | 5 | 173 | 200 | YSWYGLGPFDELRTFDLHQTTPPHGPTN | 28 | | 6 | 220 | 226 | GDPAVNN | 7 | | 7 | 238 | 261 | IHSSCYAIPSVSHKGVISSPDGTA | 24 | | 8 | 298 | 306 | LYKTVIPGQ | 9 | | 9 | 341 | 357 | IIGEPIDLTTFNTDPGL | 17 | | 10 | 385 | 393 | NALTKKPVQ | 9 | | 11 | 395 | 395 | A | 1 | | 12 | 397 | 403 | LTPLGLD | 7 | | **BepiPred Linear Epitope Prediction 2.0**  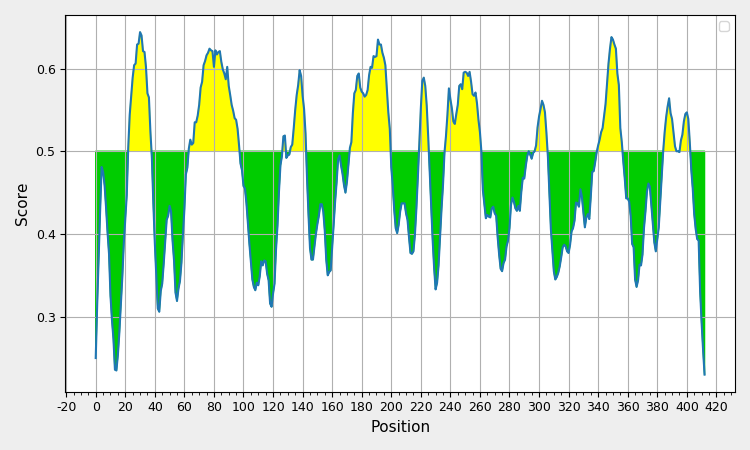 |
| --- | --- | --- | --- | --- | --- | --- | --- | --- | --- | --- | --- | --- | --- | --- | --- | --- | --- | --- | --- | --- | --- | --- | --- | --- | --- | --- | --- | --- | --- | --- | --- | --- | --- | --- | --- | --- | --- | --- | --- | --- | --- | --- | --- | --- | --- | --- | --- | --- | --- | --- | --- | --- | --- | --- | --- | --- | --- | --- | --- | --- | --- | --- | --- | --- | --- | --- |

| **Protein name: 3-carboxymuconate cyclase (Gp60–70)**  **Predicted peptides: S7**   | **No.** | **Start** | **End** | **Peptide** | **Length** | | --- | --- | --- | --- | --- | | 1 | 23 | 38 | PTSGHYYDDAESEACL | 16 | | 2 | 64 | 98 | SLLLNHATSTATGGRGGNGINPRGMPAGPDALFSQ | 35 | | 3 | 128 | 129 | PT | 2 | | 4 | 133 | 143 | VVGEPAELPGE | 11 | | 5 | 173 | 200 | YSWYGLGPFDELRTFDLHQTTPPHGPTN | 28 | | 6 | 220 | 226 | GDPAVNN | 7 | | 7 | 238 | 261 | IHSSCYAIPSVSHKGVISSPDGTA | 24 | | 8 | 298 | 306 | LYKTVIPGQ | 9 | | 9 | 341 | 357 | IIGEPIDLTTFNTDPGL | 17 | | 10 | 385 | 393 | NALTKKPVQ | 9 | | 11 | 395 | 395 | A | 1 | | 12 | 397 | 403 | LTPLGLD | 7 | | **BepiPred Linear Epitope Prediction 2.0**  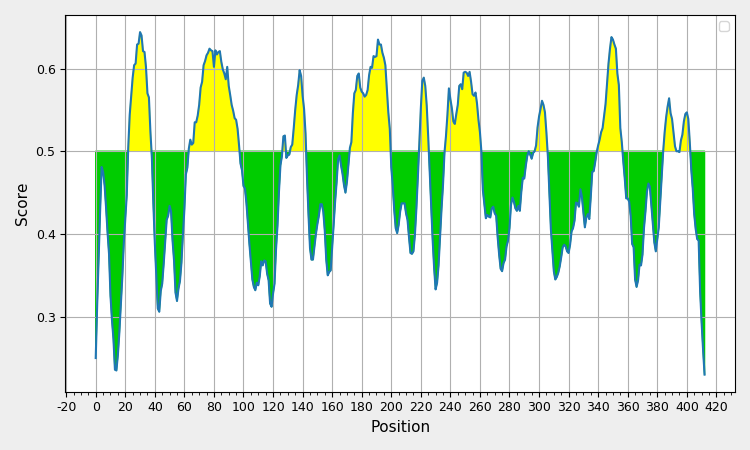 |
| --- | --- | --- | --- | --- | --- | --- | --- | --- | --- | --- | --- | --- | --- | --- | --- | --- | --- | --- | --- | --- | --- | --- | --- | --- | --- | --- | --- | --- | --- | --- | --- | --- | --- | --- | --- | --- | --- | --- | --- | --- | --- | --- | --- | --- | --- | --- | --- | --- | --- | --- | --- | --- | --- | --- | --- | --- | --- | --- | --- | --- | --- | --- | --- | --- | --- | --- |

| **Protein name: 3-carboxymuconate cyclase (Gp60–70)**  **Predicted peptides: ATCC58251**   | **No.** | **Start** | **End** | **Peptide** | **Length** | | --- | --- | --- | --- | --- | | 1 | 23 | 38 | PTSGHYYDDAESEACL | 16 | | 2 | 64 | 98 | SLLLNHATSTATGGRGGNGINPRGMPAGPDALFSQ | 35 | | 3 | 128 | 129 | PT | 2 | | 4 | 133 | 143 | VVGEPAELPGE | 11 | | 5 | 173 | 200 | YSWYGLGPFDELRTFDLHQTTPPHGPTN | 28 | | 6 | 220 | 226 | GDPAVNN | 7 | | 7 | 238 | 261 | IHSSCYAIPSVSHKGVISSPDGTA | 24 | | 8 | 298 | 306 | LYKTVIPGQ | 9 | | 9 | 341 | 357 | IIGEPIDLTTFNTDPGL | 17 | | 10 | 385 | 393 | NALTKKPVQ | 9 | | 11 | 395 | 395 | A | 1 | | 12 | 397 | 403 | LTPLGLD | 7 | | **BepiPred Linear Epitope Prediction 2.0**  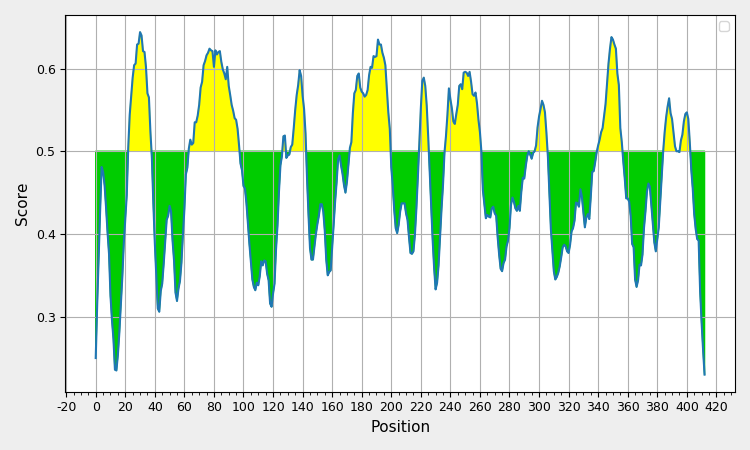 |
| --- | --- | --- | --- | --- | --- | --- | --- | --- | --- | --- | --- | --- | --- | --- | --- | --- | --- | --- | --- | --- | --- | --- | --- | --- | --- | --- | --- | --- | --- | --- | --- | --- | --- | --- | --- | --- | --- | --- | --- | --- | --- | --- | --- | --- | --- | --- | --- | --- | --- | --- | --- | --- | --- | --- | --- | --- | --- | --- | --- | --- | --- | --- | --- | --- | --- | --- |

| **Protein name: 3-carboxymuconate cyclase (Gp60–70)**  **Predicted peptides: Ss143**   | **No.** | **Start** | **End** | **Peptide** | **Length** | | --- | --- | --- | --- | --- | | 1 | 23 | 38 | PTSGHYYDDAESEACL | 16 | | 2 | 65 | 98 | LLLNHATSTATGGRGGNGINPRGMPAGPDALFSQ | 34 | | 3 | 128 | 129 | PT | 2 | | 4 | 133 | 143 | VVGEPAELPGE | 11 | | 5 | 175 | 201 | WYGLGPFDELRTFDLHQTTPPHGPTNT | 27 | | 6 | 220 | 227 | GDPAVNNT | 8 | | 7 | 238 | 262 | IHSSCYAIPSVSHKGVISSPDGTAV | 25 | | 8 | 297 | 307 | TLYKTVIPGQD | 11 | | 9 | 340 | 357 | EIIGEPIDLTTFNTDPGL | 18 | | 10 | 386 | 393 | ALTKKPVQ | 8 | | 11 | 395 | 395 | A | 1 | | 12 | 397 | 403 | LTPLGLD | 7 | | **BepiPred Linear Epitope Prediction 2.0**  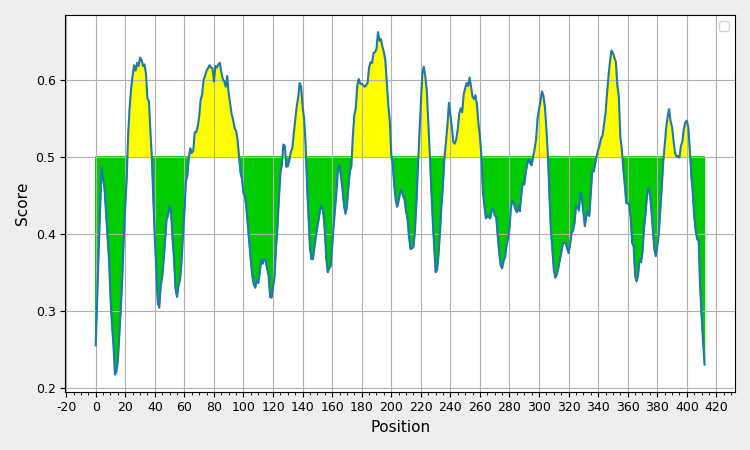 |
| --- | --- | --- | --- | --- | --- | --- | --- | --- | --- | --- | --- | --- | --- | --- | --- | --- | --- | --- | --- | --- | --- | --- | --- | --- | --- | --- | --- | --- | --- | --- | --- | --- | --- | --- | --- | --- | --- | --- | --- | --- | --- | --- | --- | --- | --- | --- | --- | --- | --- | --- | --- | --- | --- | --- | --- | --- | --- | --- | --- | --- | --- | --- | --- | --- | --- | --- |

| **Protein name: 3-carboxymuconate cyclase (Gp60–70)**  **Predicted peptides: 109918**   | **No.** | **Start** | **End** | **Peptide** | **Length** | | --- | --- | --- | --- | --- | | 1 | 23 | 38 | PTSGHYYDDAESEACL | 16 | | 2 | 64 | 98 | SLLLNHATSTATGGRGGNGINPRGMPAGPDALFSQ | 35 | | 3 | 128 | 129 | PT | 2 | | 4 | 133 | 143 | VVGEPAELPGE | 11 | | 5 | 173 | 200 | YSWYGLGPFDELRTFDLHQTTPPHGPTN | 28 | | 6 | 220 | 226 | GDPAVNN | 7 | | 7 | 238 | 261 | IHSSCYAIPSVSHKGVISSPDGTA | 24 | | 8 | 298 | 306 | LYKTVIPGQ | 9 | | 9 | 341 | 357 | IIGEPIDLTTFNTDPGL | 17 | | 10 | 385 | 393 | NALTKKPVQ | 9 | | 11 | 395 | 395 | A | 1 | | 12 | 397 | 403 | LTPLGLD | 7 | | **BepiPred Linear Epitope Prediction 2.0**  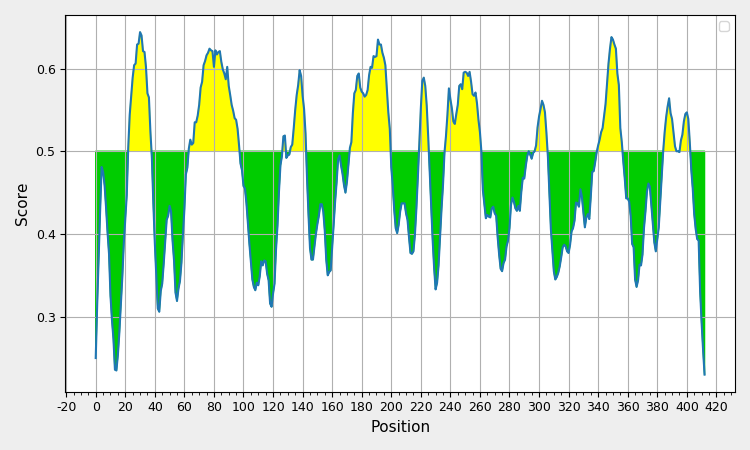 |
| --- | --- | --- | --- | --- | --- | --- | --- | --- | --- | --- | --- | --- | --- | --- | --- | --- | --- | --- | --- | --- | --- | --- | --- | --- | --- | --- | --- | --- | --- | --- | --- | --- | --- | --- | --- | --- | --- | --- | --- | --- | --- | --- | --- | --- | --- | --- | --- | --- | --- | --- | --- | --- | --- | --- | --- | --- | --- | --- | --- | --- | --- | --- | --- | --- | --- | --- |

| **Protein name: 3-carboxymuconate cyclase (Gp60–70)**  **Predicted peptides: Ss476**   | **No.** | **Start** | **End** | **Peptide** | **Length** | | --- | --- | --- | --- | --- | | 1 | 23 | 38 | PTSGHYYDDAESEACL | 16 | | 2 | 64 | 98 | SLLLNHATSTATGGRGGNGINPRGMPAGPDALFSQ | 35 | | 3 | 128 | 129 | PT | 2 | | 4 | 133 | 143 | VVGEPAELPGE | 11 | | 5 | 173 | 200 | YSWYGLGPFDELRTFDLHQTTPPHGPTN | 28 | | 6 | 220 | 226 | GDPAVNN | 7 | | 7 | 238 | 262 | IHSSCYAIPSVSHKGVISSPDGTAV | 25 | | 8 | 298 | 306 | LYKTVIPGQ | 9 | | 9 | 341 | 357 | IIGEPIDLTTFNTDPGL | 17 | | 10 | 385 | 403 | NALTKKPVQHALLTPLGLD | 19 | | **BepiPred Linear Epitope Prediction 2.0**  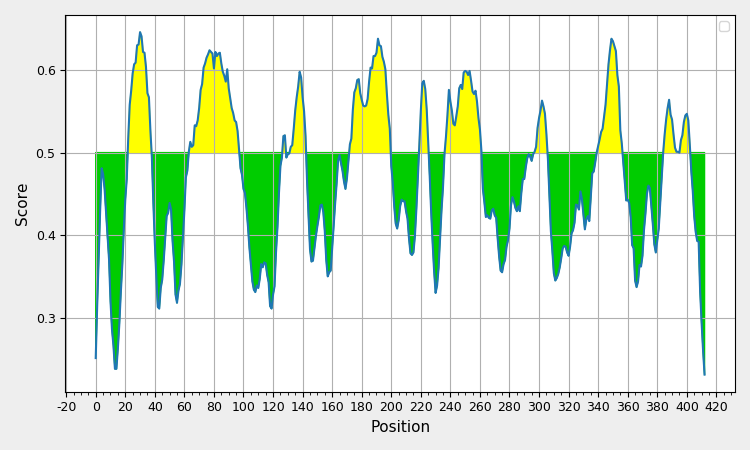 |
| --- | --- | --- | --- | --- | --- | --- | --- | --- | --- | --- | --- | --- | --- | --- | --- | --- | --- | --- | --- | --- | --- | --- | --- | --- | --- | --- | --- | --- | --- | --- | --- | --- | --- | --- | --- | --- | --- | --- | --- | --- | --- | --- | --- | --- | --- | --- | --- | --- | --- | --- | --- | --- | --- | --- | --- | --- |

| **Protein name: 3-carboxymuconate cyclase (Gp60–70)**  **Predicted peptides: Ss478**   | **No.** | **Start** | **End** | **Peptide** | **Length** | | --- | --- | --- | --- | --- | | 1 | 23 | 37 | PTSGHYYDDAESEAC | 15 | | 2 | 62 | 97 | NGSLLLNHATSTATGGRGGNGINPRGMPAGPDALFS | 36 | | 3 | 128 | 129 | PT | 2 | | 4 | 134 | 143 | VGEPAELPGE | 10 | | 5 | 175 | 200 | WYGLGPFDELRTFDLHQTTPPHGPTN | 26 | | 6 | 220 | 226 | GDPAVNN | 7 | | 7 | 238 | 261 | IHSSCYAIPSVSHKGVISSPDGTA | 24 | | 8 | 298 | 306 | LYKTVIPGQ | 9 | | 9 | 341 | 359 | IIGEPIDLTTFNTDPGLTE | 19 | | 10 | 375 | 376 | GT | 2 | | 11 | 388 | 403 | TKKPVQHALLTPLGLD | 16 | | **BepiPred Linear Epitope Prediction 2.0**  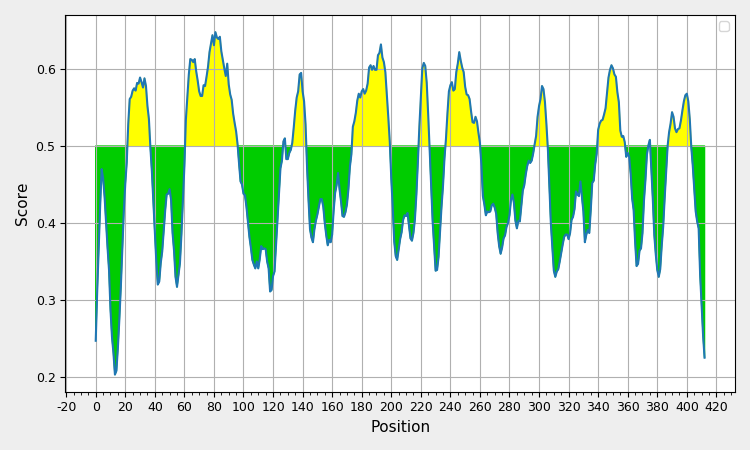 |
| --- | --- | --- | --- | --- | --- | --- | --- | --- | --- | --- | --- | --- | --- | --- | --- | --- | --- | --- | --- | --- | --- | --- | --- | --- | --- | --- | --- | --- | --- | --- | --- | --- | --- | --- | --- | --- | --- | --- | --- | --- | --- | --- | --- | --- | --- | --- | --- | --- | --- | --- | --- | --- | --- | --- | --- | --- | --- | --- | --- | --- | --- |

| **Protein name: 3-carboxymuconate cyclase (Gp60–70)**  **Predicted peptides: Ss477**   | **No.** | **Start** | **End** | **Peptide** | **Length** | | --- | --- | --- | --- | --- | | 1 | 23 | 37 | PTSGHYYDDAESEAC | 15 | | 2 | 62 | 97 | NGSLLLNHATSTATGGRGGNGINPRGMPAGPDALFS | 36 | | 3 | 128 | 129 | PT | 2 | | 4 | 134 | 143 | VGEPAELPGE | 10 | | 5 | 175 | 200 | WYGLGPFDELRTFDLHQTTPPHGPTN | 26 | | 6 | 220 | 226 | GDPAVNN | 7 | | 7 | 238 | 261 | IHSSCYAIPSVSHKGVISSPDGTA | 24 | | 8 | 298 | 306 | LYKTVIPGQ | 9 | | 9 | 341 | 359 | IIGEPIDLTTFNTDPGLTE | 19 | | 10 | 375 | 376 | GT | 2 | | 11 | 388 | 403 | TKKPVQHALLTPLGLD | 16 | | **BepiPred Linear Epitope Prediction 2.0**  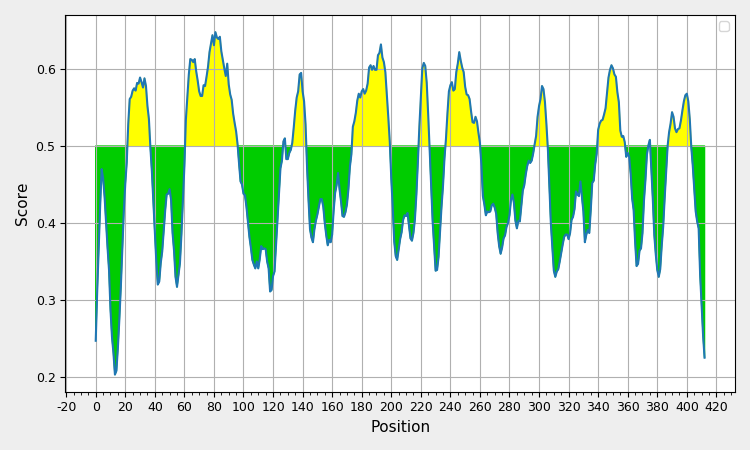 |
| --- | --- | --- | --- | --- | --- | --- | --- | --- | --- | --- | --- | --- | --- | --- | --- | --- | --- | --- | --- | --- | --- | --- | --- | --- | --- | --- | --- | --- | --- | --- | --- | --- | --- | --- | --- | --- | --- | --- | --- | --- | --- | --- | --- | --- | --- | --- | --- | --- | --- | --- | --- | --- | --- | --- | --- | --- | --- | --- | --- | --- | --- |

| **Protein name: 3-carboxymuconate cyclase (Gp60–70)**  **Predicted peptides: Ss176**   | **No.** | **Start** | **End** | **Peptide** | **Length** | | --- | --- | --- | --- | --- | | 1 | 23 | 38 | PTSGHYYDDAESEACL | 16 | | 2 | 64 | 98 | SLLLNHATSTATGGRGGNGINPRGMPAGPDALFSQ | 35 | | 3 | 128 | 129 | PT | 2 | | 4 | 133 | 143 | VVGEPAELPGE | 11 | | 5 | 173 | 200 | YSWYGLGPFDELRTFDLHQTTPPHGPTN | 28 | | 6 | 220 | 226 | GDPAVNN | 7 | | 7 | 238 | 262 | IHSSCYAIPSVSHKGVISSPDGTAV | 25 | | 8 | 298 | 306 | LYKTVIPGQ | 9 | | 9 | 341 | 357 | IIGEPIDLTTFNTDPGL | 17 | | 10 | 385 | 403 | NALTKKPVQHALLTPLGLD | 19 | | **BepiPred Linear Epitope Prediction 2.0**  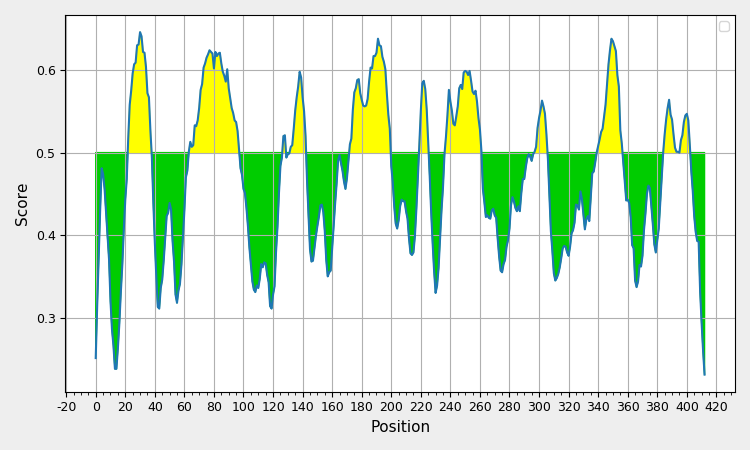 |
| --- | --- | --- | --- | --- | --- | --- | --- | --- | --- | --- | --- | --- | --- | --- | --- | --- | --- | --- | --- | --- | --- | --- | --- | --- | --- | --- | --- | --- | --- | --- | --- | --- | --- | --- | --- | --- | --- | --- | --- | --- | --- | --- | --- | --- | --- | --- | --- | --- | --- | --- | --- | --- | --- | --- | --- | --- |

| **Protein name: 3-carboxymuconate cyclase (Gp60–70)**  **Predicted peptides: Ss529**   | **No.** | **Start** | **End** | **Peptide** | **Length** | | --- | --- | --- | --- | --- | | 1 | 23 | 38 | PTSGHYYDDAESEACL | 16 | | 2 | 64 | 98 | SLLLNHATSTATGGRGGNGINPRGMPAGPDALFSQ | 35 | | 3 | 128 | 129 | PT | 2 | | 4 | 133 | 143 | VVGEPAELPGE | 11 | | 5 | 173 | 200 | YSWYGLGPFDELRTFDLHQTTPPHGPTN | 28 | | 6 | 220 | 226 | GDPAVNN | 7 | | 7 | 238 | 262 | IHSSCYAIPSVSHKGVISSPDGTAV | 25 | | 8 | 294 | 294 | E | 1 | | 9 | 297 | 306 | TLYKTVIPGQ | 10 | | 10 | 341 | 357 | IIGEPIDLTTFNTDPGL | 17 | | 11 | 386 | 395 | ALTKKPVQHA | 10 | | 12 | 397 | 403 | LTPLGLD | 7 | | **BepiPred Linear Epitope Prediction 2.0**  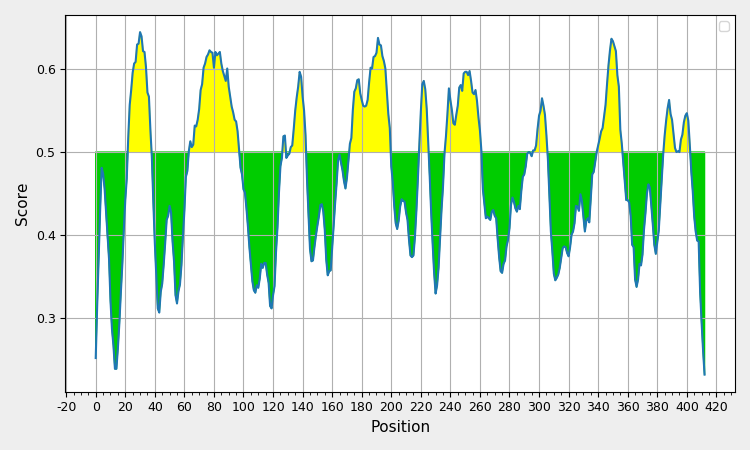 |
| --- | --- | --- | --- | --- | --- | --- | --- | --- | --- | --- | --- | --- | --- | --- | --- | --- | --- | --- | --- | --- | --- | --- | --- | --- | --- | --- | --- | --- | --- | --- | --- | --- | --- | --- | --- | --- | --- | --- | --- | --- | --- | --- | --- | --- | --- | --- | --- | --- | --- | --- | --- | --- | --- | --- | --- | --- | --- | --- | --- | --- | --- | --- | --- | --- | --- | --- |

| **Protein name: 3-carboxymuconate cyclase (Gp60–70)**  **Predicted peptides: Ss530**   | **No.** | **Start** | **End** | **Peptide** | **Length** | | --- | --- | --- | --- | --- | | 1 | 24 | 38 | TSGHYYDDAESEACL | 15 | | 2 | 64 | 97 | SLLLNHATSTATGGRGGNGINPRGMPAGPDALFS | 34 | | 3 | 128 | 129 | PT | 2 | | 4 | 131 | 143 | VTVVGEPAELPGE | 13 | | 5 | 174 | 200 | SWYGLGPFDELRTFDLHQTTPPHGPTN | 27 | | 6 | 220 | 226 | GDPAVNN | 7 | | 7 | 236 | 261 | EHIHSSCYAIPSVSHKGVISSPDGTA | 26 | | 8 | 294 | 294 | E | 1 | | 9 | 298 | 306 | LYKTVIPGQ | 9 | | 10 | 341 | 357 | IIGEPIDLTTFNTDPGL | 17 | | 11 | 374 | 375 | NG | 2 | | 12 | 388 | 403 | TKKPVQYALLTPLGLD | 16 | | **BepiPred Linear Epitope Prediction 2.0**  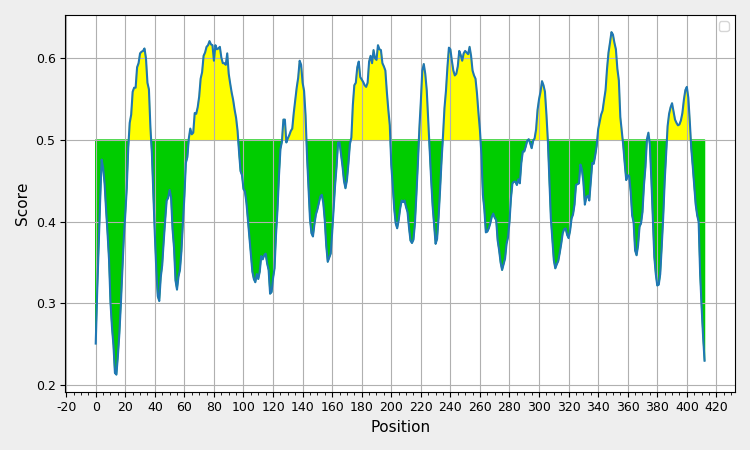 |
| --- | --- | --- | --- | --- | --- | --- | --- | --- | --- | --- | --- | --- | --- | --- | --- | --- | --- | --- | --- | --- | --- | --- | --- | --- | --- | --- | --- | --- | --- | --- | --- | --- | --- | --- | --- | --- | --- | --- | --- | --- | --- | --- | --- | --- | --- | --- | --- | --- | --- | --- | --- | --- | --- | --- | --- | --- | --- | --- | --- | --- | --- | --- | --- | --- | --- | --- |

| **Protein name: 3-carboxymuconate cyclase (Gp60–70)**  **Predicted peptides: Ss39**   | **No.** | **Start** | **End** | **Peptide** | **Length** | | --- | --- | --- | --- | --- | | 1 | 23 | 38 | PTSGHYYDDAESEACL | 16 | | 2 | 65 | 98 | LLLNHATSTATGGRGGNGINPRGMPAGPDALFSQ | 34 | | 3 | 128 | 129 | PT | 2 | | 4 | 133 | 143 | VVGEPAELPGE | 11 | | 5 | 175 | 201 | WYGLGPFDELRTFDLHQTTPPHGPTNT | 27 | | 6 | 220 | 227 | GDPAVNNT | 8 | | 7 | 238 | 261 | IHSSCYAIPSVSHKGVISSPDGTA | 24 | | 8 | 297 | 307 | TLYKTVIPGQD | 11 | | 9 | 340 | 357 | EIIGEPIDLTTFNTDPGL | 18 | | 10 | 386 | 395 | ALTKKPVQHA | 10 | | 11 | 397 | 403 | LTPLGLD | 7 | | **BepiPred Linear Epitope Prediction 2.0**  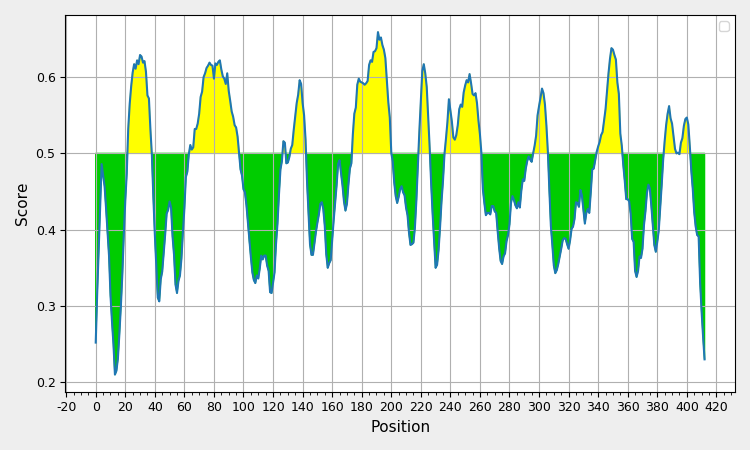 |
| --- | --- | --- | --- | --- | --- | --- | --- | --- | --- | --- | --- | --- | --- | --- | --- | --- | --- | --- | --- | --- | --- | --- | --- | --- | --- | --- | --- | --- | --- | --- | --- | --- | --- | --- | --- | --- | --- | --- | --- | --- | --- | --- | --- | --- | --- | --- | --- | --- | --- | --- | --- | --- | --- | --- | --- | --- | --- | --- | --- | --- | --- |

| **Protein name: 3-carboxymuconate cyclase (Gp60–70)**  **Predicted peptides: Ss64**   | **No.** | **Start** | **End** | **Peptide** | **Length** | | --- | --- | --- | --- | --- | | 1 | 23 | 38 | PTSGHYYDDAESEACL | 16 | | 2 | 62 | 97 | NGSLLLNHSTSTATGGRGGNGINPRGMPAGPDALFS | 36 | | 3 | 128 | 129 | PT | 2 | | 4 | 133 | 143 | VVGEPAELPGE | 11 | | 5 | 173 | 200 | YSWYGLGPFDELRTFDLHQTTPPHGPTN | 28 | | 6 | 220 | 226 | GDPAVNN | 7 | | 7 | 238 | 261 | IHSSCYAIPSVSHKGVISSPDGTA | 24 | | 8 | 298 | 306 | LYKTVIPGQ | 9 | | 9 | 341 | 357 | IIGEPIDLTTFNTDPGL | 17 | | 10 | 385 | 393 | NALTKKPVQ | 9 | | 11 | 397 | 403 | LTPLGLD | 7 | | **BepiPred Linear Epitope Prediction 2.0**  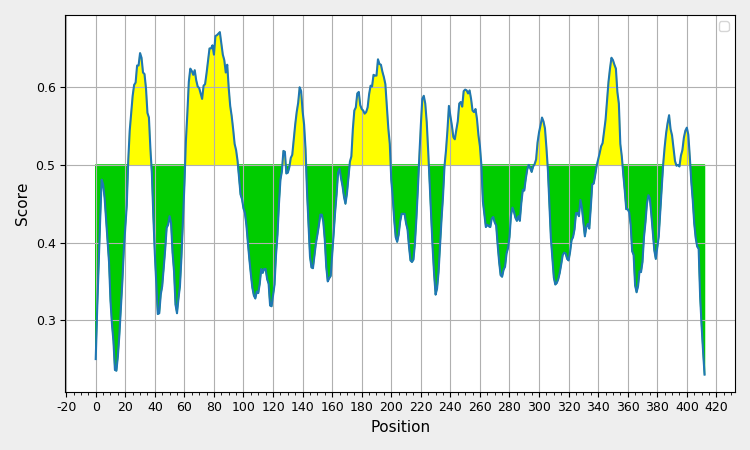 |
| --- | --- | --- | --- | --- | --- | --- | --- | --- | --- | --- | --- | --- | --- | --- | --- | --- | --- | --- | --- | --- | --- | --- | --- | --- | --- | --- | --- | --- | --- | --- | --- | --- | --- | --- | --- | --- | --- | --- | --- | --- | --- | --- | --- | --- | --- | --- | --- | --- | --- | --- | --- | --- | --- | --- | --- | --- | --- | --- | --- | --- | --- |

| **Protein name: 3-carboxymuconate cyclase (Gp60–70)**  **Predicted peptides: S6**   | **No.** | **Start** | **End** | **Peptide** | **Length** | | --- | --- | --- | --- | --- | | 1 | 23 | 38 | PTSGHYYDDAESEACL | 16 | | 2 | 64 | 98 | SLLLNHATSTATGGRGGNGINPRGMPAGPDALFSQ | 35 | | 3 | 128 | 129 | PT | 2 | | 4 | 133 | 143 | VVGEPAELPGE | 11 | | 5 | 173 | 200 | YSWYGLGPFDELRTFDLHQTTPPHGPTN | 28 | | 6 | 220 | 226 | GDPAVNN | 7 | | 7 | 238 | 261 | IHSSCYAIPSVSHKGVISSPDGTA | 24 | | 8 | 294 | 295 | EA | 2 | | 9 | 297 | 297 | T | 1 | | 10 | 299 | 306 | YKTVIPGQ | 8 | | 11 | 341 | 357 | IIGEPIDLTTFNTDPGL | 17 | | 12 | 386 | 393 | ALTKKPVQ | 8 | | 13 | 397 | 403 | LTPLGLD | 7 | | **BepiPred Linear Epitope Prediction 2.0**  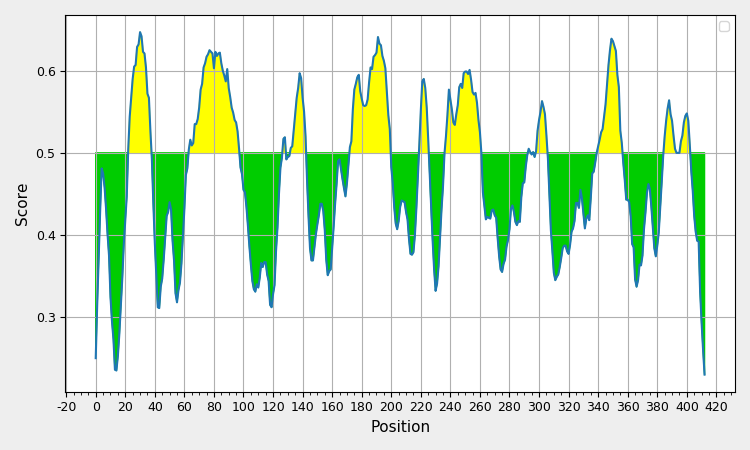 |
| --- | --- | --- | --- | --- | --- | --- | --- | --- | --- | --- | --- | --- | --- | --- | --- | --- | --- | --- | --- | --- | --- | --- | --- | --- | --- | --- | --- | --- | --- | --- | --- | --- | --- | --- | --- | --- | --- | --- | --- | --- | --- | --- | --- | --- | --- | --- | --- | --- | --- | --- | --- | --- | --- | --- | --- | --- | --- | --- | --- | --- | --- | --- | --- | --- | --- | --- | --- | --- | --- | --- | --- |

| **Protein name: 3-carboxymuconate cyclase (Gp60–70)**  **Predicted peptides: Ss118**   | **No.** | **Start** | **End** | **Peptide** | **Length** | | --- | --- | --- | --- | --- | | 1 | 24 | 39 | TSGHYYDDAESEACLN | 16 | | 2 | 64 | 97 | SLLLNHATSTATGGRGGNGINPRGMPAGPDALFS | 34 | | 3 | 128 | 129 | PT | 2 | | 4 | 133 | 143 | VVGEPAELPGE | 11 | | 5 | 175 | 200 | WYGLGPFDELRTFDLHQTTPPHGPTN | 26 | | 6 | 220 | 226 | GDPAVNN | 7 | | 7 | 238 | 261 | IHSSCYAIPSVSHKGVISSPDGTA | 24 | | 8 | 297 | 307 | TLYKTVIPGQD | 11 | | 9 | 340 | 357 | EIIGEPIDLTTFNTDPGL | 18 | | 10 | 386 | 395 | ALTKKPVQHA | 10 | | 11 | 397 | 403 | LTPLGLD | 7 | | **BepiPred Linear Epitope Prediction 2.0**  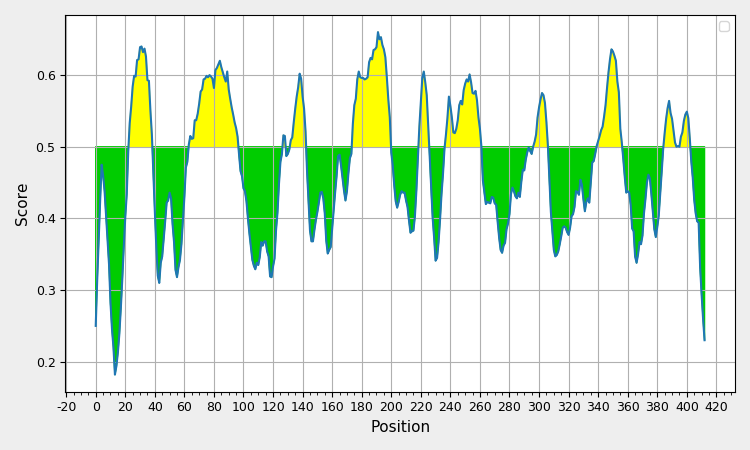 |
| --- | --- | --- | --- | --- | --- | --- | --- | --- | --- | --- | --- | --- | --- | --- | --- | --- | --- | --- | --- | --- | --- | --- | --- | --- | --- | --- | --- | --- | --- | --- | --- | --- | --- | --- | --- | --- | --- | --- | --- | --- | --- | --- | --- | --- | --- | --- | --- | --- | --- | --- | --- | --- | --- | --- | --- | --- | --- | --- | --- | --- | --- |

| **Protein name: 3-carboxymuconate cyclase (Gp60–70)**  **Predicted peptides: Ss527**   | **No.** | **Start** | **End** | **Peptide** | **Length** | | --- | --- | --- | --- | --- | | 1 | 23 | 38 | PTSGHYYDDAESEACL | 16 | | 2 | 64 | 98 | SLLLNHATSTATGGRGGNGINPRGMPAGPDALFSQ | 35 | | 3 | 134 | 143 | VGEPAELPGE | 10 | | 4 | 175 | 200 | WYGLGPFDELRTFDLHQTTPPHGPTN | 26 | | 5 | 220 | 226 | GDPAVNN | 7 | | 6 | 239 | 259 | HSSCYAIPSVSHKGVISSPDG | 21 | | 7 | 300 | 306 | KTVIPGQ | 7 | | 8 | 345 | 356 | PIDLTTFNTDPG | 12 | | 9 | 388 | 403 | TKKPVQHALLTPLGLD | 16 | | **BepiPred Linear Epitope Prediction 2.0**  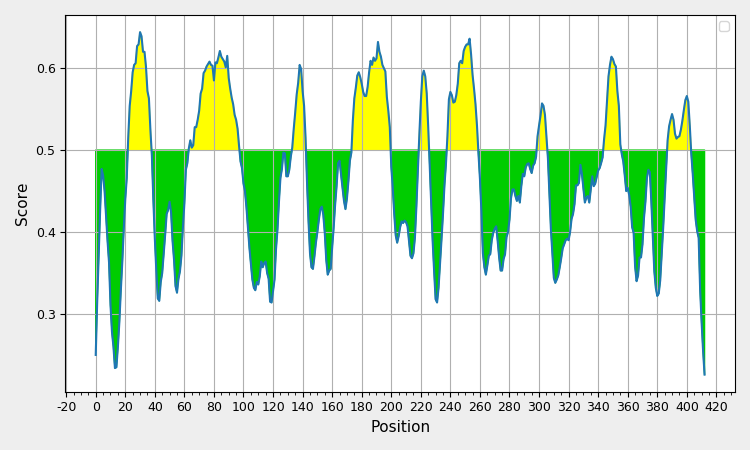 |
| --- | --- | --- | --- | --- | --- | --- | --- | --- | --- | --- | --- | --- | --- | --- | --- | --- | --- | --- | --- | --- | --- | --- | --- | --- | --- | --- | --- | --- | --- | --- | --- | --- | --- | --- | --- | --- | --- | --- | --- | --- | --- | --- | --- | --- | --- | --- | --- | --- | --- | --- | --- |

| **Protein name: 3-carboxymuconate cyclase (Gp60–70)**  **Predicted peptides: Ss162**   | **No.** | **Start** | **End** | **Peptide** | **Length** | | --- | --- | --- | --- | --- | | 1 | 23 | 38 | PTSGHYYDDAESEACL | 16 | | 2 | 64 | 98 | SLLLNHATSTATGGRGGNGINPRGMPAGPDALFSQ | 35 | | 3 | 134 | 143 | VGEPAELPGE | 10 | | 4 | 175 | 200 | WYGLGPFDELRTFDLHQTTPPHGPTN | 26 | | 5 | 220 | 226 | GDPAVNN | 7 | | 6 | 239 | 259 | HSSCYAIPSVSHKGVISSPDG | 21 | | 7 | 300 | 306 | KTVIPGQ | 7 | | 8 | 345 | 356 | PIDLTTFNTDPG | 12 | | 9 | 388 | 403 | TKKPVQHALLTPLGLD | 16 | | **BepiPred Linear Epitope Prediction 2.0**  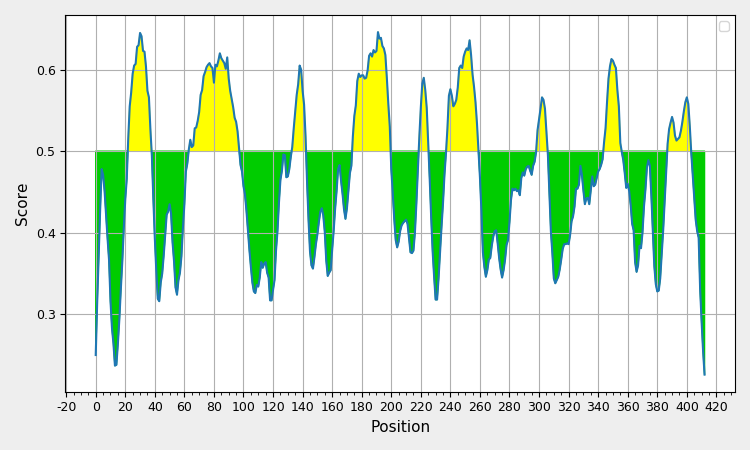 |
| --- | --- | --- | --- | --- | --- | --- | --- | --- | --- | --- | --- | --- | --- | --- | --- | --- | --- | --- | --- | --- | --- | --- | --- | --- | --- | --- | --- | --- | --- | --- | --- | --- | --- | --- | --- | --- | --- | --- | --- | --- | --- | --- | --- | --- | --- | --- | --- | --- | --- | --- | --- |

| **Protein name: 3-carboxymuconate cyclase (Gp60–70)**  **Predicted peptides: Ss164**   | **No.** | **Start** | **End** | **Peptide** | **Length** | | --- | --- | --- | --- | --- | | 1 | 23 | 38 | PTSGHYYDDAESEACL | 16 | | 2 | 64 | 98 | SLLLNHATSTATGGRGGNGINPRGMPAGPDALFSQ | 35 | | 3 | 128 | 129 | PT | 2 | | 4 | 133 | 143 | VVGEPAELPGE | 11 | | 5 | 173 | 200 | YSWYGLGPFDELRTFDLHQTTPPHGPTN | 28 | | 6 | 220 | 226 | GDPAVNN | 7 | | 7 | 238 | 261 | IHSSCYAIPSVSHKGVISSPDGTA | 24 | | 8 | 298 | 306 | LYKTVIPGQ | 9 | | 9 | 341 | 357 | IIGEPIDLTTFNTDPGL | 17 | | 10 | 385 | 393 | NALTKKPVQ | 9 | | 11 | 395 | 395 | A | 1 | | 12 | 397 | 403 | LTPLGLD | 7 | | **BepiPred Linear Epitope Prediction 2.0**  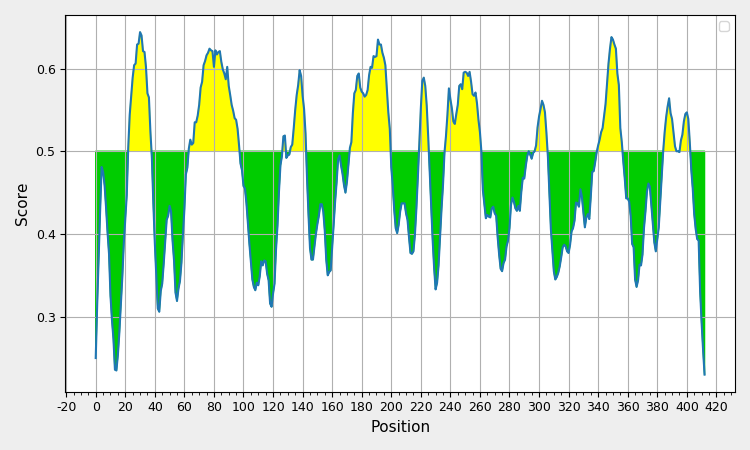 |
| --- | --- | --- | --- | --- | --- | --- | --- | --- | --- | --- | --- | --- | --- | --- | --- | --- | --- | --- | --- | --- | --- | --- | --- | --- | --- | --- | --- | --- | --- | --- | --- | --- | --- | --- | --- | --- | --- | --- | --- | --- | --- | --- | --- | --- | --- | --- | --- | --- | --- | --- | --- | --- | --- | --- | --- | --- | --- | --- | --- | --- | --- | --- | --- | --- | --- | --- |

| **Protein name: 3-carboxymuconate cyclase (Gp60–70)**  **Predicted peptides: Ss234**   | **No.** | **Start** | **End** | **Peptide** | **Length** | | --- | --- | --- | --- | --- | | 1 | 23 | 38 | PTSGHYYDDAESEACL | 16 | | 2 | 64 | 98 | SLLLNHATSTATGGRGGNGINPRGMPAGPDALFSQ | 35 | | 3 | 128 | 129 | PT | 2 | | 4 | 133 | 143 | VVGEPAELPGE | 11 | | 5 | 173 | 200 | YSWYGLGPFDELRTFDLHQTTPPHGPTN | 28 | | 6 | 220 | 226 | GDPAVNN | 7 | | 7 | 238 | 261 | IHSSCYAIPSVSHKGVISSPDGTA | 24 | | 8 | 298 | 306 | LYKTVIPGQ | 9 | | 9 | 341 | 357 | IIGEPIDLTTFNTDPGL | 17 | | 10 | 385 | 393 | NALTKKPVQ | 9 | | 11 | 395 | 395 | A | 1 | | 12 | 397 | 403 | LTPLGLD | 7 | | **BepiPred Linear Epitope Prediction 2.0**  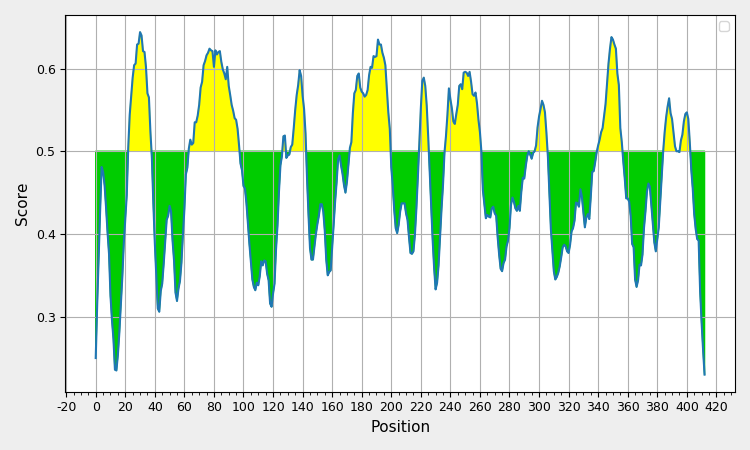 |
| --- | --- | --- | --- | --- | --- | --- | --- | --- | --- | --- | --- | --- | --- | --- | --- | --- | --- | --- | --- | --- | --- | --- | --- | --- | --- | --- | --- | --- | --- | --- | --- | --- | --- | --- | --- | --- | --- | --- | --- | --- | --- | --- | --- | --- | --- | --- | --- | --- | --- | --- | --- | --- | --- | --- | --- | --- | --- | --- | --- | --- | --- | --- | --- | --- | --- | --- |

| **Protein name: 3-carboxymuconate cyclase (Gp60–70)**  **Predicted peptides: Ss229**   | **No.** | **Start** | **End** | **Peptide** | **Length** | | --- | --- | --- | --- | --- | | 1 | 23 | 38 | PTSGHYYDDAESEACL | 16 | | 2 | 64 | 98 | SLLLNHATSTATGGRGGNGINPRGMPAGPDALFSQ | 35 | | 3 | 128 | 129 | PT | 2 | | 4 | 133 | 143 | VVGEPAELPGE | 11 | | 5 | 173 | 200 | YSWYGLGPFDELRTFDLHQTTPPHGPTN | 28 | | 6 | 220 | 226 | GDPAVNN | 7 | | 7 | 238 | 261 | IHSSCYAIPSVSHKGVISSPDGTA | 24 | | 8 | 298 | 306 | LYKTVIPGQ | 9 | | 9 | 341 | 357 | IIGEPIDLTTFNTDPGL | 17 | | 10 | 385 | 393 | NALTKKPVQ | 9 | | 11 | 395 | 395 | A | 1 | | 12 | 397 | 403 | LTPLGLD | 7 | | **BepiPred Linear Epitope Prediction 2.0**  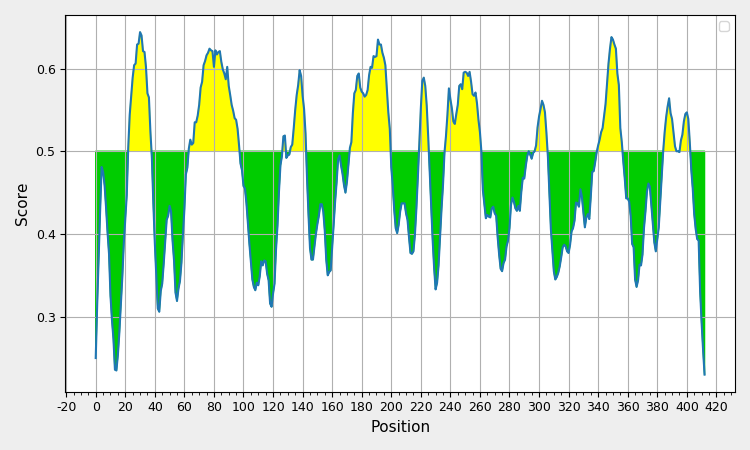 |
| --- | --- | --- | --- | --- | --- | --- | --- | --- | --- | --- | --- | --- | --- | --- | --- | --- | --- | --- | --- | --- | --- | --- | --- | --- | --- | --- | --- | --- | --- | --- | --- | --- | --- | --- | --- | --- | --- | --- | --- | --- | --- | --- | --- | --- | --- | --- | --- | --- | --- | --- | --- | --- | --- | --- | --- | --- | --- | --- | --- | --- | --- | --- | --- | --- | --- | --- |

| **Protein name: 3-carboxymuconate cyclase (Gp60–70)**  **Predicted peptides: Ss231**   | **No.** | **Start** | **End** | **Peptide** | **Length** | | --- | --- | --- | --- | --- | | 1 | 23 | 38 | PTSGHYYDDAESEACL | 16 | | 2 | 64 | 98 | SLLLNHATSTATGGRGGNGINPRGMPAGPDALFSQ | 35 | | 3 | 128 | 129 | PT | 2 | | 4 | 133 | 143 | VVGEPAELPGE | 11 | | 5 | 173 | 200 | YSWYGLGPFDELRTFDLHQTTPPHGPTN | 28 | | 6 | 220 | 226 | GDPAVNN | 7 | | 7 | 238 | 261 | IHSSCYAIPSVSHKGVISSPDGTA | 24 | | 8 | 298 | 306 | LYKTVIPGQ | 9 | | 9 | 341 | 357 | IIGEPIDLTTFNTDPGL | 17 | | 10 | 385 | 393 | NALTKKPVQ | 9 | | 11 | 395 | 395 | A | 1 | | 12 | 397 | 403 | LTPLGLD | 7 | | **BepiPred Linear Epitope Prediction 2.0**  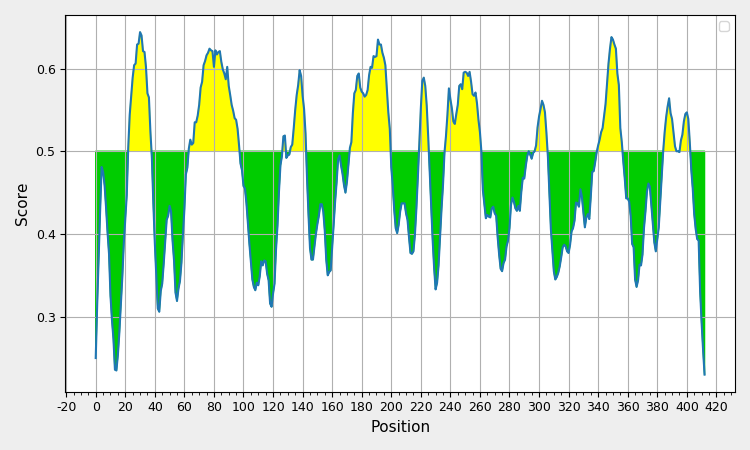 |
| --- | --- | --- | --- | --- | --- | --- | --- | --- | --- | --- | --- | --- | --- | --- | --- | --- | --- | --- | --- | --- | --- | --- | --- | --- | --- | --- | --- | --- | --- | --- | --- | --- | --- | --- | --- | --- | --- | --- | --- | --- | --- | --- | --- | --- | --- | --- | --- | --- | --- | --- | --- | --- | --- | --- | --- | --- | --- | --- | --- | --- | --- | --- | --- | --- | --- | --- |

| **Protein name: 3-carboxymuconate cyclase (Gp60–70)**  **Predicted peptides: Ss228**   | **No.** | **Start** | **End** | **Peptide** | **Length** | | --- | --- | --- | --- | --- | | 1 | 23 | 38 | PTSGHYYDDAESEACL | 16 | | 2 | 64 | 98 | SLLLNHATSTATGGRGGNGINPRGMPAGPDALFSQ | 35 | | 3 | 128 | 129 | PT | 2 | | 4 | 133 | 143 | VVGEPAELPGE | 11 | | 5 | 173 | 200 | YSWYGLGPFDELRTFDLHQTTPPHGPTN | 28 | | 6 | 220 | 226 | GDPAVNN | 7 | | 7 | 238 | 261 | IHSSCYAIPSVSHKGVISSPDGTA | 24 | | 8 | 298 | 306 | LYKTVIPGQ | 9 | | 9 | 341 | 357 | IIGEPIDLTTFNTDPGL | 17 | | 10 | 385 | 393 | NALTKKPVQ | 9 | | 11 | 395 | 395 | A | 1 | | 12 | 397 | 403 | LTPLGLD | 7 | | **BepiPred Linear Epitope Prediction 2.0**  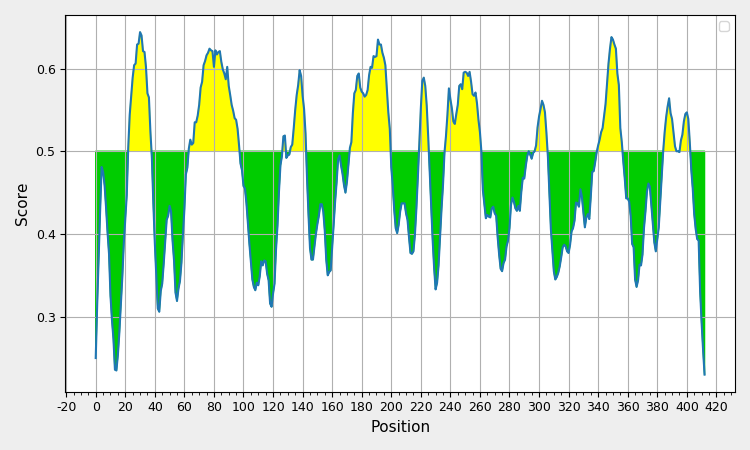 |
| --- | --- | --- | --- | --- | --- | --- | --- | --- | --- | --- | --- | --- | --- | --- | --- | --- | --- | --- | --- | --- | --- | --- | --- | --- | --- | --- | --- | --- | --- | --- | --- | --- | --- | --- | --- | --- | --- | --- | --- | --- | --- | --- | --- | --- | --- | --- | --- | --- | --- | --- | --- | --- | --- | --- | --- | --- | --- | --- | --- | --- | --- | --- | --- | --- | --- | --- |

| **Protein name: 3-carboxymuconate cyclase (Gp60–70)**  **Predicted peptides: Ss577**   | **No.** | **Start** | **End** | **Peptide** | **Length** | | --- | --- | --- | --- | --- | | 1 | 23 | 38 | PTSGHYYDDAESEACL | 16 | | 2 | 64 | 98 | SLLLNHATSTATGGRGGNGINPRGMPAGPDALFSQ | 35 | | 3 | 134 | 142 | VGEPAALPG | 9 | | 4 | 175 | 200 | WYGLGPFDELRTFDLHQTTPPHGPTN | 26 | | 5 | 220 | 226 | GDPAVNN | 7 | | 6 | 239 | 259 | HSSCYAIPSVSHKGVISSPDG | 21 | | 7 | 299 | 306 | YKTVIPGQ | 8 | | 8 | 345 | 356 | PIDLTTFNTDPG | 12 | | 9 | 375 | 375 | G | 1 | | 10 | 388 | 403 | TKKPVQHALLTPLGLD | 16 | | **BepiPred Linear Epitope Prediction 2.0**  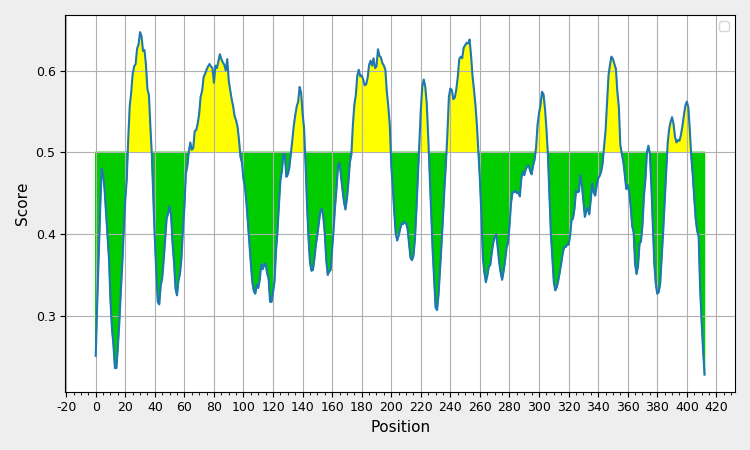 |
| --- | --- | --- | --- | --- | --- | --- | --- | --- | --- | --- | --- | --- | --- | --- | --- | --- | --- | --- | --- | --- | --- | --- | --- | --- | --- | --- | --- | --- | --- | --- | --- | --- | --- | --- | --- | --- | --- | --- | --- | --- | --- | --- | --- | --- | --- | --- | --- | --- | --- | --- | --- | --- | --- | --- | --- | --- |

| **Protein name: 3-carboxymuconate cyclase (Gp60–70)**  **Predicted peptides: Ss554**   | **No.** | **Start** | **End** | **Peptide** | **Length** | | --- | --- | --- | --- | --- | | 1 | 24 | 38 | TSGHYYDDAESEACL | 15 | | 2 | 64 | 98 | SLLLNHATSTATGGRGGNGINPRGMPACPDALFSQ | 35 | | 3 | 128 | 129 | PT | 2 | | 4 | 133 | 143 | VVGEPAELPGE | 11 | | 5 | 173 | 200 | YSWYGLGPFDELRTFDLHQTTPPHGPTN | 28 | | 6 | 220 | 226 | GDPAVNN | 7 | | 7 | 239 | 259 | HSSCYAIPSVSHKGVISSPDG | 21 | | 8 | 298 | 306 | LYKTVIPGQ | 9 | | 9 | 341 | 357 | IIGEPIDLTTFNTDPGL | 17 | | 10 | 386 | 403 | ALTKKPVQHALLTPLGLD | 18 | | **BepiPred Linear Epitope Prediction 2.0**  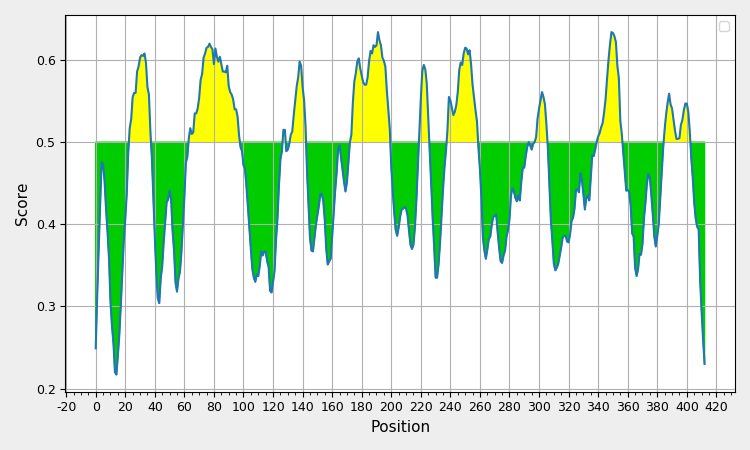 |
| --- | --- | --- | --- | --- | --- | --- | --- | --- | --- | --- | --- | --- | --- | --- | --- | --- | --- | --- | --- | --- | --- | --- | --- | --- | --- | --- | --- | --- | --- | --- | --- | --- | --- | --- | --- | --- | --- | --- | --- | --- | --- | --- | --- | --- | --- | --- | --- | --- | --- | --- | --- | --- | --- | --- | --- | --- |

| **Protein name: 3-carboxymuconate cyclase (Gp60–70)**  **Predicted peptides: Ss447**   | **No.** | **Start** | **End** | **Peptide** | **Length** | | --- | --- | --- | --- | --- | | 1 | 23 | 38 | PTSGHYYDDAESEACL | 16 | | 2 | 64 | 98 | SLLLNHATSTATGGRGGNGINPRGMPAGPDALFSQ | 35 | | 3 | 128 | 129 | PT | 2 | | 4 | 133 | 143 | VVGEPAKLPGE | 11 | | 5 | 175 | 201 | WYGLGPFDELRTFDLHQTTPPHGPTNT | 27 | | 6 | 220 | 227 | GDPAVNNT | 8 | | 7 | 238 | 261 | IHSSCYAIPSVSHKGVISSPDGTA | 24 | | 8 | 294 | 295 | EA | 2 | | 9 | 297 | 307 | TLYKTVIPGQD | 11 | | 10 | 340 | 357 | EIIGEPIDLTTFNTDPGL | 18 | | 11 | 385 | 403 | NALTKKPVQHALLTPLGLD | 19 | | **BepiPred Linear Epitope Prediction 2.0**  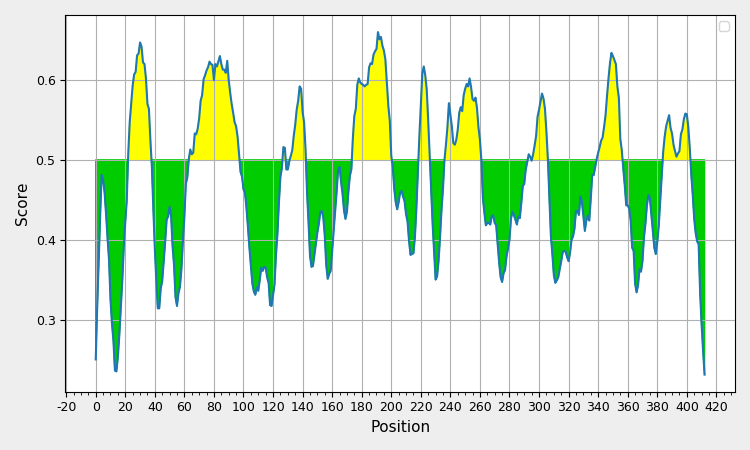 |
| --- | --- | --- | --- | --- | --- | --- | --- | --- | --- | --- | --- | --- | --- | --- | --- | --- | --- | --- | --- | --- | --- | --- | --- | --- | --- | --- | --- | --- | --- | --- | --- | --- | --- | --- | --- | --- | --- | --- | --- | --- | --- | --- | --- | --- | --- | --- | --- | --- | --- | --- | --- | --- | --- | --- | --- | --- | --- | --- | --- | --- | --- |

| **Protein name: 3-carboxymuconate cyclase (Gp60–70)**  **Predicted peptides: Ss175**   | **No.** | **Start** | **End** | **Peptide** | **Length** | | --- | --- | --- | --- | --- | | 1 | 23 | 38 | PTSGHYYDDAESEACL | 16 | | 2 | 64 | 97 | SLLLNHATSTATGGRGGNGINPRGMPAGPDALVS | 34 | | 3 | 128 | 129 | PT | 2 | | 4 | 133 | 143 | VVGEPAELPGE | 11 | | 5 | 166 | 166 | A | 1 | | 6 | 175 | 200 | WYGLGPFDELRTFDLHQTTPPHGPTN | 26 | | 7 | 220 | 225 | GDPAVN | 6 | | 8 | 237 | 261 | HIHSSCYAIPSVSHKGVISSPDGTA | 25 | | 9 | 299 | 306 | YKTVIPGQ | 8 | | 10 | 343 | 356 | GEPIDLTTFNTDPG | 14 | | 11 | 374 | 375 | NG | 2 | | 12 | 388 | 403 | TKKPVQYALLTPLGLD | 16 | | **BepiPred Linear Epitope Prediction 2.0**  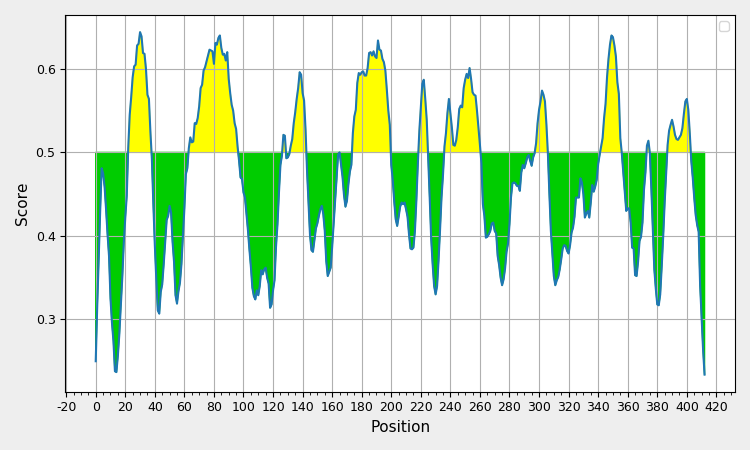 |
| --- | --- | --- | --- | --- | --- | --- | --- | --- | --- | --- | --- | --- | --- | --- | --- | --- | --- | --- | --- | --- | --- | --- | --- | --- | --- | --- | --- | --- | --- | --- | --- | --- | --- | --- | --- | --- | --- | --- | --- | --- | --- | --- | --- | --- | --- | --- | --- | --- | --- | --- | --- | --- | --- | --- | --- | --- | --- | --- | --- | --- | --- | --- | --- | --- | --- | --- |

| **Protein name: 3-carboxymuconate cyclase (Gp60–70)**  **Predicted peptides: Ss24**   | **No.** | **Start** | **End** | **Peptide** | **Length** | | --- | --- | --- | --- | --- | | 1 | 24 | 38 | TSGHYYDDAESEACL | 15 | | 2 | 64 | 96 | SLLLNHATSTATGGRGGNGINPRGMPAGPDALF | 33 | | 3 | 127 | 129 | DPT | 3 | | 4 | 131 | 143 | VTVVGEPAELPGE | 13 | | 5 | 175 | 200 | WYGLGPFDELRTFDLHQTTPPHGPTN | 26 | | 6 | 220 | 226 | GDPAVNN | 7 | | 7 | 236 | 261 | EHIHSSCYAIPSVSHKGVISTPDGTA | 26 | | 8 | 293 | 295 | EEA | 3 | | 9 | 297 | 306 | TLYKTVIPGQ | 10 | | 10 | 341 | 357 | IIGEPIDLTTFNTDPGL | 17 | | 11 | 386 | 403 | ALTKKPVQHALLTPLGLD | 18 | | **BepiPred Linear Epitope Prediction 2.0**  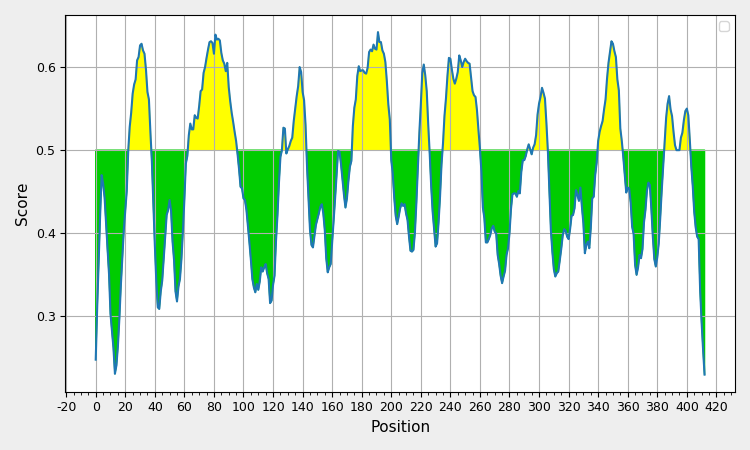 |
| --- | --- | --- | --- | --- | --- | --- | --- | --- | --- | --- | --- | --- | --- | --- | --- | --- | --- | --- | --- | --- | --- | --- | --- | --- | --- | --- | --- | --- | --- | --- | --- | --- | --- | --- | --- | --- | --- | --- | --- | --- | --- | --- | --- | --- | --- | --- | --- | --- | --- | --- | --- | --- | --- | --- | --- | --- | --- | --- | --- | --- | --- |

| **Protein name: 3-carboxymuconate cyclase (Gp60–70)**  **Predicted peptides: S13**   | **No.** | **Start** | **End** | **Peptide** | **Length** | | --- | --- | --- | --- | --- | | 1 | 24 | 38 | TSGHYYDDAESEACL | 15 | | 2 | 64 | 96 | SLLLNHATSTATGGRGGNGINPRGMPAGPDALF | 33 | | 3 | 127 | 129 | DPT | 3 | | 4 | 131 | 143 | VTVVGEPAELPGE | 13 | | 5 | 175 | 200 | WYGLGPFDELRTFDLHQTTPPHGPTN | 26 | | 6 | 220 | 226 | GDPAVNN | 7 | | 7 | 236 | 261 | EHIHSSCYAIPSVSHKGVISTPDGTA | 26 | | 8 | 293 | 295 | EEA | 3 | | 9 | 297 | 306 | TLYKTVIPGQ | 10 | | 10 | 341 | 357 | IIGEPIDLTTFNTDPGL | 17 | | 11 | 386 | 403 | ALTKKPVQHALLTPLGLD | 18 | | **BepiPred Linear Epitope Prediction 2.0**  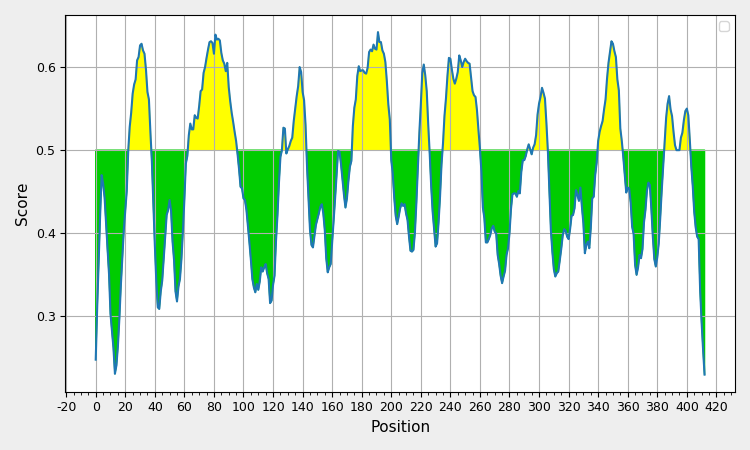 |
| --- | --- | --- | --- | --- | --- | --- | --- | --- | --- | --- | --- | --- | --- | --- | --- | --- | --- | --- | --- | --- | --- | --- | --- | --- | --- | --- | --- | --- | --- | --- | --- | --- | --- | --- | --- | --- | --- | --- | --- | --- | --- | --- | --- | --- | --- | --- | --- | --- | --- | --- | --- | --- | --- | --- | --- | --- | --- | --- | --- | --- | --- |

| **Protein name: 3-carboxymuconate cyclase (Gp60–70)**  **Predicted peptides: Ss564**   | **No.** | **Start** | **End** | **Peptide** | **Length** | | --- | --- | --- | --- | --- | | 1 | 23 | 38 | PTSGHYYDDAESEACL | 16 | | 2 | 65 | 98 | LLLNHATSTATGGRGGNGINPRGMPAGPDALFSQ | 34 | | 3 | 128 | 129 | PT | 2 | | 4 | 133 | 143 | VVGEPAELPGE | 11 | | 5 | 175 | 201 | WYGLGPFDELRTFDLHQTTPPHGPTNT | 27 | | 6 | 220 | 227 | GDPAVNNT | 8 | | 7 | 238 | 262 | IHSSCYAIPSVSHKGVISSPDGTAI | 25 | | 8 | 298 | 306 | LYKTVIPGQ | 9 | | 9 | 340 | 357 | EIIGEPIDLTTFNTDPGL | 18 | | 10 | 386 | 393 | ALTKKPVQ | 8 | | 11 | 397 | 403 | LTPLGLD | 7 | | **BepiPred Linear Epitope Prediction 2.0**  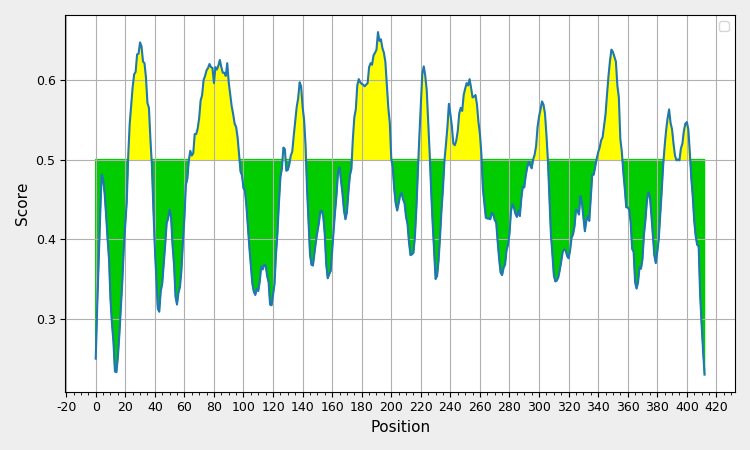 |
| --- | --- | --- | --- | --- | --- | --- | --- | --- | --- | --- | --- | --- | --- | --- | --- | --- | --- | --- | --- | --- | --- | --- | --- | --- | --- | --- | --- | --- | --- | --- | --- | --- | --- | --- | --- | --- | --- | --- | --- | --- | --- | --- | --- | --- | --- | --- | --- | --- | --- | --- | --- | --- | --- | --- | --- | --- | --- | --- | --- | --- | --- |

| **Protein name: 3-carboxymuconate cyclase (Gp60–70)**  **Predicted peptides: Ss567**   | **No.** | **Start** | **End** | **Peptide** | **Length** | | --- | --- | --- | --- | --- | | 1 | 23 | 38 | PTSGHYYDDAESEACL | 16 | | 2 | 65 | 98 | LLLNHATSTATGGRGGNGINPRGMPAGPDALFSQ | 34 | | 3 | 128 | 129 | PT | 2 | | 4 | 133 | 143 | VVGEPAELPGE | 11 | | 5 | 175 | 201 | WYGLGPFDELRTFDLHQTTPPHGPTNT | 27 | | 6 | 220 | 227 | GDPAVNNT | 8 | | 7 | 238 | 262 | IHSSCYAIPSVSHKGVISSPDGTAI | 25 | | 8 | 298 | 306 | LYKTVIPGQ | 9 | | 9 | 340 | 357 | EIIGEPIDLTTFNTDPGL | 18 | | 10 | 386 | 393 | ALTKKPVQ | 8 | | 11 | 397 | 403 | LTPLGLD | 7 | | **BepiPred Linear Epitope Prediction 2.0**  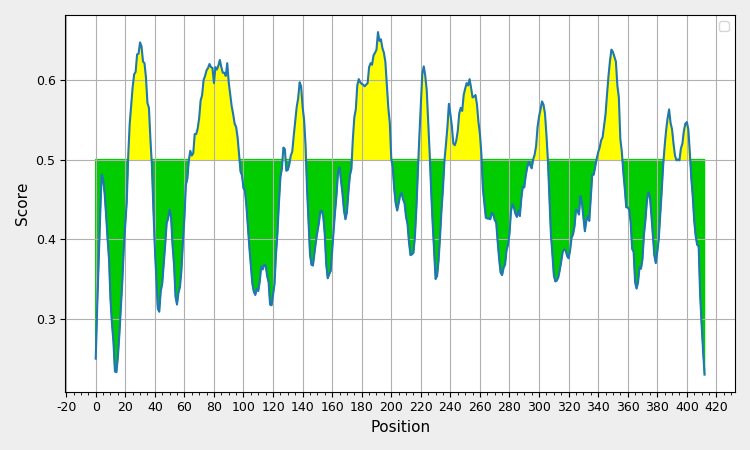 |
| --- | --- | --- | --- | --- | --- | --- | --- | --- | --- | --- | --- | --- | --- | --- | --- | --- | --- | --- | --- | --- | --- | --- | --- | --- | --- | --- | --- | --- | --- | --- | --- | --- | --- | --- | --- | --- | --- | --- | --- | --- | --- | --- | --- | --- | --- | --- | --- | --- | --- | --- | --- | --- | --- | --- | --- | --- | --- | --- | --- | --- | --- |

| **Protein name: 3-carboxymuconate cyclase (Gp60–70)**  **Predicted peptides: Ss59**   | **No.** | **Start** | **End** | **Peptide** | **Length** | | --- | --- | --- | --- | --- | | 1 | 18 | 38 | RTSAHPTSGHYYDDAESEACL | 21 | | 2 | 65 | 97 | LLLNHATSTATGGRGGNGINPRGMPAGPDALFS | 33 | | 3 | 128 | 129 | PT | 2 | | 4 | 133 | 143 | VVGEPAELPGE | 11 | | 5 | 175 | 200 | WYGLGPFDELRTFDLHQTTPPHGPTN | 26 | | 6 | 220 | 226 | GDPAVNN | 7 | | 7 | 237 | 260 | HIHSSCYAIPSVSHKGVISSPDGT | 24 | | 8 | 299 | 306 | YKTVIPGQ | 8 | | 9 | 343 | 356 | GEPIDLTTFNTDPG | 14 | | 10 | 374 | 375 | NG | 2 | | 11 | 389 | 403 | KNPVQYALLTPLGLD | 15 | | **BepiPred Linear Epitope Prediction 2.0**  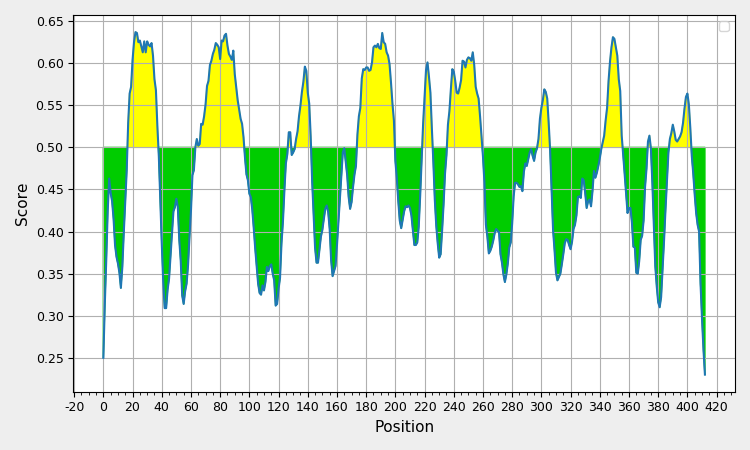 |
| --- | --- | --- | --- | --- | --- | --- | --- | --- | --- | --- | --- | --- | --- | --- | --- | --- | --- | --- | --- | --- | --- | --- | --- | --- | --- | --- | --- | --- | --- | --- | --- | --- | --- | --- | --- | --- | --- | --- | --- | --- | --- | --- | --- | --- | --- | --- | --- | --- | --- | --- | --- | --- | --- | --- | --- | --- | --- | --- | --- | --- | --- |

| **Protein name: 3-carboxymuconate cyclase (Gp60–70)**  **Predicted peptides: Ss551**   | **No.** | **Start** | **End** | **Peptide** | **Length** | | --- | --- | --- | --- | --- | | 1 | 18 | 38 | RTSAHPTSGHYYDDAESEACL | 21 | | 2 | 64 | 97 | SLLLNHATSTATGGRGGNGINPRGMPAGPDALFS | 34 | | 3 | 134 | 143 | VGEPAELPGE | 10 | | 4 | 175 | 200 | WYGLGPFDELRTFDLHQTTPPHGPTN | 26 | | 5 | 220 | 226 | GDPAVNN | 7 | | 6 | 238 | 260 | THSSCYAIPSVSHKGVISSPDGT | 23 | | 7 | 297 | 307 | TLYKTVIPGQD | 11 | | 8 | 342 | 357 | IGEPIDLTTFNTDPGL | 16 | | 9 | 388 | 403 | TKRPVQHGILTPLGLD | 16 | | **BepiPred Linear Epitope Prediction 2.0**  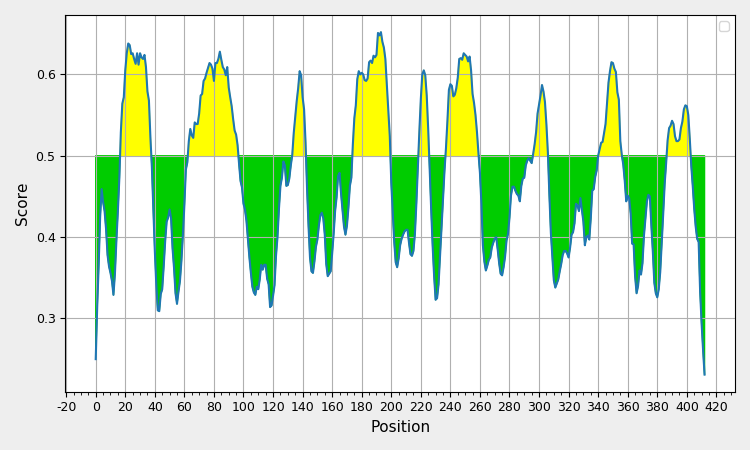 |
| --- | --- | --- | --- | --- | --- | --- | --- | --- | --- | --- | --- | --- | --- | --- | --- | --- | --- | --- | --- | --- | --- | --- | --- | --- | --- | --- | --- | --- | --- | --- | --- | --- | --- | --- | --- | --- | --- | --- | --- | --- | --- | --- | --- | --- | --- | --- | --- | --- | --- | --- | --- |

| **Protein name: 3-carboxymuconate cyclase (Gp60–70)**  **Predicted peptides: Ss13**   | **No.** | **Start** | **End** | **Peptide** | **Length** | | --- | --- | --- | --- | --- | | 1 | 21 | 39 | AHPTSGHYYADAESEACLN | 19 | | 2 | 64 | 98 | SLLLNHATSTATGGRGGNGINPRGMPAGPDALFSQ | 35 | | 3 | 128 | 129 | PT | 2 | | 4 | 133 | 143 | VVGEPAQLPGE | 11 | | 5 | 175 | 199 | WYGLGPFDELRTFDLHQTTPPHGPT | 25 | | 6 | 220 | 227 | GDPAVNNT | 8 | | 7 | 237 | 259 | DIHSSCYAIPSVSHKGFISSPDG | 23 | | 8 | 294 | 294 | E | 1 | | 9 | 297 | 307 | TLYKTVIPGQD | 11 | | 10 | 337 | 356 | ANAEIIGEPIDLTTFNNDPG | 20 | | 11 | 389 | 390 | KK | 2 | | 12 | 397 | 403 | LTPLGLD | 7 | | **BepiPred Linear Epitope Prediction 2.0**  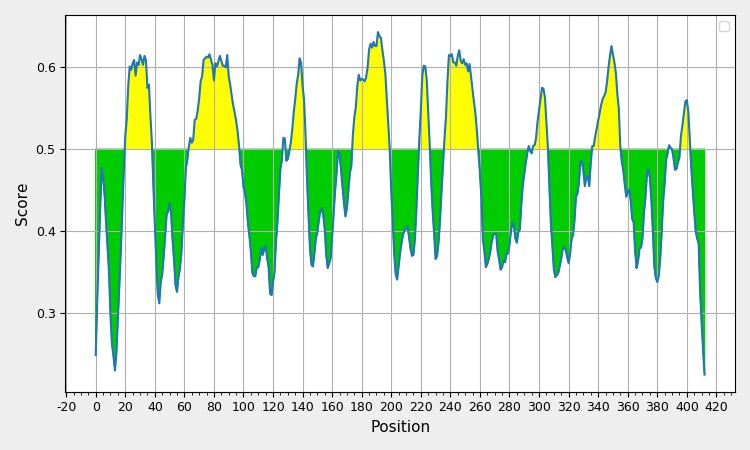 |
| --- | --- | --- | --- | --- | --- | --- | --- | --- | --- | --- | --- | --- | --- | --- | --- | --- | --- | --- | --- | --- | --- | --- | --- | --- | --- | --- | --- | --- | --- | --- | --- | --- | --- | --- | --- | --- | --- | --- | --- | --- | --- | --- | --- | --- | --- | --- | --- | --- | --- | --- | --- | --- | --- | --- | --- | --- | --- | --- | --- | --- | --- | --- | --- | --- | --- | --- |

| **Protein name: 3-carboxymuconate cyclase (Gp60–70)**  **Predicted peptides: Ss124**   | **No.** | **Start** | **End** | **Peptide** | **Length** | | --- | --- | --- | --- | --- | | 1 | 25 | 39 | SGHYYADAESEACLN | 15 | | 2 | 74 | 102 | ATGGRGGNGINPRGMPAGPDALFSQGSIT | 29 | | 3 | 134 | 143 | VGEPAQLPGE | 10 | | 4 | 173 | 199 | YSWYGLGPFDELRTFDLHQTTPPHGPT | 27 | | 5 | 220 | 227 | GDPAVNNT | 8 | | 6 | 237 | 260 | DIHSSCYAIPSVSHKGVISSPDGT | 24 | | 7 | 292 | 307 | YEEASTLYKTVIPGQD | 16 | | 8 | 341 | 357 | IIGEPIDLTTFNNDPGL | 17 | | 9 | 389 | 404 | KKPVQHALLTPLGLDR | 16 | | **BepiPred Linear Epitope Prediction 2.0**  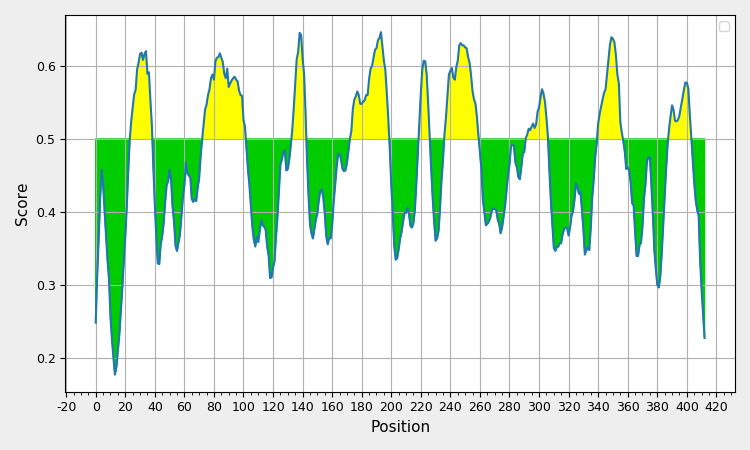 |
| --- | --- | --- | --- | --- | --- | --- | --- | --- | --- | --- | --- | --- | --- | --- | --- | --- | --- | --- | --- | --- | --- | --- | --- | --- | --- | --- | --- | --- | --- | --- | --- | --- | --- | --- | --- | --- | --- | --- | --- | --- | --- | --- | --- | --- | --- | --- | --- | --- | --- | --- | --- |

| **Protein name: 3-carboxymuconate cyclase (Gp60–70)**  **Predicted peptides: Ss137**   | **No.** | **Start** | **End** | **Peptide** | **Length** | | --- | --- | --- | --- | --- | | 1 | 23 | 39 | PTSGHYYADAESEACLN | 17 | | 2 | 70 | 103 | ATSTATGGRGGNGINPRGMPAGPDALFSQGSITI | 34 | | 3 | 126 | 130 | HDPTK | 5 | | 4 | 134 | 143 | VGEPAQLPGE | 10 | | 5 | 173 | 200 | YSWYGLGPFDELRTFDLHQTTPPHGPTN | 28 | | 6 | 220 | 227 | GDPAVNNT | 8 | | 7 | 237 | 261 | DIHSSCYAIPSVSHKGVISSPDGTA | 25 | | 8 | 283 | 283 | G | 1 | | 9 | 292 | 306 | YEEASTLYKTVIPGQ | 15 | | 10 | 341 | 357 | IIGEPIDLTTFNNDPGL | 17 | | 11 | 386 | 390 | ALTKK | 5 | | 12 | 397 | 404 | LTPLGLDR | 8 | | **BepiPred Linear Epitope Prediction 2.0**  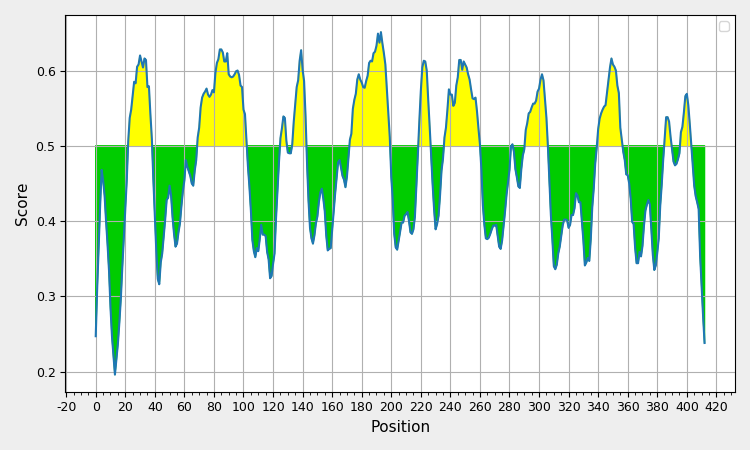 |
| --- | --- | --- | --- | --- | --- | --- | --- | --- | --- | --- | --- | --- | --- | --- | --- | --- | --- | --- | --- | --- | --- | --- | --- | --- | --- | --- | --- | --- | --- | --- | --- | --- | --- | --- | --- | --- | --- | --- | --- | --- | --- | --- | --- | --- | --- | --- | --- | --- | --- | --- | --- | --- | --- | --- | --- | --- | --- | --- | --- | --- | --- | --- | --- | --- | --- | --- |

| **Protein name: 3-carboxymuconate cyclase (Gp60–70)**  **Predicted peptides: Ss46**   | **No.** | **Start** | **End** | **Peptide** | **Length** | | --- | --- | --- | --- | --- | | 1 | 25 | 39 | SGHYYADAESEACLN | 15 | | 2 | 74 | 102 | ATGGRGGNGINPRGMPAGPDALFSQGSIT | 29 | | 3 | 134 | 143 | VGEPAQWPGE | 10 | | 4 | 174 | 199 | SWYGLGPFDELRTFDLHQTTPPHGPT | 26 | | 5 | 220 | 227 | GDPAVNNT | 8 | | 6 | 237 | 260 | DIHSSCYAIPSVSHKGVISSPDGT | 24 | | 7 | 282 | 284 | FGA | 3 | | 8 | 292 | 307 | YEEASTLYKTVIPGQD | 16 | | 9 | 341 | 359 | IIGEPIDLTTFNNDPGLTE | 19 | | 10 | 385 | 391 | NALTKKP | 7 | | 11 | 393 | 404 | QHALLTPLGLDR | 12 | | **BepiPred Linear Epitope Prediction 2.0**  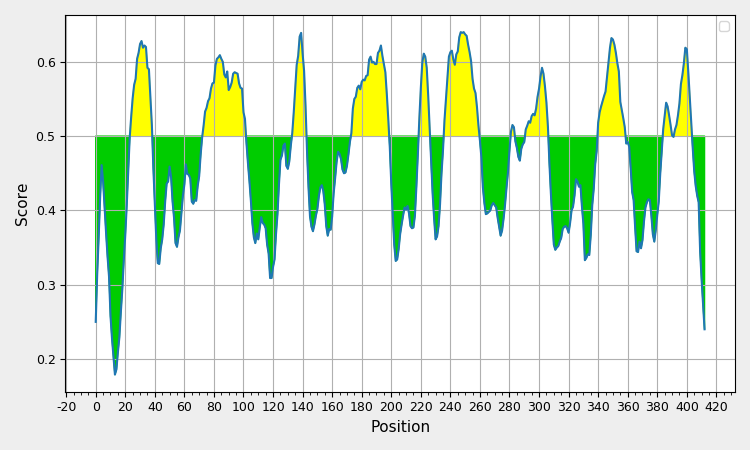 |
| --- | --- | --- | --- | --- | --- | --- | --- | --- | --- | --- | --- | --- | --- | --- | --- | --- | --- | --- | --- | --- | --- | --- | --- | --- | --- | --- | --- | --- | --- | --- | --- | --- | --- | --- | --- | --- | --- | --- | --- | --- | --- | --- | --- | --- | --- | --- | --- | --- | --- | --- | --- | --- | --- | --- | --- | --- | --- | --- | --- | --- | --- |

| **Protein name: 3-carboxymuconate cyclase (Gp60–70)**  **Predicted peptides: Ss17**   | **No.** | **Start** | **End** | **Peptide** | **Length** | | --- | --- | --- | --- | --- | | 1 | 22 | 39 | HPTSGHYYADAESEACLN | 18 | | 2 | 64 | 97 | SLLLNHATSTATGGRGGNGINPRGMPAGPDALFS | 34 | | 3 | 133 | 143 | VVGEPAQLPGE | 11 | | 4 | 175 | 200 | WYGLGPFDELRTFDLHQTTPPHGPTN | 26 | | 5 | 220 | 226 | GDPAVNN | 7 | | 6 | 237 | 262 | DIHSSCYSIPSVSHKGVISSPDGTAV | 26 | | 7 | 299 | 307 | YKTVIPGQD | 9 | | 8 | 339 | 357 | AEIIGEPIDLTTFNNDPGL | 19 | | 9 | 374 | 376 | NGT | 3 | | 10 | 389 | 404 | KKPVQHALLTPLGLDR | 16 | | **BepiPred Linear Epitope Prediction 2.0**  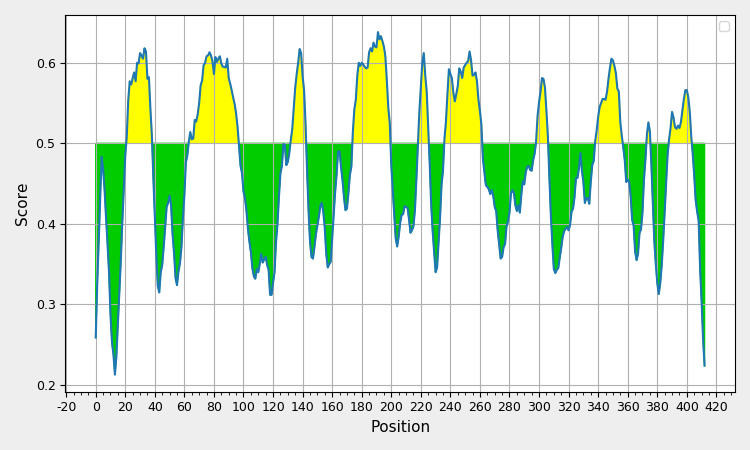 |
| --- | --- | --- | --- | --- | --- | --- | --- | --- | --- | --- | --- | --- | --- | --- | --- | --- | --- | --- | --- | --- | --- | --- | --- | --- | --- | --- | --- | --- | --- | --- | --- | --- | --- | --- | --- | --- | --- | --- | --- | --- | --- | --- | --- | --- | --- | --- | --- | --- | --- | --- | --- | --- | --- | --- | --- | --- |

| **Protein name: 3-carboxymuconate cyclase (Gp60–70)**  **Predicted peptides: SsMS1**   | **No.** | **Start** | **End** | **Peptide** | **Length** | | --- | --- | --- | --- | --- | | 1 | 23 | 39 | PTSGHYYADAESEVCLN | 17 | | 2 | 65 | 98 | LLLNHATSTATGGRGGNGINPRGMPAGPDALFSQ | 34 | | 3 | 128 | 129 | PT | 2 | | 4 | 132 | 143 | TVVGEPAQLPGE | 12 | | 5 | 176 | 200 | YGLGPFDELRTFDLHQTTPPHGPTN | 25 | | 6 | 220 | 226 | GDPAVNN | 7 | | 7 | 237 | 261 | DIHSSCYSIPSVSHKGVISSPDGTA | 25 | | 8 | 299 | 307 | YKTVIPGQD | 9 | | 9 | 339 | 357 | AEIIGEPIDLTTFNNDPGL | 19 | | 10 | 388 | 393 | TKKPVQ | 6 | | 11 | 395 | 403 | ALLTPLGLD | 9 | | **BepiPred Linear Epitope Prediction 2.0**  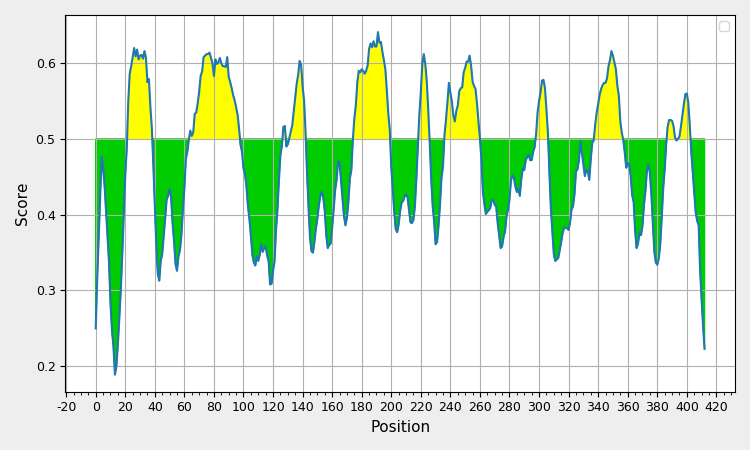 |
| --- | --- | --- | --- | --- | --- | --- | --- | --- | --- | --- | --- | --- | --- | --- | --- | --- | --- | --- | --- | --- | --- | --- | --- | --- | --- | --- | --- | --- | --- | --- | --- | --- | --- | --- | --- | --- | --- | --- | --- | --- | --- | --- | --- | --- | --- | --- | --- | --- | --- | --- | --- | --- | --- | --- | --- | --- | --- | --- | --- | --- | --- |

| **Protein name: 3-carboxymuconate cyclase (Gp60–70)**  **Predicted peptides: SsEM7**   | **No.** | **Start** | **End** | **Peptide** | **Length** | | --- | --- | --- | --- | --- | | 1 | 22 | 39 | HPTSGHYYADAESEACLN | 18 | | 2 | 64 | 97 | SLLLNHATSTATGGRGGNGINPRGMPAGPDALFS | 34 | | 3 | 133 | 143 | VVGEPAQLPGE | 11 | | 4 | 175 | 200 | WYGLGPFDELRTFDLHQTTPPHGPTN | 26 | | 5 | 220 | 226 | GDPAVNN | 7 | | 6 | 237 | 262 | DIHSSCYSIPSVSHKGVISSPDGTAV | 26 | | 7 | 299 | 307 | YKTVIPGQD | 9 | | 8 | 339 | 357 | AEIIGEPIDLTTFNNDPGL | 19 | | 9 | 374 | 376 | NGT | 3 | | 10 | 389 | 404 | KKPVQHALLTPLGLDR | 16 | | **BepiPred Linear Epitope Prediction 2.0**  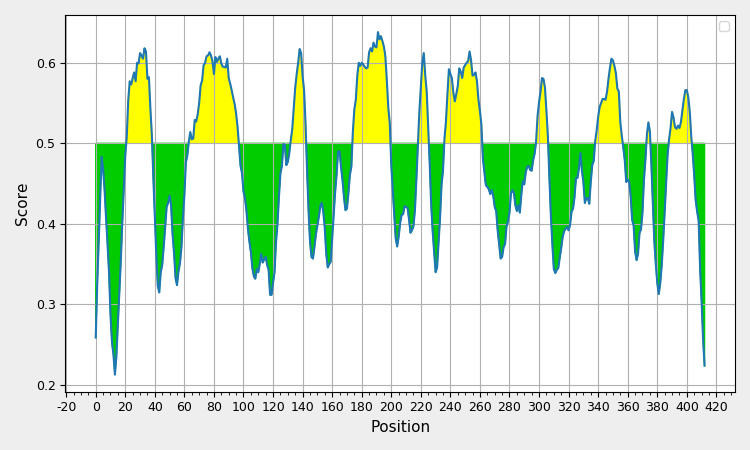 |
| --- | --- | --- | --- | --- | --- | --- | --- | --- | --- | --- | --- | --- | --- | --- | --- | --- | --- | --- | --- | --- | --- | --- | --- | --- | --- | --- | --- | --- | --- | --- | --- | --- | --- | --- | --- | --- | --- | --- | --- | --- | --- | --- | --- | --- | --- | --- | --- | --- | --- | --- | --- | --- | --- | --- | --- | --- |

| **Protein name: 3-carboxymuconate cyclase (Gp60–70)**  **Predicted peptides: Ss126**   | **No.** | **Start** | **End** | **Peptide** | **Length** | | --- | --- | --- | --- | --- | | 1 | 20 | 39 | SAHPTSGHYYADAESEACLN | 20 | | 2 | 73 | 102 | TATGGRGGNGINPRGMPAGPDALFSQGSIT | 30 | | 3 | 134 | 143 | VGEPAQLPGE | 10 | | 4 | 175 | 199 | WYGLGPFDELRTFDLHQTTPPHGPT | 25 | | 5 | 220 | 227 | GDPAVNNT | 8 | | 6 | 237 | 259 | DIHSSCYSIPSVSHKGVISSPDG | 23 | | 7 | 282 | 284 | FGA | 3 | | 8 | 292 | 307 | YEEASTLYKTVIPGQD | 16 | | 9 | 341 | 356 | IIGEPIDLTTFNNDPG | 16 | | 10 | 389 | 403 | KKPVQHALLTPLGLD | 15 | | **BepiPred Linear Epitope Prediction 2.0**  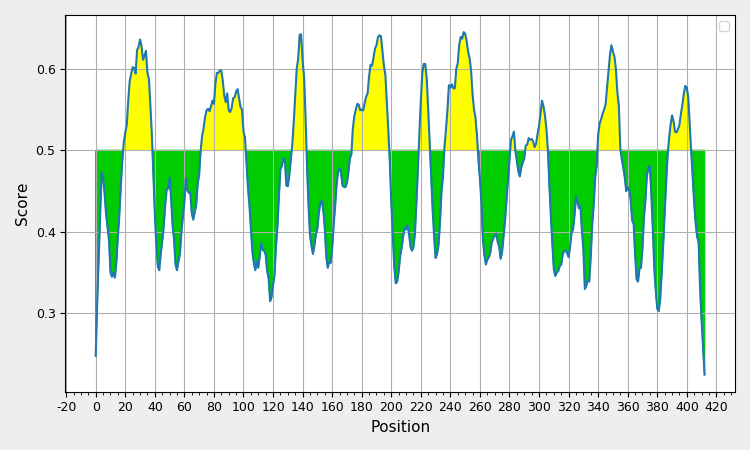 |
| --- | --- | --- | --- | --- | --- | --- | --- | --- | --- | --- | --- | --- | --- | --- | --- | --- | --- | --- | --- | --- | --- | --- | --- | --- | --- | --- | --- | --- | --- | --- | --- | --- | --- | --- | --- | --- | --- | --- | --- | --- | --- | --- | --- | --- | --- | --- | --- | --- | --- | --- | --- | --- | --- | --- | --- | --- |

| **Protein name: 3-carboxymuconate cyclase (Gp60–70)**  **Predicted peptides: Ss241**   | **No.** | **Start** | **End** | **Peptide** | **Length** | | --- | --- | --- | --- | --- | | 1 | 22 | 39 | HPTSGHYYADAESEACLN | 18 | | 2 | 64 | 97 | SLLLNHATSTATGGRGGNGINPRGMPAGPDALFS | 34 | | 3 | 133 | 143 | VVGEPAQLPGE | 11 | | 4 | 175 | 200 | WYGLGPFDELRTFDLHQTTPPHGPTN | 26 | | 5 | 220 | 226 | GDPAVNN | 7 | | 6 | 237 | 262 | DIHSSCYSIPSVSHKGVISSPDGTAV | 26 | | 7 | 299 | 307 | YKTVIPGQD | 9 | | 8 | 339 | 357 | AEIIGEPIDLTTFNNDPGL | 19 | | 9 | 374 | 376 | NGT | 3 | | 10 | 389 | 404 | KKPVQHALLTPLGLDR | 16 | | **BepiPred Linear Epitope Prediction 2.0**  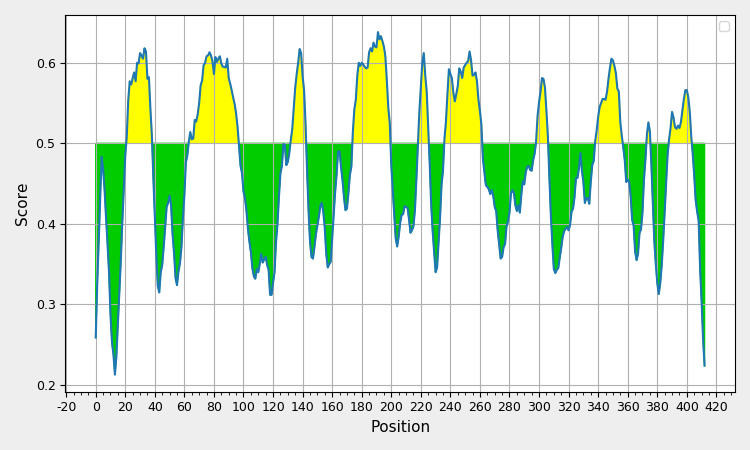 |
| --- | --- | --- | --- | --- | --- | --- | --- | --- | --- | --- | --- | --- | --- | --- | --- | --- | --- | --- | --- | --- | --- | --- | --- | --- | --- | --- | --- | --- | --- | --- | --- | --- | --- | --- | --- | --- | --- | --- | --- | --- | --- | --- | --- | --- | --- | --- | --- | --- | --- | --- | --- | --- | --- | --- | --- | --- |

| **Protein name: 3-carboxymuconate cyclase (Gp60–70)**  **Predicted peptides: Ss40**   | **No.** | **Start** | **End** | **Peptide** | **Length** | | --- | --- | --- | --- | --- | | 1 | 23 | 38 | PTGGHYYADAESEACL | 16 | | 2 | 64 | 97 | SLLLNHATSTATGGRGGNGINPRGMPAGPDALFS | 34 | | 3 | 134 | 143 | VGEPAQLPGE | 10 | | 4 | 175 | 200 | WYGLGPFDELRTFDLHQTTPPHGPTN | 26 | | 5 | 220 | 226 | GDPAVNN | 7 | | 6 | 237 | 262 | DIHSSCYSIPSVSHKGVISSPDGTAV | 26 | | 7 | 299 | 307 | YKTVIPGQD | 9 | | 8 | 339 | 357 | AEIIGEPIDLTTFNNDPGL | 19 | | 9 | 374 | 376 | NGT | 3 | | 10 | 390 | 404 | KPVQHALLTPLGLDR | 15 | | **BepiPred Linear Epitope Prediction 2.0**  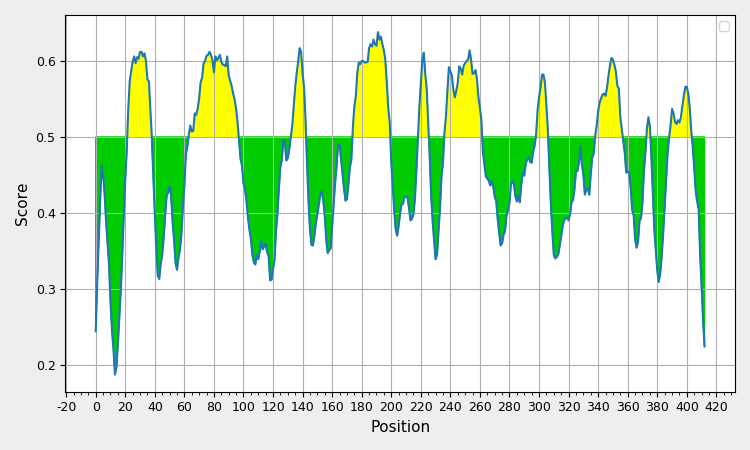 |
| --- | --- | --- | --- | --- | --- | --- | --- | --- | --- | --- | --- | --- | --- | --- | --- | --- | --- | --- | --- | --- | --- | --- | --- | --- | --- | --- | --- | --- | --- | --- | --- | --- | --- | --- | --- | --- | --- | --- | --- | --- | --- | --- | --- | --- | --- | --- | --- | --- | --- | --- | --- | --- | --- | --- | --- | --- |

| **Protein name: 3-carboxymuconate cyclase (Gp60–70)**  **Predicted peptides: Ss243**   | **No.** | **Start** | **End** | **Peptide** | **Length** | | --- | --- | --- | --- | --- | | 1 | 23 | 38 | PTGGHYYADAESEACL | 16 | | 2 | 64 | 97 | SLLLNHATSTATGGRGGNGINPRGMPAGPDALFS | 34 | | 3 | 134 | 143 | VGEPAQLPGE | 10 | | 4 | 175 | 200 | WYGLGPFDELRTFDLHQTTPPHGPTN | 26 | | 5 | 220 | 226 | GDPAVNN | 7 | | 6 | 237 | 262 | DIHSSCYSIPSVSHKGVISSPDGTAV | 26 | | 7 | 299 | 307 | YKTVIPGQD | 9 | | 8 | 339 | 357 | AEIIGEPIDLTTFNNDPGL | 19 | | 9 | 374 | 376 | NGT | 3 | | 10 | 390 | 404 | KPVQHALLTPLGLDR | 15 | | **BepiPred Linear Epitope Prediction 2.0**  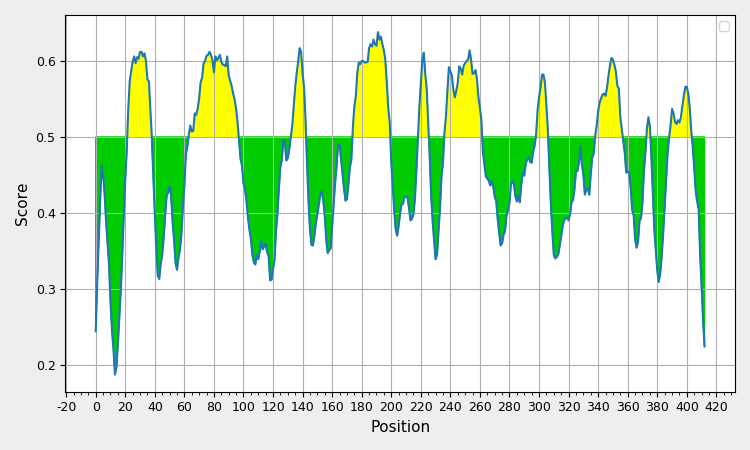 |
| --- | --- | --- | --- | --- | --- | --- | --- | --- | --- | --- | --- | --- | --- | --- | --- | --- | --- | --- | --- | --- | --- | --- | --- | --- | --- | --- | --- | --- | --- | --- | --- | --- | --- | --- | --- | --- | --- | --- | --- | --- | --- | --- | --- | --- | --- | --- | --- | --- | --- | --- | --- | --- | --- | --- | --- | --- |

| **Protein name: 3-carboxymuconate cyclase (Gp60–70)**  **Predicted peptides: Ss15**   | **No.** | **Start** | **End** | **Peptide** | **Length** | | --- | --- | --- | --- | --- | | 1 | 26 | 39 | GHYYADAESEACLN | 14 | | 2 | 65 | 98 | LLLNHATSTATGGRGGNGINPRGMPAGPDALFSQ | 34 | | 3 | 128 | 129 | PT | 2 | | 4 | 133 | 143 | VVGEPAQLPGE | 11 | | 5 | 175 | 200 | WYGLGPFDELRTFDLHQTTPPHGPTN | 26 | | 6 | 220 | 226 | GDPAVNN | 7 | | 7 | 237 | 261 | DIHSSCYSIPSVSHKGVISSPDGTA | 25 | | 8 | 299 | 307 | YKTVIPGQD | 9 | | 9 | 339 | 357 | AEIIGEPIDLTTFNNDPGL | 19 | | 10 | 387 | 403 | LTKKPVQHALLTPLGLD | 17 | | **BepiPred Linear Epitope Prediction 2.0**  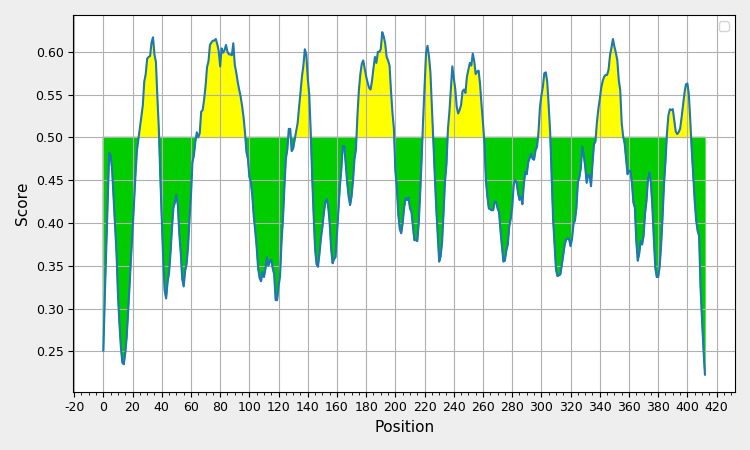 |
| --- | --- | --- | --- | --- | --- | --- | --- | --- | --- | --- | --- | --- | --- | --- | --- | --- | --- | --- | --- | --- | --- | --- | --- | --- | --- | --- | --- | --- | --- | --- | --- | --- | --- | --- | --- | --- | --- | --- | --- | --- | --- | --- | --- | --- | --- | --- | --- | --- | --- | --- | --- | --- | --- | --- | --- | --- |

| **Protein name: 3-carboxymuconate cyclase (Gp60–70)**  **Predicted peptides: Ss205**   | **No.** | **Start** | **End** | **Peptide** | **Length** | | --- | --- | --- | --- | --- | | 1 | 26 | 39 | GHYYADAESEACLN | 14 | | 2 | 74 | 102 | ATGGRGGNGINPRGMPAGPDALFSQGSIT | 29 | | 3 | 134 | 143 | VGEPAQLPGE | 10 | | 4 | 173 | 199 | YSWYGLGPFDELRTFDLHQTTPPHGPT | 27 | | 5 | 220 | 227 | GDPAVNNT | 8 | | 6 | 237 | 260 | DIHSSCYSIPSVSHKGVISSPDGT | 24 | | 7 | 293 | 307 | EEASTLYKTVIPGQD | 15 | | 8 | 341 | 358 | IIGEPIDLTTFNNDPGLT | 18 | | 9 | 389 | 403 | KKPVQHALLTPLGLD | 15 | | **BepiPred Linear Epitope Prediction 2.0**  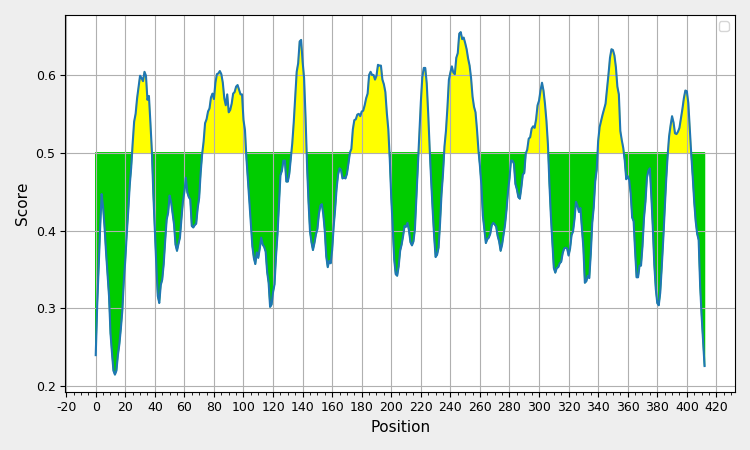 |
| --- | --- | --- | --- | --- | --- | --- | --- | --- | --- | --- | --- | --- | --- | --- | --- | --- | --- | --- | --- | --- | --- | --- | --- | --- | --- | --- | --- | --- | --- | --- | --- | --- | --- | --- | --- | --- | --- | --- | --- | --- | --- | --- | --- | --- | --- | --- | --- | --- | --- | --- | --- |

| **Protein name: 3-carboxymuconate cyclase (Gp60–70)**  **Predicted peptides: Ss22**   | **No.** | **Start** | **End** | **Peptide** | **Length** | | --- | --- | --- | --- | --- | | 1 | 26 | 39 | GHYYADAESEACLN | 14 | | 2 | 65 | 98 | LLLNHATSTATGGRGGNGINPRGMPAGPDALFSQ | 34 | | 3 | 128 | 129 | PT | 2 | | 4 | 133 | 143 | VVGEPAQLPGE | 11 | | 5 | 175 | 200 | WYGLGPFDELRTFDLHQTTPPHGPTN | 26 | | 6 | 220 | 226 | GDPAVNN | 7 | | 7 | 237 | 261 | DIHSSCYSIPSVSHKGVISSPDGTA | 25 | | 8 | 299 | 307 | YKTVIPGQD | 9 | | 9 | 339 | 357 | AEIIGEPIDLTTFNNDPGL | 19 | | 10 | 387 | 403 | LTKKPVQHALLTPLGLD | 17 | | **BepiPred Linear Epitope Prediction 2.0**  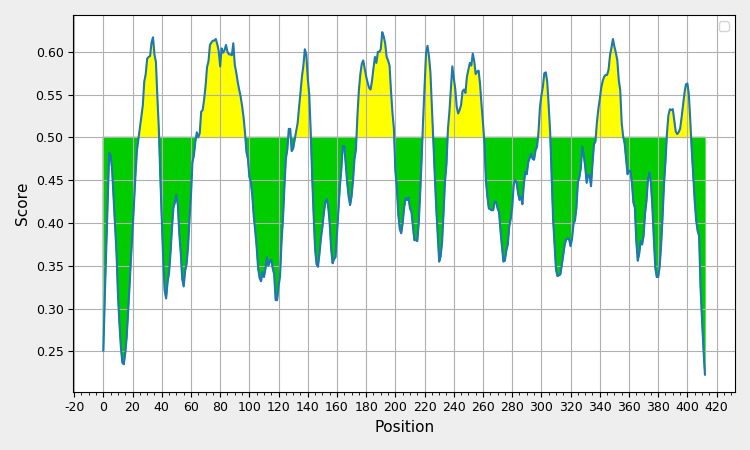 |
| --- | --- | --- | --- | --- | --- | --- | --- | --- | --- | --- | --- | --- | --- | --- | --- | --- | --- | --- | --- | --- | --- | --- | --- | --- | --- | --- | --- | --- | --- | --- | --- | --- | --- | --- | --- | --- | --- | --- | --- | --- | --- | --- | --- | --- | --- | --- | --- | --- | --- | --- | --- | --- | --- | --- | --- | --- |

| **Protein name: 3-carboxymuconate cyclase (Gp60–70)**  **Predicted peptides: Ss61**   | **No.** | **Start** | **End** | **Peptide** | **Length** | | --- | --- | --- | --- | --- | | 1 | 29 | 39 | YADAESEACLN | 11 | | 2 | 62 | 96 | NGSLLLNHATSTATGGRGGNGINPRGMPAGPDALF | 35 | | 3 | 128 | 129 | PT | 2 | | 4 | 134 | 143 | VGEPAQLPGE | 10 | | 5 | 176 | 199 | YGLGPFDELRTFDLHQTTPPHGPT | 24 | | 6 | 220 | 226 | GDPAVNN | 7 | | 7 | 237 | 260 | DIHSSCYSIPSVSHKGVISSPDGT | 24 | | 8 | 299 | 307 | YKTVIPGQD | 9 | | 9 | 342 | 357 | IGEPIDLTTFNNDPGL | 16 | | 10 | 388 | 392 | TKKPV | 5 | | 11 | 396 | 403 | LLTPLGLD | 8 | | **BepiPred Linear Epitope Prediction 2.0**  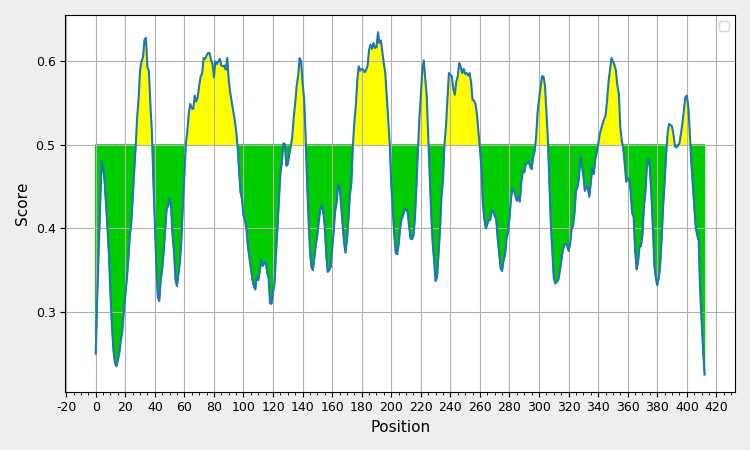 |
| --- | --- | --- | --- | --- | --- | --- | --- | --- | --- | --- | --- | --- | --- | --- | --- | --- | --- | --- | --- | --- | --- | --- | --- | --- | --- | --- | --- | --- | --- | --- | --- | --- | --- | --- | --- | --- | --- | --- | --- | --- | --- | --- | --- | --- | --- | --- | --- | --- | --- | --- | --- | --- | --- | --- | --- | --- | --- | --- | --- | --- | --- |

| **Protein name: 3-carboxymuconate cyclase (Gp60–70)**  **Predicted peptides: Ss468**   | **No.** | **Start** | **End** | **Peptide** | **Length** | | --- | --- | --- | --- | --- | | 1 | 24 | 38 | TSDHYYADAESEACL | 15 | | 2 | 62 | 97 | NGSLLTDHATSTATGGRGGNGINPRGMPAGPDALFG | 36 | | 3 | 128 | 129 | PT | 2 | | 4 | 133 | 143 | VVGEPAELPGE | 11 | | 5 | 175 | 200 | WYGLGPFDELRPFDLHQTTPPHGPTN | 26 | | 6 | 220 | 225 | GDPAVN | 6 | | 7 | 237 | 258 | HIHSSCYATPSVSHKGVISSPD | 22 | | 8 | 293 | 294 | EE | 2 | | 9 | 297 | 307 | TLYKTVIPGQD | 11 | | 10 | 340 | 362 | EIIGEPIDLTAFNNDPGLTEIRS | 23 | | 11 | 386 | 403 | ALTKKPVQHALLTPLGLD | 18 | | **BepiPred Linear Epitope Prediction 2.0**  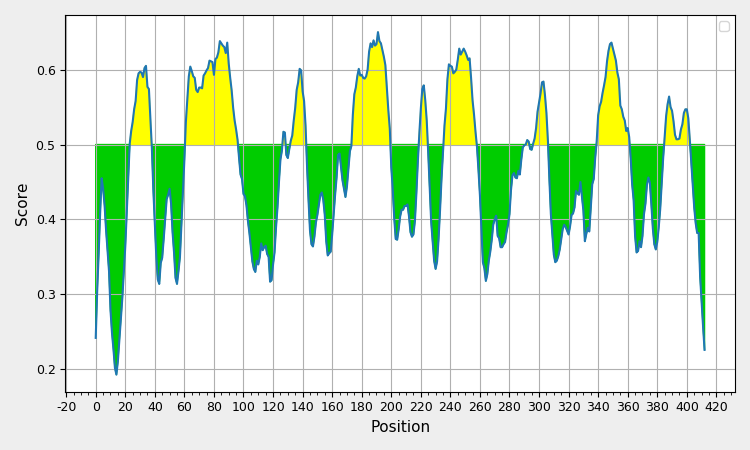 |
| --- | --- | --- | --- | --- | --- | --- | --- | --- | --- | --- | --- | --- | --- | --- | --- | --- | --- | --- | --- | --- | --- | --- | --- | --- | --- | --- | --- | --- | --- | --- | --- | --- | --- | --- | --- | --- | --- | --- | --- | --- | --- | --- | --- | --- | --- | --- | --- | --- | --- | --- | --- | --- | --- | --- | --- | --- | --- | --- | --- | --- | --- |

| **Protein name: 3-carboxymuconate cyclase (Gp60–70)**  **Predicted peptides: Ss51**   | **No.** | **Start** | **End** | **Peptide** | **Length** | | --- | --- | --- | --- | --- | | 1 | 24 | 38 | TSDHYYADAESEACL | 15 | | 2 | 62 | 97 | NGSLLTDHATSTATGGRGGNGINPRGMPAGPDALFG | 36 | | 3 | 128 | 129 | PT | 2 | | 4 | 133 | 143 | VVGEPAELPGE | 11 | | 5 | 175 | 200 | WYGLGPFDELRPFDLHQTTPPHGPTN | 26 | | 6 | 220 | 225 | GDPAVN | 6 | | 7 | 237 | 258 | HIHSSCYATPSVSHKGVISSPD | 22 | | 8 | 293 | 294 | EE | 2 | | 9 | 298 | 307 | LYKTVIPGQD | 10 | | 10 | 340 | 362 | EIIGEPIDLTAFNNDPGLTEIRS | 23 | | 11 | 386 | 403 | ALTKKPVQHALLTPLGLD | 18 | | **BepiPred Linear Epitope Prediction 2.0**  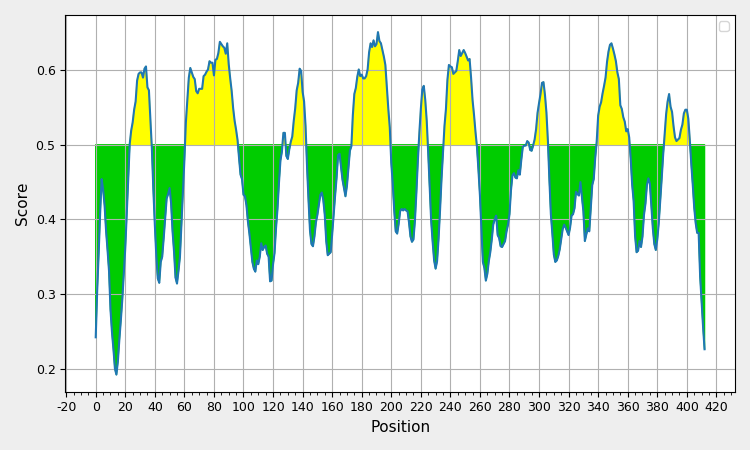 |
| --- | --- | --- | --- | --- | --- | --- | --- | --- | --- | --- | --- | --- | --- | --- | --- | --- | --- | --- | --- | --- | --- | --- | --- | --- | --- | --- | --- | --- | --- | --- | --- | --- | --- | --- | --- | --- | --- | --- | --- | --- | --- | --- | --- | --- | --- | --- | --- | --- | --- | --- | --- | --- | --- | --- | --- | --- | --- | --- | --- | --- | --- |

| **Protein name: 3-carboxymuconate cyclase (Gp60–70)**  **Predicted peptides: Ss467**   | **No.** | **Start** | **End** | **Peptide** | **Length** | | --- | --- | --- | --- | --- | | 1 | 22 | 38 | HPTNDRYYADADSEACL | 17 | | 2 | 62 | 97 | NGSLLVNHATSTATGGSGGNGINPRGMPAGPDALFG | 36 | | 3 | 127 | 129 | DPT | 3 | | 4 | 133 | 143 | VVGEPAVLPGE | 11 | | 5 | 175 | 200 | WYGLGPFDELRPFDLNQTTPPHGPTN | 26 | | 6 | 220 | 226 | GDPTVNN | 7 | | 7 | 238 | 260 | IHTSCYATPSVSYKGVMSSPNGT | 23 | | 8 | 299 | 306 | YKTVIPGQ | 8 | | 9 | 341 | 359 | IIGEPIDLTTFNNDPGLTE | 19 | | 10 | 374 | 376 | NGT | 3 | | 11 | 386 | 404 | ALTKNPVQLALLTPLGLDR | 19 | | **BepiPred Linear Epitope Prediction 2.0**  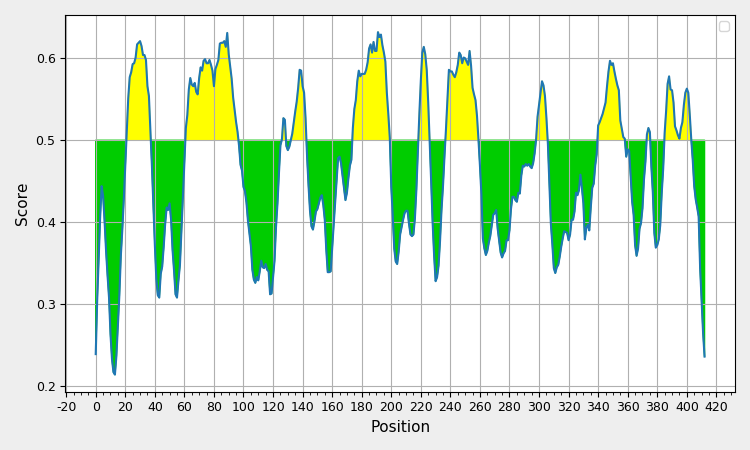 |
| --- | --- | --- | --- | --- | --- | --- | --- | --- | --- | --- | --- | --- | --- | --- | --- | --- | --- | --- | --- | --- | --- | --- | --- | --- | --- | --- | --- | --- | --- | --- | --- | --- | --- | --- | --- | --- | --- | --- | --- | --- | --- | --- | --- | --- | --- | --- | --- | --- | --- | --- | --- | --- | --- | --- | --- | --- | --- | --- | --- | --- | --- |

| **Protein name: 3-carboxymuconate cyclase (Gp60–70)**  **Predicted peptides: Ss449**   | **No.** | **Start** | **End** | **Peptide** | **Length** | | --- | --- | --- | --- | --- | | 1 | 22 | 38 | HPTNDRYYADADSEACL | 17 | | 2 | 63 | 97 | GSLLVNHATSTATGGSGGNGINPRGMPAGPDALFG | 35 | | 3 | 126 | 130 | HDPTK | 5 | | 4 | 132 | 143 | TVVGEPAVLPGE | 12 | | 5 | 175 | 200 | WYGLGPFDELRPFDLNQTTPPHGPTN | 26 | | 6 | 220 | 226 | GDPTVNN | 7 | | 7 | 238 | 260 | IHTSCYATPSVSYKGVMSSPNGT | 23 | | 8 | 300 | 306 | KTVIPGQ | 7 | | 9 | 339 | 356 | AEIIGEPIDLTTFNNDPG | 18 | | 10 | 375 | 375 | G | 1 | | 11 | 388 | 403 | TKKPVQHALLTPLGLD | 16 | | **BepiPred Linear Epitope Prediction 2.0**  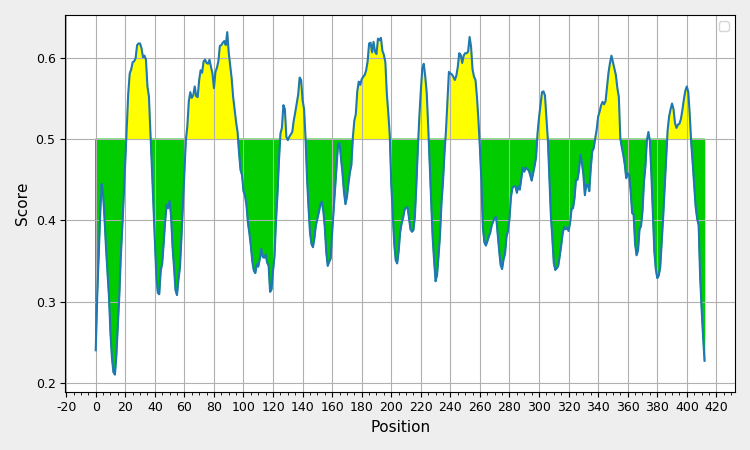 |
| --- | --- | --- | --- | --- | --- | --- | --- | --- | --- | --- | --- | --- | --- | --- | --- | --- | --- | --- | --- | --- | --- | --- | --- | --- | --- | --- | --- | --- | --- | --- | --- | --- | --- | --- | --- | --- | --- | --- | --- | --- | --- | --- | --- | --- | --- | --- | --- | --- | --- | --- | --- | --- | --- | --- | --- | --- | --- | --- | --- | --- | --- |

| **Protein name: 3-carboxymuconate cyclase (Gp60–70)**  **Predicted peptides: Ss445**   | **No.** | **Start** | **End** | **Peptide** | **Length** | | --- | --- | --- | --- | --- | | 1 | 22 | 38 | HPTNDRYYADADSEACL | 17 | | 2 | 63 | 97 | GSLLVNHATSTATGGSGGNGINPRGMPAGPDALFG | 35 | | 3 | 126 | 130 | HDPTK | 5 | | 4 | 132 | 143 | TVVGEPAVLPGE | 12 | | 5 | 175 | 200 | WYGLGPFDELRPFDLNQTTPPHGPTN | 26 | | 6 | 220 | 226 | GDPTVNN | 7 | | 7 | 238 | 260 | IHTSCYATPSVSYKGVMSSPNGT | 23 | | 8 | 300 | 306 | KTVIPGQ | 7 | | 9 | 339 | 356 | AEIIGEPIDLTTFNNDPG | 18 | | 10 | 375 | 375 | G | 1 | | 11 | 388 | 403 | TKKPVQHALLTPLGLD | 16 | | **BepiPred Linear Epitope Prediction 2.0**  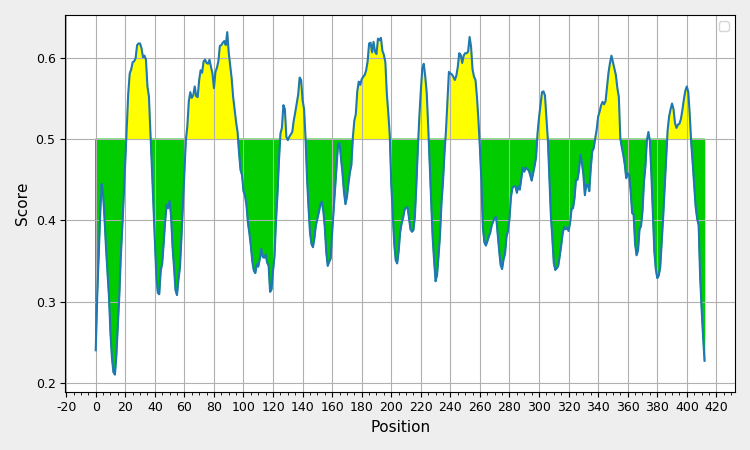 |
| --- | --- | --- | --- | --- | --- | --- | --- | --- | --- | --- | --- | --- | --- | --- | --- | --- | --- | --- | --- | --- | --- | --- | --- | --- | --- | --- | --- | --- | --- | --- | --- | --- | --- | --- | --- | --- | --- | --- | --- | --- | --- | --- | --- | --- | --- | --- | --- | --- | --- | --- | --- | --- | --- | --- | --- | --- | --- | --- | --- | --- | --- |

| **Protein name: 3-carboxymuconate cyclase (Gp60–70)**  **Predicted peptides: Ss456**   | **No.** | **Start** | **End** | **Peptide** | **Length** | | --- | --- | --- | --- | --- | | 1 | 22 | 38 | HPTNDRYYADADSEACL | 17 | | 2 | 63 | 97 | GSLLVNHATSTATGGSGGNGINPRGMPAGPDALFG | 35 | | 3 | 126 | 130 | HDPTK | 5 | | 4 | 132 | 143 | TVVGEPAVLPGE | 12 | | 5 | 175 | 200 | WYGLGPFDELRPFDLNQTTPPHGPTN | 26 | | 6 | 220 | 226 | GDPTVNN | 7 | | 7 | 238 | 260 | IHTSCYATPSVSYKGVMSSPNGT | 23 | | 8 | 300 | 306 | KTVIPGQ | 7 | | 9 | 339 | 356 | AEIIGEPIDLTTFNNDPG | 18 | | 10 | 375 | 375 | G | 1 | | 11 | 388 | 403 | TKKPVQHALLTPLGLD | 16 | | **BepiPred Linear Epitope Prediction 2.0**  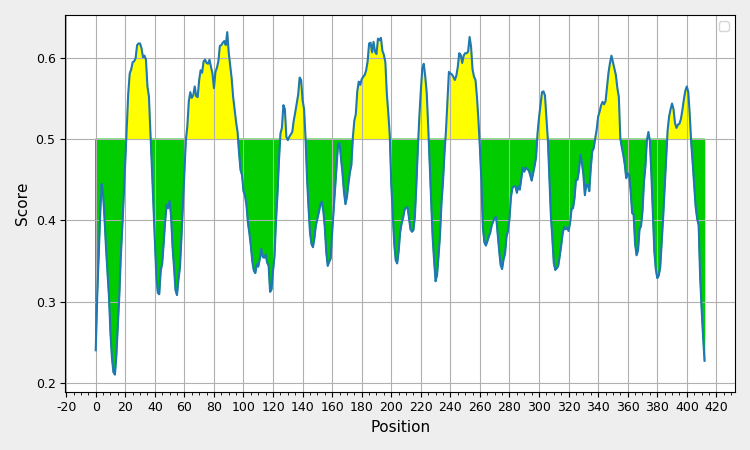 |
| --- | --- | --- | --- | --- | --- | --- | --- | --- | --- | --- | --- | --- | --- | --- | --- | --- | --- | --- | --- | --- | --- | --- | --- | --- | --- | --- | --- | --- | --- | --- | --- | --- | --- | --- | --- | --- | --- | --- | --- | --- | --- | --- | --- | --- | --- | --- | --- | --- | --- | --- | --- | --- | --- | --- | --- | --- | --- | --- | --- | --- | --- |

| **Protein name: 3-carboxymuconate cyclase (Gp60–70)**  **Predicted peptides: Ss457**   | **No.** | **Start** | **End** | **Peptide** | **Length** | | --- | --- | --- | --- | --- | | 1 | 22 | 38 | HPTNDRYYADADSEACL | 17 | | 2 | 63 | 97 | GSLLVNHATSTATGGSGGNGINPRGMPAGPDALFG | 35 | | 3 | 126 | 130 | HDPTK | 5 | | 4 | 132 | 143 | TVVGEPAVLPGE | 12 | | 5 | 175 | 200 | WYGLGPFDELRPFDLNQTTPPHGPTN | 26 | | 6 | 220 | 226 | GDPTVNN | 7 | | 7 | 238 | 260 | IHTSCYATPSVSYKGVMSSPNGT | 23 | | 8 | 300 | 306 | KTVIPGQ | 7 | | 9 | 339 | 356 | AEIIGEPIDLTTFNNDPG | 18 | | 10 | 375 | 375 | G | 1 | | 11 | 388 | 403 | TKKPVQHALLTPLGLD | 16 | | **BepiPred Linear Epitope Prediction 2.0**  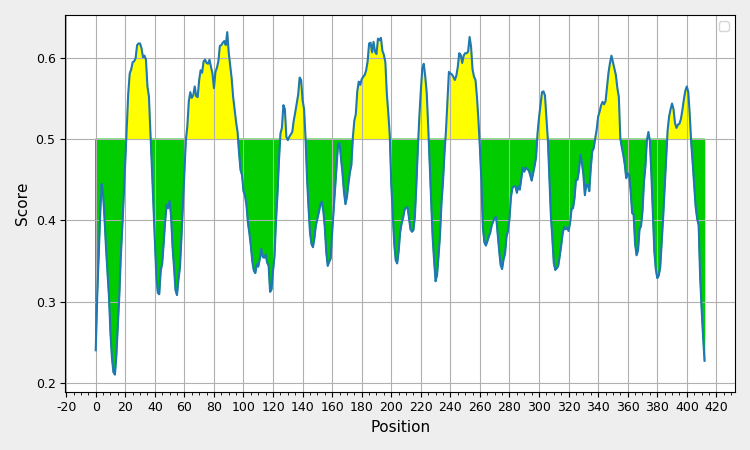 |
| --- | --- | --- | --- | --- | --- | --- | --- | --- | --- | --- | --- | --- | --- | --- | --- | --- | --- | --- | --- | --- | --- | --- | --- | --- | --- | --- | --- | --- | --- | --- | --- | --- | --- | --- | --- | --- | --- | --- | --- | --- | --- | --- | --- | --- | --- | --- | --- | --- | --- | --- | --- | --- | --- | --- | --- | --- | --- | --- | --- | --- | --- |

| **Protein name: 3-carboxymuconate cyclase (Gp60–70)**  **Predicted peptides: Ss443**   | **No.** | **Start** | **End** | **Peptide** | **Length** | | --- | --- | --- | --- | --- | | 1 | 22 | 38 | HPTNDRYYADADSEACL | 17 | | 2 | 63 | 97 | GSLLVNHATSTATGGSGGNGINPRGMPAGPDALFG | 35 | | 3 | 126 | 130 | HDPTK | 5 | | 4 | 132 | 143 | TVVGEPAVLPGE | 12 | | 5 | 175 | 200 | WYGLGPFDELRPFDLNQTTPPHGPTN | 26 | | 6 | 220 | 226 | GDPTVNN | 7 | | 7 | 238 | 260 | IHTSCYATPSVSYKGVMSSPNGT | 23 | | 8 | 300 | 306 | KTVIPGQ | 7 | | 9 | 339 | 356 | AEIIGEPIDLTTFNNDPG | 18 | | 10 | 375 | 375 | G | 1 | | 11 | 388 | 403 | TKKPVQHALLTPLGLD | 16 | | **BepiPred Linear Epitope Prediction 2.0**  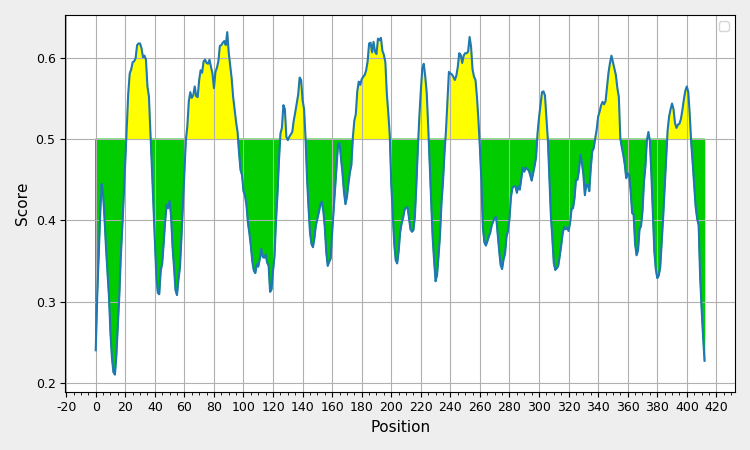 |
| --- | --- | --- | --- | --- | --- | --- | --- | --- | --- | --- | --- | --- | --- | --- | --- | --- | --- | --- | --- | --- | --- | --- | --- | --- | --- | --- | --- | --- | --- | --- | --- | --- | --- | --- | --- | --- | --- | --- | --- | --- | --- | --- | --- | --- | --- | --- | --- | --- | --- | --- | --- | --- | --- | --- | --- | --- | --- | --- | --- | --- | --- |

| **Protein name: 3-carboxymuconate cyclase (Gp60–70)**  **Predicted peptides: Ss06**   | **No.** | **Start** | **End** | **Peptide** | **Length** | | --- | --- | --- | --- | --- | | 1 | 22 | 38 | HPTNDRYYADADSEACL | 17 | | 2 | 63 | 97 | GSLLVNHATSTATGGSGGNGINPRGMPAGPDALFG | 35 | | 3 | 126 | 130 | HDPTK | 5 | | 4 | 132 | 143 | TVVGEPAVLPGE | 12 | | 5 | 175 | 200 | WYGLGPFDELRPFDLNQTTPPHGPTN | 26 | | 6 | 220 | 226 | GDPTVNN | 7 | | 7 | 238 | 260 | IHTSCYATPSVSYKGVMSSPNGT | 23 | | 8 | 300 | 306 | KTVIPGQ | 7 | | 9 | 339 | 356 | AEIIGEPIDLTTFNNDPG | 18 | | 10 | 375 | 375 | G | 1 | | 11 | 388 | 403 | TKKPVQHALLTPLGLD | 16 | | **BepiPred Linear Epitope Prediction 2.0**  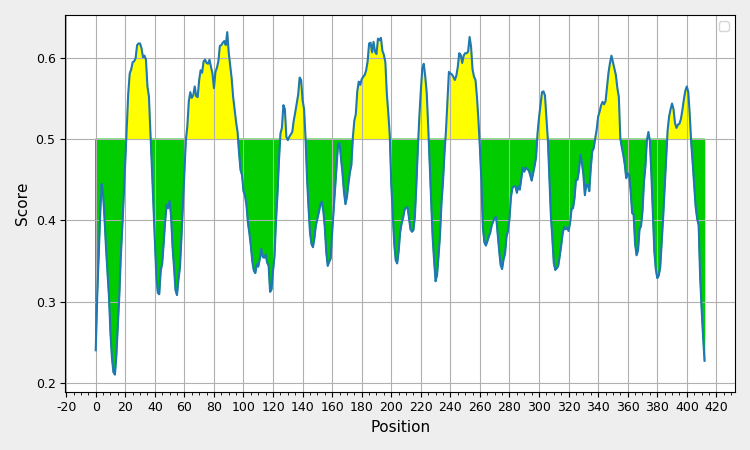 |
| --- | --- | --- | --- | --- | --- | --- | --- | --- | --- | --- | --- | --- | --- | --- | --- | --- | --- | --- | --- | --- | --- | --- | --- | --- | --- | --- | --- | --- | --- | --- | --- | --- | --- | --- | --- | --- | --- | --- | --- | --- | --- | --- | --- | --- | --- | --- | --- | --- | --- | --- | --- | --- | --- | --- | --- | --- | --- | --- | --- | --- | --- |

| **Protein name: 3-carboxymuconate cyclase (Gp60–70)**  **Predicted peptides: Ss446**   | **No.** | **Start** | **End** | **Peptide** | **Length** | | --- | --- | --- | --- | --- | | 1 | 22 | 38 | HPTNDRYYADADSEACL | 17 | | 2 | 63 | 97 | GSLLVNHATSTATGGSGGNGINPRGMPAGPDALFG | 35 | | 3 | 126 | 130 | HDPTK | 5 | | 4 | 132 | 143 | TVVGEPAVLPGE | 12 | | 5 | 175 | 200 | WYGLGPFDELRPFDLNQTTPPHGPTN | 26 | | 6 | 220 | 226 | GDPTVNN | 7 | | 7 | 238 | 260 | IHTSCYATPSVSYKGVMSSPNGT | 23 | | 8 | 300 | 306 | KTVIPGQ | 7 | | 9 | 339 | 356 | AEIIGEPIDLTTFNNDPG | 18 | | 10 | 375 | 375 | G | 1 | | 11 | 388 | 403 | TKKPVQHALLTPLGLD | 16 | | **BepiPred Linear Epitope Prediction 2.0**  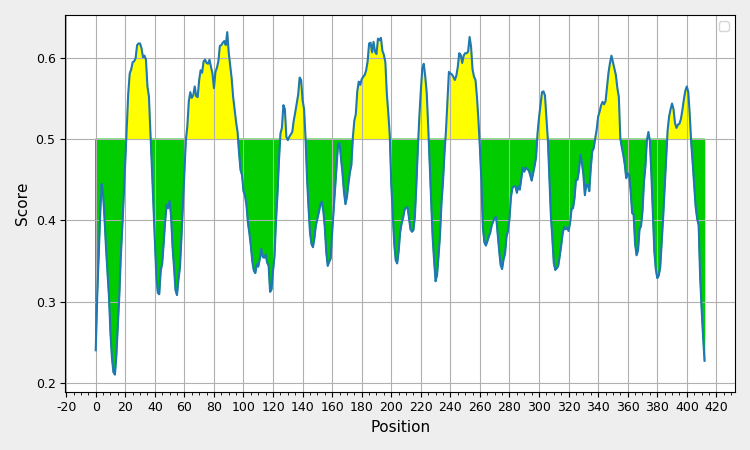 |
| --- | --- | --- | --- | --- | --- | --- | --- | --- | --- | --- | --- | --- | --- | --- | --- | --- | --- | --- | --- | --- | --- | --- | --- | --- | --- | --- | --- | --- | --- | --- | --- | --- | --- | --- | --- | --- | --- | --- | --- | --- | --- | --- | --- | --- | --- | --- | --- | --- | --- | --- | --- | --- | --- | --- | --- | --- | --- | --- | --- | --- | --- |

| **Protein name: 3-carboxymuconate cyclase (Gp60–70)**  **Predicted peptides: FMR8595**   | **No.** | **Start** | **End** | **Peptide** | **Length** | | --- | --- | --- | --- | --- | | 1 | 22 | 38 | HPTNDRYYADADSEACL | 17 | | 2 | 63 | 97 | GSLLVNHATSTATGGSGGNGINPRGMPAGPDALFG | 35 | | 3 | 126 | 130 | HDPTK | 5 | | 4 | 132 | 143 | TVVGEPAVLPGE | 12 | | 5 | 175 | 200 | WYGLGPFDELRPFDLNQTTPPHGPTN | 26 | | 6 | 220 | 226 | GDPTVNN | 7 | | 7 | 238 | 260 | IHTSCYATPSVSYKGVMSSPNGT | 23 | | 8 | 300 | 306 | KTVIPGQ | 7 | | 9 | 339 | 356 | AEIIGEPIDLTTFNNDPG | 18 | | 10 | 375 | 375 | G | 1 | | 11 | 388 | 403 | TKKPVQHALLTPLGLD | 16 | | **BepiPred Linear Epitope Prediction 2.0**  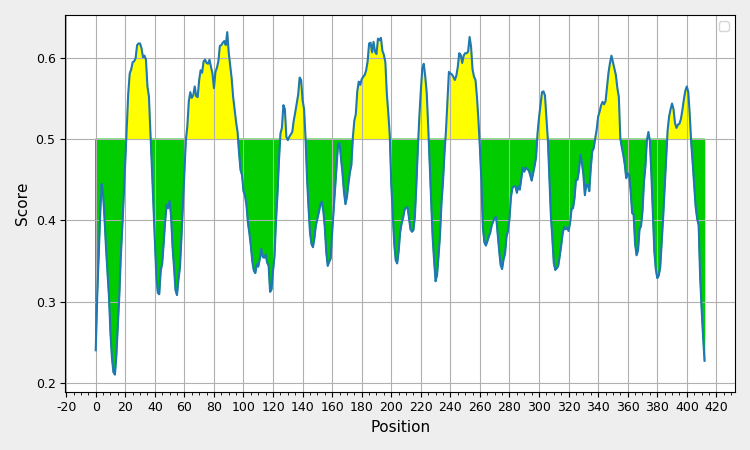 |
| --- | --- | --- | --- | --- | --- | --- | --- | --- | --- | --- | --- | --- | --- | --- | --- | --- | --- | --- | --- | --- | --- | --- | --- | --- | --- | --- | --- | --- | --- | --- | --- | --- | --- | --- | --- | --- | --- | --- | --- | --- | --- | --- | --- | --- | --- | --- | --- | --- | --- | --- | --- | --- | --- | --- | --- | --- | --- | --- | --- | --- | --- |

| **Protein name: 3-carboxymuconate cyclase (Gp60–70)**  **Predicted peptides: CBS120340**   | **No.** | **Start** | **End** | **Peptide** | **Length** | | --- | --- | --- | --- | --- | | 1 | 22 | 38 | HPTNDRYYADADSEACL | 17 | | 2 | 63 | 97 | GSLLVNHATSTATGGSGGNGINPRGMPAGPDALFG | 35 | | 3 | 126 | 130 | HDPTK | 5 | | 4 | 132 | 143 | TVVGEPAVLPGE | 12 | | 5 | 175 | 200 | WYGLGPFDELRPFDLNQTTPPHGPTN | 26 | | 6 | 220 | 226 | GDPTVNN | 7 | | 7 | 238 | 260 | IHTSCYATPSVSYKGVMSSPNGT | 23 | | 8 | 300 | 306 | KTVIPGQ | 7 | | 9 | 339 | 356 | AEIIGEPIDLTTFNNDPG | 18 | | 10 | 375 | 375 | G | 1 | | 11 | 388 | 403 | TKKPVQHALLTPLGLD | 16 | | **BepiPred Linear Epitope Prediction 2.0**  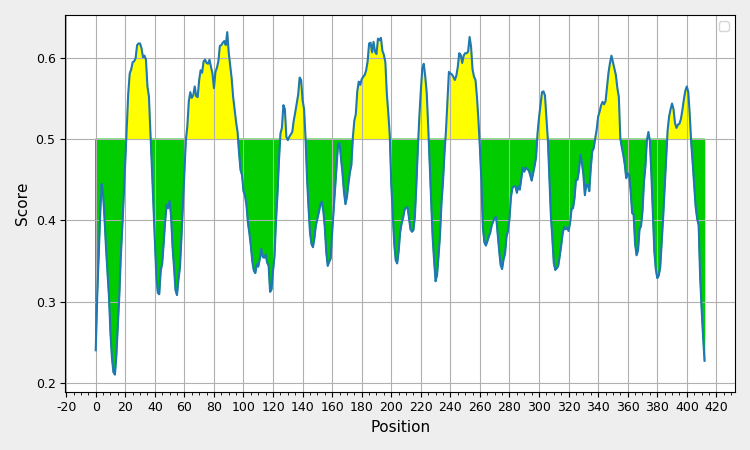 |
| --- | --- | --- | --- | --- | --- | --- | --- | --- | --- | --- | --- | --- | --- | --- | --- | --- | --- | --- | --- | --- | --- | --- | --- | --- | --- | --- | --- | --- | --- | --- | --- | --- | --- | --- | --- | --- | --- | --- | --- | --- | --- | --- | --- | --- | --- | --- | --- | --- | --- | --- | --- | --- | --- | --- | --- | --- | --- | --- | --- | --- | --- |

| **Protein name: 3-carboxymuconate cyclase (Gp60–70)**  **Predicted peptides: Ss471**   | **No.** | **Start** | **End** | **Peptide** | **Length** | | --- | --- | --- | --- | --- | | 1 | 22 | 38 | HPTNDRYYADADSEACL | 17 | | 2 | 63 | 97 | GSLLVNHATSTATGGSGGNGINPRGMPAGPDALFG | 35 | | 3 | 126 | 130 | HDPTK | 5 | | 4 | 132 | 143 | TVVGEPAVLPGE | 12 | | 5 | 175 | 200 | WYGLGPFDELRPFDLNQTTPPHGPTN | 26 | | 6 | 220 | 226 | GDPTVNN | 7 | | 7 | 238 | 260 | IHTSCYATPSVSYKGVMSSPNGT | 23 | | 8 | 300 | 306 | KTVIPGQ | 7 | | 9 | 339 | 356 | AEIIGEPIDLTTFNNDPG | 18 | | 10 | 375 | 375 | G | 1 | | 11 | 388 | 403 | TKKPVQHALLTPLGLD | 16 | | **BepiPred Linear Epitope Prediction 2.0**  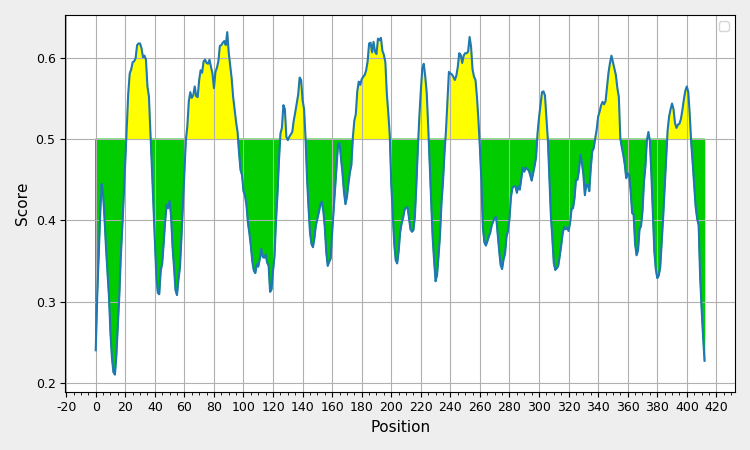 |
| --- | --- | --- | --- | --- | --- | --- | --- | --- | --- | --- | --- | --- | --- | --- | --- | --- | --- | --- | --- | --- | --- | --- | --- | --- | --- | --- | --- | --- | --- | --- | --- | --- | --- | --- | --- | --- | --- | --- | --- | --- | --- | --- | --- | --- | --- | --- | --- | --- | --- | --- | --- | --- | --- | --- | --- | --- | --- | --- | --- | --- | --- |

| **Protein name: 3-carboxymuconate cyclase (Gp60–70)**  **Predicted peptides: Ss472**   | **No.** | **Start** | **End** | **Peptide** | **Length** | | --- | --- | --- | --- | --- | | 1 | 22 | 38 | HPTNDRYYADADSEACL | 17 | | 2 | 63 | 97 | GSLLVNHATSTATGGSGGNGINPRGMPAGPDALFG | 35 | | 3 | 126 | 130 | HDPTK | 5 | | 4 | 132 | 143 | TVVGEPAVLPGE | 12 | | 5 | 175 | 200 | WYGLGPFDELRPFDLNQTTPPHGPTN | 26 | | 6 | 220 | 226 | GDPTVNN | 7 | | 7 | 238 | 260 | IHTSCYATPSVSYKGVMSSPNGT | 23 | | 8 | 300 | 306 | KTVIPGQ | 7 | | 9 | 339 | 356 | AEIIGEPIDLTTFNNDPG | 18 | | 10 | 375 | 375 | G | 1 | | 11 | 388 | 403 | TKKPVQHALLTPLGLD | 16 | | **BepiPred Linear Epitope Prediction 2.0**  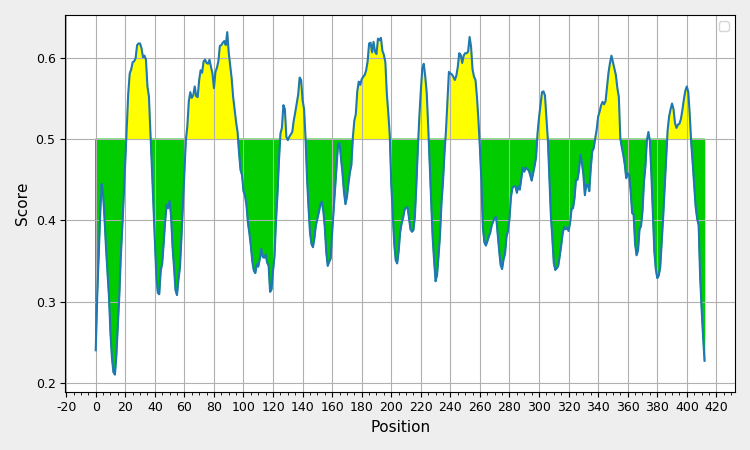 |
| --- | --- | --- | --- | --- | --- | --- | --- | --- | --- | --- | --- | --- | --- | --- | --- | --- | --- | --- | --- | --- | --- | --- | --- | --- | --- | --- | --- | --- | --- | --- | --- | --- | --- | --- | --- | --- | --- | --- | --- | --- | --- | --- | --- | --- | --- | --- | --- | --- | --- | --- | --- | --- | --- | --- | --- | --- | --- | --- | --- | --- | --- |

| **Protein name: 3-carboxymuconate cyclase (Gp60–70)**  **Predicted peptides: Ss236**   | **No.** | **Start** | **End** | **Peptide** | **Length** | | --- | --- | --- | --- | --- | | 1 | 22 | 38 | HPTNDRYYADADSEACL | 17 | | 2 | 63 | 97 | GSLLVNHATSTATGGSGGNGINPRGMPAGPDALFG | 35 | | 3 | 126 | 130 | HDPTK | 5 | | 4 | 132 | 143 | TVVGEPAVLPGE | 12 | | 5 | 175 | 200 | WYGLGPFDELRPFDLNQTTPPHGPTN | 26 | | 6 | 220 | 226 | GDPTVNN | 7 | | 7 | 238 | 260 | IHTSCYATPSVSYKGVMSSPNGT | 23 | | 8 | 300 | 306 | KTVIPGQ | 7 | | 9 | 339 | 356 | AEIIGEPIDLTTFNNDPG | 18 | | 10 | 375 | 375 | G | 1 | | 11 | 388 | 403 | TKKPVQHALLTPLGLD | 16 | | **BepiPred Linear Epitope Prediction 2.0**  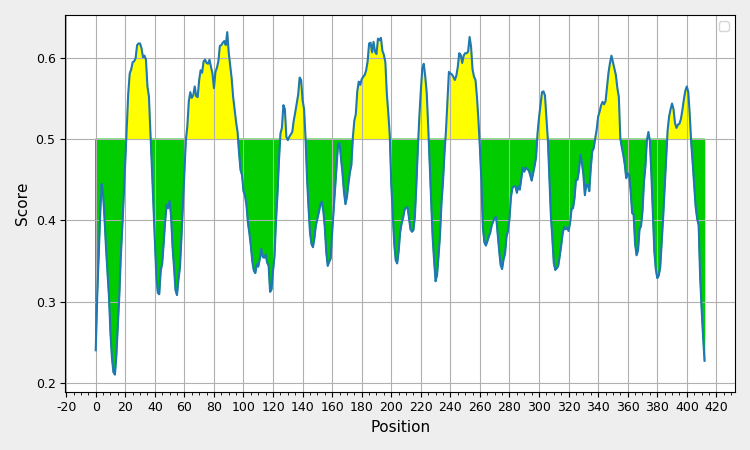 |
| --- | --- | --- | --- | --- | --- | --- | --- | --- | --- | --- | --- | --- | --- | --- | --- | --- | --- | --- | --- | --- | --- | --- | --- | --- | --- | --- | --- | --- | --- | --- | --- | --- | --- | --- | --- | --- | --- | --- | --- | --- | --- | --- | --- | --- | --- | --- | --- | --- | --- | --- | --- | --- | --- | --- | --- | --- | --- | --- | --- | --- | --- |

| **Protein name: 3-carboxymuconate cyclase (Gp60–70)**  **Predicted peptides: Ss520**   | **No.** | **Start** | **End** | **Peptide** | **Length** | | --- | --- | --- | --- | --- | | 1 | 22 | 38 | HPTNDRYYADADSEACL | 17 | | 2 | 62 | 97 | NGSLLVNHATSTATGGSGGNGINPRGMPAGPDALFG | 36 | | 3 | 126 | 130 | HDPTK | 5 | | 4 | 132 | 143 | TVVGEPAVLPGE | 12 | | 5 | 176 | 200 | YGLGPFDELRPFDLNQTTPPHGPTN | 25 | | 6 | 220 | 226 | GDPTVNN | 7 | | 7 | 238 | 260 | IHTSCYATPSVSYKGVMSSPNGT | 23 | | 8 | 300 | 306 | KTVIPGQ | 7 | | 9 | 340 | 355 | EIIGEPIDLTTFNNDP | 16 | | 10 | 375 | 375 | G | 1 | | 11 | 389 | 403 | KKPVQNALLTPLGLD | 15 | | **BepiPred Linear Epitope Prediction 2.0**  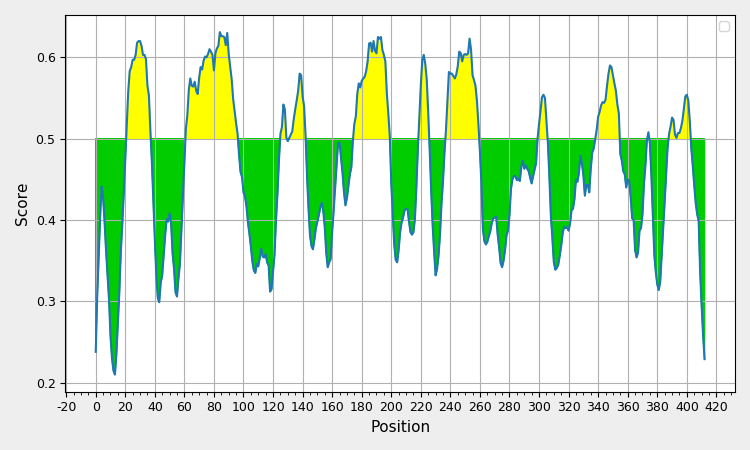 |
| --- | --- | --- | --- | --- | --- | --- | --- | --- | --- | --- | --- | --- | --- | --- | --- | --- | --- | --- | --- | --- | --- | --- | --- | --- | --- | --- | --- | --- | --- | --- | --- | --- | --- | --- | --- | --- | --- | --- | --- | --- | --- | --- | --- | --- | --- | --- | --- | --- | --- | --- | --- | --- | --- | --- | --- | --- | --- | --- | --- | --- | --- |

| **Protein name: 3-carboxymuconate cyclase (Gp60–70)**  **Predicted peptides: 5659**   | **No.** | **Start** | **End** | **Peptide** | **Length** | | --- | --- | --- | --- | --- | | 1 | 22 | 38 | HPTNDRYYADADSEACL | 17 | | 2 | 63 | 97 | GSLLVNHATSTATGGSGGNGINPRGMPAGPDALFG | 35 | | 3 | 126 | 143 | HDPTKLTVVGEPAVLPGE | 18 | | 4 | 175 | 200 | WYGLGPFDELRPFDLNQTTPPHGPTN | 26 | | 5 | 220 | 226 | GDPTVNN | 7 | | 6 | 238 | 260 | IHTSCYATPSVSYKGVMSSPNGT | 23 | | 7 | 301 | 306 | TVIPGQ | 6 | | 8 | 341 | 355 | IIGEPIDLTTFNNDP | 15 | | 9 | 374 | 375 | NG | 2 | | 10 | 389 | 403 | KKPVQHALLTPLGLD | 15 | | **BepiPred Linear Epitope Prediction 2.0**  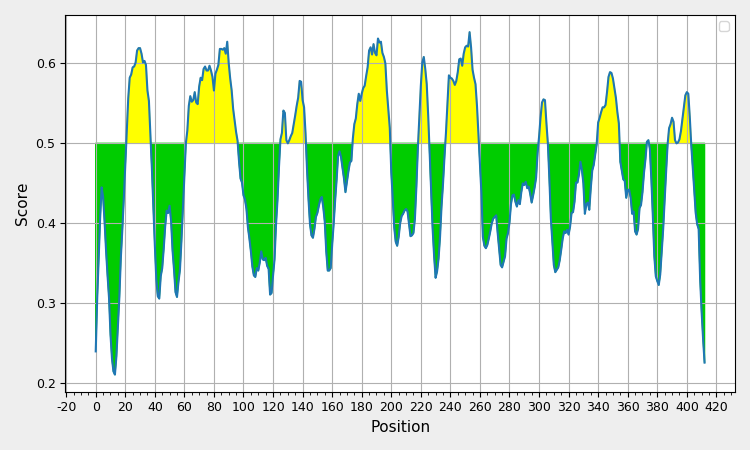 |
| --- | --- | --- | --- | --- | --- | --- | --- | --- | --- | --- | --- | --- | --- | --- | --- | --- | --- | --- | --- | --- | --- | --- | --- | --- | --- | --- | --- | --- | --- | --- | --- | --- | --- | --- | --- | --- | --- | --- | --- | --- | --- | --- | --- | --- | --- | --- | --- | --- | --- | --- | --- | --- | --- | --- | --- | --- |

| **Protein name: 3-carboxymuconate cyclase (Gp60–70)**  **Predicted peptides: Ss41**   | **No.** | **Start** | **End** | **Peptide** | **Length** | | --- | --- | --- | --- | --- | | 1 | 22 | 38 | HPTNDRYYADADSEACL | 17 | | 2 | 63 | 97 | GSLLVNHATSTATGGSGGNGINPRGMPAGPDALFG | 35 | | 3 | 126 | 130 | HDPTK | 5 | | 4 | 132 | 143 | TVVGEPAVLPGE | 12 | | 5 | 175 | 200 | WYGLGPFDELRPFDLNQTTPPHGPTN | 26 | | 6 | 220 | 226 | GDPTVNN | 7 | | 7 | 238 | 260 | IQTSCYATPSVSYKGVMSSPNGT | 23 | | 8 | 300 | 306 | KTVIPGQ | 7 | | 9 | 339 | 356 | AEIIGEPIDLTTFNNDPG | 18 | | 10 | 375 | 375 | G | 1 | | 11 | 388 | 403 | TKKPVQHALLTPLGLD | 16 | | **BepiPred Linear Epitope Prediction 2.0**  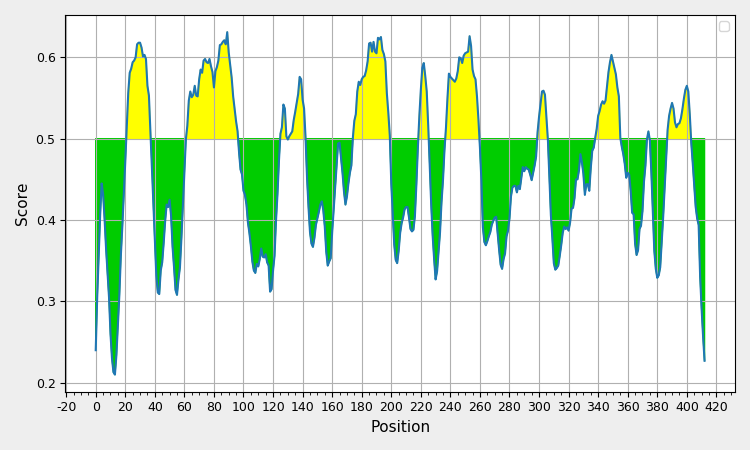 |
| --- | --- | --- | --- | --- | --- | --- | --- | --- | --- | --- | --- | --- | --- | --- | --- | --- | --- | --- | --- | --- | --- | --- | --- | --- | --- | --- | --- | --- | --- | --- | --- | --- | --- | --- | --- | --- | --- | --- | --- | --- | --- | --- | --- | --- | --- | --- | --- | --- | --- | --- | --- | --- | --- | --- | --- | --- | --- | --- | --- | --- | --- |

| **Protein name: 3-carboxymuconate cyclase (Gp60–70)**  **Predicted peptides: Ss334**   | **No.** | **Start** | **End** | **Peptide** | **Length** | | --- | --- | --- | --- | --- | | 1 | 22 | 38 | HPTNDRYYADADSEACL | 17 | | 2 | 63 | 97 | GSLLVNHATSTATGGSGGNGINPRGMPAGPDALFG | 35 | | 3 | 126 | 130 | HDPTK | 5 | | 4 | 132 | 143 | TVVGEPAVLPGE | 12 | | 5 | 175 | 200 | WYGLGPFDELRPFDLNQTTPPHGPTN | 26 | | 6 | 220 | 226 | GDPTVNN | 7 | | 7 | 238 | 260 | IQTSCYATPSVSYKGVMSSPNGT | 23 | | 8 | 300 | 306 | KTVIPGQ | 7 | | 9 | 339 | 356 | AEIIGEPIDLTTFNNDPG | 18 | | 10 | 375 | 375 | G | 1 | | 11 | 388 | 403 | TKKPVQHALLTPLGLD | 16 | | **BepiPred Linear Epitope Prediction 2.0**  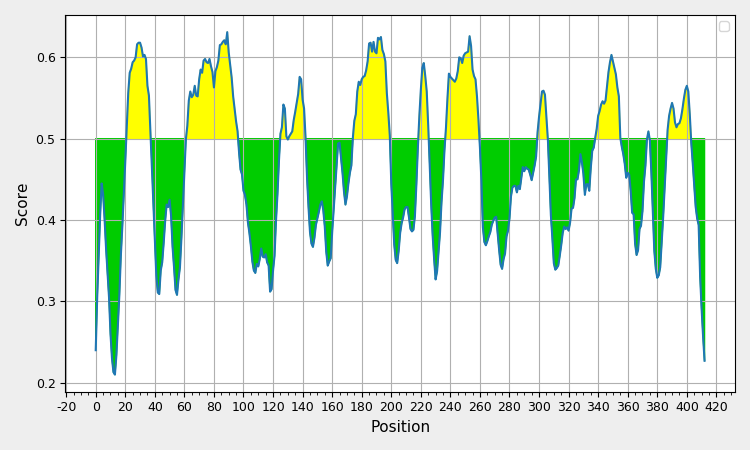 |
| --- | --- | --- | --- | --- | --- | --- | --- | --- | --- | --- | --- | --- | --- | --- | --- | --- | --- | --- | --- | --- | --- | --- | --- | --- | --- | --- | --- | --- | --- | --- | --- | --- | --- | --- | --- | --- | --- | --- | --- | --- | --- | --- | --- | --- | --- | --- | --- | --- | --- | --- | --- | --- | --- | --- | --- | --- | --- | --- | --- | --- | --- |

| **Protein name: 3-carboxymuconate cyclase (Gp60–70)**  **Predicted peptides: Ss49**   | **No.** | **Start** | **End** | **Peptide** | **Length** | | --- | --- | --- | --- | --- | | 1 | 22 | 38 | HPTNDRYYADADSEACL | 17 | | 2 | 63 | 97 | GSLLVNHATSTATGGSGGNGINPRGMPAGPDALFG | 35 | | 3 | 128 | 129 | PT | 2 | | 4 | 135 | 143 | GEPAVLPGE | 9 | | 5 | 175 | 200 | WYGLGPFDELRPFDLNQTTPPHGPTN | 26 | | 6 | 220 | 226 | GDPTVNN | 7 | | 7 | 238 | 260 | IQTSCYATPSVSYKGVMSSPNGT | 23 | | 8 | 300 | 307 | KTVIPGQD | 8 | | 9 | 340 | 355 | EIIGEPIDLTTFNNDP | 16 | | 10 | 375 | 375 | G | 1 | | 11 | 388 | 403 | TKKPVQHALLTQLGLD | 16 | | **BepiPred Linear Epitope Prediction 2.0**  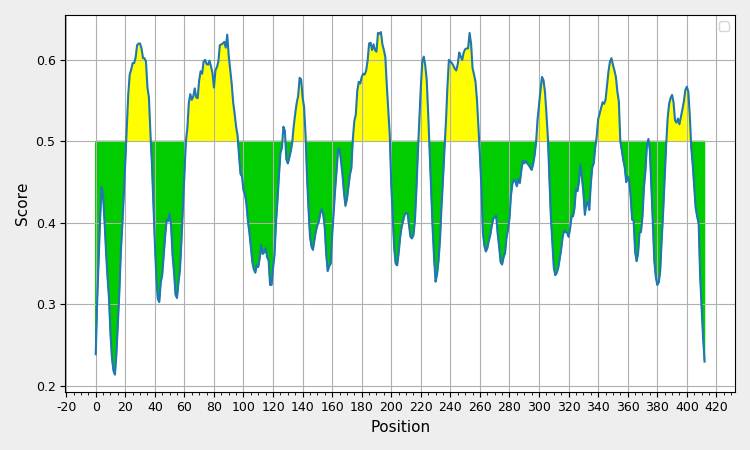 |
| --- | --- | --- | --- | --- | --- | --- | --- | --- | --- | --- | --- | --- | --- | --- | --- | --- | --- | --- | --- | --- | --- | --- | --- | --- | --- | --- | --- | --- | --- | --- | --- | --- | --- | --- | --- | --- | --- | --- | --- | --- | --- | --- | --- | --- | --- | --- | --- | --- | --- | --- | --- | --- | --- | --- | --- | --- | --- | --- | --- | --- | --- |

| **Protein name: 3-carboxymuconate cyclase (Gp60–70)**  **Predicted peptides: S17**   | **No.** | **Start** | **End** | **Peptide** | **Length** | | --- | --- | --- | --- | --- | | 1 | 22 | 38 | HPTNDRYYADADSEACL | 17 | | 2 | 63 | 97 | GSLLVNHATSTATGGSGGNGINPRGMPAGPDALFG | 35 | | 3 | 126 | 130 | HDPTK | 5 | | 4 | 132 | 143 | TVVGEPAVLPGE | 12 | | 5 | 175 | 200 | WYGLGPFDELRPFDLNQTTPPHGPTN | 26 | | 6 | 220 | 226 | GDPTVNN | 7 | | 7 | 238 | 260 | IHTSCYATPSVSYKGVMSSPNGT | 23 | | 8 | 300 | 306 | KTVIPGQ | 7 | | 9 | 339 | 356 | AEIIGEPIDLTTFNNDPG | 18 | | 10 | 375 | 375 | G | 1 | | 11 | 388 | 403 | TKKPVQHALLTPLGLD | 16 | | **BepiPred Linear Epitope Prediction 2.0**  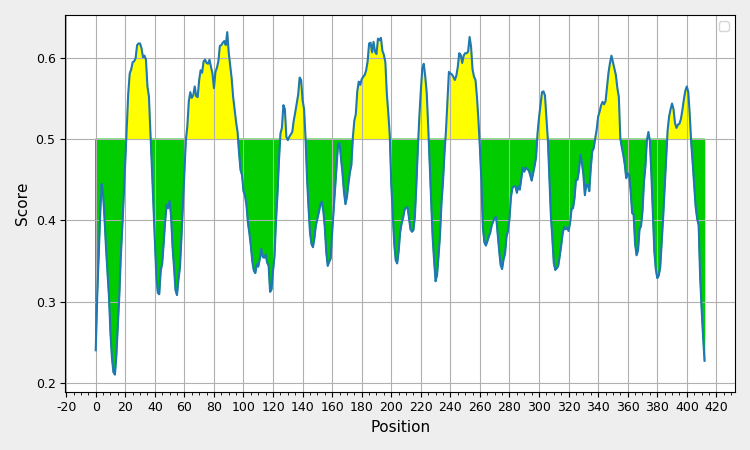 |
| --- | --- | --- | --- | --- | --- | --- | --- | --- | --- | --- | --- | --- | --- | --- | --- | --- | --- | --- | --- | --- | --- | --- | --- | --- | --- | --- | --- | --- | --- | --- | --- | --- | --- | --- | --- | --- | --- | --- | --- | --- | --- | --- | --- | --- | --- | --- | --- | --- | --- | --- | --- | --- | --- | --- | --- | --- | --- | --- | --- | --- | --- |

| **Protein name: 3-carboxymuconate cyclase (Gp60–70)**  **Predicted peptides: SS01**   | **No.** | **Start** | **End** | **Peptide** | **Length** | | --- | --- | --- | --- | --- | | 1 | 22 | 38 | HPTNDRYYADADSEACL | 17 | | 2 | 63 | 97 | GSLLVNHATSTATGGSGGNGINPRGMPAGPDALFG | 35 | | 3 | 126 | 130 | HDPTK | 5 | | 4 | 132 | 143 | TVVGEPAVLPGE | 12 | | 5 | 175 | 200 | WYGLGPFDELRPFDLNQTTPPHGPTN | 26 | | 6 | 220 | 226 | GDPTVNN | 7 | | 7 | 238 | 260 | IHTSCYATPSVSYKGVMSSPNGT | 23 | | 8 | 300 | 306 | KTVIPGQ | 7 | | 9 | 339 | 356 | AEIIGEPIDLTTFNNDPG | 18 | | 10 | 375 | 375 | G | 1 | | 11 | 388 | 403 | TKKPVQHALLTPLGLD | 16 | | **BepiPred Linear Epitope Prediction 2.0** |
| --- | --- | --- | --- | --- | --- | --- | --- | --- | --- | --- | --- | --- | --- | --- | --- | --- | --- | --- | --- | --- | --- | --- | --- | --- | --- | --- | --- | --- | --- | --- | --- | --- | --- | --- | --- | --- | --- | --- | --- | --- | --- | --- | --- | --- | --- | --- | --- | --- | --- | --- | --- | --- | --- | --- | --- | --- | --- | --- | --- | --- | --- |

| **Protein name: 3-carboxymuconate cyclase (Gp60–70)**  **Predicted peptides: Ss534**   | **No.** | **Start** | **End** | **Peptide** | **Length** | | --- | --- | --- | --- | --- | | 1 | 23 | 38 | PTNDRYYADADSEACL | 16 | | 2 | 62 | 97 | NGSLLVNHATSTATGGSGGNGINPRGMPAGPDELFG | 36 | | 3 | 128 | 129 | PT | 2 | | 4 | 133 | 142 | VVGEPAVLPG | 10 | | 5 | 175 | 199 | WYGLGPFDELRPFDLNQTTPPHGPT | 25 | | 6 | 220 | 227 | GDPTVNNT | 8 | | 7 | 238 | 260 | IHTSCYATPSVSYKGVMSSPNGT | 23 | | 8 | 300 | 307 | KTVIPGQD | 8 | | 9 | 339 | 355 | AEIIGEPIDLTTFNNDP | 17 | | 10 | 375 | 375 | G | 1 | | 11 | 390 | 392 | NPV | 3 | | 12 | 395 | 403 | ANLRPLGLD | 9 | | **BepiPred Linear Epitope Prediction 2.0** |
| --- | --- | --- | --- | --- | --- | --- | --- | --- | --- | --- | --- | --- | --- | --- | --- | --- | --- | --- | --- | --- | --- | --- | --- | --- | --- | --- | --- | --- | --- | --- | --- | --- | --- | --- | --- | --- | --- | --- | --- | --- | --- | --- | --- | --- | --- | --- | --- | --- | --- | --- | --- | --- | --- | --- | --- | --- | --- | --- | --- | --- | --- | --- | --- | --- | --- | --- |

| **Protein name: 3-carboxymuconate cyclase (Gp60–70)**  **Predicted peptides: Ss545**   | **No.** | **Start** | **End** | **Peptide** | **Length** | | --- | --- | --- | --- | --- | | 1 | 6 | 6 | K | 1 | | 2 | 23 | 38 | PTNDRYYADADSEACL | 16 | | 3 | 63 | 97 | GSLLVNHATSTATGGSGGNGINPRGMPAGPDALIG | 35 | | 4 | 126 | 130 | HDPTK | 5 | | 5 | 133 | 143 | VVGEPAVLPGE | 11 | | 6 | 176 | 199 | YGLGPFDELRPFDLNQTTPPHGPT | 24 | | 7 | 220 | 227 | GDPTVNNT | 8 | | 8 | 238 | 260 | IHTSCYATPSVSYKGVMSSPNGT | 23 | | 9 | 300 | 306 | KTVIPGQ | 7 | | 10 | 340 | 356 | EIIGEPIDLTTFNNDPG | 17 | | 11 | 375 | 375 | G | 1 | | 12 | 390 | 392 | NPV | 3 | | 13 | 394 | 403 | HANLRPLGLD | 10 | | **BepiPred Linear Epitope Prediction 2.0** |
| --- | --- | --- | --- | --- | --- | --- | --- | --- | --- | --- | --- | --- | --- | --- | --- | --- | --- | --- | --- | --- | --- | --- | --- | --- | --- | --- | --- | --- | --- | --- | --- | --- | --- | --- | --- | --- | --- | --- | --- | --- | --- | --- | --- | --- | --- | --- | --- | --- | --- | --- | --- | --- | --- | --- | --- | --- | --- | --- | --- | --- | --- | --- | --- | --- | --- | --- | --- | --- | --- | --- | --- |

| **Protein name: 3-carboxymuconate cyclase (Gp60–70)**  **Predicted peptides: Ss187**   | **No.** | **Start** | **End** | **Peptide** | **Length** | | --- | --- | --- | --- | --- | | 1 | 24 | 36 | TIEHVGVESNACI | 13 | | 2 | 61 | 80 | GTLLVDLATSTATGGSGGNV | 20 | | 3 | 105 | 107 | DPT | 3 | | 4 | 111 | 121 | VVGEPAMLPGE | 11 | | 5 | 153 | 178 | WYGLGPFDEIRPFELNQTTPPHGPTN | 26 | | 6 | 199 | 204 | DPAVNN | 6 | | 7 | 215 | 238 | NIHSSCYAIPSVSHKGVISSPNGT | 24 | | 8 | 278 | 284 | KTVIAGQ | 7 | | 9 | 319 | 333 | IIGDPIDLTPLNNDP | 15 | | 10 | 353 | 354 | GT | 2 | | 11 | 366 | 381 | TKELVQHALLTPLGLD | 16 | | **BepiPred Linear Epitope Prediction 2.0** |
| --- | --- | --- | --- | --- | --- | --- | --- | --- | --- | --- | --- | --- | --- | --- | --- | --- | --- | --- | --- | --- | --- | --- | --- | --- | --- | --- | --- | --- | --- | --- | --- | --- | --- | --- | --- | --- | --- | --- | --- | --- | --- | --- | --- | --- | --- | --- | --- | --- | --- | --- | --- | --- | --- | --- | --- | --- | --- | --- | --- | --- | --- |

**Supplementary Table S4.** Predicted glycosylation sites and their conservation across *Sporothrix* species using GlycoEP [10].

*Note: No. of sequences=104. Threshold Selected: 0.0****.***

>Ss05 Length = 412

**Potential N-Linked Glycosylated Sites:**

MHYY**N**KLKFLALASVISATSAHPTSDHYYADAESEACL**N**GKAVYVTS**N**TEH**N**SVVAIPIAR**N**GSLLV**N**YATSTATGGRGG**N**GI**N**PRGMPAGPDALFGQGS

ITIAGDYLFAV**N**AGS**N**TVTMLAIDKHDPTKVTVVGEPAELPGEFPTTVGASDKF**N**LVCVGLTGAKAGVSCASYSWYGLGPFDELRPFDLHQTTPPHGPT

**N**TVSHVFFSGDQETVFTTVKGDPAV**NN**TGFLAAYPVEHIHSSCYATPSVSHKGVISSPEGTAVLFGSTPIPDTT**N**LFATDASFGAVILGIEDYEASTLY

KTVIPGQDATCWVAICPATHTAFVTDIRM**N**RLVEMSLA**N**AEIIGEPIDLTTFS**N**DPGLTEIRSGGSFVYALSPG**N**GTTEAWITVL**N**ALTKKPVQHALLT

PLGLDR**N**AMGMAILV

------------------------------------------------

Position Residue Score Prediction

------------------------------------------------

5 **N**KL -0.77832304 Non-glycosylated

39 **N**GK -1.130584 Non-glycosylated

48 **N**TE -1.2741531 Non-glycosylated

52 **N**SV -0.83972275 Non-glycosylated

**62 NGS 1.1524071 Potential Glycosylated**

68 **N**YA -0.9795019 Non-glycosylated

81 **N**GI -1.2423291 Non-glycosylated

84 **N**PR -1.8662045 Non-glycosylated

112 **N**AG -1.0674394 Non-glycosylated

116 **N**TV -0.66346953 Non-glycosylated

155 **N**LV -1.0444821 Non-glycosylated

200 **N**TV -1.0299987 Non-glycosylated

**225 NNT 0.88963983 Potential Glycosylated**

226 **N**TG -1.0135771 Non-glycosylated

274 **N**LF -1.3425718 Non-glycosylated

328 **N**RL -1.0222076 Non-glycosylated

337 **N**AE -1.1197133 Non-glycosylated

352 **N**DP -1.1451453 Non-glycosylated

**373 NGT 1.2707122 Potential Glycosylated**

384 **N**AL -1.0307742 Non-glycosylated

404 **N**AM -1.3410327 Non-glycosylated

***********************************

>Ss07 Length = 412

**Potential N-Linked Glycosylated Sites:**

MHYY**N**KLKFLALASVISATSAHPTSDHYYADAESEACL**N**GKAVYVTS**N**TEH**N**SVVAIPIAR**N**GSLLV**N**YATSTATGGRGG**N**GI**N**PRGMPAGPDALFGQGS

ITIAGDYLFAV**N**AGS**N**TVTMLAIDKHDPTKVTVVGEPAELPGEFPTTVGASDKF**N**LVCVGLTGAKAGVSCASYSWYGLGPFDELRPFDLHQTTPPHGPT

**N**TVSHVFFSGDQETVFTTVKGDPAV**NN**TGFLAAYPVEHIHSSCYATPSVSHKGVISSPEGTAVLFGSTPIPDTT**N**LFATDASFGAVILGIEDYEASTLY

KTVIPGQDATCWVAICPATHTAFVTDIRM**N**RLVEMSLA**N**AEIIGEPIDLTTFS**N**DPGLTEIRSGGSFVYALSPG**N**GTTEAWITVL**N**ALTKKPVQHALLT

PLGLDR**N**AMGMAILV

------------------------------------------------

Position Residue Score Prediction

------------------------------------------------

5 **N**KL -0.77832304 Non-glycosylated

39 **N**GK -1.130584 Non-glycosylated

48 **N**TE -1.2741531 Non-glycosylated

52 **N**SV -0.83972275 Non-glycosylated

**62 NGS 1.1524071 Potential Glycosylated**

68 **N**YA -0.9795019 Non-glycosylated

81 **N**GI -1.2423291 Non-glycosylated

84 **N**PR -1.8662045 Non-glycosylated

112 **N**AG -1.0674394 Non-glycosylated

116 **N**TV -0.66346953 Non-glycosylated

155 **N**LV -1.0444821 Non-glycosylated

200 **N**TV -1.0299987 Non-glycosylated

**225 NNT 0.88963983 Potential Glycosylated**

226 **N**TG -1.0135771 Non-glycosylated

274 **N**LF -1.3425718 Non-glycosylated

328 **N**RL -1.0222076 Non-glycosylated

337 **N**AE -1.1197133 Non-glycosylated

352 **N**DP -1.1451453 Non-glycosylated

**373 NGT 1.2707122 Potential Glycosylated**

384 **N**AL -1.0307742 Non-glycosylated

404 **N**AM -1.3410327 Non-glycosylated

***********************************

>Ss99 Length = 412

**Potential N-Linked Glycosylated Sites:**

MHYY**N**KLKFLALASVISATSAHPTSDHYYADAESEACL**N**GKAVYVTS**N**TEH**N**SVVAIPIAR**N**GSLLV**N**YATSTATGGRGG**N**GI**N**PRGMPAGPDALFGQGS

ITIAGDYLFAV**N**AGS**N**TVTMLAIDKHDPTKVTVVGEPAELPGEFPTTVGASDKF**N**LVCVGLTGAKAGVSCASYSWYGLGPFDELRPFDLHQTTPPHGPT

**N**TVSHVFFSGDQETVFTTVKGDPAV**NN**TGFLAAYPVEHIHSSCYATPSVSHKGVISSPEGTAVLFGSTPIPDTT**N**LFATDASFGAVILGIEDYEASTLY

KTVIPGQDATCWVAICPATHTAFVTDIRM**N**RLVEMSLA**N**AEIIGEPIDLTTFS**N**DPGLTEIRSGGSFVYALSPG**N**GTTEAWITVL**N**ALTKKPVQHALLT

PLGLDR**N**AMGMAILV

------------------------------------------------

Position Residue Score Prediction

------------------------------------------------

5 **N**KL -0.77832304 Non-glycosylated

39 **N**GK -1.130584 Non-glycosylated

48 **N**TE -1.2741531 Non-glycosylated

52 **N**SV -0.83972275 Non-glycosylated

**62 NGS 1.1524071 Potential Glycosylated**

68 **N**YA -0.9795019 Non-glycosylated

81 **N**GI -1.2423291 Non-glycosylated

84 **N**PR -1.8662045 Non-glycosylated

112 **N**AG -1.0674394 Non-glycosylated

116 **N**TV -0.66346953 Non-glycosylated

155 **N**LV -1.0444821 Non-glycosylated

200 **N**TV -1.0299987 Non-glycosylated

**225 NNT 0.88963983 Potential Glycosylated**

226 **N**TG -1.0135771 Non-glycosylated

274 **N**LF -1.3425718 Non-glycosylated

328 **N**RL -1.0222076 Non-glycosylated

337 **N**AE -1.1197133 Non-glycosylated

352 **N**DP -1.1451453 Non-glycosylated

**373 NGT 1.2707122 Potential Glycosylated**

384 **N**AL -1.0307742 Non-glycosylated

404 **N**AM -1.3410327 Non-glycosylated

***********************************

>5110 Length = 412

**Potential N-Linked Glycosylated Sites:**

MHYY**N**KLKFLALASVISATSAHPTSDHYYADAESEACL**N**GKAVYVTS**N**TEH**N**SVVAIPIAR**N**GSLLV**N**YATSTATGGRGG**N**GI**N**PRGMPAGPDALFGQGS

ITIAGDYLFAV**N**AGS**N**TVTMLAIDKHDPTKVTVVGEPAELPGEFPTTVGASDKF**N**LVCVGLTGAKAGVSCASYSWYGLGPFDELRPFDLHQTTPPHGPT

**N**TVSHVFFSGDQETVFTTVKGDPAV**NN**TGFLAAYPVEHIHSSCYATPSVSHKGVISSPEGTAVLFGSTPIPDTT**N**LFATDASFGAVILGIEDYEASTLY

KTVIPGQDATCWVAICPATHTAFVTDIRM**N**RLVEMSLA**N**AEIIGEPIDLTTFS**N**DPGLTEIRSGGSFVYALSPG**N**GTTEAWITVL**N**ALTKKPVQHALLT

PLGLDR**N**AMGMAILV

------------------------------------------------

Position Residue Score Prediction

------------------------------------------------

5 **N**KL -0.77832304 Non-glycosylated

39 **N**GK -1.130584 Non-glycosylated

48 **N**TE -1.2741531 Non-glycosylated

52 **N**SV -0.83972275 Non-glycosylated

**62 NGS 1.1524071 Potential Glycosylated**

68 **N**YA -0.9795019 Non-glycosylated

81 **N**GI -1.2423291 Non-glycosylated

84 **N**PR -1.8662045 Non-glycosylated

112 **N**AG -1.0674394 Non-glycosylated

116 **N**TV -0.66346953 Non-glycosylated

155 **N**LV -1.0444821 Non-glycosylated

200 **N**TV -1.0299987 Non-glycosylated

**225 NNT 0.88963983 Potential Glycosylated**

226 **N**TG -1.0135771 Non-glycosylated

274 **N**LF -1.3425718 Non-glycosylated

328 **N**RL -1.0222076 Non-glycosylated

337 **N**AE -1.1197133 Non-glycosylated

352 **N**DP -1.1451453 Non-glycosylated

**373 NGT 1.2707122 Potential Glycosylated**

384 **N**AL -1.0307742 Non-glycosylated

404 **N**AM -1.3410327 Non-glycosylated

***********************************

>Ss14 Length = 412

**Potential N-Linked Glycosylated Sites:**

MHYY**N**KLKFLALASVISATSAHPTSDHYYADAESEACL**N**GKAVYVTS**N**TEH**N**SVVAIPIAR**N**GSLLV**N**YATSTATGGRGG**N**GI**N**PRGMPAGPDALFGQGS

ITIAGDYLFAV**N**AGS**N**TVTMLAIDKHDPTKVTVVGEPAELPGEFPTTVGASDKF**N**LVCVGLTGAKAGVSCASYSWYGLGPFDELRPFDLHQTTPPHGPT

**N**TVSHVFFSGDQETVFTTVKGDPAV**NN**TGFLAAYPVEHIHSSCYATPSVSHKGVISSPEGTAVLFGSTPIPDTT**N**LFATDASFGAVILGIEDYEASTLY

KTVIPGQDATCWVAICPATHTAFVTDIRM**N**RLVEMSLA**N**AEIIGEPIDLTTFS**N**DPGLTEIRSGGSFVYALSPG**N**GTTEAWITVL**N**ALTKKPVQHALLT

PLGLDR**N**AMGMAILV

------------------------------------------------

Position Residue Score Prediction

------------------------------------------------

5 **N**KL -0.77832304 Non-glycosylated

39 **N**GK -1.130584 Non-glycosylated

48 **N**TE -1.2741531 Non-glycosylated

52 **N**SV -0.83972275 Non-glycosylated

**62 NGS 1.1524071 Potential Glycosylated**

68 **N**YA -0.9795019 Non-glycosylated

81 **N**GI -1.2423291 Non-glycosylated

84 **N**PR -1.8662045 Non-glycosylated

112 **N**AG -1.0674394 Non-glycosylated

116 **N**TV -0.66346953 Non-glycosylated

155 **N**LV -1.0444821 Non-glycosylated

200 **N**TV -1.0299987 Non-glycosylated

**225 NNT 0.88963983 Potential Glycosylated**

226 **N**TG -1.0135771 Non-glycosylated

274 **N**LF -1.3425718 Non-glycosylated

328 **N**RL -1.0222076 Non-glycosylated

337 **N**AE -1.1197133 Non-glycosylated

352 **N**DP -1.1451453 Non-glycosylated

**373 NGT 1.2707122 Potential Glycosylated**

384 **N**AL -1.0307742 Non-glycosylated

404 **N**AM -1.3410327 Non-glycosylated

***********************************

>Ss33 Length = 412

**Potential N-Linked Glycosylated Sites:**

MHYY**N**KLKFLALASVISATSAHPTSDHYYADAESEACL**N**GKAVYVTS**N**TEH**N**SVVAIPIAR**N**GSLLV**N**YATSTATGGRGG**N**GI**N**PRGMPAGPDALFGQGS

ITIAGDYLFAV**N**AGS**N**TVTMLAIDKHDPTKVTVVGEPAELPGEFPTTVGASDKF**N**LVCVGLTGAKAGVSCASYSWYGLGPFDELRPFDLHQTTPPHGPT

**N**TVSHVFFSGDQETVFTTVKGDPAV**NN**TGFLAAYPVEHIHSSCYATPSVSHKGVISSPEGTAVLFGSTPIPDTT**N**LFATDASFGAVILGIEDYEASTLY

KTVIPGQDATCWVAICPATHTAFVTDIRM**N**RLVEMSLA**N**AEIIGEPIDLTTFS**N**DPGLTEIRSGGSFVYALSPG**N**GTTEAWITVL**N**ALTKKPVQHALLT

PLGLDR**N**AMGMAILV

------------------------------------------------

Position Residue Score Prediction

------------------------------------------------

5 **N**KL -0.77832304 Non-glycosylated

39 **N**GK -1.130584 Non-glycosylated

48 **N**TE -1.2741531 Non-glycosylated

52 **N**SV -0.83972275 Non-glycosylated

**62 NGS 1.1524071 Potential Glycosylated**

68 **N**YA -0.9795019 Non-glycosylated

81 **N**GI -1.2423291 Non-glycosylated

84 **N**PR -1.8662045 Non-glycosylated

112 **N**AG -1.0674394 Non-glycosylated

116 **N**TV -0.66346953 Non-glycosylated

155 **N**LV -1.0444821 Non-glycosylated

200 **N**TV -1.0299987 Non-glycosylated

**225 NNT 0.88963983 Potential Glycosylated**

226 **N**TG -1.0135771 Non-glycosylated

274 **N**LF -1.3425718 Non-glycosylated

328 **N**RL -1.0222076 Non-glycosylated

337 **N**AE -1.1197133 Non-glycosylated

352 **N**DP -1.1451453 Non-glycosylated

**373 NGT 1.2707122 Potential Glycosylated**

384 **N**AL -1.0307742 Non-glycosylated

404 **N**AM -1.3410327 Non-glycosylated

***********************************

>Ss354 Length = 412

**Potential N-Linked Glycosylated Sites:**

MHYY**N**KLKFLALASVISATSAHPTSDHYYADAESEACL**N**GKAVYVTS**N**TEH**N**SVVAIPIAR**N**GSLLV**N**YATSTATGGRGG**N**GI**N**PRGMPAGPDALFGQGS

ITIAGDYLFAV**N**AGS**N**TVTMLAIDKHDPTKVTVVGEPAELPGEFPTTVGASDKF**N**LVCVGLTGAKAGVSCASYSWYGLGPFDELRPFDLHQTTPPHGPT

**N**TVSHVFFSGDQETVFTTVKGDPAV**NN**TGFLAAYPVEHIHSSCYATPSVSHKGVISSPEGTAVLFGSTPIPDTT**N**LFATDASFGAVILGIEDYEASTLY

KTVIPGQDATCWVAICPATHTAFVTDIRM**N**RLVEMSLA**N**AEIIGEPIDLTTFS**N**DPGLTEIRSGGSFVYALSPG**N**GTTEAWITVL**N**ALTKKPVQHALLT

PLGLDR**N**AMGMAILV

------------------------------------------------

Position Residue Score Prediction

------------------------------------------------

5 **N**KL -0.77832304 Non-glycosylated

39 **N**GK -1.130584 Non-glycosylated

48 **N**TE -1.2741531 Non-glycosylated

52 **N**SV -0.83972275 Non-glycosylated

**62 NGS 1.1524071 Potential Glycosylated**

68 **N**YA -0.9795019 Non-glycosylated

81 **N**GI -1.2423291 Non-glycosylated

84 **N**PR -1.8662045 Non-glycosylated

112 **N**AG -1.0674394 Non-glycosylated

116 **N**TV -0.66346953 Non-glycosylated

155 **N**LV -1.0444821 Non-glycosylated

200 **N**TV -1.0299987 Non-glycosylated

**225 NNT 0.88963983 Potential Glycosylated**

226 **N**TG -1.0135771 Non-glycosylated

274 **N**LF -1.3425718 Non-glycosylated

328 **N**RL -1.0222076 Non-glycosylated

337 **N**AE -1.1197133 Non-glycosylated

352 **N**DP -1.1451453 Non-glycosylated

**373 NGT 1.2707122 Potential Glycosylated**

384 **N**AL -1.0307742 Non-glycosylated

404 **N**AM -1.3410327 Non-glycosylated

***********************************

>Ss104 Length = 412

**Potential N-Linked Glycosylated Sites:**

MHYY**N**KLEFLALASVISATSAHPTSDHYYADAESEACL**N**GKAVYVTS**N**TEH**N**SVVAIPIAR**N**GSLLV**N**YATSTATGGRGG**N**GI**N**PRGMPAGPDALFGQGS

ITIAGDYLFAV**N**AGS**N**TVTMLAIDKHDPTKVTVVGEPAELPGEFPTTVGASDKF**N**LVCVGLTGAKAGVSCASYSWYGLGPFDELRPFDLHQTTPPHGPT

**N**TVSHVFFSGDQETVFTTVKGDPAV**NN**TGFLAAYPVEHIHSSCYATPSVSHKGVISSPEGTAVLFGSTPIPDTT**N**LFATDASFGAVILGIEDYEASTLY

KTVIPGQDATCWVAICPATHTAFVTDIRM**N**RLVEMSLA**N**AEIIGEPIDLTTFS**N**DPGLTEIRSGGSFVYALSPG**N**GTTEAWITVL**N**ALTKKPVQHALLT

PLGLDR**N**AMGMAILV

------------------------------------------------

Position Residue Score Prediction

------------------------------------------------

5 **N**KL -0.77804637 Non-glycosylated

39 **N**GK -1.130584 Non-glycosylated

48 **N**TE -1.2741531 Non-glycosylated

52 **N**SV -0.83972275 Non-glycosylated

**62 NGS 1.1524071 Potential Glycosylated**

68 **N**YA -0.9795019 Non-glycosylated

81 **N**GI -1.2423291 Non-glycosylated

84 **N**PR -1.8662045 Non-glycosylated

112 **N**AG -1.0674394 Non-glycosylated

116 **N**TV -0.66346953 Non-glycosylated

155 **N**LV -1.0444821 Non-glycosylated

200 **N**TV -1.0299987 Non-glycosylated

**225 NNT 0.88963983 Potential Glycosylated**

226 **N**TG -1.0135771 Non-glycosylated

274 **N**LF -1.3425718 Non-glycosylated

328 **N**RL -1.0222076 Non-glycosylated

337 **N**AE -1.1197133 Non-glycosylated

352 **N**DP -1.1451453 Non-glycosylated

**373 NGT 1.2707122 Potential Glycosylated**

384 **N**AL -1.0307742 Non-glycosylated

404 **N**AM -1.3410327 Non-glycosylated

***********************************

>Ss245 Length = 412

**Potential N-Linked Glycosylated Sites:**

MHYY**N**KLKFLALASVISATSAHPTSDHYYADAESEACL**N**GKAVYVTS**N**TEH**N**SVVAIPIAR**N**GSLLV**N**YATSTATGGRGG**N**GI**N**PRGMPAGPDALFGQGS

ITIAGDYLFAV**N**AGS**N**TVTMLAIDKHDPTKVTVVGEPAELPGEFPTTVGASDKF**N**LVCVGLTGAKAGVSCASYSWYGLGPFDELRPFDLHQTTPPHGPT

**N**TVSHVFFSGDQETVFTTVKGDPAV**NN**TGFLAAYPVEHIHSSCYATPSVSHKGVISSPEGTAVLFGSTPIPDTT**N**LFATDASFGAVILGIEDYEASTLY

KTVIPGQDATCWVAICPATHTAFVTDIRM**N**RLVEMSLA**N**AEIIGEPIDLTTFS**N**DPGLTEIRSGGSFVYALSPG**N**GTTEAWITVL**N**ALTKKPVQHALLT

PLGLDR**N**AMGMAILV

------------------------------------------------

Position Residue Score Prediction

------------------------------------------------

5 **N**KL -0.77832304 Non-glycosylated

39 **N**GK -1.130584 Non-glycosylated

48 **N**TE -1.2741531 Non-glycosylated

52 **N**SV -0.83972275 Non-glycosylated

**62 NGS 1.1524071 Potential Glycosylated**

68 **N**YA -0.9795019 Non-glycosylated

81 **N**GI -1.2423291 Non-glycosylated

84 **N**PR -1.8662045 Non-glycosylated

112 **N**AG -1.0674394 Non-glycosylated

116 **N**TV -0.66346953 Non-glycosylated

155 **N**LV -1.0444821 Non-glycosylated

200 **N**TV -1.0299987 Non-glycosylated

**225 NNT 0.88963983 Potential Glycosylated**

226 **N**TG -1.0135771 Non-glycosylated

274 **N**LF -1.3425718 Non-glycosylated

328 **N**RL -1.0222076 Non-glycosylated

337 **N**AE -1.1197133 Non-glycosylated

352 **N**DP -1.1451453 Non-glycosylated

**373 NGT 1.2707122 Potential Glycosylated**

384 **N**AL -1.0307742 Non-glycosylated

404 **N**AM -1.3410327 Non-glycosylated

***********************************

>Ss250 Length = 412

**Potential N-Linked Glycosylated Sites:**

MHYY**N**KLKFLALASVISATSAHPTSDHYYADAESEACL**N**GKAVYVTS**N**TEH**N**SVVAIPIAR**N**GSLLV**N**YATSTATGGRGG**N**GI**N**PRGMPAGPDALFGQGS

ITIAGDYLFAV**N**AGS**N**TVTMLAIDKHDPTKVTVVGEPAELPGEFPTTVGASDKF**N**LVCVGLTGAKAGVSCASYSWYGLGPFDELRPFDLHQTTPPHGPT

**N**TVSHVFFSGDQETVFTTVKGDPAV**NN**TGFLAAYPVEHIHSSCYATPSVSHKGVISSPEGTAVLFGSTPIPDTT**N**LFATDASFGAVILGIEDYEASTLY

KTVIPGQDATCWVAICPATHTAFVTDIRM**N**RLVEMSLA**N**AEIIGEPIDLTTFS**N**DPGLTEIRSGGSFVYALSPG**N**GTTEAWITVL**N**ALTKKPVQHALLT

PLGLDR**N**AMGMAILV

------------------------------------------------

Position Residue Score Prediction

------------------------------------------------

5 **N**KL -0.77832304 Non-glycosylated

39 **N**GK -1.130584 Non-glycosylated

48 **N**TE -1.2741531 Non-glycosylated

52 **N**SV -0.83972275 Non-glycosylated

**62 NGS 1.1524071 Potential Glycosylated**

68 **N**YA -0.9795019 Non-glycosylated

81 **N**GI -1.2423291 Non-glycosylated

84 **N**PR -1.8662045 Non-glycosylated

112 **N**AG -1.0674394 Non-glycosylated

116 **N**TV -0.66346953 Non-glycosylated

155 **N**LV -1.0444821 Non-glycosylated

200 **N**TV -1.0299987 Non-glycosylated

**225 NNT 0.88963983 Potential Glycosylated**

226 **N**TG -1.0135771 Non-glycosylated

274 **N**LF -1.3425718 Non-glycosylated

328 **N**RL -1.0222076 Non-glycosylated

337 **N**AE -1.1197133 Non-glycosylated

352 **N**DP -1.1451453 Non-glycosylated

**373 NGT 1.2707122 Potential Glycosylated**

384 **N**AL -1.0307742 Non-glycosylated

404 **N**AM -1.3410327 Non-glycosylated

***********************************

>Ss52 Length = 412

**Potential N-Linked Glycosylated Sites:**

MHYY**N**KLKFLALASVISATSAHPTSDHYYADAESEACL**N**GKAVYVTS**N**TEH**N**SVVAIPIAR**N**GSLLV**N**YATSTATGGRGG**N**GI**N**PRGMPAGPDALFGQGS

ITIAGDYLFAV**N**AGS**N**TVTMLAIDKHDPTKVTVVGEPAELPGEFPTTVGASDKF**N**LVCVGLTGAKAGVSCASYSWYGLGPFDELRPFDLHQTTPPHGPT

**N**TVSHVFFSGDQETVFTTVKGDPAV**NN**TGFLAAYPVEHIHSSCYATPSVSHKGVISSPEGTAVLFGSTPIPDTT**N**LFATDASFGAVILGIEDYEASTLY

KTVIPGQDATCWVAICPATHTAFVTDIRM**N**RLVEMSLA**N**AEIIGEPIDLTTFS**N**DPGLTEIRSGGSFVYALSPG**N**GTTEAWITVL**N**ALTKKPVQHALLT

PLGLDR**N**AMGMAILV

------------------------------------------------

Position Residue Score Prediction

------------------------------------------------

5 **N**KL -0.77832304 Non-glycosylated

39 **N**GK -1.130584 Non-glycosylated

48 **N**TE -1.2741531 Non-glycosylated

52 **N**SV -0.83972275 Non-glycosylated

**62 NGS 1.1524071 Potential Glycosylated**

68 **N**YA -0.9795019 Non-glycosylated

81 **N**GI -1.2423291 Non-glycosylated

84 **N**PR -1.8662045 Non-glycosylated

112 **N**AG -1.0674394 Non-glycosylated

116 **N**TV -0.66346953 Non-glycosylated

155 **N**LV -1.0444821 Non-glycosylated

200 **N**TV -1.0299987 Non-glycosylated

**225 NNT 0.88963983 Potential Glycosylated**

226 **N**TG -1.0135771 Non-glycosylated

274 **N**LF -1.3425718 Non-glycosylated

328 **N**RL -1.0222076 Non-glycosylated

337 **N**AE -1.1197133 Non-glycosylated

352 **N**DP -1.1451453 Non-glycosylated

**373 NGT 1.2707122 Potential Glycosylated**

384 **N**AL -1.0307742 Non-glycosylated

404 **N**AM -1.3410327 Non-glycosylated

***********************************

>Ss54 Length = 412

**Potential N-Linked Glycosylated Sites:**

MHYY**N**KLKFLALASVISATSAHPTSDHYYADAESEACL**N**GKAVYVTS**N**TEH**N**SVVAIPIAR**N**GSLLV**N**YATSTATGGRGG**N**GI**N**PRGMPAGPDALFGQGS

ITIAGDYLFAV**N**AGS**N**TVTMLAIDKHDPTKVTVVGEPAELPGEFPTTVGASDKF**N**LVCVGLTGAKAGVSCASYSWYGLGPFDELRPFDLHQTTPPHGPT

**N**TVSHVFFSGDQETVFTTVKGDPAV**NN**TGFLAAYPVEHIHSSCYATPSVSHKGVISSPEGTAVLFGSTPIPDTT**N**LFATDASFGAVILGIEDYEASTLY

KTVIPGQDATCWVAICPATHTAFVTDIRM**N**RLVEMSLA**N**AEIIGEPIDLTTFS**N**DPGLTEIRSGGSFVYALSPG**N**GTTEAWITVL**N**ALTKKPVQHALLT

PLGLDR**N**AMGMAILV

------------------------------------------------

Position Residue Score Prediction

------------------------------------------------

5 **N**KL -0.77832304 Non-glycosylated

39 **N**GK -1.130584 Non-glycosylated

48 **N**TE -1.2741531 Non-glycosylated

52 **N**SV -0.83972275 Non-glycosylated

**62 NGS 1.1524071 Potential Glycosylated**

68 **N**YA -0.9795019 Non-glycosylated

81 **N**GI -1.2423291 Non-glycosylated

84 **N**PR -1.8662045 Non-glycosylated

112 **N**AG -1.0674394 Non-glycosylated

116 **N**TV -0.66346953 Non-glycosylated

155 **N**LV -1.0444821 Non-glycosylated

200 **N**TV -1.0299987 Non-glycosylated

**225 NNT 0.88963983 Potential Glycosylated**

226 **N**TG -1.0135771 Non-glycosylated

274 **N**LF -1.3425718 Non-glycosylated

328 **N**RL -1.0222076 Non-glycosylated

337 **N**AE -1.1197133 Non-glycosylated

352 **N**DP -1.1451453 Non-glycosylated

**373 NGT 1.2707122 Potential Glycosylated**

384 **N**AL -1.0307742 Non-glycosylated

404 **N**AM -1.3410327 Non-glycosylated

***********************************

>Ss178 Length = 412

**Potential N-Linked Glycosylated Sites:**

MHYY**N**KLKFLALASVITATSAHPTSDHYYADAESEACL**N**GKAVYVTS**N**TEH**N**SVVAIPIAR**N**GSLLV**N**YATSTATGGRGG**N**GI**N**PRGMPAGPDALFGQGS

ITIAGDYLFAV**N**AGS**N**TVTMLAIDKHDPTKVTVVGEPAELPGEFPTTVGASDKF**N**LVCVGLTGAKAGVSCASYSWYGLGPFDELRPFDLHQTTPPHGPT

**N**TVSHVFFSGDQETVFTTVKGDPAV**NN**TGFLAAYPVEHIHSSCYATPSVSHKGVISSPEGTAVLFGSTPIPDTT**N**LFATDASFGAVILGIEDYEASTLY

KTVIPGQDATCWVAICPATHTAFVTDIRM**N**RLVEMSLA**N**AEIIGEPIDLTTFS**N**DPGLTEIRSGGSFVYALSPG**N**GTTEAWITVL**N**ALTKKPVQHALLT

PLGLDR**N**AMGMAILV

------------------------------------------------

Position Residue Score Prediction

------------------------------------------------

5 **N**KL -0.77832304 Non-glycosylated

39 **N**GK -1.130584 Non-glycosylated

48 **N**TE -1.2741531 Non-glycosylated

52 **N**SV -0.83972275 Non-glycosylated

**62 NGS 1.1524071 Potential Glycosylated**

68 **N**YA -0.9795019 Non-glycosylated

81 **N**GI -1.2423291 Non-glycosylated

84 **N**PR -1.8662045 Non-glycosylated

112 **N**AG -1.0674394 Non-glycosylated

116 **N**TV -0.66346953 Non-glycosylated

155 **N**LV -1.0444821 Non-glycosylated

200 **N**TV -1.0299987 Non-glycosylated

**225 NNT 0.88963983 Potential Glycosylated**

226 **N**TG -1.0135771 Non-glycosylated

274 **N**LF -1.3425718 Non-glycosylated

328 **N**RL -1.0222076 Non-glycosylated

337 **N**AE -1.1197133 Non-glycosylated

352 **N**DP -1.1451453 Non-glycosylated

**373 NGT 1.2707122 Potential Glycosylated**

384 **N**AL -1.0307742 Non-glycosylated

404 **N**AM -1.3410327 Non-glycosylated

***********************************

>Ss177 Length = 412

**Potential N-Linked Glycosylated Sites:**

MHYY**N**KLKFLALASVITATSAHPTSDHYYADAESEACL**N**GKAVYVTS**N**TEH**N**SVVAIPIAR**N**GSLLV**N**YATSTATGGRGG**N**GI**N**PRGMPAGPDALFGQGS

ITIAGDYLFAV**N**AGS**N**TVTMLAIDKHDPTKVTVVGEPAELPGEFPTTVGASDKF**N**LVCVGLTGAKAGVSCASYSWYGLGPFDELRPFDLHQTTPPHGPT

**N**TVSHVFFSGDQETVFTTVKGDPAV**NN**TGFLAAYPVEHIHSSCYATPSVSHKGVISSPEGTAVLFGSTPIPDTT**N**LFATDASFGAVILGIEDYEASTLY

KTVIPGQDATCWVAICPATHTAFVTDIRM**N**RLVEMSLA**N**AEIIGEPIDLTTFS**N**DPGLTEIRSGGSFVYALSPG**N**GTTEAWITVL**N**ALTKKPVQHALLT

PLGLDR**N**AMGMAILV

------------------------------------------------

Position Residue Score Prediction

------------------------------------------------

5 **N**KL -0.77832304 Non-glycosylated

39 **N**GK -1.130584 Non-glycosylated

48 **N**TE -1.2741531 Non-glycosylated

52 **N**SV -0.83972275 Non-glycosylated

**62 NGS 1.1524071 Potential Glycosylated**

68 **N**YA -0.9795019 Non-glycosylated

81 **N**GI -1.2423291 Non-glycosylated

84 **N**PR -1.8662045 Non-glycosylated

112 **N**AG -1.0674394 Non-glycosylated

116 **N**TV -0.66346953 Non-glycosylated

155 **N**LV -1.0444821 Non-glycosylated

200 **N**TV -1.0299987 Non-glycosylated

**225 NNT 0.88963983 Potential Glycosylated**

226 **N**TG -1.0135771 Non-glycosylated

274 **N**LF -1.3425718 Non-glycosylated

328 **N**RL -1.0222076 Non-glycosylated

337 **N**AE -1.1197133 Non-glycosylated

352 **N**DP -1.1451453 Non-glycosylated

**373 NGT 1.2707122 Potential Glycosylated**

384 **N**AL -1.0307742 Non-glycosylated

404 **N**AM -1.3410327 Non-glycosylated

***********************************

>Ss252 Length = 412

**Potential N-Linked Glycosylated Sites:**

MHYY**N**KLKFLALASVISATSAHPTSDHYYADAESEACL**N**GKAVYVTS**N**TEH**N**SVVAIPIAR**N**GSLLV**N**YATSTATGGRGG**N**GI**N**PRGMPAGPDALFGQGS

ITIAGDYLFAV**N**AGS**N**TVTMLAIDKHDPTKVTVVGEPAELPGEFPTTVGASDKF**N**LVCVGLTGAKAGVSCASYSWYGLGPFDELRPFDLHQTTPPHGPT

**N**TVSHVFFSGDQETVFTTVKGDPAV**NN**TGFLAAYPVEHIHSSCYATPSVSHKGVISSPEGTGVLFGSTPIPDTT**N**LFATDASFGAVILGIEDYEASTLY

KTVIPGQDATCWVAICPATHTAFVTDIRM**N**RLVEMSLA**N**AEIIGEPIDLTTFS**N**DPGLTEIRSGGSFVYALSPG**N**GTTEAWITVL**N**ALTKKPVQHALLT

PLGLDR**N**AMGMAILV

------------------------------------------------

Position Residue Score Prediction

------------------------------------------------

5 **N**KL -0.77832304 Non-glycosylated

39 **N**GK -1.130584 Non-glycosylated

48 **N**TE -1.2741531 Non-glycosylated

52 **N**SV -0.83972275 Non-glycosylated

**62 NGS 1.1524071 Potential Glycosylated**

68 **N**YA -0.9795019 Non-glycosylated

81 **N**GI -1.2423291 Non-glycosylated

84 **N**PR -1.8662045 Non-glycosylated

112 **N**AG -1.0674394 Non-glycosylated

116 **N**TV -0.66346953 Non-glycosylated

155 **N**LV -1.0444821 Non-glycosylated

200 **N**TV -1.0299987 Non-glycosylated

**225 NNT 0.88963983 Potential Glycosylated**

226 **N**TG -1.0135771 Non-glycosylated

274 **N**LF -1.3425718 Non-glycosylated

328 **N**RL -1.0222076 Non-glycosylated

337 **N**AE -1.1197133 Non-glycosylated

352 **N**DP -1.1451453 Non-glycosylated

**373 NGT 1.2707122 Potential Glycosylated**

384 **N**AL -1.0307742 Non-glycosylated

404 **N**AM -1.3410327 Non-glycosylated

***********************************

>Ss038 Length = 412

**Potential N-Linked Glycosylated Sites:**

MHYY**N**KLKFLALASVISATSAHPTSDHYYADAESEACL**N**GKAVYVTS**N**TEH**N**SVVAIPIAR**N**GSLLV**N**YATSTATGGRGG**N**GI**N**PRGMPAGPDALFGQGS

ITIAGDYLFAV**N**AGS**N**TVTMLAIDKHDPTKVTVVGEPAELPGEFPTTVGASDKF**N**LVCVGLTGAKAGVSCASYSWYGLGPFDELRPFDLHQTTPPHGPT

**N**TVSHVFFSGDQETVFTTVKGDPAV**NN**TGFLAAYPVEHIHSSCYATPSVSHKGVISSPEGTAVLFGSTPIPDTT**N**LFATDASFGAFILGIEDYEASTLY

KTVIPGQDATCWVAICPATHTAFVTDIRM**N**RLVEMSLA**N**AEIIGEPIDLTTFS**N**DPGLTEIRSGGSFVYALSPG**N**GTTEAWITVL**N**ALTKKPVQHALLT

PLGLDR**N**AMGMAILV

------------------------------------------------

Position Residue Score Prediction

------------------------------------------------

5 **N**KL -0.77832304 Non-glycosylated

39 **N**GK -1.130584 Non-glycosylated

48 **N**TE -1.2741531 Non-glycosylated

52 **N**SV -0.83972275 Non-glycosylated

**62 NGS 1.1524071 Potential Glycosylated**

68 **N**YA -0.9795019 Non-glycosylated

81 **N**GI -1.2423291 Non-glycosylated

84 **N**PR -1.8662045 Non-glycosylated

112 **N**AG -1.0674394 Non-glycosylated

116 **N**TV -0.66346953 Non-glycosylated

155 **N**LV -1.0444821 Non-glycosylated

200 **N**TV -1.0299987 Non-glycosylated

**225 NNT 0.88963983 Potential Glycosylated**

226 **N**TG -1.0135771 Non-glycosylated

274 **N**LF -1.3425718 Non-glycosylated

328 **N**RL -1.0222076 Non-glycosylated

337 **N**AE -1.1197133 Non-glycosylated

352 **N**DP -1.1451453 Non-glycosylated

**373 NGT 1.2707122 Potential Glycosylated**

384 **N**AL -1.0307742 Non-glycosylated

404 **N**AM -1.3410327 Non-glycosylated

***********************************

>Ss333 Length = 412

**Potential N-Linked Glycosylated Sites:**

MHYY**N**KLKFLALASVISATSAHPTSDHYYADAESEACL**N**GKAVYVTS**N**TEH**N**SVVAIPIAR**N**GSLLV**N**YATSTATGGRGG**N**GI**N**PRGMPAGPDALFGQGS

ITIAGDYLFAV**N**AGS**N**TVTMLAIDKHDPTKVTVVGEPAELPGEFPTTVGASDKF**N**LVCVGLTGAKAGVSCASYSWYGLGPFDELRPFDLHQTTPPHGPT

**N**TVSHVFFSGDQETVFTTVKGDPAV**NN**TGFLAAYPVEHIHSSCYATPSVSHKGVISSPEGTAVLFGSTPIPDTT**N**LFATDASFGAVILGIEDYEASTLY

KTVIPGQDATCWVAICPATHTAFVTDIRM**N**RLVEMSLA**N**AEIIGEPIDLTTFS**N**DPGLTEIRSGGSFVYALSPG**N**GTTEAWITVL**N**ALTKKPVQHALLT

PLGLDR**N**AMGMAILV

------------------------------------------------

Position Residue Score Prediction

------------------------------------------------

5 **N**KL -0.77832304 Non-glycosylated

39 **N**GK -1.130584 Non-glycosylated

48 **N**TE -1.2741531 Non-glycosylated

52 **N**SV -0.83972275 Non-glycosylated

**62 NGS 1.1524071 Potential Glycosylated**

68 **N**YA -0.9795019 Non-glycosylated

81 **N**GI -1.2423291 Non-glycosylated

84 **N**PR -1.8662045 Non-glycosylated

112 **N**AG -1.0674394 Non-glycosylated

116 **N**TV -0.66346953 Non-glycosylated

155 **N**LV -1.0444821 Non-glycosylated

200 **N**TV -1.0299987 Non-glycosylated

**225 NNT 0.88963983 Potential Glycosylated**

226 **N**TG -1.0135771 Non-glycosylated

274 **N**LF -1.3425718 Non-glycosylated

328 **N**RL -1.0222076 Non-glycosylated

337 **N**AE -1.1197133 Non-glycosylated

352 **N**DP -1.1451453 Non-glycosylated

**373 NGT 1.2707122 Potential Glycosylated**

384 **N**AL -1.0307742 Non-glycosylated

404 **N**AM -1.3410327 Non-glycosylated

***********************************

>Ss248 Length = 412

**Potential N-Linked Glycosylated Sites:**

MHYY**N**KLKFLALASVISATSAHPTSDHYYADAESEACL**N**GKAVYVTS**N**TEH**N**SVVAIPIAR**N**GSLLV**N**YATSTATGGRGG**N**GI**N**PRGMPAGPDALFGQGS

ITIAGDYLFAV**N**AGS**N**TVTMLAIDKHDPTKVTVVGEPAELPGEFPTTVGASDKF**N**LVCVGLTGAKAGVSCASYSWYGLGPFDELRPFDLHQTTPPHGPT

**N**TVSHVFFSGDQETVFTTVKGDPAV**NN**TGFLAAYPVEHIHSSCYATPSVSHKGVISSPEGTAVLFGSTPIPDTT**N**LFATDASFGAVILGIEDYEASTLY

KTVIPGQDATCWVAICPATHTAFVTDIRM**N**RLVEMSLA**N**AEIIGEPIDLTTFS**N**DPGLTEIRSGGSFVYALSPG**N**GTTEAWITVL**N**ALTK**N**PVQHGLLT

PLGLDR**N**AMGMAILV

------------------------------------------------

Position Residue Score Prediction

------------------------------------------------

5 **N**KL -0.77832304 Non-glycosylated

39 **N**GK -1.130584 Non-glycosylated

48 **N**TE -1.2741531 Non-glycosylated

52 **N**SV -0.83972275 Non-glycosylated

**62 NGS 1.1524071 Potential Glycosylated**

68 **N**YA -0.9795019 Non-glycosylated

81 **N**GI -1.2423291 Non-glycosylated

84 **N**PR -1.8662045 Non-glycosylated

112 **N**AG -1.0674394 Non-glycosylated

116 **N**TV -0.66346953 Non-glycosylated

155 **N**LV -1.0444821 Non-glycosylated

200 **N**TV -1.0299987 Non-glycosylated

**225 NNT 0.88963983 Potential Glycosylated**

226 **N**TG -1.0135771 Non-glycosylated

274 **N**LF -1.3425718 Non-glycosylated

328 **N**RL -1.0222076 Non-glycosylated

337 **N**AE -1.1197133 Non-glycosylated

352 **N**DP -1.1451453 Non-glycosylated

**373 NGT 1.2707122 Potential Glycosylated**

384 **N**AL -1.0622605 Non-glycosylated

389 **N**PV -1.0347753 Non-glycosylated

404 **N**AM -1.3497988 Non-glycosylated

***********************************

>Ss246 Length = 412

**Potential N-Linked Glycosylated Sites:**

MHYY**N**KLKFLALASVISATSAHPTSDHYYADAESEACL**N**GKAVYVTS**N**TEH**N**SVVAIPIAR**N**GSLLV**N**YATSTATGGRGG**N**GI**N**PRGMPAGPDALFGQGS

ITIAGDYLFAV**N**AGS**N**TVTMLAIDKHDPTKVTVVGEPAELPGEFPTTVGASDKF**N**LVCVGLTGAKAGVSCASYSWYGLGPFDELRPFDLHQTTPPHGPT

**N**TVSHVFFSGDQETVFTTVKGDPAV**NN**TGFLAAYPVEHIHSSCYATPSVSHKGVISSPEGTAVLFGSTPIPDTT**N**LFATDASFGAVILGIEDYEASTLY

KTVIPGQDATCWVAICPATHTAFVTDIRM**N**RLVEMSLA**N**AEIIGEPIDLTTFS**N**DPGLTEIRSGGSFVYALSPG**N**GTTEAWITVL**N**ALTK**N**PAQHALLT

PLGLDR**N**AMGMAILV

------------------------------------------------

Position Residue Score Prediction

------------------------------------------------

5 **N**KL -0.77832304 Non-glycosylated

39 **N**GK -1.130584 Non-glycosylated

48 **N**TE -1.2741531 Non-glycosylated

52 **N**SV -0.83972275 Non-glycosylated

**62 NGS 1.1524071 Potential Glycosylated**

68 **N**YA -0.9795019 Non-glycosylated

81 **N**GI -1.2423291 Non-glycosylated

84 **N**PR -1.8662045 Non-glycosylated

112 **N**AG -1.0674394 Non-glycosylated

116 **N**TV -0.66346953 Non-glycosylated

155 **N**LV -1.0444821 Non-glycosylated

200 **N**TV -1.0299987 Non-glycosylated

**225 NNT 0.88963983 Potential Glycosylated**

226 **N**TG -1.0135771 Non-glycosylated

274 **N**LF -1.3425718 Non-glycosylated

328 **N**RL -1.0222076 Non-glycosylated

337 **N**AE -1.1197133 Non-glycosylated

352 **N**DP -1.1451453 Non-glycosylated

**373 NGT 1.2707122 Potential Glycosylated**

384 **N**AL -1.0062079 Non-glycosylated

389 **N**PA -1.0692624 Non-glycosylated

404 **N**AM -1.3410327 Non-glycosylated

***********************************

>Ss254 Length = 412

**Potential N-Linked Glycosylated Sites:**

MHYY**N**KLKFLALASVISATSAHPTSDHYYADAESEACL**N**GKAVYVTS**N**TEH**N**SVVAIPIAR**N**GSLLV**N**YATSTATGGRGG**N**GI**N**PRGMPAGPDALFGQGS

ITIAGDYLFAV**N**AGS**N**TVTMLAIDKHDPTKVTVVGEPAELPGEFPTTVGASDKF**N**LVCVGLTGAKAGVSCASYSWYGLGPFDELRPFDLHQTTPPHGPT

**N**TVSHVFFSGDQETVFTTVKGDPAV**NN**TGFLAAYPVEHIHSSCYATPSVSHKGVISSPEGTAVLFGSTPIPDTT**N**LFATDASFGAVILGIEDYEASTLY

KTVIPGQDATCWVAICPATHTAFVTDIRM**N**RLVEMSLA**N**AEIIGEPIDLTTFS**N**DPGLTEIRSGGSFVYALSPG**N**GTTEAWITVL**N**ALTK**N**PVQHALLT

PLGLDR**N**AMGMAILV

------------------------------------------------

Position Residue Score Prediction

------------------------------------------------

5 **N**KL -0.77832304 Non-glycosylated

39 **N**GK -1.130584 Non-glycosylated

48 **N**TE -1.2741531 Non-glycosylated

52 **N**SV -0.83972275 Non-glycosylated

**62 NGS 1.1524071 Potential Glycosylated**

68 **N**YA -0.9795019 Non-glycosylated

81 **N**GI -1.2423291 Non-glycosylated

84 **N**PR -1.8662045 Non-glycosylated

112 **N**AG -1.0674394 Non-glycosylated

116 **N**TV -0.66346953 Non-glycosylated

155 **N**LV -1.0444821 Non-glycosylated

200 **N**TV -1.0299987 Non-glycosylated

**225 NNT 0.88963983 Potential Glycosylated**

226 **N**TG -1.0135771 Non-glycosylated

274 **N**LF -1.3425718 Non-glycosylated

328 **N**RL -1.0222076 Non-glycosylated

337 **N**AE -1.1197133 Non-glycosylated

352 **N**DP -1.1451453 Non-glycosylated

**373 NGT 1.2707122 Potential Glycosylated**

384 **N**AL -0.96353409 Non-glycosylated

389 **N**PV -1.0464722 Non-glycosylated

404 **N**AM -1.3410327 Non-glycosylated

***********************************

>Ss044 Length = 412

**Potential N-Linked Glycosylated Sites:**

MHYY**N**KLKFLALASVISATSAHPTSDHYYADAESEACL**N**GKAVYVTS**N**TEH**N**SVVAIPIAR**N**GSLLV**N**YATSTATGGRGG**N**GI**N**PRGMPAGPDALFGQGS

ITIAGDYLFAV**N**AGS**N**TVTMLAIDKHDPTKVTVVGEPAELPGEFPTTVGASDKF**N**LVCVGLTGAKAGVSCASYSWYGLGPFDELRPFDLHQTTPPHGPT

**N**TVSHVFFSGDQETVFTTVKGDPAV**NN**TGFLAAYPVEHIHSSCYATPSVSHKGVISSPEGTAVLFGSTPIPDTT**N**LFATDASFGAVILGIEDYEASTLY

KTVIPGQDATCWVAICPATHTAFVTDIRM**N**RLVEMSLA**N**AEIIGEPIDLTTFS**N**DPGLTEIRSGGSFVYALSPG**N**GTTEAWITVL**N**ALTK**N**PVQHALLT

PLGLDR**N**AMGMAILV

------------------------------------------------

Position Residue Score Prediction

------------------------------------------------

5 **N**KL -0.77832304 Non-glycosylated

39 **N**GK -1.130584 Non-glycosylated

48 **N**TE -1.2741531 Non-glycosylated

52 **N**SV -0.83972275 Non-glycosylated

**62 NGS 1.1524071 Potential Glycosylated**

68 **N**YA -0.9795019 Non-glycosylated

81 **N**GI -1.2423291 Non-glycosylated

84 **N**PR -1.8662045 Non-glycosylated

112 **N**AG -1.0674394 Non-glycosylated

116 **N**TV -0.66346953 Non-glycosylated

155 **N**LV -1.0444821 Non-glycosylated

200 **N**TV -1.0299987 Non-glycosylated

**225 NNT 0.88963983 Potential Glycosylated**

226 **N**TG -1.0135771 Non-glycosylated

274 **N**LF -1.3425718 Non-glycosylated

328 **N**RL -1.0222076 Non-glycosylated

337 **N**AE -1.1197133 Non-glycosylated

352 **N**DP -1.1451453 Non-glycosylated

**373 NGT 1.2707122 Potential Glycosylated**

384 **N**AL -0.96353409 Non-glycosylated

389 **N**PV -1.0464722 Non-glycosylated

404 **N**AM -1.3410327 Non-glycosylated

***********************************

>Ss057 Length = 412

**Potential N-Linked Glycosylated Sites:**

MHYY**N**KLKFLALASVISATSAHPTSDHYYADAESEACL**N**GKAVYVTS**N**TEH**N**SVVAIPIAR**N**GSLLV**N**YATSTATGGRGG**N**GI**N**PRGMPAGPDALFGQGS

ITIAGDYLFAV**N**AGS**N**TVTMLAIDKHDPTKVTVVGEPAELPGEFPTTVGASDKF**N**LVCVGLTGAKAGVSCASYSWYGLGPFDELRPFDLHQTTPPHGPT

**N**TVSHVFFSGDQETVFTTVKGDPAV**NN**TGFLAAYPVEHIHSSCYATPSVSHKGVISSPEGTAVLFGSTPIPDTT**N**LFATDASFGAVILGIEDYEASTLY

KTVIPGQDATCWVAICPATHTAFVTDIRM**N**RLVEMSLA**N**AEIIGEPIDLTTFS**N**DPGLTEIRSGGSFVYALSPG**N**GTTEAWITVL**N**ALTK**N**LVQHALLT

PLGLDR**N**AMGMAILV

------------------------------------------------

Position Residue Score Prediction

------------------------------------------------

5 **N**KL -0.77832304 Non-glycosylated

39 **N**GK -1.130584 Non-glycosylated

48 **N**TE -1.2741531 Non-glycosylated

52 **N**SV -0.83972275 Non-glycosylated

**62 NGS 1.1524071 Potential Glycosylated**

68 **N**YA -0.9795019 Non-glycosylated

81 **N**GI -1.2423291 Non-glycosylated

84 **N**PR -1.8662045 Non-glycosylated

112 **N**AG -1.0674394 Non-glycosylated

116 **N**TV -0.66346953 Non-glycosylated

155 **N**LV -1.0444821 Non-glycosylated

200 **N**TV -1.0299987 Non-glycosylated

**225 NNT 0.88963983 Potential Glycosylated**

226 **N**TG -1.0135771 Non-glycosylated

274 **N**LF -1.3425718 Non-glycosylated

328 **N**RL -1.0222076 Non-glycosylated

337 **N**AE -1.1197133 Non-glycosylated

352 **N**DP -1.1451453 Non-glycosylated

**373 NGT 1.2707122 Potential Glycosylated**

384 **N**AL -1.0302355 Non-glycosylated

389 **N**LV -0.50739378 Non-glycosylated

404 **N**AM -1.3410327 Non-glycosylated

***********************************

>Ss360 Length = 412

**Potential N-Linked Glycosylated Sites:**

MHYY**N**KLKFLALASVISATSAHPTSDHYYADAESEACL**N**GKAVYVTS**N**TEH**N**SVVAIPIAR**N**GSLLV**N**YATSTATGGRGG**N**GI**N**PRGMPAGPDALFGQGS

ITIAGDYLFAV**N**AGS**N**TVTMLAIDKHDPTKVTVVGEPAELPGEFPTTVGASDKF**N**LVCVGLTGAKAGVSCASYSWYGLGPFDELRPFDLHQTTPPHGPT

**N**TVSHVFFSGDQETVFTTVKGDPAV**NN**TGFLAAYPVEHIHSSCYATPSVSHKGVISSPEGTAVLFGSTPIPDTT**N**LFATDASFGAVILGIEDYEASTLY

KTVIPGQDATCWVAICPATHTAFVTDIRM**N**RLVEMSLA**N**AEIIGEPIDLTTFS**N**DPGLTEIRSGGSFVYALSPG**N**GTTEAWITVL**N**ALTKKPVQHALLT

PLGLDR**N**AMGMAILV

------------------------------------------------

Position Residue Score Prediction

------------------------------------------------

5 **N**KL -0.77832304 Non-glycosylated

39 **N**GK -1.130584 Non-glycosylated

48 **N**TE -1.2741531 Non-glycosylated

52 **N**SV -0.83972275 Non-glycosylated

**62 NGS 1.1524071 Potential Glycosylated**

68 **N**YA -0.9795019 Non-glycosylated

81 **N**GI -1.2423291 Non-glycosylated

84 **N**PR -1.8662045 Non-glycosylated

112 **N**AG -1.0674394 Non-glycosylated

116 **N**TV -0.66346953 Non-glycosylated

155 **N**LV -1.0444821 Non-glycosylated

200 **N**TV -1.0299987 Non-glycosylated

**225 NNT 0.88963983 Potential Glycosylated**

226 **N**TG -1.0135771 Non-glycosylated

274 **N**LF -1.3425718 Non-glycosylated

328 **N**RL -1.0222076 Non-glycosylated

337 **N**AE -1.1197133 Non-glycosylated

352 **N**DP -1.1451453 Non-glycosylated

**373 NGT 1.2707122 Potential Glycosylated**

384 **N**AL -1.0307742 Non-glycosylated

404 **N**AM -1.3410327 Non-glycosylated

***********************************

>Ss338 Length = 412

**Potential N-Linked Glycosylated Sites:**

MHYY**N**KLKFLALASVISATSAHPTSDHYYADAESEACL**N**GKAVYVTS**N**TEH**N**SVVAIPIAR**N**GSLLV**N**YATSTATGGRGG**N**GI**N**PRGMPAGPDALFGQGS

ITIAGDYLFAV**N**AGS**N**TVTMLAIDKHDPTKVTVVGEPAELPGEFPTTVGASDKF**N**LVCVGLTGAKAGVSCASYSWYGLGPFDELRPFDLHQTTPPHGPT

**N**TVSHVFFSGDQETVFTTVKGDPAV**NN**TGFLAAYPVEHIHSSCYATPSVSHKGVISSPEGTAVLFGSTPIPDTT**N**LFATDASFGAVILGIEDYEASTLY

KTVIPGQDATCWVAICPATHTAFVTDIRM**N**RLVEMSLA**N**AEIIGEPIDLTTFS**N**DPGLTEIRSGGSFVYALSPG**N**GTTEAWITVLYALTKKPVQHALLT

PLGLDRYAMGMAILV

------------------------------------------------

Position Residue Score Prediction

------------------------------------------------

5 **N**KL -0.77832304 Non-glycosylated

39 **N**GK -1.130584 Non-glycosylated

48 **N**TE -1.2741531 Non-glycosylated

52 **N**SV -0.83972275 Non-glycosylated

**62 NGS 1.1524071 Potential Glycosylated**

68 **N**YA -0.9795019 Non-glycosylated

81 **N**GI -1.2423291 Non-glycosylated

84 **N**PR -1.8662045 Non-glycosylated

112 **N**AG -1.0674394 Non-glycosylated

116 **N**TV -0.66346953 Non-glycosylated

155 **N**LV -1.0444821 Non-glycosylated

200 **N**TV -1.0299987 Non-glycosylated

**225 NNT 0.88963983 Potential Glycosylated**

226 **N**TG -1.0135771 Non-glycosylated

274 **N**LF -1.3425718 Non-glycosylated

328 **N**RL -1.0222076 Non-glycosylated

337 **N**AE -1.1197133 Non-glycosylated

352 **N**DP -1.1451453 Non-glycosylated

**373 NGT 1.2707122 Potential Glycosylated**

***********************************

>Ss10 Length = 412

**Potential N-Linked Glycosylated Sites:**

MHYY**N**KLKFLALASVITATSAHPTSDHYYADAESEACL**N**GKAVYVTS**N**TEH**N**SVVAIPIAR**N**GSLLV**N**YATSTATGGRGG**N**GI**N**PRGMPAGPDALFGQGS

ITIAGDYLFAV**N**AGS**N**TVTMLAIDKHDPTKVTVVGEPPELPGEFPTTVGASDKF**N**LVCVGLTGAKAGVSCASYSWYGLGPFDELRPFDLHQTTPPHGPT

**N**TVSHVFFSGDQETVFTTVKGDPAV**NN**TGFLAAYPVEHIHSSCYATPSVSHKAVISSPEGTAVLFGSTPIPDTT**N**LFATDASFGAVILGIEDYEASTLY

KTVIPGQDATCWVAICPATHTAFVTDIRM**N**RLVEMSLA**N**AEIIGEPIDLTTFS**N**DPGLTEIRSGGSFVYALSPG**N**GTTEAWITVL**N**ALTKKPLQHALLT

PLGLDR**N**AMGMAILV

------------------------------------------------

Position Residue Score Prediction

------------------------------------------------

5 **N**KL -0.77832304 Non-glycosylated

39 **N**GK -1.130584 Non-glycosylated

48 **N**TE -1.2741531 Non-glycosylated

52 **N**SV -0.83972275 Non-glycosylated

**62 NGS 1.1524071 Potential Glycosylated**

68 **N**YA -0.9795019 Non-glycosylated

81 **N**GI -1.2423291 Non-glycosylated

84 **N**PR -1.8662045 Non-glycosylated

112 **N**AG -1.0674394 Non-glycosylated

116 **N**TV -0.66346953 Non-glycosylated

155 **N**LV -1.0444821 Non-glycosylated

200 **N**TV -1.0299987 Non-glycosylated

**225 NNT 0.88963983 Potential Glycosylated**

226 **N**TG -1.0135771 Non-glycosylated

274 **N**LF -1.3425718 Non-glycosylated

328 **N**RL -1.0222076 Non-glycosylated

337 **N**AE -1.1197133 Non-glycosylated

352 **N**DP -1.1451453 Non-glycosylated

**373 NGT 1.2707122 Potential Glycosylated**

384 **N**AL -1.0555029 Non-glycosylated

404 **N**AM -1.3410327 Non-glycosylated

***********************************

>Ss332 Length = 412

**Potential N-Linked Glycosylated Sites:**

MHYY**N**KLKFLALASVISATSAHPTSDHYYADAESEACL**N**GKAVYVTS**N**TEH**N**SVVAIPIAR**N**GSLLV**N**YATSTATGGRGG**N**GI**N**PRGMPAGPDALFGQGS

ITIAGDYLFAV**N**AGS**N**TVTMLAIDKHDPTKVTVVGEPAELPGEFPTTVGASDKF**N**LVCVGLTGAKAGVSCASYSWYGLGPFDELRPFDLHQTTPPHGPT

**N**TVSHVFFSGDQETVFTTVKGDPAV**NN**TGFLAAYPVEHIHSSCYATPSVSHKGVISSPEGTAVLFGSTPIPDTT**N**LFATDASFGAVILGIEDYEASTLY

KTVIPGQDATCWVAICPATHTAFVTDIRM**N**RLVEMSLA**N**AEIIGEPIDLTTFS**N**DPGLTEIRSGGSFVYALSPG**N**GTTEAWITVL**N**ALTK**N**PQQHALLT

PLGLDR**N**AMGMAILV

------------------------------------------------

Position Residue Score Prediction

------------------------------------------------

5 **N**KL -0.77832304 Non-glycosylated

39 **N**GK -1.130584 Non-glycosylated

48 **N**TE -1.2741531 Non-glycosylated

52 **N**SV -0.83972275 Non-glycosylated

**62 NGS 1.1524071 Potential Glycosylated**

68 **N**YA -0.9795019 Non-glycosylated

81 **N**GI -1.2423291 Non-glycosylated

84 **N**PR -1.8662045 Non-glycosylated

112 **N**AG -1.0674394 Non-glycosylated

116 **N**TV -0.66346953 Non-glycosylated

155 **N**LV -1.0444821 Non-glycosylated

200 **N**TV -1.0299987 Non-glycosylated

**225 NNT 0.88963983 Potential Glycosylated**

226 **N**TG -1.0135771 Non-glycosylated

274 **N**LF -1.3425718 Non-glycosylated

328 **N**RL -1.0222076 Non-glycosylated

337 **N**AE -1.1197133 Non-glycosylated

352 **N**DP -1.1451453 Non-glycosylated

**373 NGT 1.2707122 Potential Glycosylated**

384 **N**AL -0.93013976 Non-glycosylated

389 **N**PQ -0.85753812 Non-glycosylated

404 **N**AM -1.3410327 Non-glycosylated

***********************************

>Ss265 Length = 412

**Potential N-Linked Glycosylated Sites:**

MHYY**N**KLKFLALASVISATSAHPTSDHYYADAESEACL**N**GKAVYVTS**N**TEH**N**SVVAIPIDR**N**GSLLV**N**YATSTATGGRGG**N**GI**N**PRGMPAGPDALFGQGS

ITIAGDYLFAV**N**AGS**N**TVTMLAIDKHDPTKVTVVGEPAELPGEFPTTVGASDKF**N**LVCVGLTGAKAGVSCASYSWYGLGPFDELRPFDLHQTTPPHGPT

**N**TVSHVFFSGDQETVFTTVKGDPAV**NN**TGFLAAYPVEHIHSSCYATPSVSHKGVISSPEGTAVLFGSTPIPDTT**N**LFATDASFGAVILGIEDYEASTLY

KTVIPGQDATCWVAICPATHTAFVTDIRM**N**RLVEMSLA**N**AEIIGEPIDLTTFS**N**DPGLTEIRSGGSFVYALSPG**N**GTTEAWITVL**N**ALTKKPVQHALLT

PLGLDR**N**AMGMAILV

------------------------------------------------

Position Residue Score Prediction

------------------------------------------------

5 **N**KL -0.77832304 Non-glycosylated

39 **N**GK -1.130584 Non-glycosylated

48 **N**TE -1.2741531 Non-glycosylated

52 **N**SV -0.82172973 Non-glycosylated

**62 NGS 1.0956073 Potential Glycosylated**

68 **N**YA -0.96574406 Non-glycosylated

81 **N**GI -1.2423291 Non-glycosylated

84 **N**PR -1.8662045 Non-glycosylated

112 **N**AG -1.0674394 Non-glycosylated

116 **N**TV -0.66346953 Non-glycosylated

155 **N**LV -1.0444821 Non-glycosylated

200 **N**TV -1.0299987 Non-glycosylated

**225 NNT 0.88963983 Potential Glycosylated**

226 **N**TG -1.0135771 Non-glycosylated

274 **N**LF -1.3425718 Non-glycosylated

328 **N**RL -1.0222076 Non-glycosylated

337 **N**AE -1.1197133 Non-glycosylated

352 **N**DP -1.1451453 Non-glycosylated

**373 NGT 1.2707122 Potential Glycosylated**

384 **N**AL -1.0307742 Non-glycosylated

404 **N**AM -1.3410327 Non-glycosylated

***********************************

>Ss226 Length = 412

**Potential N-Linked Glycosylated Sites:**

MHYY**N**KLKFLALASVISATSAHPTSDHYYADAESEACL**N**GKAVYVTS**N**TEH**N**SVVAIPIAR**N**GSLLV**N**YATSTATGGRGG**N**GI**N**PRGMPAGPDALFGQGS

ITIAGDYLFAV**N**AGS**N**TVTMLAIDKHDPTKVTVVGEPAELPGEFPTTVGASDKF**N**LVCVGLTGAKAGVSCASYSWYGLGPFDELRPFDLHQTTPPHGPT

**N**TVSHVFFSGDQETVFTTVKGDPAV**NN**TGFLAAYPVEHIHSSCYATPSVSHKGVISSPEGTAVLFGSTPIPDTT**N**LFATDASFGAVILGIDDYEASTLY

KTVIPGQDATCWVAICPATHTAFVTDIRM**N**RLVEMSLVDAGIIGEPIDLTTFS**N**DPGLTEIRSGGSFVYALSPG**N**GTTEAWITVL**N**ALTKKPVQHALLT

PLGLDR**N**AMGMAILV

------------------------------------------------

Position Residue Score Prediction

------------------------------------------------

5 **N**KL -0.77832304 Non-glycosylated

39 **N**GK -1.130584 Non-glycosylated

48 **N**TE -1.2741531 Non-glycosylated

52 **N**SV -0.83972275 Non-glycosylated

**62 NGS 1.1524071 Potential Glycosylated**

68 **N**YA -0.9795019 Non-glycosylated

81 **N**GI -1.2423291 Non-glycosylated

84 **N**PR -1.8662045 Non-glycosylated

112 **N**AG -1.0674394 Non-glycosylated

116 **N**TV -0.66346953 Non-glycosylated

155 **N**LV -1.0444821 Non-glycosylated

200 **N**TV -1.0299987 Non-glycosylated

**225 NNT 0.88963983 Potential Glycosylated**

226 **N**TG -1.0135771 Non-glycosylated

274 **N**LF -1.3425718 Non-glycosylated

328 **N**RL -0.99138009 Non-glycosylated

352 **N**DP -1.1451453 Non-glycosylated

**373 NGT 1.2707122 Potential Glycosylated**

384 **N**AL -1.0307742 Non-glycosylated

404 **N**AM -1.3410327 Non-glycosylated

***********************************

>Ss172 Length = 412

**Potential N-Linked Glycosylated Sites:**

MHYY**N**KLKFLALASVISATSAHPTSDHYYADAESEACL**N**GKAVYVTS**N**TEH**N**SVVAIPIDR**N**GSLLV**N**HATSTATGGRGG**N**GI**N**PRGMPAGPDALFGQGS

ITIAGDYLFAV**N**AGS**N**TVTMLAIDKHDPTKVTVVGEPAELPGEFPTTVGASDKF**N**LVCVGLTGAKAGVSCASYSWYGLGPFDELRPFDLHQTTPPHGPT

**N**TVSHVFFSGDQETVFTTVKGDPAV**NN**TGFLAAYPVEHIHSSCYATPSVSHKGVISSPEGTAVLFGSTPIPDTT**N**LFATDASFGAVILGIDDYEASTLY

KTVIPGQDATCWVAICPATHTAFVTDIRM**N**RLVEMSLVDAGIIGEPIDLTTFS**N**DPGLTEIRSGGSFVYALSPG**N**GTTEAWITVL**N**ALTK**N**PVQHALLT

PLGLDR**N**AMGMAILV

------------------------------------------------

Position Residue Score Prediction

------------------------------------------------

5 **N**KL -0.77832304 Non-glycosylated

39 **N**GK -1.130584 Non-glycosylated

48 **N**TE -1.2741531 Non-glycosylated

52 **N**SV -0.82172973 Non-glycosylated

**62 NGS 1.168385 Potential Glycosylated**

68 **N**HA -0.90186243 Non-glycosylated

81 **N**GI -1.2423291 Non-glycosylated

84 **N**PR -1.8662045 Non-glycosylated

112 **N**AG -1.0674394 Non-glycosylated

116 **N**TV -0.66346953 Non-glycosylated

155 **N**LV -1.0444821 Non-glycosylated

200 **N**TV -1.0299987 Non-glycosylated

**225 NNT 0.88963983 Potential Glycosylated**

226 **N**TG -1.0135771 Non-glycosylated

274 **N**LF -1.3425718 Non-glycosylated

328 **N**RL -0.99138009 Non-glycosylated

352 **N**DP -1.1451453 Non-glycosylated

**373 NGT 1.2707122 Potential Glycosylated**

384 **N**AL -0.96353409 Non-glycosylated

389 **N**PV -1.0464722 Non-glycosylated

404 **N**AM -1.3410327 Non-glycosylated

***********************************

>Ss174 Length = 412

**Potential N-Linked Glycosylated Sites:**

MHYY**N**KLKFLALASVISATSAHPTSDHYYADAESEACL**N**GKAVYVTS**N**TEH**N**SVVAIPIDR**N**GSLLV**N**HATSTATGGRGG**N**GI**N**PRGMPAGPDALFGQGS

ITIAGDYLFAV**N**AGS**N**TVTMLAIDKHDPTKVTVVGEPAELPGEFPTTVGASDKF**N**LVCVGLTGAKAGVSCASYSWYGLGPFDELRPFDLHQTTPPHGPT

**N**TVSHVFFSGDQETVFTTVKGDPAV**NN**TGFLAAYPVEHIHSSCYATPSVSHKGVISSPEGTAVLFGSTPIPDTT**N**LFATDASFGAVILGIDDYEASTLY

KTVIPGQDATCWVAICPATHTAFVTDIRM**N**RLVEMSLVDAGIIGEPIDLTTFS**N**DPGLTEIRSGGSFVYALSPG**N**GTTEAWITVL**N**ALTKKPVQHALLT

PLGLDR**N**AMGMAILV

------------------------------------------------

Position Residue Score Prediction

------------------------------------------------

5 **N**KL -0.77832304 Non-glycosylated

39 **N**GK -1.130584 Non-glycosylated

48 **N**TE -1.2741531 Non-glycosylated

52 **N**SV -0.82172973 Non-glycosylated

**62 NGS 1.168385 Potential Glycosylated**

68 **N**HA -0.90186243 Non-glycosylated

81 **N**GI -1.2423291 Non-glycosylated

84 **N**PR -1.8662045 Non-glycosylated

112 **N**AG -1.0674394 Non-glycosylated

116 **N**TV -0.66346953 Non-glycosylated

155 **N**LV -1.0444821 Non-glycosylated

200 **N**TV -1.0299987 Non-glycosylated

**225 NNT 0.88963983 Potential Glycosylated**

226 **N**TG -1.0135771 Non-glycosylated

274 **N**LF -1.3425718 Non-glycosylated

328 **N**RL -0.99138009 Non-glycosylated

352 **N**DP -1.1451453 Non-glycosylated

**373 NGT 1.2707122 Potential Glycosylated**

384 **N**AL -1.0307742 Non-glycosylated

404 **N**AM -1.3410327 Non-glycosylated

***********************************

>Ss171 Length = 412

**Potential N-Linked Glycosylated Sites:**

MHYY**N**KLKFLALASVISATSAHPTSDHYYADAESEACL**N**GKAVYVTS**N**TEH**N**SVVAIPIDR**N**GSLLV**N**HATSTATGGRGG**N**GI**N**PRGMPAGPDALFGQGS

ITIAGDYLFAV**N**AGS**N**TVTMLAIDKHDPTKVTVVGEPAELPGEFPTTVGASDKF**N**LVCVGLTGAKAGVSCASYSWYGLGPFDELRPFDLHQTTPPHGPT

**N**TVSHVFFSGDQETVFTTVKGDPAV**NN**TGFLAAYPVEHIHSSCYATPSVSHKGVISSPEGTAVLFGSTPIPDTT**N**LFATDASFGAVILGIDDYEASTLY

KTVIPGQDATCWVAICPATHTAFVTDIRM**N**RLVEMSLVDAGIIGEPIDLTTFS**N**DPGLTEIRSGGSFVYALSPG**N**GTTEAWITVL**N**ALTKKPVQHALLT

PLGLDR**N**AMGMAILV

------------------------------------------------

Position Residue Score Prediction

------------------------------------------------

5 **N**KL -0.77832304 Non-glycosylated

39 **N**GK -1.130584 Non-glycosylated

48 **N**TE -1.2741531 Non-glycosylated

52 **N**SV -0.82172973 Non-glycosylated

**62 NGS 1.168385 Potential Glycosylated**

68 **N**HA -0.90186243 Non-glycosylated

81 **N**GI -1.2423291 Non-glycosylated

84 **N**PR -1.8662045 Non-glycosylated

112 **N**AG -1.0674394 Non-glycosylated

116 **N**TV -0.66346953 Non-glycosylated

155 **N**LV -1.0444821 Non-glycosylated

200 **N**TV -1.0299987 Non-glycosylated

**225 NNT 0.88963983 Potential Glycosylated**

226 **N**TG -1.0135771 Non-glycosylated

274 **N**LF -1.3425718 Non-glycosylated

328 **N**RL -0.99138009 Non-glycosylated

352 **N**DP -1.1451453 Non-glycosylated

**373 NGT 1.2707122 Potential Glycosylated**

384 **N**AL -1.0307742 Non-glycosylated

404 **N**AM -1.3410327 Non-glycosylated

***********************************

>Ss034 Length = 412

**Potential N-Linked Glycosylated Sites:**

MHYY**N**KLKFLALASVISATSAHPTSDHYYADAESEACL**N**GKAVYVTS**N**TEH**N**SVVAIPIAR**N**GSLLV**N**HATSTATGGRGG**N**GI**N**PRGMPAGPDALFGQGS

ITRAGDYLFTV**N**AGS**N**TVTMLAIDKHDPTKVTVVGEPAELPGEFPTTVGASDKF**N**LVCVGLTGAKAGVSCASYSWYGLGPFDELRPFDLHQTTPPHGPT

**N**TVSHVFFSGDQETVFTTVKGDPAV**NN**TGFLAAYPVEHIHSSCYATPSVSHKGVISSPDGTAVLFGSTPIPDTT**N**LFATDASFGAVILGIDDYEASTLY

KTVIPGQDATCWVAICPATHTAFVTDIRM**N**RLVEMSLVDAGIIGEPFDLTTFS**N**DPGLTEIRSGGSFVYALSPGYGTTEAWITVL**N**ALTKKPVQHALLT

PLGLDR**N**AMGMAILV

------------------------------------------------

Position Residue Score Prediction

------------------------------------------------

5 **N**KL -0.77832304 Non-glycosylated

39 **N**GK -1.130584 Non-glycosylated

48 **N**TE -1.2741531 Non-glycosylated

52 **N**SV -0.83972275 Non-glycosylated

**62 NGS 1.1964524 Potential Glycosylated**

68 **N**HA -0.91851837 Non-glycosylated

81 **N**GI -1.2423291 Non-glycosylated

84 **N**PR -1.8662045 Non-glycosylated

112 **N**AG -1.2004567 Non-glycosylated

116 **N**TV -0.71455394 Non-glycosylated

155 **N**LV -1.0444821 Non-glycosylated

200 **N**TV -1.0299987 Non-glycosylated

**225 NNT 0.88963983 Potential Glycosylated**

226 **N**TG -1.0135771 Non-glycosylated

274 **N**LF -1.3425718 Non-glycosylated

328 **N**RL -0.99138009 Non-glycosylated

352 **N**DP -1.152516 Non-glycosylated

384 **N**AL -1.0307742 Non-glycosylated

404 **N**AM -1.3410327 Non-glycosylated

***********************************

>Ss185 Length = 413

**Potential N-Linked Glycosylated Sites:**

MHYY**N**KLKFLALASVFSVTSAHPTSGHYYDDAESEACL**N**GKAVYVTS**N**TEH**N**SVVAIPIAR**N**GSLLL**N**HATSTATGGRGG**N**GI**N**PRGMPAGPDALFSQGS

ITSAG**N**YLFAV**N**AGS**N**TVTMLAIDEHDPTKVTVVGEPAELPGEFPTTVGASDKF**N**LVCVGLTGAKAGVSCASYSWYGLGPFDELRTFDLHQTTPPHGPT

**N**TVSHVFFSGDQETVFTTVKGDPAV**NN**TGFLAAYPVEHIHSSCYAIPSVSHKGVISSPDGTAVLFGSTPIPDTT**N**LFVTDASFGAAILGIDDYEEASTL

YKTVIPGQDATCWVAICPATHTAFVTDIRM**N**RLVEMSLV**N**AEIIGEPIDLTTF**N**TDPGLTEIRSGGSFVYALSPG**N**GTTEAWITVL**N**ALTKKPVQHALL

TPLGLDR**N**AMGMAILV

------------------------------------------------

Position Residue Score Prediction

------------------------------------------------

5 **N**KL -0.77832304 Non-glycosylated

39 **N**GK -1.1287597 Non-glycosylated

48 **N**TE -1.2741531 Non-glycosylated

52 **N**SV -0.83972275 Non-glycosylated

**62 NGS 1.1882916 Potential Glycosylated**

68 **N**HA -0.89887659 Non-glycosylated

81 **N**GI -1.2423291 Non-glycosylated

84 **N**PR -1.8662045 Non-glycosylated

106 **N**YL -0.7359858 Non-glycosylated

112 **N**AG -0.84383581 Non-glycosylated

116 **N**TV -0.70783397 Non-glycosylated

155 **N**LV -1.0444821 Non-glycosylated

200 **N**TV -1.0299987 Non-glycosylated

**225 NNT 0.88963983 Potential Glycosylated**

226 **N**TG -1.0135771 Non-glycosylated

274 **N**LF -1.2838351 Non-glycosylated

329 **N**RL -1.0665658 Non-glycosylated

338 **N**AE -1.1315083 Non-glycosylated

352 **N**TD -1.3580699 Non-glycosylated

**374 NGT 1.2707122 Potential Glycosylated**

385 **N**AL -1.0307742 Non-glycosylated

405 **N**AM -1.3410327 Non-glycosylated

***********************************

>S7 Length = 413

**Potential N-Linked Glycosylated Sites:**

MHYY**N**KLKFLALASVFSVTSAHPTSGHYYDDAESEACL**N**GKAVYVTS**N**TEH**N**SVVAIPIAR**N**GSLLL**N**HATSTATGGRGG**N**GI**N**PRGMPAGPDALFSQGS

ITSAG**N**YLFAV**N**AGS**N**TVTMLAIDEHDPTKVTVVGEPAELPGEFPTTVGASDKF**N**LVCVGLTGAKAGVSCASYSWYGLGPFDELRTFDLHQTTPPHGPT

**N**TVSHVFFSGDQETVFTTVKGDPAV**NN**TGFLAAYPVEHIHSSCYAIPSVSHKGVISSPDGTAVLFGSTPIPDTT**N**LFVTDASFGAAILGIDDYEEASTL

YKTVIPGQDATCWVAICPATHTAFVTDIRM**N**RLVEMSLV**N**AEIIGEPIDLTTF**N**TDPGLTEIRSGGSFVYALSPG**N**GTTEAWITVL**N**ALTKKPVQHALL

TPLGLDR**N**AMGMAILV

------------------------------------------------

Position Residue Score Prediction

------------------------------------------------

5 **N**KL -0.77832304 Non-glycosylated

39 **N**GK -1.1287597 Non-glycosylated

48 **N**TE -1.2741531 Non-glycosylated

52 **N**SV -0.83972275 Non-glycosylated

**62 NGS 1.1882916 Potential Glycosylated**

68 **N**HA -0.89887659 Non-glycosylated

81 **N**GI -1.2423291 Non-glycosylated

84 **N**PR -1.8662045 Non-glycosylated

106 **N**YL -0.7359858 Non-glycosylated

112 **N**AG -0.84383581 Non-glycosylated

116 **N**TV -0.70783397 Non-glycosylated

155 **N**LV -1.0444821 Non-glycosylated

200 **N**TV -1.0299987 Non-glycosylated

**225 NNT 0.88963983 Potential Glycosylated**

226 **N**TG -1.0135771 Non-glycosylated

274 **N**LF -1.2838351 Non-glycosylated

329 **N**RL -1.0665658 Non-glycosylated

338 **N**AE -1.1315083 Non-glycosylated

352 **N**TD -1.3580699 Non-glycosylated

**374 NGT 1.2707122 Potential Glycosylated**

385 **N**AL -1.0307742 Non-glycosylated

405 **N**AM -1.3410327 Non-glycosylated

***********************************

>ATCC58251 Length = 413

**Potential N-Linked Glycosylated Sites:**

MHYY**N**KLKFLALASVFSVTSAHPTSGHYYDDAESEACL**N**GKAVYVTS**N**TEH**N**SVVAIPIAR**N**GSLLL**N**HATSTATGGRGG**N**GI**N**PRGMPAGPDALFSQGS

ITSAG**N**YLFAV**N**AGS**N**TVTMLAIDEHDPTKVTVVGEPAELPGEFPTTVGASDKF**N**LVCVGLTGAKAGVSCASYSWYGLGPFDELRTFDLHQTTPPHGPT

**N**TVSHVFFSGDQETVFTTVKGDPAV**NN**TGFLAAYPVEHIHSSCYAIPSVSHKGVISSPDGTAVLFGSTPIPDTT**N**LFVTDASFGAAILGIDDYEEASTL

YKTVIPGQDATCWVAICPATHTAFVTDIRM**N**RLVEMSLV**N**AEIIGEPIDLTTF**N**TDPGLTEIRSGGSFVYALSPG**N**GTTEAWITVL**N**ALTKKPVQHALL

TPLGLDR**N**AMGMAILV

------------------------------------------------

Position Residue Score Prediction

------------------------------------------------

5 **N**KL -0.77832304 Non-glycosylated

39 **N**GK -1.1287597 Non-glycosylated

48 **N**TE -1.2741531 Non-glycosylated

52 **N**SV -0.83972275 Non-glycosylated

**62 NGS 1.1882916 Potential Glycosylated**

68 **N**HA -0.89887659 Non-glycosylated

81 **N**GI -1.2423291 Non-glycosylated

84 **N**PR -1.8662045 Non-glycosylated

106 **N**YL -0.7359858 Non-glycosylated

112 **N**AG -0.84383581 Non-glycosylated

116 **N**TV -0.70783397 Non-glycosylated

155 **N**LV -1.0444821 Non-glycosylated

200 **N**TV -1.0299987 Non-glycosylated

**225 NNT 0.88963983 Potential Glycosylated**

226 **N**TG -1.0135771 Non-glycosylated

274 **N**LF -1.2838351 Non-glycosylated

329 **N**RL -1.0665658 Non-glycosylated

338 **N**AE -1.1315083 Non-glycosylated

352 **N**TD -1.3580699 Non-glycosylated

**374 NGT 1.2707122 Potential Glycosylated**

385 **N**AL -1.0307742 Non-glycosylated

405 **N**AM -1.3410327 Non-glycosylated

***********************************

>Ss143 Length = 413

**Potential N-Linked Glycosylated Sites:**

MHYY**N**KLKFLALASVFSITSAHPTSGHYYDDAESEACL**N**GKAVYVTS**N**TEH**N**SVVAIPIAR**N**GSLLL**N**HATSTATGGRGG**N**GI**N**PRGMPAGPDALFSQGS

ITSAG**N**YLFAV**N**AGS**N**TVTMLAIDEHDPTKVTVVGEPAELPGEFPTTVGASDKF**N**LVCVGLTGAKAGVSCASYSWYGLGPFDELRTFDLHQTTPPHGPT

**N**TVSHVFFSGDQETVFTTVKGDPAV**NN**TGFLAAYPVEHIHSSCYAIPSVSHKGVISSPDGTAVLFGSTPIPDTT**N**LFVTDASFGAAILGIDDYEEASTL

YKTVIPGQDATCWVAICPATHTAFVTDIRM**N**RLVEMSLV**N**AEIIGEPIDLTTF**N**TDPGLTEIRSGGSFVYALSPG**N**GTTEAWITVL**N**ALTKKPVQHALL

TPLGLDR**N**AMGMAILV

------------------------------------------------

Position Residue Score Prediction

------------------------------------------------

5 **N**KL -0.77832304 Non-glycosylated

39 **N**GK -1.1287597 Non-glycosylated

48 **N**TE -1.2741531 Non-glycosylated

52 **N**SV -0.83972275 Non-glycosylated

**62 NGS 1.1882916 Potential Glycosylated**

68 **N**HA -0.89887659 Non-glycosylated

81 **N**GI -1.2423291 Non-glycosylated

84 **N**PR -1.8662045 Non-glycosylated

106 **N**YL -0.7359858 Non-glycosylated

112 **N**AG -0.84383581 Non-glycosylated

116 **N**TV -0.70783397 Non-glycosylated

155 **N**LV -1.0444821 Non-glycosylated

200 **N**TV -1.0299987 Non-glycosylated

**225 NNT 0.88963983 Potential Glycosylated**

226 **N**TG -1.0135771 Non-glycosylated

274 **N**LF -1.2838351 Non-glycosylated

329 **N**RL -1.0665658 Non-glycosylated

338 **N**AE -1.1315083 Non-glycosylated

352 **N**TD -1.3580699 Non-glycosylated

**374 NGT 1.2707122 Potential Glycosylated**

385 **N**AL -1.0307742 Non-glycosylated

405 **N**AM -1.3410327 Non-glycosylated

***********************************

>109918 Length = 413

**Potential N-Linked Glycosylated Sites:**

MHYY**N**KLKFLALASVFSVTSAHPTSGHYYDDAESEACL**N**GKAVYVTS**N**TEH**N**SVVAIPIAR**N**GSLLL**N**HATSTATGGRGG**N**GI**N**PRGMPAGPDALFSQGS

ITSAG**N**YLFAV**N**AGS**N**TVTMLAIDEHDPTKVTVVGEPAELPGEFPTTVGASDKF**N**LVCVGLTGAKAGVSCASYSWYGLGPFDELRTFDLHQTTPPHGPT

**N**TVSHVFFSGDQETVFTTVKGDPAV**NN**TGFLAAYPVEHIHSSCYAIPSVSHKGVISSPDGTAVLFGSTPIPDTT**N**LFVTDASFGAAILGIDDYEEASTL

YKTVIPGQDATCWVAICPATHTAFVTDIRM**N**RLVEMSLV**N**AEIIGEPIDLTTF**N**TDPGLTEIRSGGSFVYALSPG**N**GTTEAWITVL**N**ALTKKPVQHALL

TPLGLDR**N**AMGMAILV

------------------------------------------------

Position Residue Score Prediction

------------------------------------------------

5 **N**KL -0.77832304 Non-glycosylated

39 **N**GK -1.1287597 Non-glycosylated

48 **N**TE -1.2741531 Non-glycosylated

52 **N**SV -0.83972275 Non-glycosylated

**62 NGS 1.1882916 Potential Glycosylated**

68 **N**HA -0.89887659 Non-glycosylated

81 **N**GI -1.2423291 Non-glycosylated

84 **N**PR -1.8662045 Non-glycosylated

106 **N**YL -0.7359858 Non-glycosylated

112 **N**AG -0.84383581 Non-glycosylated

116 **N**TV -0.70783397 Non-glycosylated

155 **N**LV -1.0444821 Non-glycosylated

200 **N**TV -1.0299987 Non-glycosylated

**225 NNT 0.88963983 Potential Glycosylated**

226 **N**TG -1.0135771 Non-glycosylated

274 **N**LF -1.2838351 Non-glycosylated

329 **N**RL -1.0665658 Non-glycosylated

338 **N**AE -1.1315083 Non-glycosylated

352 **N**TD -1.3580699 Non-glycosylated

**374 NGT 1.2707122 Potential Glycosylated**

385 **N**AL -1.0307742 Non-glycosylated

405 **N**AM -1.3410327 Non-glycosylated

***********************************

>Ss476 Length = 413

**Potential N-Linked Glycosylated Sites:**

MHYY**N**KLKFLALASVFSATSAHPTSGHYYDDAESEACL**N**GKAVYVTS**N**TEH**N**SVVAIPIAR**N**GSLLL**N**HATSTATGGRGG**N**GI**N**PRGMPAGPDALFSQGS

ITSAG**N**YLFAV**N**AGS**N**TVTMLAIDEHDPTKVTVVGEPAELPGEFPTTVGASDKF**N**LVCVGLTGAKAGVSCASYSWYGLGPFDELRTFDLHQTTPPHGPT

**N**TVSHVFFSGDQETVFTTVKGDPAV**NN**TGFLAAYPVEHIHSSCYAIPSVSHKGVISSPDGTAVLFGSTPIPDTT**N**LFVTDASFGAAILGIDDYEEASTL

YKTVIPGQDATCWVAICPATHTAFVTDIRM**N**RLVEMSLV**N**AEIIGEPIDLTTF**N**TDPGLTEIRSGGSFVYALSPG**N**GTTEAWITVL**N**ALTKKPVQHALL

TPLGLDR**N**AMGMAILV

------------------------------------------------

Position Residue Score Prediction

------------------------------------------------

5 **N**KL -0.77832304 Non-glycosylated

39 **N**GK -1.1287597 Non-glycosylated

48 **N**TE -1.2741531 Non-glycosylated

52 **N**SV -0.83972275 Non-glycosylated

**62 NGS 1.1882916 Potential Glycosylated**

68 **N**HA -0.89887659 Non-glycosylated

81 **N**GI -1.2423291 Non-glycosylated

84 **N**PR -1.8662045 Non-glycosylated

106 **N**YL -0.7359858 Non-glycosylated

112 **N**AG -0.84383581 Non-glycosylated

116 **N**TV -0.70783397 Non-glycosylated

155 **N**LV -1.0444821 Non-glycosylated

200 **N**TV -1.0299987 Non-glycosylated

**225 NNT 0.88963983 Potential Glycosylated**

226 **N**TG -1.0135771 Non-glycosylated

274 **N**LF -1.2838351 Non-glycosylated

329 **N**RL -1.0665658 Non-glycosylated

338 **N**AE -1.1315083 Non-glycosylated

352 **N**TD -1.3580699 Non-glycosylated

**374 NGT 1.2707122 Potential Glycosylated**

385 **N**AL -1.0307742 Non-glycosylated

405 **N**AM -1.3410327 Non-glycosylated

***********************************

>Ss478 Length = 413

**Potential N-Linked Glycosylated Sites:**

MHYY**N**KLKFLALASVFSATSAHPTSGHYYDDAESEACL**N**GKAVYVTS**N**TEH**N**SVVAIPIAR**N**GSLLL**N**HATSTATGGRGG**N**GI**N**PRGMPAGPDALFSQGS

ITSAG**N**YLFAV**N**AGS**N**TVTMLAIDEHDPTKVTVVGEPAELPGEFPTTVGASDKF**N**LVCVGLTGAKAGVSCASYSWYGLGPFDELRTFDLHQTTPPHGPT

**N**TVSHVFFSGDQETVFTTVKGDPAV**NN**TGFLAAYPVEHIHSSCYAIPSVSHKGVISSPDGTAVLFGSTPIPDTT**N**LFVTDASFGAAILGIDDYGEASTL

YKTVIPGQDATCWVAICPATHTAFVTDIRM**N**RLVEMSLV**N**AEIIGEPIDLTTF**N**TDPGLTEIRSGGSFVYALSPG**N**GTTEAWITVL**N**ALTKKPVQHALL

TPLGLDR**N**AMGMAILV

------------------------------------------------

Position Residue Score Prediction

------------------------------------------------

5 **N**KL -0.77832304 Non-glycosylated

39 **N**GK -1.1287597 Non-glycosylated

48 **N**TE -1.2741531 Non-glycosylated

52 **N**SV -0.83972275 Non-glycosylated

**62 NGS 1.1882916 Potential Glycosylated**

68 **N**HA -0.89887659 Non-glycosylated

81 **N**GI -1.2423291 Non-glycosylated

84 **N**PR -1.8662045 Non-glycosylated

106 **N**YL -0.7359858 Non-glycosylated

112 **N**AG -0.84383581 Non-glycosylated

116 **N**TV -0.70783397 Non-glycosylated

155 **N**LV -1.0444821 Non-glycosylated

200 **N**TV -1.0299987 Non-glycosylated

**225 NNT 0.88963983 Potential Glycosylated**

226 **N**TG -1.0135771 Non-glycosylated

274 **N**LF -1.2838351 Non-glycosylated

329 **N**RL -1.0665658 Non-glycosylated

338 **N**AE -1.1315083 Non-glycosylated

352 **N**TD -1.3580699 Non-glycosylated

**374 NGT 1.2707122 Potential Glycosylated**

385 **N**AL -1.0307742 Non-glycosylated

405 **N**AM -1.3410327 Non-glycosylated

***********************************

>Ss477 Length = 413

**Potential N-Linked Glycosylated Sites:**

MHYY**N**KLKFLALASVFSATSAHPTSGHYYDDAESEACL**N**GKAVYVTS**N**TEH**N**SVVAIPIAR**N**GSLLL**N**HATSTATGGRGG**N**GI**N**PRGMPAGPDALFSQGS

ITSAG**N**YLFAV**N**AGS**N**TVTMLAIDEHDPTKVTVVGEPAELPGEFPTTVGASDKF**N**LVCVGLTGAKAGVSCASYSWYGLGPFDELRTFDLHQTTPPHGPT

**N**TVSHVFFSGDQETVFTTVKGDPAV**NN**TGFLAAYPVEHIHSSCYAIPSVSHKGVISSPDGTAVLFGSTPIPDTT**N**LFVTDASFGAAILGIDDYGEASTL

YKTVIPGQDATCWVAICPATHTAFVTDIRM**N**RLVEMSLV**N**AEIIGEPIDLTTF**N**TDPGLTEIRSGGSFVYALSPG**N**GTTEAWITVL**N**ALTKKPVQHALL

TPLGLDR**N**AMGMAILV

------------------------------------------------

Position Residue Score Prediction

------------------------------------------------

5 **N**KL -0.77832304 Non-glycosylated

39 **N**GK -1.1287597 Non-glycosylated

48 **N**TE -1.2741531 Non-glycosylated

52 **N**SV -0.83972275 Non-glycosylated

**62 NGS 1.1882916 Potential Glycosylated**

68 **N**HA -0.89887659 Non-glycosylated

81 **N**GI -1.2423291 Non-glycosylated

84 **N**PR -1.8662045 Non-glycosylated

106 **N**YL -0.7359858 Non-glycosylated

112 **N**AG -0.84383581 Non-glycosylated

116 **N**TV -0.70783397 Non-glycosylated

155 **N**LV -1.0444821 Non-glycosylated

200 **N**TV -1.0299987 Non-glycosylated

**225 NNT 0.88963983 Potential Glycosylated**

226 **N**TG -1.0135771 Non-glycosylated

274 **N**LF -1.2838351 Non-glycosylated

329 **N**RL -1.0665658 Non-glycosylated

338 **N**AE -1.1315083 Non-glycosylated

352 **N**TD -1.3580699 Non-glycosylated

**374 NGT 1.2707122 Potential Glycosylated**

385 **N**AL -1.0307742 Non-glycosylated

405 **N**AM -1.3410327 Non-glycosylated

***********************************

>Ss176 Length = 413

**Potential N-Linked Glycosylated Sites:**

MHYY**N**KLKFLALASVFSATSAHPTSGHYYDDAESEACL**N**GKAVYVTS**N**TEH**N**SVVAIPIAR**N**GSLLL**N**HATSTATGGRGG**N**GI**N**PRGMPAGPDALFSQGS

ITSAG**N**YLFAV**N**AGS**N**TVTMLAIDEHDPTKVTVVGEPAELPGEFPTTVGASDKF**N**LVCVGLTGAKAGVSCASYSWYGLGPFDELRTFDLHQTTPPHGPT

**N**TVSHVFFSGDQETVFTTVKGDPAV**NN**TGFLAAYPVEHIHSSCYAIPSVSHKGVISSPDGTAVLFGSTPIPDTT**N**LFVTDASFGAAILGIDDYEEASTL

YKTVIPGQDATCWVAICPATHTAFVTDIRM**N**RLVEMSLV**N**AEIIGEPIDLTTF**N**TDPGLTEIRSGGSFVYALSPG**N**GTTEAWITVL**N**ALTKKPVQHALL

TPLGLDR**N**AMGMAILV

------------------------------------------------

Position Residue Score Prediction

------------------------------------------------

5 **N**KL -0.77832304 Non-glycosylated

39 **N**GK -1.1287597 Non-glycosylated

48 **N**TE -1.2741531 Non-glycosylated

52 **N**SV -0.83972275 Non-glycosylated

**62 NGS 1.1882916 Potential Glycosylated**

68 **N**HA -0.89887659 Non-glycosylated

81 **N**GI -1.2423291 Non-glycosylated

84 **N**PR -1.8662045 Non-glycosylated

106 **N**YL -0.7359858 Non-glycosylated

112 **N**AG -0.84383581 Non-glycosylated

116 **N**TV -0.70783397 Non-glycosylated

155 **N**LV -1.0444821 Non-glycosylated

200 **N**TV -1.0299987 Non-glycosylated

**225 NNT 0.88963983 Potential Glycosylated**

226 **N**TG -1.0135771 Non-glycosylated

274 **N**LF -1.2838351 Non-glycosylated

329 **N**RL -1.0665658 Non-glycosylated

338 **N**AE -1.1315083 Non-glycosylated

352 **N**TD -1.3580699 Non-glycosylated

**374 NGT 1.2707122 Potential Glycosylated**

385 **N**AL -1.0307742 Non-glycosylated

405 **N**AM -1.3410327 Non-glycosylated

***********************************

>Ss529 Length = 413

**Potential N-Linked Glycosylated Sites:**

MHYY**N**KLKFLALASVFSATSAHPTSGHYYDDAESEACL**N**GKAVYVTS**N**TEH**N**SVVAIPIAR**N**GSLLL**N**HATSTATGGRGG**N**GI**N**PRGMPAGPDALFSQGS

ITSAG**N**YLFAV**N**AGS**N**TVTMLAIDEHDPTKVTVVGEPAELPGEFPTTVGASDKF**N**LVCVGLTGAKAGVSCASYSWYGLGPFDELRTFDLHQTTPPHGPT

**N**TVSHVFFSGDQETVFTTVKGDPAV**NN**TGFLAAYPVEHIHSSCYAIPSVSHKGVISSPDGTAVLFGSTPIPDTT**N**LFVTDASFGAAILGIDDYEEAFTL

YKTVIPGQDATCWVAICPATHTAFVTDIRM**N**RLVEMSLV**N**AEIIGEPIDLTTF**N**TDPGLTEIRSGGSFVYALSPG**N**GTTEAWITVL**N**ALTKKPVQHALL

TPLGLDR**N**AMGMAILV

------------------------------------------------

Position Residue Score Prediction

------------------------------------------------

5 **N**KL -0.77832304 Non-glycosylated

39 **N**GK -1.1287597 Non-glycosylated

48 **N**TE -1.2741531 Non-glycosylated

52 **N**SV -0.83972275 Non-glycosylated

**62 NGS 1.1882916 Potential Glycosylated**

68 **N**HA -0.89887659 Non-glycosylated

81 **N**GI -1.2423291 Non-glycosylated

84 **N**PR -1.8662045 Non-glycosylated

106 **N**YL -0.7359858 Non-glycosylated

112 **N**AG -0.84383581 Non-glycosylated

116 **N**TV -0.70783397 Non-glycosylated

155 **N**LV -1.0444821 Non-glycosylated

200 **N**TV -1.0299987 Non-glycosylated

**225 NNT 0.88963983 Potential Glycosylated**

226 **N**TG -1.0135771 Non-glycosylated

274 **N**LF -1.2838351 Non-glycosylated

329 **N**RL -1.0665658 Non-glycosylated

338 **N**AE -1.1315083 Non-glycosylated

352 **N**TD -1.3580699 Non-glycosylated

**374 NGT 1.2707122 Potential Glycosylated**

385 **N**AL -1.0307742 Non-glycosylated

405 **N**AM -1.3410327 Non-glycosylated

***********************************

>Ss530 Length = 413

**Potential N-Linked Glycosylated Sites:**

MHYY**N**KLKFLALTSVISVTSAHPTSGHYYDDAESEACL**N**GKAVYVTS**N**TEH**N**SVVAIPIAR**N**GSLLL**N**HATSTATGGRGG**N**GI**N**PRGMPAGPDALFSQGS

ITSAG**N**YLFAV**N**AGS**N**TVTMLAIDEHDPTKVTVVGEPAELPGEFPTTVGASDKF**N**LVCVGLTGAKAGVSCASYSWYGLGPFDELRTFDLHQTTPPHGPT

**N**TVSHVFFSGDQETVFTTVKGDPAV**NN**TGFLAAYPVEHIHSSCYAIPSVSHKGVISSPDGTAVLFGSTPIPDTT**N**LFVTDASFGAAILGIDDYEEASTL

YKTVIPGQDATCWVAICPATHTAFVTDIRM**N**RLVEMSLV**N**AEIIGEPIDLTTF**N**TDPGLTEIRSGGSFVYALSPG**N**GTTEAWITVL**N**ALTKKPVQYALL

TPLGLDR**N**AMGMAILV

------------------------------------------------

Position Residue Score Prediction

------------------------------------------------

5 **N**KL -0.7253544 Non-glycosylated

39 **N**GK -1.1287597 Non-glycosylated

48 **N**TE -1.2741531 Non-glycosylated

52 **N**SV -0.83972275 Non-glycosylated

**62 NGS 1.1882916 Potential Glycosylated**

68 **N**HA -0.89887659 Non-glycosylated

81 **N**GI -1.2423291 Non-glycosylated

84 **N**PR -1.8662045 Non-glycosylated

106 **N**YL -0.7359858 Non-glycosylated

112 **N**AG -0.84383581 Non-glycosylated

116 **N**TV -0.70783397 Non-glycosylated

155 **N**LV -1.0444821 Non-glycosylated

200 **N**TV -1.0299987 Non-glycosylated

**225 NNT 0.88963983 Potential Glycosylated**

226 **N**TG -1.0135771 Non-glycosylated

274 **N**LF -1.2838351 Non-glycosylated

329 **N**RL -1.0665658 Non-glycosylated

338 **N**AE -1.1315083 Non-glycosylated

352 **N**TD -1.3580699 Non-glycosylated

**374 NGT 1.2707122 Potential Glycosylated**

385 **N**AL -1.1293307 Non-glycosylated

405 **N**AM -1.3410327 Non-glycosylated

***********************************

>Ss39 Length = 413

**Potential N-Linked Glycosylated Sites:**

MHYY**N**KLKFLALASVFSLTSAHPTSGHYYDDAESEACL**N**GKAVYVTS**N**TEH**N**SVVAIPIAR**N**GSLLL**N**HATSTATGGRGG**N**GI**N**PRGMPAGPDALFSQGS

ITSAG**N**YLFAV**N**AGS**N**TVTMLAIDEHDPTKVTVVGEPAELPGEFPTTVGASDKF**N**LVCVGLTGAKAGVSCASYSWYGLGPFDELRTFDLHQTTPPHGPT

**N**TVSHVFFSGDQETVFTTVKGDPAV**NN**TGFLAAYPVEHIHSSCYAIPSVSHKGVISSPDGTAVLFGSTPIPDTT**N**LFVTDASFGAAILGIDDYEEASTL

YKTVIPGQDATCWVAICPATHTAFVTDIRM**N**RLVEMSLV**N**AEIIGEPIDLTTF**N**TDPGLTEIRSGGSFVYALSPG**N**GTTEAWITVL**N**ALTKKPVQHALL

TPLGLDR**N**AMGMAILV

------------------------------------------------

Position Residue Score Prediction

------------------------------------------------

5 **N**KL -0.77832304 Non-glycosylated

39 **N**GK -1.1287597 Non-glycosylated

48 **N**TE -1.2741531 Non-glycosylated

52 **N**SV -0.83972275 Non-glycosylated

**62 NGS 1.1882916 Potential Glycosylated**

68 **N**HA -0.89887659 Non-glycosylated

81 **N**GI -1.2423291 Non-glycosylated

84 **N**PR -1.8662045 Non-glycosylated

106 **N**YL -0.7359858 Non-glycosylated

112 **N**AG -0.84383581 Non-glycosylated

116 **N**TV -0.70783397 Non-glycosylated

155 **N**LV -1.0444821 Non-glycosylated

200 **N**TV -1.0299987 Non-glycosylated

**225 NNT 0.88963983 Potential Glycosylated**

226 **N**TG -1.0135771 Non-glycosylated

274 **N**LF -1.2838351 Non-glycosylated

329 **N**RL -1.0665658 Non-glycosylated

338 **N**AE -1.1315083 Non-glycosylated

352 **N**TD -1.3580699 Non-glycosylated

**374 NGT 1.2707122 Potential Glycosylated**

385 **N**AL -1.0307742 Non-glycosylated

405 **N**AM -1.3410327 Non-glycosylated

***********************************

>Ss64 Length = 413

**Potential N-Linked Glycosylated Sites:**

MHYY**N**KLKFLALASVFSVTSAHPTSGHYYDDAESEACL**N**GKAVYVTS**N**TEH**N**SVVAIPIAR**N**GSLLL**N**HSTSTATGGRGG**N**GI**N**PRGMPAGPDALFSQGS

ITSAG**N**YLFAV**N**AGS**N**TVTMLAIDEHDPTKVTVVGEPAELPGEFPTTVGASDKF**N**LVCVGLTGAKAGVSCASYSWYGLGPFDELRTFDLHQTTPPHGPT

**N**TVSHVFFSGDQETVFTTVKGDPAV**NN**TGFLAAYPVEHIHSSCYAIPSVSHKGVISSPDGTAVLFGSTPIPDTT**N**LFVTDASFGAAILGIDDYEEASTL

YKTVIPGQDATCWVAICPATHTAFVTDIRM**N**RLVEMSLV**N**AEIIGEPIDLTTF**N**TDPGLTEIRSGGSFVYALSPG**N**GTTEAWITVL**N**ALTKKPVQHALL

TPLGLDR**N**AMGMAILV

------------------------------------------------

Position Residue Score Prediction

------------------------------------------------

5 **N**KL -0.77832304 Non-glycosylated

39 **N**GK -1.1287597 Non-glycosylated

48 **N**TE -1.2741531 Non-glycosylated

52 **N**SV -0.83972275 Non-glycosylated

**62 NGS 1.1900245 Potential Glycosylated**

**68 NHS 1.0941601 Potential Glycosylated**

81 **N**GI -1.2423291 Non-glycosylated

84 **N**PR -1.8662045 Non-glycosylated

106 **N**YL -0.7359858 Non-glycosylated

112 **N**AG -0.84383581 Non-glycosylated

116 **N**TV -0.70783397 Non-glycosylated

155 **N**LV -1.0444821 Non-glycosylated

200 **N**TV -1.0299987 Non-glycosylated

**225 NNT 0.88963983 Potential Glycosylated**

226 **N**TG -1.0135771 Non-glycosylated

274 **N**LF -1.2838351 Non-glycosylated

329 **N**RL -1.0665658 Non-glycosylated

338 **N**AE -1.1315083 Non-glycosylated

352 **N**TD -1.3580699 Non-glycosylated

**374 NGT 1.2707122 Potential Glycosylated**

385 **N**AL -1.0307742 Non-glycosylated

405 **N**AM -1.3410327 Non-glycosylated

***********************************

>S6 Length = 413

**Potential N-Linked Glycosylated Sites:**

MHYY**N**KLKFLALASVFSVTSAHPTSGHYYDDAESEACL**N**GKAVYVTS**N**TEH**N**SVVAIPIAR**N**GSLLL**N**HATSTATGGRGG**N**GI**N**PRGMPAGPDALFSQGS

ITSAG**N**YLFAV**N**AGS**N**TVTMLAIDEHDPTKVTVVGEPAELPGEFPTTVGASDKF**N**LVCVGLTGAKAGVSCASYSWYGLGPFDELRTFDLHQTTPPHGPT

**N**TVSHVFFSGDQETVFTTVKGDPAV**NN**TGFLAAYPVEHIHSSCYAIPSVSHKGVISSPDGTAVLFGSTPIPDTT**N**LFVTDASFGAAILGI**N**DYEEASTL

YKTVIPGQDATCWVAICPATHTAFVTDIRM**N**RLVEMSLV**N**AEIIGEPIDLTTF**N**TDPGLTEIRSGGSFVYALSPG**N**GTTEAWITVL**N**ALTKKPVQHALL

TPLGLDR**N**AMGMAILV

------------------------------------------------

Position Residue Score Prediction

------------------------------------------------

5 **N**KL -0.77832304 Non-glycosylated

39 **N**GK -1.1287597 Non-glycosylated

48 **N**TE -1.2741531 Non-glycosylated

52 **N**SV -0.83972275 Non-glycosylated

**62 NGS 1.1882916 Potential Glycosylated**

68 **N**HA -0.89887659 Non-glycosylated

81 **N**GI -1.2423291 Non-glycosylated

84 **N**PR -1.8662045 Non-glycosylated

106 **N**YL -0.7359858 Non-glycosylated

112 **N**AG -0.84383581 Non-glycosylated

116 **N**TV -0.70783397 Non-glycosylated

155 **N**LV -1.0444821 Non-glycosylated

200 **N**TV -1.0299987 Non-glycosylated

**225 NNT 0.88963983 Potential Glycosylated**

226 **N**TG -1.0135771 Non-glycosylated

274 **N**LF -1.2838351 Non-glycosylated

290 **N**DY -0.90617534 Non-glycosylated

329 **N**RL -1.0665658 Non-glycosylated

338 **N**AE -1.1315083 Non-glycosylated

352 **N**TD -1.3580699 Non-glycosylated

**374 NGT 1.2707122 Potential Glycosylated**

385 **N**AL -1.0307742 Non-glycosylated

405 **N**AM -1.3410327 Non-glycosylated

***********************************

>Ss118 Length = 413

**Potential N-Linked Glycosylated Sites:**

MHYY**N**KLKFLALASVISVTSAHPTSGHYYDDAESEACL**N**GKAVYVTS**N**TEH**N**SVVAIPIAR**N**GSLLL**N**HATSTATGGRGG**N**GI**N**PRGMPAGPDALFSQGS

ITSAG**N**YLFAV**N**AGS**N**TVTMLAIDEHDPTKVTVVGEPAELPGEFPTTVGASDKF**N**LVCVGLTGAKAGVSCASYSWYGLGPFDELRTFDLHQTTPPHGPT

**N**TVSHVFFSGDQETVFTTVKGDPAV**NN**TGFLAAYPVEHIHSSCYAIPSVSHKGVISSPDGTAVLFGSTPIPDTT**N**LFVTDASFGAAILGIDDYEEASTL

YKTVIPGQDATCWVAICPATHTAFVTDIRM**N**RLVEMSLV**N**AEIIGEPIDLTTF**N**TDPGLTEIRSGGSFVYALSPG**N**GTTEAWITVL**N**ALTKKPVQHALL

TPLGLDR**N**AMGMAILV

------------------------------------------------

Position Residue Score Prediction

------------------------------------------------

5 **N**KL -0.77832304 Non-glycosylated

39 **N**GK -1.1287597 Non-glycosylated

48 **N**TE -1.2741531 Non-glycosylated

52 **N**SV -0.83972275 Non-glycosylated

**62 NGS 1.1882916 Potential Glycosylated**

68 **N**HA -0.89887659 Non-glycosylated

81 **N**GI -1.2423291 Non-glycosylated

84 **N**PR -1.8662045 Non-glycosylated

106 **N**YL -0.7359858 Non-glycosylated

112 **N**AG -0.84383581 Non-glycosylated

116 **N**TV -0.70783397 Non-glycosylated

155 **N**LV -1.0444821 Non-glycosylated

200 **N**TV -1.0299987 Non-glycosylated

**225 NNT 0.88963983 Potential Glycosylated**

226 **N**TG -1.0135771 Non-glycosylated

274 **N**LF -1.2838351 Non-glycosylated

329 **N**RL -1.0665658 Non-glycosylated

338 **N**AE -1.1315083 Non-glycosylated

352 **N**TD -1.3580699 Non-glycosylated

**374 NGT 1.2707122 Potential Glycosylated**

385 **N**AL -1.0307742 Non-glycosylated

405 **N**AM -1.3410327 Non-glycosylated

***********************************

>Ss527 Length = 413

**Potential N-Linked Glycosylated Sites:**

MHYY**N**KLKFLALASVFSATSAHPTSGHYYDDAESEACL**N**GKAVYVTS**N**TEH**N**SVVAIPIAR**N**GSLLL**N**HATSTATGGRGG**N**GI**N**PRGMPAGPDALFSQGS

ITSAG**N**YLFAV**N**AGS**N**TVTMLAIDEHDPTKVTVVGEPAELPGEFPTTVGASDKF**N**LVCVGLTGAKAGVSCASYSWYGLGPFDELRTFDLHQTTPPHGPT

**N**TVSHVFFSGDQETVFTTVKGDPAV**NN**TGFLAAYPVEHTHSSCYAIPSVSHKGVISSPDGTAVLFGSTPIPDTT**N**LFVTDASFGAAILGIDDYEEASTL

YKTVIPGQDATCWVAICPATHTAFVTDIRM**N**RLVEMSLV**N**AEIIGEPIDLTTF**N**TDPGLTEIRSGGSFVYALSPG**N**GTTEAWITVL**N**ALTKKPVQHALL

TPLGLDR**N**AMGMAILV

------------------------------------------------

Position Residue Score Prediction

------------------------------------------------

5 **N**KL -0.77832304 Non-glycosylated

39 **N**GK -1.1287597 Non-glycosylated

48 **N**TE -1.2741531 Non-glycosylated

52 **N**SV -0.83972275 Non-glycosylated

**62 NGS 1.1882916 Potential Glycosylated**

68 **N**HA -0.89887659 Non-glycosylated

81 **N**GI -1.2423291 Non-glycosylated

84 **N**PR -1.8662045 Non-glycosylated

106 **N**YL -0.7359858 Non-glycosylated

112 **N**AG -0.84383581 Non-glycosylated

116 **N**TV -0.70783397 Non-glycosylated

155 **N**LV -1.0444821 Non-glycosylated

200 **N**TV -1.0299987 Non-glycosylated

**225 NNT 0.88963983 Potential Glycosylated**

226 **N**TG -1.0135771 Non-glycosylated

274 **N**LF -1.2838351 Non-glycosylated

329 **N**RL -1.0665658 Non-glycosylated

338 **N**AE -1.1315083 Non-glycosylated

352 **N**TD -1.3580699 Non-glycosylated

**374 NGT 1.2707122 Potential Glycosylated**

385 **N**AL -1.0307742 Non-glycosylated

405 **N**AM -1.3410327 Non-glycosylated

***********************************

>Ss162 Length = 413

**Potential N-Linked Glycosylated Sites:**

MHYY**N**KLKFLALASVFSATSAHPTSGHYYDDAESEACL**N**GKAVYVTS**N**TEH**N**SVVAIPIAR**N**GSLLL**N**HATSTATGGRGG**N**GI**N**PRGMPAGPDALFSQGS

ITSAG**N**YLFAV**N**AGS**N**TVTMLAIDEHDPTKVTVVGEPAELPGEFPTTVGASDKF**N**LVCVGLTGAKAGVSCASYSWYGLGPFDELRTFDLHQTTPPHGPT

**N**TVSHVFFSGDQETVFTTVKGDPAV**NN**TGFLAAYPVEHTHSSCYAIPSVSHKGVISSPDGTAVLFGSTPIPDTT**N**LFVTDASFGSAILGIDDYEEASTL

YKTVIPGQDATCWVAICPATHTAFVTDIRM**N**RLVEMSLV**N**AEIIGEPIDLTTF**N**TDPGLTEIRSGGSFVYALSPG**N**GTTEAWITVL**N**ALTKKPVQHALL

TPLGLDR**N**AMGMAILV

------------------------------------------------

Position Residue Score Prediction

------------------------------------------------

5 **N**KL -0.77832304 Non-glycosylated

39 **N**GK -1.1287597 Non-glycosylated

48 **N**TE -1.2741531 Non-glycosylated

52 **N**SV -0.83972275 Non-glycosylated

**62 NGS 1.1882916 Potential Glycosylated**

68 **N**HA -0.89887659 Non-glycosylated

81 **N**GI -1.2423291 Non-glycosylated

84 **N**PR -1.8662045 Non-glycosylated

106 **N**YL -0.7359858 Non-glycosylated

112 **N**AG -0.84383581 Non-glycosylated

116 **N**TV -0.70783397 Non-glycosylated

155 **N**LV -1.0444821 Non-glycosylated

200 **N**TV -1.0299987 Non-glycosylated

**225 NNT 0.88963983 Potential Glycosylated**

226 **N**TG -1.0135771 Non-glycosylated

274 **N**LF -1.2727541 Non-glycosylated

329 **N**RL -1.0665658 Non-glycosylated

338 **N**AE -1.1315083 Non-glycosylated

352 **N**TD -1.3580699 Non-glycosylated

**374 NGT 1.2707122 Potential Glycosylated**

385 **N**AL -1.0307742 Non-glycosylated

405 **N**AM -1.3410327 Non-glycosylated

***********************************

>Ss164 Length = 413

**Potential N-Linked Glycosylated Sites:**

MHYY**N**KLKFLALASVFSVTSAHPTSGHYYDDAESEACL**N**GKAVYVTS**N**TEH**N**SVVAIPIAR**N**GSLLL**N**HATSTATGGRGG**N**GI**N**PRGMPAGPDALFSQGS

ITSAG**N**YLFAV**N**AGS**N**TVTMLAIDEHDPTKVTVVGEPAELPGEFPTTVGASDKF**N**LVCVGLTGAKAGVSCASYSWYGLGPFDELRTFDLHQTTPPHGPT

**N**TVSHVFFSGDQETVFTTVKGDPAV**NN**TGFLAAYPVEHIHSSCYAIPSVSHKGVISSPDGTAVLFGSTPIPDTT**N**LFVTDASFGAAILGIDDYEEASTL

YKTVIPGQDATCWVAICPATHTAFVTDIRM**N**RLVEMSLV**N**AEIIGEPIDLTTF**N**TDPGLTEIRSGGSFVYALSPG**N**GTTEAWITVL**N**ALTKKPVQHALL

TPLGLDR**N**AMGMAILV

------------------------------------------------

Position Residue Score Prediction

------------------------------------------------

5 **N**KL -0.77832304 Non-glycosylated

39 **N**GK -1.1287597 Non-glycosylated

48 **N**TE -1.2741531 Non-glycosylated

52 **N**SV -0.83972275 Non-glycosylated

**62 NGS 1.1882916 Potential Glycosylated**

68 **N**HA -0.89887659 Non-glycosylated

81 **N**GI -1.2423291 Non-glycosylated

84 **N**PR -1.8662045 Non-glycosylated

106 **N**YL -0.7359858 Non-glycosylated

112 **N**AG -0.84383581 Non-glycosylated

116 **N**TV -0.70783397 Non-glycosylated

155 **N**LV -1.0444821 Non-glycosylated

200 **N**TV -1.0299987 Non-glycosylated

**225 NNT 0.88963983 Potential Glycosylated**

226 **N**TG -1.0135771 Non-glycosylated

274 **N**LF -1.2838351 Non-glycosylated

329 **N**RL -1.0665658 Non-glycosylated

338 **N**AE -1.1315083 Non-glycosylated

352 **N**TD -1.3580699 Non-glycosylated

**374 NGT 1.2707122 Potential Glycosylated**

385 **N**AL -1.0307742 Non-glycosylated

405 **N**AM -1.3410327 Non-glycosylated

***********************************

>Ss234 Length = 413

**Potential N-Linked Glycosylated Sites:**

MHYY**N**KLKFLALASVFSVTSAHPTSGHYYDDAESEACL**N**GKAVYVTS**N**TEH**N**SVVAIPIAR**N**GSLLL**N**HATSTATGGRGG**N**GI**N**PRGMPAGPDALFSQGS

ITSAG**N**YLFAV**N**AGS**N**TVTMLAIDEHDPTKVTVVGEPAELPGEFPTTVGASDKF**N**LVCVGLTGAKAGVSCASYSWYGLGPFDELRTFDLHQTTPPHGPT

**N**TVSHVFFSGDQETVFTTVKGDPAV**NN**TGFLAAYPVEHIHSSCYAIPSVSHKGVISSPDGTAVLFGSTPIPDTT**N**LFVTDASFGAAILGIDDYEEASTL

YKTVIPGQDATCWVAICPATHTAFVTDIRM**N**RLVEMSLV**N**AEIIGEPIDLTTF**N**TDPGLTEIRSGGSFVYALSPG**N**GTTEAWITVL**N**ALTKKPVQHALL

TPLGLDR**N**AMGMAILV

------------------------------------------------

Position Residue Score Prediction

------------------------------------------------

5 **N**KL -0.77832304 Non-glycosylated

39 **N**GK -1.1287597 Non-glycosylated

48 **N**TE -1.2741531 Non-glycosylated

52 **N**SV -0.83972275 Non-glycosylated

**62 NGS 1.1882916 Potential Glycosylated**

68 **N**HA -0.89887659 Non-glycosylated

81 **N**GI -1.2423291 Non-glycosylated

84 **N**PR -1.8662045 Non-glycosylated

106 **N**YL -0.7359858 Non-glycosylated

112 **N**AG -0.84383581 Non-glycosylated

116 **N**TV -0.70783397 Non-glycosylated

155 **N**LV -1.0444821 Non-glycosylated

200 **N**TV -1.0299987 Non-glycosylated

**225 NNT 0.88963983 Potential Glycosylated**

226 **N**TG -1.0135771 Non-glycosylated

274 **N**LF -1.2838351 Non-glycosylated

329 **N**RL -1.0665658 Non-glycosylated

338 **N**AE -1.1315083 Non-glycosylated

352 **N**TD -1.3580699 Non-glycosylated

**374 NGT 1.2707122 Potential Glycosylated**

385 **N**AL -1.0307742 Non-glycosylated

405 **N**AM -1.3410327 Non-glycosylated

***********************************

>Ss229 Length = 413

**Potential N-Linked Glycosylated Sites:**

MHYY**N**KLKFLALASVFSVTSAHPTSGHYYDDAESEACL**N**GKAVYVTS**N**TEH**N**SVVAIPIAR**N**GSLLL**N**HATSTATGGRGG**N**GI**N**PRGMPAGPDALFSQGS

ITSAG**N**YLFAV**N**AGS**N**TVTMLAIDEHDPTKVTVVGEPAELPGEFPTTVGASDKF**N**LVCVGLTGAKAGVSCASYSWYGLGPFDELRTFDLHQTTPPHGPT

**N**TVSHVFFSGDQETVFTTVKGDPAV**NN**TGFLAAYPVEHIHSSCYAIPSVSHKGVISSPDGTAVLFGSTPIPDTT**N**LFVTDASFGAAILGIDDYEEASTL

YKTVIPGQDATCWVAICPATHTAFVTDIRM**N**RLVEMSLV**N**AEIIGEPIDLTTF**N**TDPGLTEIRSGGSFVYALSPG**N**GTTEAWITVL**N**ALTKKPVQHALL

TPLGLDR**N**AMGMAILV

------------------------------------------------

Position Residue Score Prediction

------------------------------------------------

5 **N**KL -0.77832304 Non-glycosylated

39 **N**GK -1.1287597 Non-glycosylated

48 **N**TE -1.2741531 Non-glycosylated

52 **N**SV -0.83972275 Non-glycosylated

**62 NGS 1.1882916 Potential Glycosylated**

68 **N**HA -0.89887659 Non-glycosylated

81 **N**GI -1.2423291 Non-glycosylated

84 **N**PR -1.8662045 Non-glycosylated

106 **N**YL -0.7359858 Non-glycosylated

112 **N**AG -0.84383581 Non-glycosylated

116 **N**TV -0.70783397 Non-glycosylated

155 **N**LV -1.0444821 Non-glycosylated

200 **N**TV -1.0299987 Non-glycosylated

**225 NNT 0.88963983 Potential Glycosylated**

226 **N**TG -1.0135771 Non-glycosylated

274 **N**LF -1.2838351 Non-glycosylated

329 **N**RL -1.0665658 Non-glycosylated

338 **N**AE -1.1315083 Non-glycosylated

352 **N**TD -1.3580699 Non-glycosylated

**374 NGT 1.2707122 Potential Glycosylated**

385 **N**AL -1.0307742 Non-glycosylated

405 **N**AM -1.3410327 Non-glycosylated

***********************************

>Ss231 Length = 413

**Potential N-Linked Glycosylated Sites:**

MHYY**N**KLKFLALASVFSVTSAHPTSGHYYDDAESEACL**N**GKAVYVTS**N**TEH**N**SVVAIPIAR**N**GSLLL**N**HATSTATGGRGG**N**GI**N**PRGMPAGPDALFSQGS

ITSAG**N**YLFAV**N**AGS**N**TVTMLAIDEHDPTKVTVVGEPAELPGEFPTTVGASDKF**N**LVCVGLTGAKAGVSCASYSWYGLGPFDELRTFDLHQTTPPHGPT

**N**TVSHVFFSGDQETVFTTVKGDPAV**NN**TGFLAAYPVEHIHSSCYAIPSVSHKGVISSPDGTAVLFGSTPIPDTT**N**LFVTDASFGAAILGIDDYEEASTL

YKTVIPGQDATCWVAICPATHTAFVTDIRM**N**RLVEMSLV**N**AEIIGEPIDLTTF**N**TDPGLTEIRSGGSFVYALSPG**N**GTTEAWITVL**N**ALTKKPVQHALL

TPLGLDR**N**AMGMAILV

------------------------------------------------

Position Residue Score Prediction

------------------------------------------------

5 **N**KL -0.77832304 Non-glycosylated

39 **N**GK -1.1287597 Non-glycosylated

48 **N**TE -1.2741531 Non-glycosylated

52 **N**SV -0.83972275 Non-glycosylated

**62 NGS 1.1882916 Potential Glycosylated**

68 **N**HA -0.89887659 Non-glycosylated

81 **N**GI -1.2423291 Non-glycosylated

84 **N**PR -1.8662045 Non-glycosylated

106 **N**YL -0.7359858 Non-glycosylated

112 **N**AG -0.84383581 Non-glycosylated

116 **N**TV -0.70783397 Non-glycosylated

155 **N**LV -1.0444821 Non-glycosylated

200 **N**TV -1.0299987 Non-glycosylated

**225 NNT 0.88963983 Potential Glycosylated**

226 **N**TG -1.0135771 Non-glycosylated

274 **N**LF -1.2838351 Non-glycosylated

329 **N**RL -1.0665658 Non-glycosylated

338 **N**AE -1.1315083 Non-glycosylated

352 **N**TD -1.3580699 Non-glycosylated

**374 NGT 1.2707122 Potential Glycosylated**

385 **N**AL -1.0307742 Non-glycosylated

405 **N**AM -1.3410327 Non-glycosylated

***********************************

>Ss228 Length = 413

**Potential N-Linked Glycosylated Sites:**

MHYY**N**KLKFLALASVFSVTSAHPTSGHYYDDAESEACL**N**GKAVYVTS**N**TEH**N**SVVAIPIAR**N**GSLLL**N**HATSTATGGRGG**N**GI**N**PRGMPAGPDALFSQGS

ITSAG**N**YLFAV**N**AGS**N**TVTMLAIDEHDPTKVTVVGEPAELPGEFPTTVGASDKF**N**LVCVGLTGAKAGVSCASYSWYGLGPFDELRTFDLHQTTPPHGPT

**N**TVSHVFFSGDQETVFTTVKGDPAV**NN**TGFLAAYPVEHIHSSCYAIPSVSHKGVISSPDGTAVLFGSTPIPDTT**N**LFVTDASFGAAILGIDDYEEASTL

YKTVIPGQDATCWVAICPATHTAFVTDIRM**N**RLVEMSLV**N**AEIIGEPIDLTTF**N**TDPGLTEIRSGGSFVYALSPG**N**GTTEAWITVL**N**ALTKKPVQHALL

TPLGLDR**N**AMGMAILV

------------------------------------------------

Position Residue Score Prediction

------------------------------------------------

5 **N**KL -0.77832304 Non-glycosylated

39 **N**GK -1.1287597 Non-glycosylated

48 **N**TE -1.2741531 Non-glycosylated

52 **N**SV -0.83972275 Non-glycosylated

**62 NGS 1.1882916 Potential Glycosylated**

68 **N**HA -0.89887659 Non-glycosylated

81 **N**GI -1.2423291 Non-glycosylated

84 **N**PR -1.8662045 Non-glycosylated

106 **N**YL -0.7359858 Non-glycosylated

112 **N**AG -0.84383581 Non-glycosylated

116 **N**TV -0.70783397 Non-glycosylated

155 **N**LV -1.0444821 Non-glycosylated

200 **N**TV -1.0299987 Non-glycosylated

**225 NNT 0.88963983 Potential Glycosylated**

226 **N**TG -1.0135771 Non-glycosylated

274 **N**LF -1.2838351 Non-glycosylated

329 **N**RL -1.0665658 Non-glycosylated

338 **N**AE -1.1315083 Non-glycosylated

352 **N**TD -1.3580699 Non-glycosylated

**374 NGT 1.2707122 Potential Glycosylated**

385 **N**AL -1.0307742 Non-glycosylated

405 **N**AM -1.3410327 Non-glycosylated

***********************************

>Ss577 Length = 413

**Potential N-Linked Glycosylated Sites:**

MHYY**N**KLKFLALASVFSATSAHPTSGHYYDDAESEACL**N**GKAVYVTS**N**TEH**N**SVVAIPIAR**N**GSLLL**N**HATSTATGGRGG**N**GI**N**PRGMPAGPDALFSQGS

ITSAG**N**YLFAV**N**AGS**N**TVTMLAIDEHDPTKVTVVGEPAALPGEFPTTVGASDKF**N**LVCVGLTGAKAGVSCASYSWYGLGPFDELRTFDLHQTTPPHGPT

**N**TVSHVFFSGDQETVFTTVKGDPAV**NN**TGFLAAYPVEHTHSSCYAIPSVSHKGVISSPDGTAVLFGSTPIPDTT**N**LFVTDASFGAAILGIDDYEEASTL

YKTVIPGQDATCWVAICPATQTAFVTDIRM**N**RLVEMSLV**N**AEIIGEPIDLTTF**N**TDPGLTEIRSGGSFVYALSPG**N**GTTEAWITVL**N**ALTKKPVQHALL

TPLGLDR**N**AMGMAILV

------------------------------------------------

Position Residue Score Prediction

------------------------------------------------

5 **N**KL -0.77832304 Non-glycosylated

39 **N**GK -1.1287597 Non-glycosylated

48 **N**TE -1.2741531 Non-glycosylated

52 **N**SV -0.83972275 Non-glycosylated

**62 NGS 1.1882916 Potential Glycosylated**

68 **N**HA -0.89887659 Non-glycosylated

81 **N**GI -1.2423291 Non-glycosylated

84 **N**PR -1.8662045 Non-glycosylated

106 **N**YL -0.7359858 Non-glycosylated

112 **N**AG -0.84383581 Non-glycosylated

116 **N**TV -0.70783397 Non-glycosylated

155 **N**LV -1.0444821 Non-glycosylated

200 **N**TV -1.0299987 Non-glycosylated

**225 NNT 0.88963983 Potential Glycosylated**

226 **N**TG -1.0135771 Non-glycosylated

274 **N**LF -1.2838351 Non-glycosylated

329 **N**RL -1.0615395 Non-glycosylated

338 **N**AE -1.1315083 Non-glycosylated

352 **N**TD -1.3580699 Non-glycosylated

**374 NGT 1.2707122 Potential Glycosylated**

385 **N**AL -1.0307742 Non-glycosylated

405 **N**AM -1.3410327 Non-glycosylated

***********************************

>Ss554 Length = 413

**Potential N-Linked Glycosylated Sites:**

MHYY**N**KLKFLALTSVISVTSAHPTSGHYYDDAESEACL**N**GKAVYVTS**N**TEH**N**SVVAIPIAR**N**GSLLL**N**HATSTATGGRGG**N**GI**N**PRGMPACPDALFSQGS

ITSAG**N**YLFAV**N**AGS**N**TVTMLAIDEHDPTKVTVVGEPAELPGEFPTTVGASDKF**N**LVCVGLTGAKAGVSCASYSWYGLGPFDELRTFDLHQTTPPHGPT

**N**TVSHVFFSGDQETVFTTVKGDPAV**NN**TGFLAAYPVEHTHSSCYAIPSVSHKGVISSPDGTAVLFGSTPIPDTT**N**LFVTDASFGAAILGIDDYEEASTL

YKTVIPGQDATCWVAICPATHTAFVTDIRM**N**RLVEMSLV**N**AEIIGEPIDLTTF**N**TDPGLTEIRSGGSFVYALSPG**N**GTTEAWITVL**N**ALTKKPVQHALL

TPLGLDR**N**AMGMAILV

------------------------------------------------

Position Residue Score Prediction

------------------------------------------------

5 **N**KL -0.7253544 Non-glycosylated

39 **N**GK -1.1287597 Non-glycosylated

48 **N**TE -1.2741531 Non-glycosylated

52 **N**SV -0.83972275 Non-glycosylated

**62 NGS 1.1882916 Potential Glycosylated**

68 **N**HA -0.89887659 Non-glycosylated

81 **N**GI -1.1868748 Non-glycosylated

84 **N**PR -1.8738794 Non-glycosylated

106 **N**YL -0.7359858 Non-glycosylated

112 **N**AG -0.84383581 Non-glycosylated

116 **N**TV -0.70783397 Non-glycosylated

155 **N**LV -1.0444821 Non-glycosylated

200 **N**TV -1.0299987 Non-glycosylated

**225 NNT 0.88963983 Potential Glycosylated**

226 **N**TG -1.0135771 Non-glycosylated

274 **N**LF -1.2838351 Non-glycosylated

329 **N**RL -1.0665658 Non-glycosylated

338 **N**AE -1.1315083 Non-glycosylated

352 **N**TD -1.3580699 Non-glycosylated

**374 NGT 1.2707122 Potential Glycosylated**

385 **N**AL -1.0307742 Non-glycosylated

405 **N**AM -1.3410327 Non-glycosylated

***********************************

>Ss447 Length = 413

**Potential N-Linked Glycosylated Sites:**

MHYY**N**KLKFLALASVFSVTSAHPTSGHYYDDAESEACL**N**GKAVYVTS**N**TEH**N**SVVAIPIAR**N**GSLLL**N**HATSTATGGRGG**N**GI**N**PRGMPAGPDALFSQGS

ITSAG**N**YLFAV**N**AGS**N**TVTMLAIDEHDPTKVTVVGEPAKLPGEFPTTVGASDKF**N**LVCVGLTGAKAGVSCASYSWYGLGPFDELRTFDLHQTTPPHGPT

**N**TVSHVFFSGDQETVFTTVKGDPAV**NN**TGFLAAYPVEHIHSSCYAIPSVSHKGVISSPDGTAVLFGSTPIPDTT**N**LFVTDASFGAAILGIDEYEEASTL

YKTVIPGQDATCWVAICPATHTAFVTDIRM**N**RLVEMSLV**N**AEIIGEPIDLTTF**N**TDPGLTEIRSGGSFVYALSPG**N**GTTEAWITVL**N**ALTKKPVQHALL

TPLGLDR**N**AMGMAILV

------------------------------------------------

Position Residue Score Prediction

------------------------------------------------

5 **N**KL -0.77832304 Non-glycosylated

39 **N**GK -1.1287597 Non-glycosylated

48 **N**TE -1.2741531 Non-glycosylated

52 **N**SV -0.83972275 Non-glycosylated

**62 NGS 1.1882916 Potential Glycosylated**

68 **N**HA -0.89887659 Non-glycosylated

81 **N**GI -1.2423291 Non-glycosylated

84 **N**PR -1.8662045 Non-glycosylated

106 **N**YL -0.7359858 Non-glycosylated

112 **N**AG -0.84383581 Non-glycosylated

116 **N**TV -0.70783397 Non-glycosylated

155 **N**LV -1.0444821 Non-glycosylated

200 **N**TV -1.0299987 Non-glycosylated

**225 NNT 0.88963983 Potential Glycosylated**

226 **N**TG -1.0135771 Non-glycosylated

274 **N**LF -1.2838351 Non-glycosylated

329 **N**RL -1.0665658 Non-glycosylated

338 **N**AE -1.1315083 Non-glycosylated

352 **N**TD -1.3580699 Non-glycosylated

**374 NGT 1.2707122 Potential Glycosylated**

385 **N**AL -1.0307742 Non-glycosylated

405 **N**AM -1.3410327 Non-glycosylated

***********************************

>Ss175 Length = 413

**Potential N-Linked Glycosylated Sites:**

MHYY**N**KLKFLALASVFSVTSAHPTSGHYYDDAESEACL**N**GKAVYVTS**N**TEH**N**SVVAIPIAR**N**GSLLL**N**HATSTATGGRGG**N**GI**N**PRGMPAGPDALVSQGS

ITSAG**N**YLFAV**N**AGS**N**TVTMLAIDEHDPTKVTVVGEPAELPGEFPTTVGASDKF**N**LVCVGLTGAKAGVSCASYSWYGLGPFDELRTFDLHQTTPPHGPT

**N**TVSHVFFSGDQETVFTTVKGDPAV**NN**TGFLAAYPVEHIHSSCYAIPSVSHKGVISSPDGTAVLFGSTPIPDTT**N**LFVTDASFGAAILGIDDYEEASTL

YKTVIPGQDATCWVAICPATHTAFVTDIRM**N**RLVEMSLV**N**AEIIGEPIDLTTF**N**TDPGLTEIRSGGSFVYALSPG**N**GTTEAWITVL**N**ALTKKPVQYALL

TPLGLDR**N**AMGMAILV

------------------------------------------------

Position Residue Score Prediction

------------------------------------------------

5 **N**KL -0.77832304 Non-glycosylated

39 **N**GK -1.1287597 Non-glycosylated

48 **N**TE -1.2741531 Non-glycosylated

52 **N**SV -0.83972275 Non-glycosylated

**62 NGS 1.1882916 Potential Glycosylated**

68 **N**HA -0.89887659 Non-glycosylated

81 **N**GI -1.2423291 Non-glycosylated

84 **N**PR -1.8662045 Non-glycosylated

106 **N**YL -0.75391601 Non-glycosylated

112 **N**AG -0.84383581 Non-glycosylated

116 **N**TV -0.70783397 Non-glycosylated

155 **N**LV -1.0444821 Non-glycosylated

200 **N**TV -1.0299987 Non-glycosylated

**225 NNT 0.88963983 Potential Glycosylated**

226 **N**TG -1.0135771 Non-glycosylated

274 **N**LF -1.2838351 Non-glycosylated

329 **N**RL -1.0665658 Non-glycosylated

338 **N**AE -1.1315083 Non-glycosylated

352 **N**TD -1.3580699 Non-glycosylated

**374 NGT 1.2707122 Potential Glycosylated**

385 **N**AL -1.1293307 Non-glycosylated

405 **N**AM -1.3410327 Non-glycosylated

***********************************

>S24 Length = 413

**Potential N-Linked Glycosylated Sites:**

MHYY**N**KLKFLTLASVFSVTSAHPTSGHYYDDAESEACL**N**GKAVYVTS**N**TEH**N**SVVAIPIAR**N**GSLLL**N**HATSTATGGRGG**N**GI**N**PRGMPAGPDALFSQGS

ITSAG**N**YLFAV**N**AGS**N**TVTMLAIDEHDPTKVTVVGEPAELPGEFPTTVGASDKF**N**LVCVGLTGAKAGVSCASYSWYGLGPFDELRTFDLHQTTPPHGPT

**N**TVSHVFFSGDQETVFTTVKGDPAV**NN**TGFLAAYPVEHIHSSCYAIPSVSHKGVISTPDGTAVLFGSTPIPDTT**N**LFVTDASFGAAILGIDDYEEASTL

YKTVIPGQDATCWVAICPATHTAFVTDIRM**N**RLVELSLV**N**AEIIGEPIDLTTF**N**TDPGLTEIRSGGSFVYALSPG**N**GTTEAWITVL**N**ALTKKPVQHALL

TPLGLDR**N**AMGMAILV

------------------------------------------------

Position Residue Score Prediction

------------------------------------------------

5 **N**KL -0.71806834 Non-glycosylated

39 **N**GK -1.1287597 Non-glycosylated

48 **N**TE -1.2741531 Non-glycosylated

52 **N**SV -0.83972275 Non-glycosylated

**62 NGS 1.1882916 Potential Glycosylated**

68 **N**HA -0.89887659 Non-glycosylated

81 **N**GI -1.2423291 Non-glycosylated

84 **N**PR -1.8662045 Non-glycosylated

106 **N**YL -0.7359858 Non-glycosylated

112 **N**AG -0.84383581 Non-glycosylated

116 **N**TV -0.70783397 Non-glycosylated

155 **N**LV -1.0444821 Non-glycosylated

200 **N**TV -1.0299987 Non-glycosylated

**225 NNT 0.88963983 Potential Glycosylated**

226 **N**TG -1.0135771 Non-glycosylated

274 **N**LF -1.2838351 Non-glycosylated

329 **N**RL -1.1071936 Non-glycosylated

338 **N**AE -1.1421628 Non-glycosylated

352 **N**TD -1.3580699 Non-glycosylated

**374 NGT 1.2707122 Potential Glycosylated**

385 **N**AL -1.0307742 Non-glycosylated

405 **N**AM -1.3410327 Non-glycosylated

***********************************

>S13 Length = 413

**Potential N-Linked Glycosylated Sites:**

MHYY**N**KLKFLTLASVFSVTSAHPTSGHYYDDAESEACL**N**GKAVYVTS**N**TEH**N**SVVAIPIAR**N**GSLLL**N**HATSTATGGRGG**N**GI**N**PRGMPAGPDALFSQGS

ITSAG**N**YLFAV**N**AGS**N**TVTMLAIDEHDPTKVTVVGEPAELPGEFPTTVGASDKF**N**LVCVGLTGAKAGVSCASYSWYGLGPFDELRTFDLHQTTPPHGPT

**N**TVSHVFFSGDQETVFTTVKGDPAV**NN**TGFLAAYPVEHIHSSCYAIPSVSHKGVISTPDGTAVLFGSTPIPDTT**N**LFVTDASFGAAILGIDDYEEASTL

YKTVIPGQDATCWVAICPATHTAFVTDIRM**N**RLVELSLV**N**AEIIGEPIDLTTF**N**TDPGLTEIRSGGSFVYALSPG**N**GTTEAWITVL**N**ALTKKPVQHALL

TPLGLDR**N**AMGMAILV

------------------------------------------------

Position Residue Score Prediction

------------------------------------------------

5 **N**KL -0.71806834 Non-glycosylated

39 **N**GK -1.1287597 Non-glycosylated

48 **N**TE -1.2741531 Non-glycosylated

52 **N**SV -0.83972275 Non-glycosylated

**62 NGS 1.1882916 Potential Glycosylated**

68 **N**HA -0.89887659 Non-glycosylated

81 **N**GI -1.2423291 Non-glycosylated

84 **N**PR -1.8662045 Non-glycosylated

106 **N**YL -0.7359858 Non-glycosylated

112 **N**AG -0.84383581 Non-glycosylated

116 **N**TV -0.70783397 Non-glycosylated

155 **N**LV -1.0444821 Non-glycosylated

200 **N**TV -1.0299987 Non-glycosylated

**225 NNT 0.88963983 Potential Glycosylated**

226 **N**TG -1.0135771 Non-glycosylated

274 **N**LF -1.2838351 Non-glycosylated

329 **N**RL -1.1071936 Non-glycosylated

338 **N**AE -1.1421628 Non-glycosylated

352 **N**TD -1.3580699 Non-glycosylated

**374 NGT 1.2707122 Potential Glycosylated**

385 **N**AL -1.0307742 Non-glycosylated

405 **N**AM -1.3410327 Non-glycosylated

***********************************

>Ss564 Length = 413

**Potential N-Linked Glycosylated Sites:**

MHYY**N**KLKFLALASVFSVTSAHPTSGHYYDDAESEACL**N**GKAVYVTS**N**TEH**N**SVVAIPIAR**N**GSLLL**N**HATSTATGGRGG**N**GI**N**PRGMPAGPDALFSQGS

ITSAG**N**YLFAV**N**AGS**N**TVTMLAIDEHDPTKVTVVGEPAELPGEFPTTVGASDKF**N**LVCVGLTGAKAGVSCASYSWYGLGPFDELRTFDLHQTTPPHGPT

**N**TVSHVFFSGDQETVFTTVKGDPAV**NN**TGFLAAYPVEHIHSSCYAIPSVSHKGVISSPDGTAILFGSTPIPDTT**N**LFVTDASFGAAILGIDDYEEASTL

YKTVIPGQDATCWVAICPATHTAFVTDIRM**N**RLVEMSLV**N**AEIIGEPIDLTTF**N**TDPGLTEIRSGGSFVYALSPG**N**GTTEAWITVL**N**ALTKKPVQHALL

TPLGLDR**N**AMGMAILV

------------------------------------------------

Position Residue Score Prediction

------------------------------------------------

5 **N**KL -0.77832304 Non-glycosylated

39 **N**GK -1.1287597 Non-glycosylated

48 **N**TE -1.2741531 Non-glycosylated

52 **N**SV -0.83972275 Non-glycosylated

**62 NGS 1.1882916 Potential Glycosylated**

68 **N**HA -0.89887659 Non-glycosylated

81 **N**GI -1.2423291 Non-glycosylated

84 **N**PR -1.8662045 Non-glycosylated

106 **N**YL -0.7359858 Non-glycosylated

112 **N**AG -0.84383581 Non-glycosylated

116 **N**TV -0.70783397 Non-glycosylated

155 **N**LV -1.0444821 Non-glycosylated

200 **N**TV -1.0299987 Non-glycosylated

**225 NNT 0.88963983 Potential Glycosylated**

226 **N**TG -1.0135771 Non-glycosylated

274 **N**LF -1.2838351 Non-glycosylated

329 **N**RL -1.0665658 Non-glycosylated

338 **N**AE -1.1315083 Non-glycosylated

352 **N**TD -1.3580699 Non-glycosylated

**374 NGT 1.2707122 Potential Glycosylated**

385 **N**AL -1.0307742 Non-glycosylated

405 **N**AM -1.3410327 Non-glycosylated

***********************************

>Ss567 Length = 413

**Potential N-Linked Glycosylated Sites:**

MHYY**N**KLKFLALASVFSVTSAHPTSGHYYDDAESEACL**N**GKAVYVTS**N**TEH**N**SVVAIPIAR**N**GSLLL**N**HATSTATGGRGG**N**GI**N**PRGMPAGPDALFSQGS

ITSAG**N**YLFAV**N**AGS**N**TVTMLAIDEHDPTKVTVVGEPAELPGEFPTTVGASDKF**N**LVCVGLTGAKAGVSCASYSWYGLGPFDELRTFDLHQTTPPHGPT

**N**TVSHVFFSGDQETVFTTVKGDPAV**NN**TGFLAAYPVEHIHSSCYAIPSVSHKGVISSPDGTAILFGSTPIPDTT**N**LFVTDASFGAAILGIDDYEEASTL

YKTVIPGQDATCWVAICPATHTAFVTDIRM**N**RLVEMSLV**N**AEIIGEPIDLTTF**N**TDPGLTEIRSGGSFVYALSPG**N**GTTEAWITVL**N**ALTKKPVQHALL

TPLGLDR**N**AMGMAILV

------------------------------------------------

Position Residue Score Prediction

------------------------------------------------

5 **N**KL -0.77832304 Non-glycosylated

39 **N**GK -1.1287597 Non-glycosylated

48 **N**TE -1.2741531 Non-glycosylated

52 **N**SV -0.83972275 Non-glycosylated

**62 NGS 1.1882916 Potential Glycosylated**

68 **N**HA -0.89887659 Non-glycosylated

81 **N**GI -1.2423291 Non-glycosylated

84 **N**PR -1.8662045 Non-glycosylated

106 **N**YL -0.7359858 Non-glycosylated

112 **N**AG -0.84383581 Non-glycosylated

116 **N**TV -0.70783397 Non-glycosylated

155 **N**LV -1.0444821 Non-glycosylated

200 **N**TV -1.0299987 Non-glycosylated

**225 NNT 0.88963983 Potential Glycosylated**

226 **N**TG -1.0135771 Non-glycosylated

274 **N**LF -1.2838351 Non-glycosylated

329 **N**RL -1.0665658 Non-glycosylated

338 **N**AE -1.1315083 Non-glycosylated

352 **N**TD -1.3580699 Non-glycosylated

**374 NGT 1.2707122 Potential Glycosylated**

385 **N**AL -1.0307742 Non-glycosylated

405 **N**AM -1.3410327 Non-glycosylated

***********************************

>Ss59 Length = 413

**Potential N-Linked Glycosylated Sites:**

MHYY**N**KLKFLALASDFLRTSAHPTSGHYYDDAESEACL**N**GKAVYVTS**N**TEH**N**SVVAIPIAR**N**GSLLL**N**HATSTATGGRGG**N**GI**N**PRGMPAGPDALFSQGS

ITSAG**N**YLFAV**N**AGS**N**TVTMLAIDEHDPTKVTVVGEPAELPGEFPTTVGASDKF**N**LVCVGLTGAKAGVSCASYSWYGLGPFDELRTFDLHQTTPPHGPT

**N**TVSHVFFSGDQETVFTTVKGDPAV**NN**TGFLAAYPVEHIHSSCYAIPSVSHKGVISSPDGTAVLFGSTPIPDTT**N**LFVTDASFGAAILGIDDYEEASTL

YKTVIPGQDATCWVAICPATHTAFVTDIRM**N**RLVEMSLV**N**AEIIGEPIDLTTF**N**TDPGLTEIRSGGSFVYALSPG**N**GTTEAWITVL**N**ALTK**N**PVQYALL

TPLGLDR**N**AMGMAILV

------------------------------------------------

Position Residue Score Prediction

------------------------------------------------

5 **N**KL -0.72731404 Non-glycosylated

39 **N**GK -1.1287597 Non-glycosylated

48 **N**TE -1.2741531 Non-glycosylated

52 **N**SV -0.83972275 Non-glycosylated

**62 NGS 1.1882916 Potential Glycosylated**

68 **N**HA -0.89887659 Non-glycosylated

81 **N**GI -1.2423291 Non-glycosylated

84 **N**PR -1.8662045 Non-glycosylated

106 **N**YL -0.7359858 Non-glycosylated

112 **N**AG -0.84383581 Non-glycosylated

116 **N**TV -0.70783397 Non-glycosylated

155 **N**LV -1.0444821 Non-glycosylated

200 **N**TV -1.0299987 Non-glycosylated

**225 NNT 0.88963983 Potential Glycosylated**

226 **N**TG -1.0135771 Non-glycosylated

274 **N**LF -1.2838351 Non-glycosylated

329 **N**RL -1.0665658 Non-glycosylated

338 **N**AE -1.1315083 Non-glycosylated

352 **N**TD -1.3580699 Non-glycosylated

**374 NGT 1.2707122 Potential Glycosylated**

385 **N**AL -1.0571189 Non-glycosylated

390 **N**PV -1.007434 Non-glycosylated

405 **N**AM -1.3410327 Non-glycosylated

***********************************

>Ss551 Length = 413

**Potential N-Linked Glycosylated Sites:**

MHYY**N**KLKFLALASDFLRTSAHPTSGHYYDDAESEACL**N**GKAVYVTSTTEH**N**SVVAIPIAR**N**GSLLL**N**HATSTATGGRGG**N**GI**N**PRGMPAGPDALFSQGS

ITSAG**N**YLFAV**N**AGS**N**TVTMLAIDEHDPTKVTVVGEPAELPGEFPTTVGASDKF**N**LVCVGLTGAKAGVSCASYSWYGLGPFDELRTFDLHQTTPPHGPT

**N**TVSHVFFSGDQETVFTTVKGDPAV**NN**TGFLAAYPVEHTHSSCYAIPSVSHKGVISSPDGTAVLFGSTPIPDTT**N**LFVTDASFGAAILGIDDYEEASTL

YKTVIPGQDATCWVAICPATHTAFVTDIRM**N**RLVEMSLV**N**AEIIGEPIDLTTF**N**TDPGLTEIRSGGSFVYALSPG**N**GTTEAWITVL**N**ALTKRPVQHGIL

TPLGLDR**N**AMGMAILV

------------------------------------------------

Position Residue Score Prediction

------------------------------------------------

5 **N**KL -0.72731404 Non-glycosylated

39 **N**GK -1.0437187 Non-glycosylated

52 **N**SV -0.91612523 Non-glycosylated

**62 NGS 1.1882916 Potential Glycosylated**

68 **N**HA -0.89887659 Non-glycosylated

81 **N**GI -1.2423291 Non-glycosylated

84 **N**PR -1.8662045 Non-glycosylated

106 **N**YL -0.7359858 Non-glycosylated

112 **N**AG -0.84383581 Non-glycosylated

116 **N**TV -0.70783397 Non-glycosylated

155 **N**LV -1.0444821 Non-glycosylated

200 **N**TV -1.0299987 Non-glycosylated

**225 NNT 0.88963983 Potential Glycosylated**

226 **N**TG -1.0135771 Non-glycosylated

274 **N**LF -1.2838351 Non-glycosylated

329 **N**RL -1.0665658 Non-glycosylated

338 **N**AE -1.1315083 Non-glycosylated

352 **N**TD -1.3580699 Non-glycosylated

**374 NGT 1.2707122 Potential Glycosylated**

385 **N**AL -1.1227668 Non-glycosylated

405 **N**AM -1.2786885 Non-glycosylated

***********************************

>Ss13 Length = 413

**Potential N-Linked Glycosylated Sites:**

MHYY**N**KLKFLALASVISVTSAHPTSGHYYADAESEACL**N**GKAVYVTS**N**TEH**N**SVVAIPIAR**N**GSLLL**N**HATSTATGGRGG**N**GI**N**PRGMPAGPDALFSQGS

ITIAGDYLFAV**N**SGS**N**TVTMLAIDEHDPTKVTVVGEPAQLPGEFPTTVGASDKF**N**LVCVGLTGAKAGVSCASYSWYGLGPFDELRTFDLHQTTPPHGPT

**N**TVSHVFFSGDQETVFTTVKGDPAV**NN**TGFLAAYPVEDIHSSCYAIPSVSHKGFISSPDGTAVLFGSTPIPDTT**N**LFVTDASFGAAILGIDDYEEASTL

YKTVIPGQDATCWVAICPATHTAFVTDIRM**N**RLVEMSLA**N**AEIIGEPIDLTTF**NN**DPGLTEIRSGGSFVYALSPG**N**GTTEAWITVL**N**ALTKKPVQHALL

TPLGLDR**N**AMGMAILV

------------------------------------------------

Position Residue Score Prediction

------------------------------------------------

5 **N**KL -0.77832304 Non-glycosylated

39 **N**GK -1.130584 Non-glycosylated

48 **N**TE -1.2741531 Non-glycosylated

52 **N**SV -0.83972275 Non-glycosylated

**62 NGS 1.1882916 Potential Glycosylated**

68 **N**HA -0.89887659 Non-glycosylated

81 **N**GI -1.2423291 Non-glycosylated

84 **N**PR -1.8662045 Non-glycosylated

112 **N**SG -1.1057068 Non-glycosylated

116 **N**TV -0.90010362 Non-glycosylated

155 **N**LV -1.0444821 Non-glycosylated

200 **N**TV -1.0299987 Non-glycosylated

**225 NNT 0.88963983 Potential Glycosylated**

226 **N**TG -1.0135771 Non-glycosylated

274 **N**LF -1.2838351 Non-glycosylated

329 **N**RL -1.0222076 Non-glycosylated

338 **N**AE -1.1197133 Non-glycosylated

352 **NN**D -1.2714523 Non-glycosylated

353 **N**DP -1.1516042 Non-glycosylated

**374 NGT 1.2707122 Potential Glycosylated**

385 **N**AL -1.0307742 Non-glycosylated

405 **N**AM -1.3410327 Non-glycosylated

***********************************

>Ss124 Length = 413

**Potential N-Linked Glycosylated Sites:**

MHYY**N**KLKFLALASVISVTSAHPTSGHYYADAESEACL**N**GKAVYVTS**N**TEH**N**SVVAIPIAR**N**GSLLL**N**HATSTATGGRGG**N**GI**N**PRGMPAGPDALFSQGS

ITIAGDYLFAV**N**AGS**N**TVTMLAIDEHDPTKVTVVGEPAQLPGEFPTTVGASDKF**N**LVCVGLTGAKAGVSCASYSWYGLGPFDELRTFDLHQTTPPHGPT

**N**TVSHVFFSGDQETVFTTVKGDPAV**NN**TGFLAAYPVEDIHSSCYAIPSVSHKGVISSPDGTAVLFGSTPIPDTT**N**LFVTDASFGAAILGIDDYEEASTL

YKTVIPGQDATCWVAICPATHTAFVTDIRM**N**RLVEMSLA**N**AEIIGEPIDLTTF**NN**DPGLTEIRSGGSFVYALSPG**N**GTTEAWITVL**N**ALTKKPVQHALL

TPLGLDR**N**AMGMAILV

------------------------------------------------

Position Residue Score Prediction

------------------------------------------------

5 **N**KL -0.77832304 Non-glycosylated

39 **N**GK -1.130584 Non-glycosylated

48 **N**TE -1.2741531 Non-glycosylated

52 **N**SV -0.83972275 Non-glycosylated

**62 NGS 1.1882916 Potential Glycosylated**

68 **N**HA -0.89887659 Non-glycosylated

81 **N**GI -1.2423291 Non-glycosylated

84 **N**PR -1.8662045 Non-glycosylated

112 **N**AG -1.0674394 Non-glycosylated

116 **N**TV -0.71392911 Non-glycosylated

155 **N**LV -1.0444821 Non-glycosylated

200 **N**TV -1.0299987 Non-glycosylated

**225 NNT 0.88963983 Potential Glycosylated**

226 **N**TG -1.0135771 Non-glycosylated

274 **N**LF -1.2838351 Non-glycosylated

329 **N**RL -1.0222076 Non-glycosylated

338 **N**AE -1.1197133 Non-glycosylated

352 **NN**D -1.2714523 Non-glycosylated

353 **N**DP -1.1516042 Non-glycosylated

**374 NGT 1.2707122 Potential Glycosylated**

385 **N**AL -1.0307742 Non-glycosylated

405 **N**AM -1.3410327 Non-glycosylated

***********************************

>Ss137 Length = 413

**Potential N-Linked Glycosylated Sites:**

MHYY**N**KLKFLALASVISVTSAHPTSGHYYADAESEACL**N**GKAVYVTS**N**TEH**N**SVVAIPIAR**N**GSLLL**N**HATSTATGGRGG**N**GI**N**PRGMPAGPDALFSQGS

ITIAGDYLFAV**N**AGS**N**TVTMLAIDEHDPTKVTVVGEPAQLPGEFPTTVGASDKF**N**LVCVGLTGAKAGVSCASYSWYGLGPFDELRTFDLHQTTPPHGPT

**N**TVSHVFFSGDQETVFTTVKGDPAV**NN**TGFLAAYPVEDIHSSCYAIPSVSHKGVISSPDGTAVLFGSTPIPDTT**N**LFVTDASFGAAILGIDDYEEASTL

YKTVIPGQDATCWVAICPDTHTAFVTDIRM**N**RLVEMSLA**N**AEIIGEPIDLTTF**NN**DPGLTEIRSGGSFVYALSPG**N**GTTEAWITVL**N**ALTKKPVQHALL

TPLGLDR**N**AMGMAILV

------------------------------------------------

Position Residue Score Prediction

------------------------------------------------

5 **N**KL -0.77832304 Non-glycosylated

39 **N**GK -1.130584 Non-glycosylated

48 **N**TE -1.2741531 Non-glycosylated

52 **N**SV -0.83972275 Non-glycosylated

**62 NGS 1.1882916 Potential Glycosylated**

68 **N**HA -0.89887659 Non-glycosylated

81 **N**GI -1.2423291 Non-glycosylated

84 **N**PR -1.8662045 Non-glycosylated

112 **N**AG -1.0674394 Non-glycosylated

116 **N**TV -0.71392911 Non-glycosylated

155 **N**LV -1.0444821 Non-glycosylated

200 **N**TV -1.0299987 Non-glycosylated

**225 NNT 0.88963983 Potential Glycosylated**

226 **N**TG -1.0135771 Non-glycosylated

274 **N**LF -1.2838351 Non-glycosylated

329 **N**RL -1.0222076 Non-glycosylated

338 **N**AE -1.1197133 Non-glycosylated

352 **NN**D -1.2714523 Non-glycosylated

353 **N**DP -1.1516042 Non-glycosylated

**374 NGT 1.2707122 Potential Glycosylated**

385 **N**AL -1.0307742 Non-glycosylated

405 **N**AM -1.3410327 Non-glycosylated

***********************************

>Ss46 Length = 413

**Potential N-Linked Glycosylated Sites:**

MHYY**N**KLKFLALASVISVTSAHPTSGHYYADAESEACL**N**GKAVYVTS**N**TEH**N**SVVAIPIAR**N**GSLLL**N**HATSTATGGRGG**N**GI**N**PRGMPAGPDALFSQGS

ITIAGDYLFAV**N**AGS**N**TVTMLAIDEHDPTKVTVVGEPAQWPGEFPTTVGASDKF**N**LVCVGLTGAKAGVSCASYSWYGLGPFDELRTFDLHQTTPPHGPT

**N**TVSHVFFSGDQETVFTTVKGDPAV**NN**TGFLAAYPVEDIHSSCYAIPSVSHKGVISSPDGTAVLFGSTPIPDTT**N**LFVTDASFGAAILGIDDYEEASTL

YKTVIPGQDATCWVAICPATHTAFVTDIRM**N**RLVEMSLA**N**AEIIGEPIDLTTF**NN**DPGLTEIRSGGSFVYALSPG**N**GTTEAWITVL**N**ALTKKPVQHALL

TPLGLDR**N**AMGMAILV

------------------------------------------------

Position Residue Score Prediction

------------------------------------------------

5 **N**KL -0.77832304 Non-glycosylated

39 **N**GK -1.130584 Non-glycosylated

48 **N**TE -1.2741531 Non-glycosylated

52 **N**SV -0.83972275 Non-glycosylated

**62 NGS 1.1882916 Potential Glycosylated**

68 **N**HA -0.89887659 Non-glycosylated

81 **N**GI -1.2423291 Non-glycosylated

84 **N**PR -1.8662045 Non-glycosylated

112 **N**AG -1.0674394 Non-glycosylated

116 **N**TV -0.71392911 Non-glycosylated

155 **N**LV -1.0444821 Non-glycosylated

200 **N**TV -1.0299987 Non-glycosylated

**225 NNT 0.88963983 Potential Glycosylated**

226 **N**TG -1.0135771 Non-glycosylated

274 **N**LF -1.2838351 Non-glycosylated

329 **N**RL -1.0222076 Non-glycosylated

338 **N**AE -1.1197133 Non-glycosylated

352 **NN**D -1.2714523 Non-glycosylated

353 **N**DP -1.1516042 Non-glycosylated

**374 NGT 1.2707122 Potential Glycosylated**

385 **N**AL -1.0307742 Non-glycosylated

405 **N**AM -1.3410327 Non-glycosylated

***********************************

>Ss17 Length = 413

**Potential N-Linked Glycosylated Sites:**

MHYY**N**KLKFLALASVISVTSAHPTSGHYYADAESEACL**N**GKAVYVTS**N**TEH**N**SVVAIPIAR**N**GSLLL**N**HATSTATGGRGG**N**GI**N**PRGMPAGPDALFSQGS

ITIAGDYLFAV**N**AGS**N**TVTMLAIDEHDPTKVTVVGEPAQLPGEFPTTVGASDKF**N**LVCVGLTGAKAGVSCASYSWYGLGPFDELRTFDLHQTTPPHGPT

**N**TVSHVFFSGDQETVFTTVKGDPAV**NN**TGFLAAYPVEDIHSSCYSIPSVSHKGVISSPDGTAVLFGSTPIPDTT**N**LFVTDASFGAAILGIDDYEEASTL

YKTVIPGQDATCWVAICPATHTAFVTDIRM**N**RLVEMSLA**N**AEIIGEPIDLTTF**NN**DPGLTEIRSGGSFVYALSPG**N**GTTEAWITVL**N**ALTKKPVQHALL

TPLGLDR**N**AMGMAILV

------------------------------------------------

Position Residue Score Prediction

------------------------------------------------

5 **N**KL -0.77832304 Non-glycosylated

39 **N**GK -1.130584 Non-glycosylated

48 **N**TE -1.2741531 Non-glycosylated

52 **N**SV -0.83972275 Non-glycosylated

**62 NGS 1.1882916 Potential Glycosylated**

68 **N**HA -0.89887659 Non-glycosylated

81 **N**GI -1.2423291 Non-glycosylated

84 **N**PR -1.8662045 Non-glycosylated

112 **N**AG -1.0674394 Non-glycosylated

116 **N**TV -0.71392911 Non-glycosylated

155 **N**LV -1.0444821 Non-glycosylated

200 **N**TV -1.0299987 Non-glycosylated

**225 NNT 0.88963983 Potential Glycosylated**

226 **N**TG -1.0135771 Non-glycosylated

274 **N**LF -1.2838351 Non-glycosylated

329 **N**RL -1.0222076 Non-glycosylated

338 **N**AE -1.1197133 Non-glycosylated

352 **NN**D -1.2714523 Non-glycosylated

353 **N**DP -1.1516042 Non-glycosylated

**374 NGT 1.2707122 Potential Glycosylated**

385 **N**AL -1.0307742 Non-glycosylated

405 **N**AM -1.3410327 Non-glycosylated

***********************************

>SsMS1 Length = 413

**Potential N-Linked Glycosylated Sites:**

MHYY**N**KLKFLALASVISVTSAHPTSGHYYADAESEVCL**N**GKAVYVTS**N**TEH**N**SVVAIPIAR**N**GSLLL**N**HATSTATGGRGG**N**GI**N**PRGMPAGPDALFSQGS

ITIAGDYLFAV**N**AGS**N**TVTMLAIDEHDPTKVTVVGEPAQLPGEFPTTVGASDKF**N**LVCVGLTGAKAGVSCASYSWYGLGPFDELRTFDLHQTTPPHGPT

**N**TVSHVFFSGDQETVFTTVKGDPAV**NN**TGFLAAYPVEDIHSSCYSIPSVSHKGVISSPDGTAVLFGSTPIPDTT**N**LFVTDASFGAAILGIDDYEEASTL

YKTVIPGQDATCWVAICPATHTAFVTDIRM**N**RLVEMSLA**N**AEIIGEPIDLTTF**NN**DPGLTEIRSGGSFVYALSPG**N**GTTEAWITVL**N**ALTKKPVQHALL

TPLGLDR**N**AMGMAILV

------------------------------------------------

Position Residue Score Prediction

------------------------------------------------

5 **N**KL -0.77832304 Non-glycosylated

39 **N**GK -1.2587877 Non-glycosylated

48 **N**TE -1.2741531 Non-glycosylated

52 **N**SV -0.83972275 Non-glycosylated

**62 NGS 1.1882916 Potential Glycosylated**

68 **N**HA -0.89887659 Non-glycosylated

81 **N**GI -1.2423291 Non-glycosylated

84 **N**PR -1.8662045 Non-glycosylated

112 **N**AG -1.0674394 Non-glycosylated

116 **N**TV -0.71392911 Non-glycosylated

155 **N**LV -1.0444821 Non-glycosylated

200 **N**TV -1.0299987 Non-glycosylated

**225 NNT 0.88963983 Potential Glycosylated**

226 **N**TG -1.0135771 Non-glycosylated

274 **N**LF -1.2838351 Non-glycosylated

329 **N**RL -1.0222076 Non-glycosylated

338 **N**AE -1.1197133 Non-glycosylated

352 **NN**D -1.2714523 Non-glycosylated

353 **N**DP -1.1516042 Non-glycosylated

**374 NGT 1.2707122 Potential Glycosylated**

385 **N**AL -1.0307742 Non-glycosylated

405 **N**AM -1.3410327 Non-glycosylated

***********************************

>SsEM7 Length = 413

**Potential N-Linked Glycosylated Sites:**

MHYY**N**KLKFLALASVISVTSAHPTSGHYYADAESEACL**N**GKAVYVTS**N**TEH**N**SVVAIPIAR**N**GSLLL**N**HATSTATGGRGG**N**GI**N**PRGMPAGPDALFSQGS

ITIAGDYLFAV**N**AGS**N**TVTMLAIDEHDPTKVTVVGEPAQLPGEFPTTVGASDKF**N**LVCVGLTGAKAGVSCASYSWYGLGPFDELRTFDLHQTTPPHGPT

**N**TVSHVFFSGDQETVFTTVKGDPAV**NN**TGFLAAYPVEDIHSSCYSIPSVSHKGVISSPDGTAVLFGSTPIPDTT**N**LFVTDASFGAAILGIDDYEEASTL

YKTVIPGQDATCWVAICPATHTAFVTDIRM**N**RLVEMSLA**N**AEIIGEPIDLTTF**NN**DPGLTEIRSGGSFVYALSPG**N**GTTEAWITVL**N**ALTKKPVQHALL

TPLGLDR**N**AMGMAILV

------------------------------------------------

Position Residue Score Prediction

------------------------------------------------

5 **N**KL -0.77832304 Non-glycosylated

39 **N**GK -1.130584 Non-glycosylated

48 **N**TE -1.2741531 Non-glycosylated

52 **N**SV -0.83972275 Non-glycosylated

**62 NGS 1.1882916 Potential Glycosylated**

68 **N**HA -0.89887659 Non-glycosylated

81 **N**GI -1.2423291 Non-glycosylated

84 **N**PR -1.8662045 Non-glycosylated

112 **N**AG -1.0674394 Non-glycosylated

116 **N**TV -0.71392911 Non-glycosylated

155 **N**LV -1.0444821 Non-glycosylated

200 **N**TV -1.0299987 Non-glycosylated

**225 NNT 0.88963983 Potential Glycosylated**

226 **N**TG -1.0135771 Non-glycosylated

274 **N**LF -1.2838351 Non-glycosylated

329 **N**RL -1.0222076 Non-glycosylated

338 **N**AE -1.1197133 Non-glycosylated

352 **NN**D -1.2714523 Non-glycosylated

353 **N**DP -1.1516042 Non-glycosylated

**374 NGT 1.2707122 Potential Glycosylated**

385 **N**AL -1.0307742 Non-glycosylated

405 **N**AM -1.3410327 Non-glycosylated

***********************************

>Ss126 Length = 413

**Potential N-Linked Glycosylated Sites:**

MHYY**N**KLKFLALASVIPVTSAHPTSGHYYADAESEACL**N**GKAVYVTS**N**TEH**N**SVVAIPIAR**N**GSLLL**N**HATSTATGGRGG**N**GI**N**PRGMPAGPDALFSQGS

ITIAGDYLFAV**N**AGS**N**TVTMLAIDEHDPTKVTVVGEPAQLPGEFPTTVGASDKF**N**LVCVGLTGAKAGVSCASYSWYGLGPFDELRTFDLHQTTPPHGPT

**N**TVSHVFFSGDQETVFTTVKGDPAV**NN**TGFLAAYPVEDIHSSCYSIPSVSHKGVISSPDGTAVLFGSTPIPDTT**N**LFVTDASFGAAVLGIDDYEEASTL

YKTVIPGQDATCWVAICPATHTAFVTDIRM**N**RLVEMSLA**N**AEIIGEPIDLTTF**NN**DPGLTEIRSGGSFVYALSPG**N**GTTEAWITVL**N**ALTKKPVQHALL

TPLGLDR**N**AMGMAILV

------------------------------------------------

Position Residue Score Prediction

------------------------------------------------

5 **N**KL -0.77832304 Non-glycosylated

39 **N**GK -1.130584 Non-glycosylated

48 **N**TE -1.2741531 Non-glycosylated

52 **N**SV -0.83972275 Non-glycosylated

**62 NGS 1.1882916 Potential Glycosylated**

68 **N**HA -0.89887659 Non-glycosylated

81 **N**GI -1.2423291 Non-glycosylated

84 **N**PR -1.8662045 Non-glycosylated

112 **N**AG -1.0674394 Non-glycosylated

116 **N**TV -0.71392911 Non-glycosylated

155 **N**LV -1.0444821 Non-glycosylated

200 **N**TV -1.0299987 Non-glycosylated

**225 NNT 0.88963983 Potential Glycosylated**

226 **N**TG -1.0135771 Non-glycosylated

274 **N**LF -1.2838351 Non-glycosylated

329 **N**RL -1.0222076 Non-glycosylated

338 **N**AE -1.1197133 Non-glycosylated

352 **NN**D -1.2714523 Non-glycosylated

353 **N**DP -1.1516042 Non-glycosylated

**374 NGT 1.2707122 Potential Glycosylated**

385 **N**AL -1.0307742 Non-glycosylated

405 **N**AM -1.3410327 Non-glycosylated

***********************************

>Ss241 Length = 413

**Potential N-Linked Glycosylated Sites:**

MHYY**N**KLKFLALASVISVTSAHPTSGHYYADAESEACL**N**GKAVYVTS**N**TEH**N**SVVAIPIAR**N**GSLLL**N**HATSTATGGRGG**N**GI**N**PRGMPAGPDALFSQGS

ITIAGDYLFAV**N**AGS**N**TVTMLAIDEHDPTKVTVVGEPAQLPGEFPTTVGASDKF**N**LVCVGLTGAKAGVSCASYSWYGLGPFDELRTFDLHQTTPPHGPT

**N**TVSHVFFSGDQETVFTTVKGDPAV**NN**TGFLAAYPVEDIHSSCYSIPSVSHKGVISSPDGTAVLFGSTPIPDTT**N**LFVTDASFGAAILGIDDYEEASTL

YKTVIPGQDATCWVAICPATHTAFVTDIRM**N**RLVEMSLA**N**AEIIGEPIDLTTF**NN**DPGLTEIRSGGSFVYALSPG**N**GTTEAWITVL**N**ALTKKPVQHALL

TPLGLDR**N**AMGMAILV

------------------------------------------------

Position Residue Score Prediction

------------------------------------------------

5 **N**KL -0.77832304 Non-glycosylated

39 **N**GK -1.130584 Non-glycosylated

48 **N**TE -1.2741531 Non-glycosylated

52 **N**SV -0.83972275 Non-glycosylated

**62 NGS 1.1882916 Potential Glycosylated**

68 **N**HA -0.89887659 Non-glycosylated

81 **N**GI -1.2423291 Non-glycosylated

84 **N**PR -1.8662045 Non-glycosylated

112 **N**AG -1.0674394 Non-glycosylated

116 **N**TV -0.71392911 Non-glycosylated

155 **N**LV -1.0444821 Non-glycosylated

200 **N**TV -1.0299987 Non-glycosylated

**225 NNT 0.88963983 Potential Glycosylated**

226 **N**TG -1.0135771 Non-glycosylated

274 **N**LF -1.2838351 Non-glycosylated

329 **N**RL -1.0222076 Non-glycosylated

338 **N**AE -1.1197133 Non-glycosylated

352 **NN**D -1.2714523 Non-glycosylated

353 **N**DP -1.1516042 Non-glycosylated

**374 NGT 1.2707122 Potential Glycosylated**

385 **N**AL -1.0307742 Non-glycosylated

405 **N**AM -1.3410327 Non-glycosylated

***********************************

>Ss40 Length = 413

**Potential N-Linked Glycosylated Sites:**

MHYY**N**KLKFLALASVISVTSAHPTGGHYYADAESEACL**N**GKAVYVTS**N**TEH**N**SVVAIPIAR**N**GSLLL**N**HATSTATGGRGG**N**GI**N**PRGMPAGPDALFSQGS

ITIAGDYLFAV**N**AGS**N**TVTMLAIDEHDPTKVTVVGEPAQLPGEFPTTVGASDKF**N**LVCVGLTGAKAGVSCASYSWYGLGPFDELRTFDLHQTTPPHGPT

**N**TVSHVFFSGDQETVFTTVKGDPAV**NN**TGFLAAYPVEDIHSSCYSIPSVSHKGVISSPDGTAVLFGSTPIPDTT**N**LFVTDASFGAAILGIDDYEEASTL

YKTVIPGQDATCWVAICPATHTAFVTDIRM**N**RLVEMSLA**N**AEIIGEPIDLTTF**NN**DPGLTEIRSGGSFVYALSPG**N**GTTEAWITVL**N**ALTKKPVQHALL

TPLGLDR**N**AMGMAILV

------------------------------------------------

Position Residue Score Prediction

------------------------------------------------

5 **N**KL -0.77832304 Non-glycosylated

39 **N**GK -1.130584 Non-glycosylated

48 **N**TE -1.2741531 Non-glycosylated

52 **N**SV -0.83972275 Non-glycosylated

**62 NGS 1.1882916 Potential Glycosylated**

68 **N**HA -0.89887659 Non-glycosylated

81 **N**GI -1.2423291 Non-glycosylated

84 **N**PR -1.8662045 Non-glycosylated

112 **N**AG -1.0674394 Non-glycosylated

116 **N**TV -0.71392911 Non-glycosylated

155 **N**LV -1.0444821 Non-glycosylated

200 **N**TV -1.0299987 Non-glycosylated

**225 NNT 0.88963983 Potential Glycosylated**

226 **N**TG -1.0135771 Non-glycosylated

274 **N**LF -1.2838351 Non-glycosylated

329 **N**RL -1.0222076 Non-glycosylated

338 **N**AE -1.1197133 Non-glycosylated

352 **NN**D -1.2714523 Non-glycosylated

353 **N**DP -1.1516042 Non-glycosylated

**374 NGT 1.2707122 Potential Glycosylated**

385 **N**AL -1.0307742 Non-glycosylated

405 **N**AM -1.3410327 Non-glycosylated

***********************************

>Ss243 Length = 413

**Potential N-Linked Glycosylated Sites:**

MHYY**N**KLKFLALASVISVTSAHPTGGHYYADAESEACL**N**GKAVYVTS**N**TEH**N**SVVAIPIAR**N**GSLLL**N**HATSTATGGRGG**N**GI**N**PRGMPAGPDALFSQGS

ITIAGDYLFAV**N**AGS**N**TVTMLAIDEHDPTKVTVVGEPAQLPGEFPTTVGASDKF**N**LVCVGLTGAKAGVSCASYSWYGLGPFDELRTFDLHQTTPPHGPT

**N**TVSHVFFSGDQETVFTTVKGDPAV**NN**TGFLAAYPVEDIHSSCYSIPSVSHKGVISSPDGTAVLFGSTPIPDTT**N**LFVTDASFGAAILGIDDYEEASTL

YKTVIPGQDATCWVAICPATHTAFVTDIRM**N**RLVEMSLA**N**AEIIGEPIDLTTF**NN**DPGLTEIRSGGSFVYALSPG**N**GTTEAWITVL**N**ALTKKPVQHALL

TPLGLDR**N**AMGMAILV

------------------------------------------------

Position Residue Score Prediction

------------------------------------------------

5 **N**KL -0.77832304 Non-glycosylated

39 **N**GK -1.130584 Non-glycosylated

48 **N**TE -1.2741531 Non-glycosylated

52 **N**SV -0.83972275 Non-glycosylated

**62 NGS 1.1882916 Potential Glycosylated**

68 **N**HA -0.89887659 Non-glycosylated

81 **N**GI -1.2423291 Non-glycosylated

84 **N**PR -1.8662045 Non-glycosylated

112 **N**AG -1.0674394 Non-glycosylated

116 **N**TV -0.71392911 Non-glycosylated

155 **N**LV -1.0444821 Non-glycosylated

200 **N**TV -1.0299987 Non-glycosylated

**225 NNT 0.88963983 Potential Glycosylated**

226 **N**TG -1.0135771 Non-glycosylated

274 **N**LF -1.2838351 Non-glycosylated

329 **N**RL -1.0222076 Non-glycosylated

338 **N**AE -1.1197133 Non-glycosylated

352 **NN**D -1.2714523 Non-glycosylated

353 **N**DP -1.1516042 Non-glycosylated

**374 NGT 1.2707122 Potential Glycosylated**

385 **N**AL -1.0307742 Non-glycosylated

405 **N**AM -1.3410327 Non-glycosylated

***********************************

>Ss15 Length = 413

**Potential N-Linked Glycosylated Sites:**

MHYY**N**KLKFLALASVISRTSAHPTSGHYYADAESEACL**N**GKAVYVTS**N**TEH**N**SVVAIPIAR**N**GSLLL**N**HATSTATGGRGG**N**GI**N**PRGMPAGPDALFSQGS

ITIAGDYLFAV**N**AGS**N**TVTMLAIDEHDPTKVTVVGEPAQLPGEFPTTVGASDKF**N**LVCVGLTGAKAGVSCASYSWYGLGPFDELRTFDLHQTTPPHGPT

**N**TVSHVFFSGDQETVFTTVKGDPAV**NN**TGFLAAYPVEDIHSSCYSIPSVSHKGVISSPDGTAVLFGSTPIPDTT**N**LFVTDASFGAAILGIDDYEEASTL

YKTVIPGQDATCWVAICPATHTAFVTDIRM**N**RLVEMSLA**N**AEIIGEPIDLTTF**NN**DPGLTEIRSGGSFVYALSPG**N**GTTEAWITVL**N**ALTKKPVQHALL

TPLGLDR**N**AMGMAILV

------------------------------------------------

Position Residue Score Prediction

------------------------------------------------

5 **N**KL -0.77832304 Non-glycosylated

39 **N**GK -1.130584 Non-glycosylated

48 **N**TE -1.2741531 Non-glycosylated

52 **N**SV -0.83972275 Non-glycosylated

**62 NGS 1.1882916 Potential Glycosylated**

68 **N**HA -0.89887659 Non-glycosylated

81 **N**GI -1.2423291 Non-glycosylated

84 **N**PR -1.8662045 Non-glycosylated

112 **N**AG -1.0674394 Non-glycosylated

116 **N**TV -0.71392911 Non-glycosylated

155 **N**LV -1.0444821 Non-glycosylated

200 **N**TV -1.0299987 Non-glycosylated

**225 NNT 0.88963983 Potential Glycosylated**

226 **N**TG -1.0135771 Non-glycosylated

274 **N**LF -1.2838351 Non-glycosylated

329 **N**RL -1.0222076 Non-glycosylated

338 **N**AE -1.1197133 Non-glycosylated

352 **NN**D -1.2714523 Non-glycosylated

353 **N**DP -1.1516042 Non-glycosylated

**374 NGT 1.2707122 Potential Glycosylated**

385 **N**AL -1.0307742 Non-glycosylated

405 **N**AM -1.3410327 Non-glycosylated

***********************************

>Ss205 Length = 413

**Potential N-Linked Glycosylated Sites:**

MHYY**N**KLKFLALASVISRTSAHPTSGHYYADAESEACL**N**GKAVYVTS**N**TEH**N**SVVAIPIAR**N**GSLLL**N**HATSTATGGRGG**N**GI**N**PRGMPAGPDALFSQGS

ITIAGDYLFAV**N**AGS**N**TVTMLAIDEHDPTKVTVVGEPAQLPGEFPTTVGASDKF**N**LVCVGLTGAKAGVSCASYSWYGLGPFDELRTFDLHQTTPPHGPT

**N**TVSHVFFSGDQETVFTTVKGDPAV**NN**TGFLAAYPVEDIHSSCYSIPSVSHKGVISSPDGTAVLFGSTPIPDTT**N**LFVTDASFGAAVLGIDDYEEASTL

YKTVIPGQDATCWVAICPATHTAFVTDIRM**N**RLVEMSLA**N**AEIIGEPIDLTTF**NN**DPGLTEIRSGGSFVYALSPG**N**GTTEAWITVL**N**ALTKKPVQHALL

TPLGLDR**N**AMGMAILV

------------------------------------------------

Position Residue Score Prediction

------------------------------------------------

5 **N**KL -0.77832304 Non-glycosylated

39 **N**GK -1.130584 Non-glycosylated

48 **N**TE -1.2741531 Non-glycosylated

52 **N**SV -0.83972275 Non-glycosylated

**62 NGS 1.1882916 Potential Glycosylated**

68 **N**HA -0.89887659 Non-glycosylated

81 **N**GI -1.2423291 Non-glycosylated

84 **N**PR -1.8662045 Non-glycosylated

112 **N**AG -1.0674394 Non-glycosylated

116 **N**TV -0.71392911 Non-glycosylated

155 **N**LV -1.0444821 Non-glycosylated

200 **N**TV -1.0299987 Non-glycosylated

**225 NNT 0.88963983 Potential Glycosylated**

226 **N**TG -1.0135771 Non-glycosylated

274 **N**LF -1.2838351 Non-glycosylated

329 **N**RL -1.0222076 Non-glycosylated

338 **N**AE -1.1197133 Non-glycosylated

352 **NN**D -1.2714523 Non-glycosylated

353 **N**DP -1.1516042 Non-glycosylated

**374 NGT 1.2707122 Potential Glycosylated**

385 **N**AL -1.0307742 Non-glycosylated

405 **N**AM -1.3410327 Non-glycosylated

***********************************

>Ss22 Length = 413

**Potential N-Linked Glycosylated Sites:**

MHYY**N**KLKFLALASVISRTSAHPTSGHYYADAESEACL**N**GKAVYVTS**N**TEH**N**SVVAIPIAR**N**GSLLL**N**HATSTATGGRGG**N**GI**N**PRGMPAGPDALFSQGS

ITIAGDYLFAV**N**AGS**N**TVTMLAIDEHDPTKVTVVGEPAQLPGEFPTTVGASDKF**N**LVCVGLTGAKAGVSCASYSWYGLGPFDELRTFDLHQTTPPHGPT

**N**TVSHVFFSGDQETVFTTVKGDPAV**NN**TGFLAAYPVEDIHSSCYSIPSVSHKGVISSPDGTAVLFGSTPIPDTT**N**LFVTDASFGAAILGIDDYEEASTL

YKTVIPGQDATCWVAICPATHTAFVTDIRM**N**RLVEMSLA**N**AEIIGEPIDLTTF**NN**DPGLTEIRSGGSFVYALSPG**N**GTTEAWITVL**N**ALTKKPVQHALL

TPLGLDR**N**AMGMAILV

------------------------------------------------

Position Residue Score Prediction

------------------------------------------------

5 **N**KL -0.77832304 Non-glycosylated

39 **N**GK -1.130584 Non-glycosylated

48 **N**TE -1.2741531 Non-glycosylated

52 **N**SV -0.83972275 Non-glycosylated

**62 NGS 1.1882916 Potential Glycosylated**

68 **N**HA -0.89887659 Non-glycosylated

81 **N**GI -1.2423291 Non-glycosylated

84 **N**PR -1.8662045 Non-glycosylated

112 **N**AG -1.0674394 Non-glycosylated

116 **N**TV -0.71392911 Non-glycosylated

155 **N**LV -1.0444821 Non-glycosylated

200 **N**TV -1.0299987 Non-glycosylated

**225 NNT 0.88963983 Potential Glycosylated**

226 **N**TG -1.0135771 Non-glycosylated

274 **N**LF -1.2838351 Non-glycosylated

329 **N**RL -1.0222076 Non-glycosylated

338 **N**AE -1.1197133 Non-glycosylated

352 **NN**D -1.2714523 Non-glycosylated

353 **N**DP -1.1516042 Non-glycosylated

**374 NGT 1.2707122 Potential Glycosylated**

385 **N**AL -1.0307742 Non-glycosylated

405 **N**AM -1.3410327 Non-glycosylated

***********************************

>Ss61 Length = 413

**Potential N-Linked Glycosylated Sites:**

MHYY**N**KLKFLALASVISRTSAHPTGGHYYADAESEACL**N**GKAVYVTS**N**TEH**N**SVVAIPIAR**N**GSLLL**N**HATSTATGGRGG**N**GI**N**PRGMPAGPDALFSQGS

ITIAGDYLFAV**N**AGS**N**TVTMLAIDEHDPTKVTVVGEPAQLPGEFPTTVGASDKF**N**LVCVGLTGAKAGVSCASYSWYGLGPFDELRTFDLHQTTPPHGPT

**N**TVSHVFFSGDQETVFTTVKGDPAV**NN**TGFLAAYPVEDIHSSCYSIPSVSHKGVISSPDGTAVLFGSTPIPDTT**N**LFVTDASFGAAILGIDDYEEASTL

YKTVIPGQDATCWVAICPATHTAFVTDIRM**N**RLVEMSLA**N**AEIIGEPIDLTTF**NN**DPGLTEIRSGGSFVYALSPG**N**GTTEAWITVL**N**ALTKKPVQHALL

TPLGLDR**N**AMGMAILV

------------------------------------------------

Position Residue Score Prediction

------------------------------------------------

5 **N**KL -0.77832304 Non-glycosylated

39 **N**GK -1.130584 Non-glycosylated

48 **N**TE -1.2741531 Non-glycosylated

52 **N**SV -0.83972275 Non-glycosylated

**62 NGS 1.1882916 Potential Glycosylated**

68 **N**HA -0.89887659 Non-glycosylated

81 **N**GI -1.2423291 Non-glycosylated

84 **N**PR -1.8662045 Non-glycosylated

112 **N**AG -1.0674394 Non-glycosylated

116 **N**TV -0.71392911 Non-glycosylated

155 **N**LV -1.0444821 Non-glycosylated

200 **N**TV -1.0299987 Non-glycosylated

**225 NNT 0.88963983 Potential Glycosylated**

226 **N**TG -1.0135771 Non-glycosylated

274 **N**LF -1.2838351 Non-glycosylated

329 **N**RL -1.0222076 Non-glycosylated

338 **N**AE -1.1197133 Non-glycosylated

352 **NN**D -1.2714523 Non-glycosylated

353 **N**DP -1.1516042 Non-glycosylated

**374 NGT 1.2707122 Potential Glycosylated**

385 **N**AL -1.0307742 Non-glycosylated

405 **N**AM -1.3410327 Non-glycosylated

***********************************

>Ss468 Length = 413

**Potential N-Linked Glycosylated Sites:**

MHYY**N**KLKFLALTSVISVTSAHPTSDHYYADAESEACL**N**GKAVYVTS**N**TEH**N**SVVAIPIAR**N**GSLLTDHATSTATGGRGG**N**GI**N**PRGMPAGPDALFGQGS

ITIAGDYLFAV**N**AGS**N**TVTMLAIDKHDPTKVTVVGEPAELPGEFPTTVGASDKF**N**LVCVGLTGAKAGVSCASYSWYGLGPFDELRPFDLHQTTPPHGPT

**N**TVSHVFFS**N**DQETVFTTVKGDPAV**NN**TGFLAAYPVEHIHSSCYATPSVSHKGVISSPDGTAVLFGSIPIPETT**N**LFATDASFGAVILGIDDYEEASTL

YKTVIPGQDATCWVAICPATHTAFVTDIRM**N**RLVEMSLV**N**AEIIGEPIDLTAF**NN**DPGLTEIRSGGSFVYALSPG**N**GTTEAWITVL**N**ALTKKPVQHALL

TPLGLDR**N**AMGMAILV

------------------------------------------------

Position Residue Score Prediction

------------------------------------------------

5 **N**KL -0.7253544 Non-glycosylated

39 **N**GK -1.130584 Non-glycosylated

48 **N**TE -1.2741531 Non-glycosylated

52 **N**SV -0.83972275 Non-glycosylated

**62 NGS 1.245631 Potential Glycosylated**

81 **N**GI -1.2423291 Non-glycosylated

84 **N**PR -1.8662045 Non-glycosylated

112 **N**AG -1.0674394 Non-glycosylated

116 **N**TV -0.66346953 Non-glycosylated

155 **N**LV -1.0444821 Non-glycosylated

200 **N**TV -1.0355588 Non-glycosylated

209 **N**DQ -0.69609684 Non-glycosylated

**225 NNT 0.88963983 Potential Glycosylated**

226 **N**TG -1.0135771 Non-glycosylated

274 **N**LF -1.3818825 Non-glycosylated

329 **N**RL -1.0665658 Non-glycosylated

338 **N**AE -1.1315083 Non-glycosylated

352 **NN**D -1.1048736 Non-glycosylated

353 **N**DP -1.096786 Non-glycosylated

**374 NGT 1.2707122 Potential Glycosylated**

385 **N**AL -1.0307742 Non-glycosylated

405 **N**AM -1.3410327 Non-glycosylated

***********************************

>Ss51 Length = 413

**Potential N-Linked Glycosylated Sites:**

MHYY**N**KLKFLALTSVISVTSAHPTSDHYYADAESEACL**N**GKAVYVTS**N**TEH**N**SVVAIPIAR**N**GSLLTDHATSTATGGRGG**N**GI**N**PRGMPAGPDALFGQGS

ITIAGDYLFAV**N**AGS**N**TVTMLAIDKHDPTKVTVVGEPAELPGEFPTTVGASDKF**N**LVCVGLTGAKAGVSCASYSWYGLGPFDELRPFDLHQTTPPHGPT

**N**TVSHVFFSDDQETVFTTVKGDPAV**NN**TGFLAAYPVEHIHSSCYATPSVSHKGVISSPDGTAVLFGSIPIPETT**N**LFATDASFGAVILGIDDYEEASTL

YKTVIPGQDATCWVAICPATHTAFVTDIRM**N**RLVEMSLV**N**AEIIGEPIDLTAF**NN**DPGLTEIRSGGSFVYALSPG**N**GTTEAWITVL**N**ALTKKPVQHALL

TPLGLDR**N**AMGMAILV

------------------------------------------------

Position Residue Score Prediction

------------------------------------------------

5 **N**KL -0.7253544 Non-glycosylated

39 **N**GK -1.130584 Non-glycosylated

48 **N**TE -1.2741531 Non-glycosylated

52 **N**SV -0.83972275 Non-glycosylated

**62 NGS 1.245631 Potential Glycosylated**

81 **N**GI -1.2423291 Non-glycosylated

84 **N**PR -1.8662045 Non-glycosylated

112 **N**AG -1.0674394 Non-glycosylated

116 **N**TV -0.66346953 Non-glycosylated

155 **N**LV -1.0444821 Non-glycosylated

200 **N**TV -1.0214888 Non-glycosylated

**225 NNT 0.88963983 Potential Glycosylated**

226 **N**TG -1.0135771 Non-glycosylated

274 **N**LF -1.3818825 Non-glycosylated

329 **N**RL -1.0665658 Non-glycosylated

338 **N**AE -1.1315083 Non-glycosylated

352 **NN**D -1.1048736 Non-glycosylated

353 **N**DP -1.096786 Non-glycosylated

**374 NGT 1.2707122 Potential Glycosylated**

385 **N**AL -1.0307742 Non-glycosylated

405 **N**AM -1.3410327 Non-glycosylated

***********************************

>Ss467 Length = 413

**Potential N-Linked Glycosylated Sites:**

MHYY**N**KLKLLALASVISVTSAHPT**N**DRYYADADSEACL**N**GKAVYVTS**N**TAH**N**SVVALPIAR**N**GSLLV**N**HATSTATGGSGG**N**GI**N**PRGMPAGPDALFGQGS

ITVAGDYLFSV**N**AGS**N**TVTMLAIDKHDPTKLTVVGEPAVLPGEFP**N**TVGASDKF**N**LVCVGLTGAKAGVSCASYSWYGLGPFDELRPFDL**N**QTTPPHGPT

**N**TVSHVFFSGDQETVFATVKGDPTV**NN**TGFLAAYPVE**N**IHTSCYATPSVSYKGVMSSP**N**GTAVLFGSTPIPDTT**N**LFVTDASFGATILGVDDYGKASTL

YKTVIPGQDATCWVAICPATHTAFVTDIRV**N**RLVEMSLV**N**AEIIGEPIDLTTF**NN**DPGLTEIRSSGSFVYALSPG**N**GTTEAWITVL**N**ALTK**N**PVQLALL

TPLGLDR**N**AMGMAILV

------------------------------------------------

Position Residue Score Prediction

------------------------------------------------

5 **N**KL -0.68032779 Non-glycosylated

25 **N**DR -0.90706694 Non-glycosylated

39 **N**GK -1.2018316 Non-glycosylated

48 **N**TA -1.28992 Non-glycosylated

52 **N**SV -0.63933703 Non-glycosylated

**62 NGS 1.1913176 Potential Glycosylated**

68 **N**HA -0.93420911 Non-glycosylated

81 **N**GI -1.2485877 Non-glycosylated

84 **N**PR -1.8602168 Non-glycosylated

112 **N**AG -1.1497502 Non-glycosylated

116 **N**TV -0.70159048 Non-glycosylated

146 **N**TV -1.1767199 Non-glycosylated

155 **N**LV -0.99315998 Non-glycosylated

**190 NQT 0.98748688 Potential Glycosylated**

200 **N**TV -0.97636502 Non-glycosylated

**225 NNT 0.83519615 Potential Glycosylated**

226 **N**TG -1.089221 Non-glycosylated

237 **N**IH -1.0717965 Non-glycosylated

**258 NGT 1.0356973 Potential Glycosylated**

274 **N**LF -1.2838351 Non-glycosylated

329 **N**RL -1.1062136 Non-glycosylated

338 **N**AE -1.0761808 Non-glycosylated

352 **NN**D -1.2714523 Non-glycosylated

353 **N**DP -1.0824142 Non-glycosylated

**374 NGT 1.2707122 Potential Glycosylated**

385 **N**AL -1.0222289 Non-glycosylated

390 **N**PV -1.0342313 Non-glycosylated

405 **N**AM -1.3410327 Non-glycosylated

***********************************

>Ss449 Length = 413

**Potential N-Linked Glycosylated Sites:**

MHYY**N**KLKLLALASVISVTSAHPT**N**DRYYADADSEACL**N**GKAVYVTS**N**TAH**N**SVVALPIAR**N**GSLLV**N**HATSTATGGSGG**N**GI**N**PRGMPAGPDALFGQGS

ITVAGDYLFSV**N**AGS**N**TVTMLAIDKHDPTKLTVVGEPAVLPGEFP**N**TVGASDKF**N**LVCVGLTGAKAGVSCASYSWYGLGPFDELRPFDL**N**QTTPPHGPT

**N**TVSHVFFSGDQETVFATVKGDPTV**NN**TGFLAAYPVE**N**IHTSCYATPSVSYKGVMSSP**N**GTAVLFGSTPIPDTT**N**LFVTDASFGATILGVDDYGKASTL

YKTVIPGQDATCWVAICPATHTAFVTDIRV**N**RLVEMSLV**N**AEIIGEPIDLTTF**NN**DPGLTEIRSSGSFVYALSPG**N**GTTEAWITVL**N**ALTKKPVQHALL

TPLGLDR**N**AMGMAILV

------------------------------------------------

Position Residue Score Prediction

------------------------------------------------

5 **N**KL -0.68032779 Non-glycosylated

25 **N**DR -0.90706694 Non-glycosylated

39 **N**GK -1.2018316 Non-glycosylated

48 **N**TA -1.28992 Non-glycosylated

52 **N**SV -0.63933703 Non-glycosylated

**62 NGS 1.1913176 Potential Glycosylated**

68 **N**HA -0.93420911 Non-glycosylated

81 **N**GI -1.2485877 Non-glycosylated

84 **N**PR -1.8602168 Non-glycosylated

112 **N**AG -1.1497502 Non-glycosylated

116 **N**TV -0.70159048 Non-glycosylated

146 **N**TV -1.1767199 Non-glycosylated

155 **N**LV -0.99315998 Non-glycosylated

**190 NQT 0.98748688 Potential Glycosylated**

200 **N**TV -0.97636502 Non-glycosylated

**225 NNT 0.83519615 Potential Glycosylated**

226 **N**TG -1.089221 Non-glycosylated

237 **N**IH -1.0717965 Non-glycosylated

**258 NGT 1.0356973 Potential Glycosylated**

274 **N**LF -1.2838351 Non-glycosylated

329 **N**RL -1.1062136 Non-glycosylated

338 **N**AE -1.0761808 Non-glycosylated

352 **NN**D -1.2714523 Non-glycosylated

353 **N**DP -1.0824142 Non-glycosylated

**374 NGT 1.2707122 Potential Glycosylated**

385 **N**AL -1.0307742 Non-glycosylated

405 **N**AM -1.3410327 Non-glycosylated

***********************************

>Ss445 Length = 413

**Potential N-Linked Glycosylated Sites:**

MHYY**N**KLKLLALASVISVTSAHPT**N**DRYYADADSEACL**N**GKAVYVTS**N**TAH**N**SVVALPIAR**N**GSLLV**N**HATSTATGGSGG**N**GI**N**PRGMPAGPDALFGQGS

ITVAGDYLFSV**N**AGS**N**TVTMLAIDKHDPTKLTVVGEPAVLPGEFP**N**TVGASDKF**N**LVCVGLTGAKAGVSCASYSWYGLGPFDELRPFDL**N**QTTPPHGPT

**N**TVSHVFFSGDQETVFATVKGDPTV**NN**TGFLAAYPVE**N**IHTSCYATPSVSYKGVMSSP**N**GTAVLFGSTPIPDTT**N**LFVTDASFGATILGVDDYGKASTL

YKTVIPGQDATCWVAICPATHTAFVTDIRV**N**RLVEMSLV**N**AEIIGEPIDLTTF**NN**DPGLTEIRSSGSFVYALSPG**N**GTTEAWITVL**N**ALTKKPVQHALL

TPLGLDR**N**AMGMAILV

------------------------------------------------

Position Residue Score Prediction

------------------------------------------------

5 **N**KL -0.68032779 Non-glycosylated

25 **N**DR -0.90706694 Non-glycosylated

39 **N**GK -1.2018316 Non-glycosylated

48 **N**TA -1.28992 Non-glycosylated

52 **N**SV -0.63933703 Non-glycosylated

**62 NGS 1.1913176 Potential Glycosylated**

68 **N**HA -0.93420911 Non-glycosylated

81 **N**GI -1.2485877 Non-glycosylated

84 **N**PR -1.8602168 Non-glycosylated

112 **N**AG -1.1497502 Non-glycosylated

116 **N**TV -0.70159048 Non-glycosylated

146 **N**TV -1.1767199 Non-glycosylated

155 **N**LV -0.99315998 Non-glycosylated

**190 NQT 0.98748688 Potential Glycosylated**

200 **N**TV -0.97636502 Non-glycosylated

**225 NNT 0.83519615 Potential Glycosylated**

226 **N**TG -1.089221 Non-glycosylated

237 **N**IH -1.0717965 Non-glycosylated

**258 NGT 1.0356973 Potential Glycosylated**

274 **N**LF -1.2838351 Non-glycosylated

329 **N**RL -1.1062136 Non-glycosylated

338 **N**AE -1.0761808 Non-glycosylated

352 **NN**D -1.2714523 Non-glycosylated

353 **N**DP -1.0824142 Non-glycosylated

**374 NGT 1.2707122 Potential Glycosylated**

385 **N**AL -1.0307742 Non-glycosylated

405 **N**AM -1.3410327 Non-glycosylated

***********************************

>Ss456 Length = 413

**Potential N-Linked Glycosylated Sites:**

MHYY**N**KLKLLALASVISVTSAHPT**N**DRYYADADSEACL**N**GKAVYVTS**N**TAH**N**SVVALPIAR**N**GSLLV**N**HATSTATGGSGG**N**GI**N**PRGMPAGPDALFGQGS

ITVAGDYLFSV**N**AGS**N**TVTMLAIDKHDPTKLTVVGEPAVLPGEFP**N**TVGASDKF**N**LVCVGLTGAKAGVSCASYSWYGLGPFDELRPFDL**N**QTTPPHGPT

**N**TVSHVFFSGDQETVFATVKGDPTV**NN**TGFLAAYPVE**N**IHTSCYATPSVSYKGVMSSP**N**GTAVLFGSTPIPDTT**N**LFVTDASFGATILGVDDYGKASTL

YKTVIPGQDATCWVAICPATHTAFVTDIRV**N**RLVEMSLV**N**AEIIGEPIDLTTF**NN**DPGLTEIRSSGSFVYALSPG**N**GTTEAWITVL**N**ALTKKPVQHALL

TPLGLDR**N**AMGMAILV

------------------------------------------------

Position Residue Score Prediction

------------------------------------------------

5 **N**KL -0.68032779 Non-glycosylated

25 **N**DR -0.90706694 Non-glycosylated

39 **N**GK -1.2018316 Non-glycosylated

48 **N**TA -1.28992 Non-glycosylated

52 **N**SV -0.63933703 Non-glycosylated

**62 NGS 1.1913176 Potential Glycosylated**

68 **N**HA -0.93420911 Non-glycosylated

81 **N**GI -1.2485877 Non-glycosylated

84 **N**PR -1.8602168 Non-glycosylated

112 **N**AG -1.1497502 Non-glycosylated

116 **N**TV -0.70159048 Non-glycosylated

146 **N**TV -1.1767199 Non-glycosylated

155 **N**LV -0.99315998 Non-glycosylated

**190 NQT 0.98748688 Potential Glycosylated**

200 **N**TV -0.97636502 Non-glycosylated

**225 NNT 0.83519615 Potential Glycosylated**

226 **N**TG -1.089221 Non-glycosylated

237 **N**IH -1.0717965 Non-glycosylated

**258 NGT 1.0356973 Potential Glycosylated**

274 **N**LF -1.2838351 Non-glycosylated

329 **N**RL -1.1062136 Non-glycosylated

338 **N**AE -1.0761808 Non-glycosylated

352 **NN**D -1.2714523 Non-glycosylated

353 **N**DP -1.0824142 Non-glycosylated

**374 NGT 1.2707122 Potential Glycosylated**

385 **N**AL -1.0307742 Non-glycosylated

405 **N**AM -1.3410327 Non-glycosylated

***********************************

>Ss457 Length = 413

**Potential N-Linked Glycosylated Sites:**

MHYY**N**KLKLLALASVISVTSAHPT**N**DRYYADADSEACL**N**GKAVYVTS**N**TAH**N**SVVALPIAR**N**GSLLV**N**HATSTATGGSGG**N**GI**N**PRGMPAGPDALFGQGS

ITVAGDYLFSV**N**AGS**N**TVTMLAIDKHDPTKLTVVGEPAVLPGEFP**N**TVGASDKF**N**LVCVGLTGAKAGVSCASYSWYGLGPFDELRPFDL**N**QTTPPHGPT

**N**TVSHVFFSGDQETVFATVKGDPTV**NN**TGFLAAYPVE**N**IHTSCYATPSVSYKGVMSSP**N**GTAVLFGSTPIPDTT**N**LFVTDASFGATILGVDDYGKASTL

YKTVIPGQDATCWVAICPATHTAFVTDIRV**N**RLVEMSLV**N**AEIIGEPIDLTTF**NN**DPGLTEIRSSGSFVYALSPG**N**GTTEAWITVL**N**ALTKKPVQHALL

TPLGLDR**N**AMGMAILV

------------------------------------------------

Position Residue Score Prediction

------------------------------------------------

5 **N**KL -0.68032779 Non-glycosylated

25 **N**DR -0.90706694 Non-glycosylated

39 **N**GK -1.2018316 Non-glycosylated

48 **N**TA -1.28992 Non-glycosylated

52 **N**SV -0.63933703 Non-glycosylated

**62 NGS 1.1913176 Potential Glycosylated**

68 **N**HA -0.93420911 Non-glycosylated

81 **N**GI -1.2485877 Non-glycosylated

84 **N**PR -1.8602168 Non-glycosylated

112 **N**AG -1.1497502 Non-glycosylated

116 **N**TV -0.70159048 Non-glycosylated

146 **N**TV -1.1767199 Non-glycosylated

155 **N**LV -0.99315998 Non-glycosylated

**190 NQT 0.98748688 Potential Glycosylated**

200 **N**TV -0.97636502 Non-glycosylated

**225 NNT 0.83519615 Potential Glycosylated**

226 **N**TG -1.089221 Non-glycosylated

237 **N**IH -1.0717965 Non-glycosylated

**258 NGT 1.0356973 Potential Glycosylated**

274 **N**LF -1.2838351 Non-glycosylated

329 **N**RL -1.1062136 Non-glycosylated

338 **N**AE -1.0761808 Non-glycosylated

352 **NN**D -1.2714523 Non-glycosylated

353 **N**DP -1.0824142 Non-glycosylated

**374 NGT 1.2707122 Potential Glycosylated**

385 **N**AL -1.0307742 Non-glycosylated

405 **N**AM -1.3410327 Non-glycosylated

***********************************

>Ss443 Length = 413

**Potential N-Linked Glycosylated Sites:**

MHYY**N**KLKLLALASVISVTSAHPT**N**DRYYADADSEACL**N**GKAVYVTS**N**TAH**N**SVVALPIAR**N**GSLLV**N**HATSTATGGSGG**N**GI**N**PRGMPAGPDALFGQGS

ITVAGDYLFSV**N**AGS**N**TVTMLAIDKHDPTKLTVVGEPAVLPGEFP**N**TVGASDKF**N**LVCVGLTGAKAGVSCASYSWYGLGPFDELRPFDL**N**QTTPPHGPT

**N**TVSHVFFSGDQETVFATVKGDPTV**NN**TGFLAAYPVE**N**IHTSCYATPSVSYKGVMSSP**N**GTAVLFGSTPIPDTT**N**LFVTDASFGATILGVDDYGKASTL

YKTVIPGQDATCWVAICPATHTAFVTDIRV**N**RLVEMSLV**N**AEIIGEPIDLTTF**NN**DPGLTEIRSSGSFVYALSPG**N**GTTEAWITVL**N**ALTKKPVQHALL

TPLGLDR**N**AMGMAILV

------------------------------------------------

Position Residue Score Prediction

------------------------------------------------

5 **N**KL -0.68032779 Non-glycosylated

25 **N**DR -0.90706694 Non-glycosylated

39 **N**GK -1.2018316 Non-glycosylated

48 **N**TA -1.28992 Non-glycosylated

52 **N**SV -0.63933703 Non-glycosylated

**62 NGS 1.1913176 Potential Glycosylated**

68 **N**HA -0.93420911 Non-glycosylated

81 **N**GI -1.2485877 Non-glycosylated

84 **N**PR -1.8602168 Non-glycosylated

112 **N**AG -1.1497502 Non-glycosylated

116 **N**TV -0.70159048 Non-glycosylated

146 **N**TV -1.1767199 Non-glycosylated

155 **N**LV -0.99315998 Non-glycosylated

**190 NQT 0.98748688 Potential Glycosylated**

200 **N**TV -0.97636502 Non-glycosylated

**225 NNT 0.83519615 Potential Glycosylated**

226 **N**TG -1.089221 Non-glycosylated

237 **N**IH -1.0717965 Non-glycosylated

**258 NGT 1.0356973 Potential Glycosylated**

274 **N**LF -1.2838351 Non-glycosylated

329 **N**RL -1.1062136 Non-glycosylated

338 **N**AE -1.0761808 Non-glycosylated

352 **NN**D -1.2714523 Non-glycosylated

353 **N**DP -1.0824142 Non-glycosylated

**374 NGT 1.2707122 Potential Glycosylated**

385 **N**AL -1.0307742 Non-glycosylated

405 **N**AM -1.3410327 Non-glycosylated

***********************************

>Ss06 Length = 413

**Potential N-Linked Glycosylated Sites:**

MHYY**N**KLKLLALASVISVTSAHPT**N**DRYYADADSEACL**N**GKAVYVTS**N**TAH**N**SVVALPIAR**N**GSLLV**N**HATSTATGGSGG**N**GI**N**PRGMPAGPDALFGQGS

ITVAGDYLFSV**N**AGS**N**TVTMLAIDKHDPTKLTVVGEPAVLPGEFP**N**TVGASDKF**N**LVCVGLTGAKAGVSCASYSWYGLGPFDELRPFDL**N**QTTPPHGPT

**N**TVSHVFFSGDQETVFATVKGDPTV**NN**TGFLAAYPVE**N**IHTSCYATPSVSYKGVMSSP**N**GTAVLFGSTPIPDTT**N**LFVTDASFGATILGVDDYGKASTL

YKTVIPGQDATCWVAICPATHTAFVTDIRV**N**RLVEMSLV**N**AEIIGEPIDLTTF**NN**DPGLTEIRSSGSFVYALSPG**N**GTTEAWITVL**N**ALTKKPVQHALL

TPLGLDR**N**AMGMAILV

------------------------------------------------

Position Residue Score Prediction

------------------------------------------------

5 **N**KL -0.68032779 Non-glycosylated

25 **N**DR -0.90706694 Non-glycosylated

39 **N**GK -1.2018316 Non-glycosylated

48 **N**TA -1.28992 Non-glycosylated

52 **N**SV -0.63933703 Non-glycosylated

**62 NGS 1.1913176 Potential Glycosylated**

68 **N**HA -0.93420911 Non-glycosylated

81 **N**GI -1.2485877 Non-glycosylated

84 **N**PR -1.8602168 Non-glycosylated

112 **N**AG -1.1497502 Non-glycosylated

116 **N**TV -0.70159048 Non-glycosylated

146 **N**TV -1.1767199 Non-glycosylated

155 **N**LV -0.99315998 Non-glycosylated

**190 NQT 0.98748688 Potential Glycosylated**

200 **N**TV -0.97636502 Non-glycosylated

**225 NNT 0.83519615 Potential Glycosylated**

226 **N**TG -1.089221 Non-glycosylated

237 **N**IH -1.0717965 Non-glycosylated

**258 NGT 1.0356973 Potential Glycosylated**

274 **N**LF -1.2838351 Non-glycosylated

329 **N**RL -1.1062136 Non-glycosylated

338 **N**AE -1.0761808 Non-glycosylated

352 **NN**D -1.2714523 Non-glycosylated

353 **N**DP -1.0824142 Non-glycosylated

**374 NGT 1.2707122 Potential Glycosylated**

385 **N**AL -1.0307742 Non-glycosylated

405 **N**AM -1.3410327 Non-glycosylated

***********************************

>Ss446 Length = 413

**Potential N-Linked Glycosylated Sites:**

MHYY**N**KLKLLALASVISVTSAHPT**N**DRYYADADSEACL**N**GKAVYVTS**N**TAH**N**SVVALPIAR**N**GSLLV**N**HATSTATGGSGG**N**GI**N**PRGMPAGPDALFGQGS

ITVAGDYLFSV**N**AGS**N**TVTMLAIDKHDPTKLTVVGEPAVLPGEFP**N**TVGASDKF**N**LVCVGLTGAKAGVSCASYSWYGLGPFDELRPFDL**N**QTTPPHGPT

**N**TVSHVFFSGDQETVFATVKGDPTV**NN**TGFLAAYPVE**N**IHTSCYATPSVSYKGVMSSP**N**GTAVLFGSTPIPDTT**N**LFVTDASFGATILGVDDYGKASTL

YKTVIPGQDATCWVAICPATHTAFVTDIRV**N**RLVEMSLV**N**AEIIGEPIDLTTF**NN**DPGLTEIRSSGSFVYALSPG**N**GTTEAWITVL**N**ALTKKPVQHALL

TPLGLDR**N**AMGMAILV

------------------------------------------------

Position Residue Score Prediction

------------------------------------------------

5 **N**KL -0.68032779 Non-glycosylated

25 **N**DR -0.90706694 Non-glycosylated

39 **N**GK -1.2018316 Non-glycosylated

48 **N**TA -1.28992 Non-glycosylated

52 **N**SV -0.63933703 Non-glycosylated

**62 NGS 1.1913176 Potential Glycosylated**

68 **N**HA -0.93420911 Non-glycosylated

81 **N**GI -1.2485877 Non-glycosylated

84 **N**PR -1.8602168 Non-glycosylated

112 **N**AG -1.1497502 Non-glycosylated

116 **N**TV -0.70159048 Non-glycosylated

146 **N**TV -1.1767199 Non-glycosylated

155 **N**LV -0.99315998 Non-glycosylated

**190 NQT 0.98748688 Potential Glycosylated**

200 **N**TV -0.97636502 Non-glycosylated

**225 NNT 0.83519615 Potential Glycosylated**

226 **N**TG -1.089221 Non-glycosylated

237 **N**IH -1.0717965 Non-glycosylated

**258 NGT 1.0356973 Potential Glycosylated**

274 **N**LF -1.2838351 Non-glycosylated

329 **N**RL -1.1062136 Non-glycosylated

338 **N**AE -1.0761808 Non-glycosylated

352 **NN**D -1.2714523 Non-glycosylated

353 **N**DP -1.0824142 Non-glycosylated

**374 NGT 1.2707122 Potential Glycosylated**

385 **N**AL -1.0307742 Non-glycosylated

405 **N**AM -1.3410327 Non-glycosylated

***********************************

>FMR8595 Length = 413

**Potential N-Linked Glycosylated Sites:**

MHYY**N**KLKLLALASVISVTSAHPT**N**DRYYADADSEACL**N**GKAVYVTS**N**TAH**N**SVVALPIAR**N**GSLLV**N**HATSTATGGSGG**N**GI**N**PRGMPAGPDALFGQGS

ITVAGDYLFSV**N**AGS**N**TVTMLAIDKHDPTKLTVVGEPAVLPGEFP**N**TVGASDKF**N**LVCVGLTGAKAGVSCASYSWYGLGPFDELRPFDL**N**QTTPPHGPT

**N**TVSHVFFSGDQETVFATVKGDPTV**NN**TGFLAAYPVE**N**IHTSCYATPSVSYKGVMSSP**N**GTAVLFGSTPIPDTT**N**LFVTDASFGATILGVDDYGKASTL

YKTVIPGQDATCWVAICPATHTAFVTDIRV**N**RLVEMSLV**N**AEIIGEPIDLTTF**NN**DPGLTEIRSSGSFVYALSPG**N**GTTEAWITVL**N**ALTKKPVQHALL

TPLGLDR**N**AMGMAILV

------------------------------------------------

Position Residue Score Prediction

------------------------------------------------

5 **N**KL -0.68032779 Non-glycosylated

25 **N**DR -0.90706694 Non-glycosylated

39 **N**GK -1.2018316 Non-glycosylated

48 **N**TA -1.28992 Non-glycosylated

52 **N**SV -0.63933703 Non-glycosylated

**62 NGS 1.1913176 Potential Glycosylated**

68 **N**HA -0.93420911 Non-glycosylated

81 **N**GI -1.2485877 Non-glycosylated

84 **N**PR -1.8602168 Non-glycosylated

112 **N**AG -1.1497502 Non-glycosylated

116 **N**TV -0.70159048 Non-glycosylated

146 **N**TV -1.1767199 Non-glycosylated

155 **N**LV -0.99315998 Non-glycosylated

**190 NQT 0.98748688 Potential Glycosylated**

200 **N**TV -0.97636502 Non-glycosylated

**225 NNT 0.83519615 Potential Glycosylated**

226 **N**TG -1.089221 Non-glycosylated

237 **N**IH -1.0717965 Non-glycosylated

**258 NGT 1.0356973 Potential Glycosylated**

274 **N**LF -1.2838351 Non-glycosylated

329 **N**RL -1.1062136 Non-glycosylated

338 **N**AE -1.0761808 Non-glycosylated

352 **NN**D -1.2714523 Non-glycosylated

353 **N**DP -1.0824142 Non-glycosylated

**374 NGT 1.2707122 Potential Glycosylated**

385 **N**AL -1.0307742 Non-glycosylated

405 **N**AM -1.3410327 Non-glycosylated

***********************************

>CBS120340 Length = 413

**Potential N-Linked Glycosylated Sites:**

MHYY**N**KLKLLALASVISVTSAHPT**N**DRYYADADSEACL**N**GKAVYVTS**N**TAH**N**SVVALPIAR**N**GSLLV**N**HATSTATGGSGG**N**GI**N**PRGMPAGPDALFGQGS

ITVAGDYLFSV**N**AGS**N**TVTMLAIDKHDPTKLTVVGEPAVLPGEFP**N**TVGASDKF**N**LVCVGLTGAKAGVSCASYSWYGLGPFDELRPFDL**N**QTTPPHGPT

**N**TVSHVFFSGDQETVFATVKGDPTV**NN**TGFLAAYPVE**N**IHTSCYATPSVSYKGVMSSP**N**GTAVLFGSTPIPDTT**N**LFVTDASFGATILGVDDYGKASTL

YKTVIPGQDATCWVAICPATHTAFVTDIRV**N**RLVEMSLV**N**AEIIGEPIDLTTF**NN**DPGLTEIRSSGSFVYALSPG**N**GTTEAWITVL**N**ALTKKPVQHALL

TPLGLDR**N**AMGMAILV

------------------------------------------------

Position Residue Score Prediction

------------------------------------------------

5 **N**KL -0.68032779 Non-glycosylated

25 **N**DR -0.90706694 Non-glycosylated

39 **N**GK -1.2018316 Non-glycosylated

48 **N**TA -1.28992 Non-glycosylated

52 **N**SV -0.63933703 Non-glycosylated

**62 NGS 1.1913176 Potential Glycosylated**

68 **N**HA -0.93420911 Non-glycosylated

81 **N**GI -1.2485877 Non-glycosylated

84 **N**PR -1.8602168 Non-glycosylated

112 **N**AG -1.1497502 Non-glycosylated

116 **N**TV -0.70159048 Non-glycosylated

146 **N**TV -1.1767199 Non-glycosylated

155 **N**LV -0.99315998 Non-glycosylated

**190 NQT 0.98748688 Potential Glycosylated**

200 **N**TV -0.97636502 Non-glycosylated

**225 NNT 0.83519615 Potential Glycosylated**

226 **N**TG -1.089221 Non-glycosylated

237 **N**IH -1.0717965 Non-glycosylated

**258 NGT 1.0356973 Potential Glycosylated**

274 **N**LF -1.2838351 Non-glycosylated

329 **N**RL -1.1062136 Non-glycosylated

338 **N**AE -1.0761808 Non-glycosylated

352 **NN**D -1.2714523 Non-glycosylated

353 **N**DP -1.0824142 Non-glycosylated

**374 NGT 1.2707122 Potential Glycosylated**

385 **N**AL -1.0307742 Non-glycosylated

405 **N**AM -1.3410327 Non-glycosylated

***********************************

>Ss471 Length = 413

**Potential N-Linked Glycosylated Sites:**

MHYY**N**KLKLLALASVISVTSAHPT**N**DRYYADADSEACL**N**GKAVYVTS**N**TAH**N**SVVALPIAR**N**GSLLV**N**HATSTATGGSGG**N**GI**N**PRGMPAGPDALFGQGS

ITVAGDYLFSV**N**AGS**N**TVTMLAIDKHDPTKLTVVGEPAVLPGEFP**N**TVGASDKF**N**LVCVGLTGAKAGVSCASYSWYGLGPFDELRPFDL**N**QTTPPHGPT

**N**TVSHVFFSGDQETVFATVKGDPTV**NN**TGFLAAYPVE**N**IHTSCYATPSVSYKGVMSSP**N**GTAVLFGSTPIPDTT**N**LFVTDASFGATILGVDDYGKASTL

YKTVIPGQDATCWVAICPATHTAFVTDIRV**N**RLVEMSLV**N**AEIIGEPIDLTTF**NN**DPGLTEIRSSGSFVYALSPG**N**GTTEAWITVL**N**ALTKKPVQHALL

TPLGLDR**N**AMGMAILV

------------------------------------------------

Position Residue Score Prediction

------------------------------------------------

5 **N**KL -0.68032779 Non-glycosylated

25 **N**DR -0.90706694 Non-glycosylated

39 **N**GK -1.2018316 Non-glycosylated

48 **N**TA -1.28992 Non-glycosylated

52 **N**SV -0.63933703 Non-glycosylated

**62 NGS 1.1913176 Potential Glycosylated**

68 **N**HA -0.93420911 Non-glycosylated

81 **N**GI -1.2485877 Non-glycosylated

84 **N**PR -1.8602168 Non-glycosylated

112 **N**AG -1.1497502 Non-glycosylated

116 **N**TV -0.70159048 Non-glycosylated

146 **N**TV -1.1767199 Non-glycosylated

155 **N**LV -0.99315998 Non-glycosylated

**190 NQT 0.98748688 Potential Glycosylated**

200 **N**TV -0.97636502 Non-glycosylated

**225 NNT 0.83519615 Potential Glycosylated**

226 **N**TG -1.089221 Non-glycosylated

237 **N**IH -1.0717965 Non-glycosylated

**258 NGT 1.0356973 Potential Glycosylated**

274 **N**LF -1.2838351 Non-glycosylated

329 **N**RL -1.1062136 Non-glycosylated

338 **N**AE -1.0761808 Non-glycosylated

352 **NN**D -1.2714523 Non-glycosylated

353 **N**DP -1.0824142 Non-glycosylated

**374 NGT 1.2707122 Potential Glycosylated**

385 **N**AL -1.0307742 Non-glycosylated

405 **N**AM -1.3410327 Non-glycosylated

***********************************

>Ss472 Length = 413

**Potential N-Linked Glycosylated Sites:**

MHYY**N**KLKLLALASVISVTSAHPT**N**DRYYADADSEACL**N**GKAVYVTS**N**TAH**N**SVVALPIAR**N**GSLLV**N**HATSTATGGSGG**N**GI**N**PRGMPAGPDALFGQGS

ITVAGDYLFSV**N**AGS**N**TVTMLAIDKHDPTKLTVVGEPAVLPGEFP**N**TVGASDKF**N**LVCVGLTGAKAGVSCASYSWYGLGPFDELRPFDL**N**QTTPPHGPT

**N**TVSHVFFSGDQETVFATVKGDPTV**NN**TGFLAAYPVE**N**IHTSCYATPSVSYKGVMSSP**N**GTAVLFGSTPIPDTT**N**LFVTDASFGATILGVDDYGKASTL

YKTVIPGQDATCWVAICPATHTAFVTDIRV**N**RLVEMSLV**N**AEIIGEPIDLTTF**NN**DPGLTEIRSSGSFVYALSPG**N**GTTEAWITVL**N**ALTKKPVQHALL

TPLGLDR**N**AMGMAILV

------------------------------------------------

Position Residue Score Prediction

------------------------------------------------

5 **N**KL -0.68032779 Non-glycosylated

25 **N**DR -0.90706694 Non-glycosylated

39 **N**GK -1.2018316 Non-glycosylated

48 **N**TA -1.28992 Non-glycosylated

52 **N**SV -0.63933703 Non-glycosylated

**62 NGS 1.1913176 Potential Glycosylated**

68 **N**HA -0.93420911 Non-glycosylated

81 **N**GI -1.2485877 Non-glycosylated

84 **N**PR -1.8602168 Non-glycosylated

112 **N**AG -1.1497502 Non-glycosylated

116 **N**TV -0.70159048 Non-glycosylated

146 **N**TV -1.1767199 Non-glycosylated

155 **N**LV -0.99315998 Non-glycosylated

**190 NQT 0.98748688 Potential Glycosylated**

200 **N**TV -0.97636502 Non-glycosylated

**225 NNT 0.83519615 Potential Glycosylated**

226 **N**TG -1.089221 Non-glycosylated

237 **N**IH -1.0717965 Non-glycosylated

**258 NGT 1.0356973 Potential Glycosylated**

274 **N**LF -1.2838351 Non-glycosylated

329 **N**RL -1.1062136 Non-glycosylated

338 **N**AE -1.0761808 Non-glycosylated

352 **NN**D -1.2714523 Non-glycosylated

353 **N**DP -1.0824142 Non-glycosylated

**374 NGT 1.2707122 Potential Glycosylated**

385 **N**AL -1.0307742 Non-glycosylated

405 **N**AM -1.3410327 Non-glycosylated

***********************************

>Ss236 Length = 413

**Potential N-Linked Glycosylated Sites:**

MHYY**N**KLKLLALASVISVTSAHPT**N**DRYYADADSEACL**N**GKAVYVTS**N**TAH**N**SVVALPIAR**N**GSLLV**N**HATSTATGGSGG**N**GI**N**PRGMPAGPDALFGQGS

ITVAGDYLFSV**N**AGS**N**TVTMLAIDKHDPTKLTVVGEPAVLPGEFP**N**TVGASDKF**N**LVCVGLTGAKAGVSCASYSWYGLGPFDELRPFDL**N**QTTPPHGPT

**N**TVSHVFFSGDQETVFATVKGDPTV**NN**TGFLAAYPVE**N**IHTSCYATPSVSYKGVMSSP**N**GTAVLFGSTPIPDTT**N**LFVTDASFGATILGVDDYGKASTL

YKTVIPGQDATCWVAICPATHTAFVTDIRV**N**RLVEMSLV**N**AEIIGEPIDLTTF**NN**DPGLTEIRSSGSFVYALSPG**N**GTTEAWITVL**N**ALTKKPVQHALL

TPLGLDR**N**AMGMAILV

------------------------------------------------

Position Residue Score Prediction

------------------------------------------------

5 **N**KL -0.68032779 Non-glycosylated

25 **N**DR -0.90706694 Non-glycosylated

39 **N**GK -1.2018316 Non-glycosylated

48 **N**TA -1.28992 Non-glycosylated

52 **N**SV -0.63933703 Non-glycosylated

**62 NGS 1.1913176 Potential Glycosylated**

68 **N**HA -0.93420911 Non-glycosylated

81 **N**GI -1.2485877 Non-glycosylated

84 **N**PR -1.8602168 Non-glycosylated

112 **N**AG -1.1497502 Non-glycosylated

116 **N**TV -0.70159048 Non-glycosylated

146 **N**TV -1.1767199 Non-glycosylated

155 **N**LV -0.99315998 Non-glycosylated

**190 NQT 0.98748688 Potential Glycosylated**

200 **N**TV -0.97636502 Non-glycosylated

**225 NNT 0.83519615 Potential Glycosylated**

226 **N**TG -1.089221 Non-glycosylated

237 **N**IH -1.0717965 Non-glycosylated

**258 NGT 1.0356973 Potential Glycosylated**

274 **N**LF -1.2838351 Non-glycosylated

329 **N**RL -1.1062136 Non-glycosylated

338 **N**AE -1.0761808 Non-glycosylated

352 **NN**D -1.2714523 Non-glycosylated

353 **N**DP -1.0824142 Non-glycosylated

**374 NGT 1.2707122 Potential Glycosylated**

385 **N**AL -1.0307742 Non-glycosylated

405 **N**AM -1.3410327 Non-glycosylated

***********************************

>Ss520 Length = 413

**Potential N-Linked Glycosylated Sites:**

MHYY**N**KLKLLALASVISVTSAHPT**N**DRYYADADSEACL**N**GKAVYVTS**N**TAH**N**SVVALPIAR**N**GSLLV**N**HATSTATGGSGG**N**GI**N**PRGMPAGPDALFGQGS

ITVAGDYLFSV**N**AGS**N**TVTMLAIDKHDPTKLTVVGEPAVLPGEFP**N**TVGASDKF**N**LVCVGLTGAKAGVSCASYSWYGLGPFDELRPFDL**N**QTTPPHGPT

**N**TVSHVFFSGDQETVFATVKGDPTV**NN**TGFLAAYPVE**N**IHTSCYATPSVSYKGVMSSP**N**GTAVLFGSTPIPDTT**N**LFVTDASFGATILGVDDYGKASTL

YKTVIPGQDATCWVAICPATHTAFVTDIRV**N**RLVEMSLV**N**AEIIGEPIDLTTF**NN**DPGLTEIRSSGSFVYALSPG**N**GTTEAWITVL**N**ALTKKPVQ**N**ALL

TPLGLDR**N**AMGMAILV

------------------------------------------------

Position Residue Score Prediction

------------------------------------------------

5 **N**KL -0.68032779 Non-glycosylated

25 **N**DR -0.90706694 Non-glycosylated

39 **N**GK -1.2018316 Non-glycosylated

48 **N**TA -1.28992 Non-glycosylated

52 **N**SV -0.63933703 Non-glycosylated

**62 NGS 1.1913176 Potential Glycosylated**

68 **N**HA -0.93420911 Non-glycosylated

81 **N**GI -1.2485877 Non-glycosylated

84 **N**PR -1.8602168 Non-glycosylated

112 **N**AG -1.1497502 Non-glycosylated

116 **N**TV -0.70159048 Non-glycosylated

146 **N**TV -1.1767199 Non-glycosylated

155 **N**LV -0.99315998 Non-glycosylated

**190 NQT 0.98748688 Potential Glycosylated**

200 **N**TV -0.97636502 Non-glycosylated

**225 NNT 0.83519615 Potential Glycosylated**

226 **N**TG -1.089221 Non-glycosylated

237 **N**IH -1.0717965 Non-glycosylated

**258 NGT 1.0356973 Potential Glycosylated**

274 **N**LF -1.2838351 Non-glycosylated

329 **N**RL -1.1062136 Non-glycosylated

338 **N**AE -1.0761808 Non-glycosylated

352 **NN**D -1.2714523 Non-glycosylated

353 **N**DP -1.0824142 Non-glycosylated

**374 NGT 1.2707122 Potential Glycosylated**

385 **N**AL -1.1562567 Non-glycosylated

394 **N**AL -0.95337747 Non-glycosylated

405 **N**AM -1.3410327 Non-glycosylated

***********************************

>5659 Length = 413

**Potential N-Linked Glycosylated Sites:**

MHYY**N**KLKLLALASVISVTSAHPT**N**DRYYADADSEACL**N**GKAVYVTS**N**TAH**N**SVVALPIAR**N**GSLLV**N**HATSTATGGSGG**N**GI**N**PRGMPAGPDALFGQGS

ITVAGDYLFSV**N**AGS**N**TVTMLAIDKHDPTKLTVVGEPAVLPGEFP**N**TVGASDKF**N**LVCVGLTGAKAGVSCASYSWYGLGPFDELRPFDL**N**QTTPPHGPT

**N**TVSHVFFSGDQETVFATVKGDPTV**NN**TGFLAAYPVE**N**IHTSCYATPSVSYKGVMSSP**N**GTAVLFGSTPIPDTT**N**LFVTDASFGATILGVDDYGKASTL

YKTVIPGQDATCWVAICPATHTAFVTDIRV**N**RLVEMSLV**N**AEIIGEPIDLTTF**NN**DPGLTEIRSSGSFVYAWSPG**N**GTTEAWITVL**N**ALTKKPVQHALL

TPLGLDR**N**AMGMAILV

------------------------------------------------

Position Residue Score Prediction

------------------------------------------------

5 **N**KL -0.68032779 Non-glycosylated

25 **N**DR -0.90706694 Non-glycosylated

39 **N**GK -1.2018316 Non-glycosylated

48 **N**TA -1.28992 Non-glycosylated

52 **N**SV -0.63933703 Non-glycosylated

**62 NGS 1.1913176 Potential Glycosylated**

68 **N**HA -0.93420911 Non-glycosylated

81 **N**GI -1.2485877 Non-glycosylated

84 **N**PR -1.8602168 Non-glycosylated

112 **N**AG -1.1497502 Non-glycosylated

116 **N**TV -0.70159048 Non-glycosylated

146 **N**TV -1.1767199 Non-glycosylated

155 **N**LV -0.99315998 Non-glycosylated

**190 NQT 0.98748688 Potential Glycosylated**

200 **N**TV -0.97636502 Non-glycosylated

**225 NNT 0.83519615 Potential Glycosylated**

226 **N**TG -1.089221 Non-glycosylated

237 **N**IH -1.0717965 Non-glycosylated

**258 NGT 1.0356973 Potential Glycosylated**

274 **N**LF -1.2838351 Non-glycosylated

329 **N**RL -1.1062136 Non-glycosylated

338 **N**AE -1.0761808 Non-glycosylated

352 **NN**D -1.2714523 Non-glycosylated

353 **N**DP -1.0824142 Non-glycosylated

**374 NGT 1.3092983 Potential Glycosylated**

385 **N**AL -1.0307742 Non-glycosylated

405 **N**AM -1.3410327 Non-glycosylated

***********************************

>Ss41 Length = 413

**Potential N-Linked Glycosylated Sites:**

MHYY**N**KLKLLALASVISVTSAHPT**N**DRYYADADSEACL**N**GKAVYVTS**N**TAH**N**SVVALPIAR**N**GSLLV**N**HATSTATGGSGG**N**GI**N**PRGMPAGPDALFGQGS

ITVAGDYLFSV**N**AGS**N**TVTMLAIDKHDPTKLTVVGEPAVLPGEFP**N**TVGASDKF**N**LVCVGLTGAKAGVSCASYSWYGLGPFDELRPFDL**N**QTTPPHGPT

**N**TVSHVFFSGDQETVFATVKGDPTV**NN**TGFLAAYPVE**N**IQTSCYATPSVSYKGVMSSP**N**GTAVLFGSTPIPDTT**N**LFVTDASFGATILGVDDYGKASTL

YKTVIPGQDATCWVAICPATHTAFVTDIRV**N**RLVEMSLV**N**AEIIGEPIDLTTF**NN**DPGLTEIRSSGSFVYALSPG**N**GTTEAWITVL**N**ALTKKPVQHALL

TPLGLDR**N**AMGMAILV

------------------------------------------------

Position Residue Score Prediction

------------------------------------------------

5 **N**KL -0.68032779 Non-glycosylated

25 **N**DR -0.90706694 Non-glycosylated

39 **N**GK -1.2018316 Non-glycosylated

48 **N**TA -1.28992 Non-glycosylated

52 **N**SV -0.63933703 Non-glycosylated

**62 NGS 1.1913176 Potential Glycosylated**

68 **N**HA -0.93420911 Non-glycosylated

81 **N**GI -1.2485877 Non-glycosylated

84 **N**PR -1.8602168 Non-glycosylated

112 **N**AG -1.1497502 Non-glycosylated

116 **N**TV -0.70159048 Non-glycosylated

146 **N**TV -1.1767199 Non-glycosylated

155 **N**LV -0.99315998 Non-glycosylated

**190 NQT 0.98748688 Potential Glycosylated**

200 **N**TV -0.97636502 Non-glycosylated

**225 NNT 0.83519615 Potential Glycosylated**

226 **N**TG -1.089221 Non-glycosylated

237 **N**IQ -0.80909324 Non-glycosylated

**258 NGT 1.0356973 Potential Glycosylated**

274 **N**LF -1.2838351 Non-glycosylated

329 **N**RL -1.1062136 Non-glycosylated

338 **N**AE -1.0761808 Non-glycosylated

352 **NN**D -1.2714523 Non-glycosylated

353 **N**DP -1.0824142 Non-glycosylated

**374 NGT 1.2707122 Potential Glycosylated**

385 **N**AL -1.0307742 Non-glycosylated

405 **N**AM -1.3410327 Non-glycosylated

***********************************

>Ss334 Length = 413

**Potential N-Linked Glycosylated Sites:**

MHYY**N**KLKLLALASVISVTSAHPT**N**DRYYADADSEACL**N**GKAVYVTS**N**TAH**N**SVVALPIAR**N**GSLLV**N**HATSTATGGSGG**N**GI**N**PRGMPAGPDALFGQGS

ITVAGDYLFSV**N**AGS**N**TVTMLAIDKHDPTKLTVVGEPAVLPGEFP**N**TVGASDKF**N**LVCVGLTGAKAGVSCASYSWYGLGPFDELRPFDL**N**QTTPPHGPT

**N**TVSHVFFSGDQETVFATVKGDPTV**NN**TGFLAAYPVE**N**IQTSCYATPSVSYKGVMSSP**N**GTAVLFGSTPIPDTT**N**LFVTDASFGATILGVDDYGKASTL

YKTVIPGQDATCWVAICPATHTAFVTDIRV**N**RLVEMSLV**N**AEIIGEPIDLTTF**NN**DPGLTEIRSSGSFVYALSPG**N**GTTEAWITVL**N**ALTKKPVQHALL

TPLGLDR**N**AMGMAILV

------------------------------------------------

Position Residue Score Prediction

------------------------------------------------

5 **N**KL -0.68032779 Non-glycosylated

25 **N**DR -0.90706694 Non-glycosylated

39 **N**GK -1.2018316 Non-glycosylated

48 **N**TA -1.28992 Non-glycosylated

52 **N**SV -0.63933703 Non-glycosylated

**62 NGS 1.1913176 Potential Glycosylated**

68 **N**HA -0.93420911 Non-glycosylated

81 **N**GI -1.2485877 Non-glycosylated

84 **N**PR -1.8602168 Non-glycosylated

112 **N**AG -1.1497502 Non-glycosylated

116 **N**TV -0.70159048 Non-glycosylated

146 **N**TV -1.1767199 Non-glycosylated

155 **N**LV -0.99315998 Non-glycosylated

**190 NQT 0.98748688 Potential Glycosylated**

200 **N**TV -0.97636502 Non-glycosylated

**225 NNT 0.83519615 Potential Glycosylated**

226 **N**TG -1.089221 Non-glycosylated

237 **N**IQ -0.80909324 Non-glycosylated

**258 NGT 1.0356973 Potential Glycosylated**

274 **N**LF -1.2838351 Non-glycosylated

329 **N**RL -1.1062136 Non-glycosylated

338 **N**AE -1.0761808 Non-glycosylated

352 **NN**D -1.2714523 Non-glycosylated

353 **N**DP -1.0824142 Non-glycosylated

**374 NGT 1.2707122 Potential Glycosylated**

385 **N**AL -1.0307742 Non-glycosylated

405 **N**AM -1.3410327 Non-glycosylated

***********************************

>Ss49 Length = 413

**Potential N-Linked Glycosylated Sites:**

MHYY**N**KLKLLALASVISVTSAHPT**N**DRYYADADSEACL**N**GKAVYVTS**N**TAH**N**SVVALPIAR**N**GSLLV**N**HATSTATGGSGG**N**GI**N**PRGMPAGPDALFGQGS

ITVAGDYLFSV**N**AGS**N**TVTMLAIDKHDPTKLTVVGEPAVLPGEFP**N**TVGASDKF**N**LVCVGLTGAKAGVSCASYSWYGLGPFDELRPFDL**N**QTTPPHGPT

**N**TVSHVFFSGDQETVFATVKGDPTV**NN**TGFLAAYPVE**N**IQTSCYATPSVSYKGVMSSP**N**GTAVLFGSTPIPDTT**N**LFVTDASFGATILGVDDYGKASTL

YKTVIPGQDATCWVAICPATHTAFVTDIRV**N**RLVEMSLV**N**AEIIGEPIDLTTF**NN**DPGLTEIRSSGSFVYALSPG**N**GTTEAWITVL**N**ALTKKPVQHALL

TQLGLDR**N**AMGMAILV

------------------------------------------------

Position Residue Score Prediction

------------------------------------------------

5 **N**KL -0.68032779 Non-glycosylated

25 **N**DR -0.90706694 Non-glycosylated

39 **N**GK -1.2018316 Non-glycosylated

48 **N**TA -1.28992 Non-glycosylated

52 **N**SV -0.63933703 Non-glycosylated

**62 NGS 1.1913176 Potential Glycosylated**

68 **N**HA -0.93420911 Non-glycosylated

81 **N**GI -1.2485877 Non-glycosylated

84 **N**PR -1.8602168 Non-glycosylated

112 **N**AG -1.1497502 Non-glycosylated

116 **N**TV -0.70159048 Non-glycosylated

146 **N**TV -1.1767199 Non-glycosylated

155 **N**LV -0.99315998 Non-glycosylated

**190 NQT 0.98748688 Potential Glycosylated**

200 **N**TV -0.97636502 Non-glycosylated

**225 NNT 0.83519615 Potential Glycosylated**

226 **N**TG -1.089221 Non-glycosylated

237 **N**IQ -0.80909324 Non-glycosylated

**258 NGT 1.0356973 Potential Glycosylated**

274 **N**LF -1.2838351 Non-glycosylated

329 **N**RL -1.1062136 Non-glycosylated

338 **N**AE -1.0761808 Non-glycosylated

352 **NN**D -1.2714523 Non-glycosylated

353 **N**DP -1.0824142 Non-glycosylated

**374 NGT 1.2707122 Potential Glycosylated**

385 **N**AL -1.0307742 Non-glycosylated

405 **N**AM -1.3766757 Non-glycosylated

***********************************

>S17 Length = 413

**Potential N-Linked Glycosylated Sites:**

MHYY**N**KLKLLALASVISVTSAHPT**N**DRYYADADSEACL**N**GKAVYVTS**N**TAH**N**SVVALPIAR**N**GSLLV**N**HATSTATGGSGG**N**GI**N**PRGMPAGPDALFGQGS

ITVAGDYLFSV**N**AGS**N**TVTMLAIDKHDPTKLTVVGEPAVLPGEFP**N**TVGASDKF**N**LVCVGLTGAKAGVSCASYSWYGLGPFDELRPFDL**N**QTTPPHGPT

**N**TVSHVFFSGDQETVFATVKGDPTV**NN**TGFLAAYPVE**N**IHTSCYATPSVSYKGVMSSP**N**GTAVLFGSTPIPDTT**N**LFVTDASFGATILGVDDYGKASTL

YKTVIPGQDATCWVAICPATHTAFVTDIRV**N**RLVEMSLV**N**AEIIGEPIDLTTF**NN**DPGLTEIRSSGSFVYALSPG**N**GTTEAWITVL**N**ALTKKPVQHALL

TPLGLDR**N**AMGMAILV

------------------------------------------------

Position Residue Score Prediction

------------------------------------------------

5 **N**KL -0.68032779 Non-glycosylated

25 **N**DR -0.90706694 Non-glycosylated

39 **N**GK -1.2018316 Non-glycosylated

48 **N**TA -1.28992 Non-glycosylated

52 **N**SV -0.63933703 Non-glycosylated

**62 NGS 1.1913176 Potential Glycosylated**

68 **N**HA -0.93420911 Non-glycosylated

81 **N**GI -1.2485877 Non-glycosylated

84 **N**PR -1.8602168 Non-glycosylated

112 **N**AG -1.1497502 Non-glycosylated

116 **N**TV -0.70159048 Non-glycosylated

146 **N**TV -1.1767199 Non-glycosylated

155 **N**LV -0.99315998 Non-glycosylated

**190 NQT 0.98748688 Potential Glycosylated**

200 **N**TV -0.97636502 Non-glycosylated

**225 NNT 0.83519615 Potential Glycosylated**

226 **N**TG -1.089221 Non-glycosylated

237 **N**IH -1.0717965 Non-glycosylated

**258 NGT 1.0356973 Potential Glycosylated**

274 **N**LF -1.2838351 Non-glycosylated

329 **N**RL -1.1062136 Non-glycosylated

338 **N**AE -1.0761808 Non-glycosylated

352 **NN**D -1.2714523 Non-glycosylated

353 **N**DP -1.0824142 Non-glycosylated

**374 NGT 1.2707122 Potential Glycosylated**

385 **N**AL -1.0307742 Non-glycosylated

405 **N**AM -1.3410327 Non-glycosylated

***********************************

>SS01 Length = 413

**Potential N-Linked Glycosylated Sites:**

MHYY**N**KLKLLALASVISVTSAHPT**N**DRYYADADSEACL**N**GKAVYVTS**N**TAH**N**SVVALPIAR**N**GSLLV**N**HATSTATGGSGG**N**GI**N**PRGMPAGPDALFGQGS

ITVAGDYLFSV**N**AGS**N**TVTMLAIDKHDPTKLTVVGEPAVLPGEFP**N**TVGASDKF**N**LVCVGLTGAKAGVSCASYSWYGLGPFDELRPFDL**N**QTTPPHGPT

**N**TVSHVFFSGDQETVFATVKGDPTV**NN**TGFLAAYPVE**N**IHTSCYATPSVSYKGVMSSP**N**GTAVLFGSTPIPDTT**N**LFVTDASFGATILGVDDYGKASTL

YKTVIPGQDATCWVAICPATHTAFVTDIRV**N**RLVEMSLV**N**AEIIGEPIDLTTF**NN**DPGLTEIRSSGSFVYALSPG**N**GTTEAWITVL**N**ALTKKPVQHALL

TPLGLDR**N**AMGMAILV

------------------------------------------------

Position Residue Score Prediction

------------------------------------------------

5 **N**KL -0.68032779 Non-glycosylated

25 **N**DR -0.90706694 Non-glycosylated

39 **N**GK -1.2018316 Non-glycosylated

48 **N**TA -1.28992 Non-glycosylated

52 **N**SV -0.63933703 Non-glycosylated

**62 NGS 1.1913176 Potential Glycosylated**

68 **N**HA -0.93420911 Non-glycosylated

81 **N**GI -1.2485877 Non-glycosylated

84 **N**PR -1.8602168 Non-glycosylated

112 **N**AG -1.1497502 Non-glycosylated

116 **N**TV -0.70159048 Non-glycosylated

146 **N**TV -1.1767199 Non-glycosylated

155 **N**LV -0.99315998 Non-glycosylated

**190 NQT 0.98748688 Potential Glycosylated**

200 **N**TV -0.97636502 Non-glycosylated

**225 NNT 0.83519615 Potential Glycosylated**

226 **N**TG -1.089221 Non-glycosylated

237 **N**IH -1.0717965 Non-glycosylated

**258 NGT 1.0356973 Potential Glycosylated**

274 **N**LF -1.2838351 Non-glycosylated

329 **N**RL -1.1062136 Non-glycosylated

338 **N**AE -1.0761808 Non-glycosylated

352 **NN**D -1.2714523 Non-glycosylated

353 **N**DP -1.0824142 Non-glycosylated

**374 NGT 1.2707122 Potential Glycosylated**

385 **N**AL -1.0307742 Non-glycosylated

405 **N**AM -1.3410327 Non-glycosylated

***********************************

>Ss534 Length = 413

**Potential N-Linked Glycosylated Sites:**

MHYY**N**KLKLLALDSVISVTSAHPT**N**DRYYADADSEACL**N**GKAVYVTS**N**TAH**N**SVVALPIAR**N**GSLLV**N**HATSTATGGSGG**N**GI**N**PRGMPAGPDELFGQGS

ITVAGDYLFSV**N**AGS**N**TVTMLAIDKHDPTKLTVVGEPAVLPGEFP**N**TVGASDKF**N**LVCVGLTGAKAGVSCASYSWYGLGPFDELRPFDL**N**QTTPPHGPT

**N**TVSHVFFSGDQETVFATVKGDPTV**NN**TGFLAAYPVE**N**IHTSCYATPSVSYKGVMSSP**N**GTAVLFGSTPIPDTT**N**LFVTDASFGATILGVDDYGKASTL

YKTVIPGQDATCWVAICPATHTAFVTDIRV**N**RLVEMSLM**N**AEIIGEPIDLTTF**NN**DPGLTEIRSSGSFVYALSPG**N**GTTEAWITVL**N**ALTK**N**PVQHA**N**L

RPLGLDR**N**AMGMAILV

------------------------------------------------

Position Residue Score Prediction

------------------------------------------------

5 **N**KL -0.66881027 Non-glycosylated

25 **N**DR -0.90706694 Non-glycosylated

39 **N**GK -1.2018316 Non-glycosylated

48 **N**TA -1.28992 Non-glycosylated

52 **N**SV -0.63933703 Non-glycosylated

**62 NGS 1.1913176 Potential Glycosylated**

68 **N**HA -0.93420911 Non-glycosylated

81 **N**GI -1.2485877 Non-glycosylated

84 **N**PR -1.8312927 Non-glycosylated

112 **N**AG -1.1497502 Non-glycosylated

116 **N**TV -0.70159048 Non-glycosylated

146 **N**TV -1.1767199 Non-glycosylated

155 **N**LV -0.99315998 Non-glycosylated

**190 NQT 0.98748688 Potential Glycosylated**

200 **N**TV -0.97636502 Non-glycosylated

**225 NNT 0.83519615 Potential Glycosylated**

226 **N**TG -1.089221 Non-glycosylated

237 **N**IH -1.0717965 Non-glycosylated

**258 NGT 1.0356973 Potential Glycosylated**

274 **N**LF -1.2838351 Non-glycosylated

329 **N**RL -1.0185371 Non-glycosylated

338 **N**AE -1.0709863 Non-glycosylated

352 **NN**D -1.2714523 Non-glycosylated

353 **N**DP -1.0824142 Non-glycosylated

**374 NGT 1.2707122 Potential Glycosylated**

385 **N**AL -0.96353409 Non-glycosylated

390 **N**PV -1.1038253 Non-glycosylated

396 **N**LR -1.2686344 Non-glycosylated

405 **N**AM -1.390216 Non-glycosylated

***********************************

>Ss545 Length = 413

**Potential N-Linked Glycosylated Sites:**

MHYY**N**KLKLLALDSVISVTSAHPT**N**DRYYADADSEACL**N**GKAVYVTS**N**TAH**N**SVVALPIARTGSLLV**N**HATSTATGGSGG**N**GI**N**PRGMPAGPDALIGQGS

ITVAGDYLFSV**N**AGS**N**TVTMLAIDKHDPTKLTVVGEPAVLPGEFP**N**TVGASDKF**N**LVCVGLTGAKAGVSCASYSWYGLGPFDELRPFDL**N**QTTPPHGPT

**N**TVSHVFFSGDQETVFATVKGDPTV**NN**TGFLAAYPVE**N**IHTSCYATPSVSYKGVMSSP**N**GTAVLFGSTPIPDTT**N**LFVTDASFGATILGVDDYGKASTL

YKTVIPGQDATCWVAICPATHTAFVTDIRV**N**RLVEMSLM**N**AEIIGEPIDLTTF**NN**DPGLTEIRSSGSFVYALSPG**N**GTTEAWITVL**N**ALTK**N**PVQHA**N**L

RPLGLDR**N**AMGMAILV

------------------------------------------------

Position Residue Score Prediction

------------------------------------------------

5 **N**KL -0.66881027 Non-glycosylated

25 **N**DR -0.90706694 Non-glycosylated

39 **N**GK -1.2018316 Non-glycosylated

48 **N**TA -1.28992 Non-glycosylated

52 **N**SV -0.6848634 Non-glycosylated

68 **N**HA -1.022878 Non-glycosylated

81 **N**GI -1.2485877 Non-glycosylated

84 **N**PR -1.8602168 Non-glycosylated

112 **N**AG -1.1497502 Non-glycosylated

116 **N**TV -0.70159048 Non-glycosylated

146 **N**TV -1.1767199 Non-glycosylated

155 **N**LV -0.99315998 Non-glycosylated

**190 NQT 0.98748688 Potential Glycosylated**

200 **N**TV -0.97636502 Non-glycosylated

**225 NNT 0.83519615 Potential Glycosylated**

226 **N**TG -1.089221 Non-glycosylated

237 **N**IH -1.0717965 Non-glycosylated

**258 NGT 1.0356973 Potential Glycosylated**

274 **N**LF -1.2838351 Non-glycosylated

329 **N**RL -1.0185371 Non-glycosylated

338 **N**AE -1.0709863 Non-glycosylated

352 **NN**D -1.2714523 Non-glycosylated

353 **N**DP -1.0824142 Non-glycosylated

**374 NGT 1.2707122 Potential Glycosylated**

385 **N**AL -0.96353409 Non-glycosylated

390 **N**PV -1.1038253 Non-glycosylated

396 **N**LR -1.2686344 Non-glycosylated

405 **N**AM -1.390216 Non-glycosylated

***********************************

>Ss187 Length = 391

**Potential N-Linked Glycosylated Sites:**

MHYH**NN**LKLLALASVASLTSAYPTIEHVGVES**N**ACI**N**GRAVYVTS**N**KEH**N**AVVALPIAR**N**GTLLVDLATSTATGGSGG**N**VLTKQYLFAV**N**AGS**N**TLTMLA

IDKHDPTKLTVVGEPAMLPGEFP**N**TVGASDKF**N**LVCVGLTGAKAGVSCASYSWYGLGPFDEIRPFEL**N**QTTPPHGPT**N**TVADVFFSGDQRTVFVTVKGD

PAV**NN**TGFLAAYPVE**N**IHSSCYAIPSVSHKGVISSP**N**GTAVLFGSTPIPDTT**N**LFVTDASFGAAILDI**N**DYGEASTLYKTVIAGQDATCWVAICPATHT

AFVTDIRM**N**RLVEMSLV**N**ARIIGDPIDLTPL**NN**DPGLTDIQSGGSFVYALSPG**N**GTTEAWISVL**N**AFTKELVQHALLTPLGLDR**N**TMGMAILV

------------------------------------------------

Position Residue Score Prediction

------------------------------------------------

5 **NN**L -0.64418282 Non-glycosylated

6 **N**LK -0.67198443 Non-glycosylated

33 **N**AC -1.0785908 Non-glycosylated

37 **N**GR -0.96723568 Non-glycosylated

46 **N**KE -1.012695 Non-glycosylated

50 **N**AV -0.82412323 Non-glycosylated

**60 NGT 1.0939099 Potential Glycosylated**

79 **N**VL -1.0705938 Non-glycosylated

90 **N**AG -1.1207349 Non-glycosylated

94 **N**TL -0.53380037 Non-glycosylated

124 **N**TV -1.1766113 Non-glycosylated

133 **N**LV -0.99315998 Non-glycosylated

**168 NQT 0.99623604 Potential Glycosylated**

178 **N**TV -1.0736751 Non-glycosylated

**203 NNT 0.90735217 Potential Glycosylated**

204 **N**TG -1.0185101 Non-glycosylated

215 **N**IH -1.1756742 Non-glycosylated

**236 NGT 0.84744638 Potential Glycosylated**

252 **N**LF -1.2838351 Non-glycosylated

268 **N**DY -0.91366625 Non-glycosylated

307 **N**RL -1.0665658 Non-glycosylated

316 **N**AR -1.218827 Non-glycosylated

330 **NN**D -1.3223533 Non-glycosylated

331 **N**DP -1.3079132 Non-glycosylated

**352 NGT 1.1865941 Potential Glycosylated**

363 **N**AF -1.1909572 Non-glycosylated

383 **N**TM -1.3285687 Non-glycosylated

**Supplementary Table S5.** Predicted *N*-linked glycosylation sites and their conservation across *Sordariomycetes* species using GlycoEP [10].

*Note: No. of sequences=74. Threshold Selected: 0.0****.***

>XP_016591720Sporothrix_sc Length = 413

**Potential N-Linked Glycosylated Sites:**

MHYY**N**KLKFLALASVFSVTSAHPTSGHYYDDAESEACL**N**GKAVYVTS**N**TEH**N**SVVAIPIAR**N**GSLLL**N**HATSTATGGRGG**N**GI**N**PRGMPAGPDALFSQGS

ITSAG**N**YLFAV**N**AGS**N**TVTMLAIDEHDPTKVTVVGEPAELPGEFPTTVGASDKF**N**LVCVGLTGAKAGVSCASYSWYGLGPFDELRTFDLHQTTPPHGPT

**N**TVSHVFFSGDQETVFTTVKGDPAV**NN**TGFLAAYPVEHIHSSCYAIPSVSHKGVISSPDGTAVLFGSTPIPDTT**N**LFVTDASFGAAILGIDDYEEASTL

YKTVIPGQDATCWVAICPATHTAFVTDIRM**N**RLVEMSLV**N**AEIIGEPIDLTTF**N**TDPGLTEIRSGGSFVYALSPG**N**GTTEAWITVL**N**ALTKKPVQHALL

TPLGLDR**N**AMGMAILV

------------------------------------------------

Position Residue Score Prediction

------------------------------------------------

5 **N**KL -0.77832304 Non-glycosylated

39 **N**GK -1.1287597 Non-glycosylated

48 **N**TE -1.2741531 Non-glycosylated

52 **N**SV -0.83972275 Non-glycosylated

**62 NGS 1.1882916 Potential Glycosylated**

68 **N**HA -0.89887659 Non-glycosylated

81 **N**GI -1.2423291 Non-glycosylated

84 **N**PR -1.8662045 Non-glycosylated

106 **N**YL -0.7359858 Non-glycosylated

112 **N**AG -0.84383581 Non-glycosylated

116 **N**TV -0.70783397 Non-glycosylated

155 **N**LV -1.0444821 Non-glycosylated

200 **N**TV -1.0299987 Non-glycosylated

**225 NNT 0.88963983 Potential Glycosylated**

226 **N**TG -1.0135771 Non-glycosylated

274 **N**LF -1.2838351 Non-glycosylated

329 **N**RL -1.0665658 Non-glycosylated

338 **N**AE -1.1315083 Non-glycosylated

352 **N**TD -1.3580699 Non-glycosylated

**374 NGT 1.2707122 Potential Glycosylated**

385 **N**AL -1.0307742 Non-glycosylated

405 **N**AM -1.3410327 Non-glycosylated

***********************************

>ERS98762Sporothrix_schenc Length = 413

**Potential N-Linked Glycosylated Sites:**

MHYY**N**KLKFLALASVFSVTSAHPTSGHYYDDAESEACL**N**GKAVYVTS**N**TEH**N**SVVAIPIAR**N**GSLLL**N**HATSTATGGRGG**N**GI**N**PRGMPAGPDALFSQGS

ITSAG**N**YLFAV**N**AGS**N**TVTMLAIDEHDPTKVTVVGEPAELPGEFPTTVGASDKF**N**LVCVGLTGAKAGVSCASYSWYGLGPFDELRTFDLHQTTPPHGPT

**N**TVSHVFFSGDQETVFTTVKGDPAV**NN**TGFLAAYPVEHIHSSCYAIPSVSHKGVISSPDGTAVLFGSTPIPDTT**N**LFVTDASFGAAILGIDDYEEASTL

YKTVIPGQDATCWVAICPATHTAFVTDIRM**N**RLVEMSLV**N**AEIIGEPIDLTTF**N**TDPGLTEIRSGGSFVYALSPG**N**GTTEAWITVL**N**ALTKKPVQHALL

TPLGLDR**N**AMGMAILV

------------------------------------------------

Position Residue Score Prediction

------------------------------------------------

5 **N**KL -0.77832304 Non-glycosylated

39 **N**GK -1.1287597 Non-glycosylated

48 **N**TE -1.2741531 Non-glycosylated

52 **N**SV -0.83972275 Non-glycosylated

**62 NGS 1.1882916 Potential Glycosylated**

68 **N**HA -0.89887659 Non-glycosylated

81 **N**GI -1.2423291 Non-glycosylated

84 **N**PR -1.8662045 Non-glycosylated

106 **N**YL -0.7359858 Non-glycosylated

112 **N**AG -0.84383581 Non-glycosylated

116 **N**TV -0.70783397 Non-glycosylated

155 **N**LV -1.0444821 Non-glycosylated

200 **N**TV -1.0299987 Non-glycosylated

**225 NNT 0.88963983 Potential Glycosylated**

226 **N**TG -1.0135771 Non-glycosylated

274 **N**LF -1.2838351 Non-glycosylated

329 **N**RL -1.0665658 Non-glycosylated

338 **N**AE -1.1315083 Non-glycosylated

352 **N**TD -1.3580699 Non-glycosylated

**374 NGT 1.2707122 Potential Glycosylated**

385 **N**AL -1.0307742 Non-glycosylated

405 **N**AM -1.3410327 Non-glycosylated

***********************************

>XP_040615995Sporothrix_br Length = 412

**Potential N-Linked Glycosylated Sites:**

MHYY**N**KLKFLALASVISATSAHPTSDHYYADAESEACL**N**GKAVYVTS**N**TEH**N**SVVAIPIAR**N**GSLLV**N**YATSTATGGRGG**N**GI**N**PRGMPAGPDALFGQGS

ITIAGDYLFAV**N**AGS**N**TVTMLAIDKHDPTKVTVVGEPAELPGEFPTTVGASDKF**N**LVCVGLTGAKAGVSCASYSWYGLGPFDELRPFDLHQTTPPHGPT

**N**TVSHVFFSGDQETVFTTVKGDPAV**NN**TGFLAAYPVEHIHSSCYATPSVSHKGVISSPEGTAVLFGSTPIPDTT**N**LFATDASFGAVILGIEDYEASTLY

KTVIPGQDATCWVAICPATHTAFVTDIRM**N**RLVEMSLA**N**AEIIGEPIDLTTFS**N**DPGLTEIRSGGSFVYALSPG**N**GTTEAWITVL**N**ALTKKPVQHALLT

PLGLDR**N**AMGMAILV

------------------------------------------------

Position Residue Score Prediction

------------------------------------------------

5 **N**KL -0.77832304 Non-glycosylated

39 **N**GK -1.130584 Non-glycosylated

48 **N**TE -1.2741531 Non-glycosylated

52 **N**SV -0.83972275 Non-glycosylated

**62 NGS 1.1524071 Potential Glycosylated**

68 **N**YA -0.9795019 Non-glycosylated

81 **N**GI -1.2423291 Non-glycosylated

84 **N**PR -1.8662045 Non-glycosylated

112 **N**AG -1.0674394 Non-glycosylated

116 **N**TV -0.66346953 Non-glycosylated

155 **N**LV -1.0444821 Non-glycosylated

200 **N**TV -1.0299987 Non-glycosylated

**225 NNT 0.88963983 Potential Glycosylated**

226 **N**TG -1.0135771 Non-glycosylated

274 **N**LF -1.3425718 Non-glycosylated

328 **N**RL -1.0222076 Non-glycosylated

337 **N**AE -1.1197133 Non-glycosylated

352 **N**DP -1.1451453 Non-glycosylated

**373 NGT 1.2707122 Potential Glycosylated**

384 **N**AL -1.0307742 Non-glycosylated

404 **N**AM -1.3410327 Non-glycosylated

***********************************

>Sporothrix_globosa Length = 413

**Potential N-Linked Glycosylated Sites:**

MHYY**N**KLKLLALASVISVTSAHPT**N**DRYYADADSEACL**N**GKAVYVTS**N**TAH**N**SVVALPIAR**N**GSLLV**N**HATSTATGGSGG**N**GI**N**PRGMPAGPDALFGQGS

ITVAGDYLFSV**N**AGS**N**TVTMLAIDKHDPTKLTVVGEPAVLPGEFP**N**TVGASDKF**N**LVCVGLTGAKAGVSCASYSWYGLGPFDELRPFDL**N**QTTPPHGPT

**N**TVSHVFFSGDQETVFATVKGDPTV**NN**TGFLAAYPVE**N**IHTSCYATPSVSYKGVMSSP**N**GTAVLFGSTPIPDTT**N**LFVTDASFGATILGVDDYGKASTL

YKTVIPGQDATCWVAICPATHTAFVTDIRV**N**RLVEMSLV**N**AEIIGEPIDLTTF**NN**DPGLTEIRSSGSFVYALSPG**N**GTTEAWITVL**N**ALTKKPVQHALL

TPLGLDR**N**AMGMAILV

------------------------------------------------

Position Residue Score Prediction

------------------------------------------------

5 **N**KL -0.68032779 Non-glycosylated

25 **N**DR -0.90706694 Non-glycosylated

39 **N**GK -1.2018316 Non-glycosylated

48 **N**TA -1.28992 Non-glycosylated

52 **N**SV -0.63933703 Non-glycosylated

**62 NGS 1.1913176 Potential Glycosylated**

68 **N**HA -0.93420911 Non-glycosylated

81 **N**GI -1.2485877 Non-glycosylated

84 **N**PR -1.8602168 Non-glycosylated

112 **N**AG -1.1497502 Non-glycosylated

116 **N**TV -0.70159048 Non-glycosylated

146 **N**TV -1.1767199 Non-glycosylated

155 **N**LV -0.99315998 Non-glycosylated

**190 NQT 0.98748688 Potential Glycosylated**

200 **N**TV -0.97636502 Non-glycosylated

**225 NNT 0.83519615 Potential Glycosylated**

226 **N**TG -1.089221 Non-glycosylated

237 **N**IH -1.0717965 Non-glycosylated

**258 NGT 1.0356973 Potential Glycosylated**

274 **N**LF -1.2838351 Non-glycosylated

329 **N**RL -1.1062136 Non-glycosylated

338 **N**AE -1.0761808 Non-glycosylated

352 **NN**D -1.2714523 Non-glycosylated

353 **N**DP -1.0824142 Non-glycosylated

**374 NGT 1.2707122 Potential Glycosylated**

385 **N**AL -1.0307742 Non-glycosylated

405 **N**AM -1.3410327 Non-glycosylated

***********************************

>Sporothrix_luriei Length = 391

**Potential N-Linked Glycosylated Sites:**

MHYH**NN**LKLLALASVASLTSAYPTIEHVGVES**N**ACI**N**GRAVYVTS**N**KEH**N**AVVALPIAR**N**GTLLVDLATSTATGGSGG**N**VLTKQYLFAV**N**AGS**N**TLTMLA

IDKHDPTKLTVVGEPAMLPGEFP**N**TVGASDKF**N**LVCVGLTGAKAGVSCASYSWYGLGPFDEIRPFEL**N**QTTPPHGPT**N**TVADVFFSGDQRTVFVTVKGD

PAV**NN**TGFLAAYPVE**N**IHSSCYAIPSVSHKGVISSP**N**GTAVLFGSTPIPDTT**N**LFVTDASFGAAILDI**N**DYGEASTLYKTVIAGQDATCWVAICPATHT

AFVTDIRM**N**RLVEMSLV**N**ARIIGDPIDLTPL**NN**DPGLTDIQSGGSFVYALSPG**N**GTTEAWISVL**N**AFTKELVQHALLTPLGLDR**N**TMGMAILV

------------------------------------------------

Position Residue Score Prediction

------------------------------------------------

5 **NN**L -0.64418282 Non-glycosylated

6 **N**LK -0.67198443 Non-glycosylated

33 **N**AC -1.0785908 Non-glycosylated

37 **N**GR -0.96723568 Non-glycosylated

46 **N**KE -1.012695 Non-glycosylated

50 **N**AV -0.82412323 Non-glycosylated

**60 NGT 1.0939099 Potential Glycosylated**

79 **N**VL -1.0705938 Non-glycosylated

90 **N**AG -1.1207349 Non-glycosylated

94 **N**TL -0.53380037 Non-glycosylated

124 **N**TV -1.1766113 Non-glycosylated

133 **N**LV -0.99315998 Non-glycosylated

**168 NQT 0.99623604 Potential Glycosylated**

178 **N**TV -1.0736751 Non-glycosylated

**203 NNT 0.90735217 Potential Glycosylated**

204 **N**TG -1.0185101 Non-glycosylated

215 **N**IH -1.1756742 Non-glycosylated

**236 NGT 0.84744638 Potential Glycosylated**

252 **N**LF -1.2838351 Non-glycosylated

268 **N**DY -0.91366625 Non-glycosylated

307 **N**RL -1.0665658 Non-glycosylated

316 **N**AR -1.218827 Non-glycosylated

330 **NN**D -1.3223533 Non-glycosylated

331 **N**DP -1.3079132 Non-glycosylated

**352 NGT 1.1865941 Potential Glycosylated**

363 **N**AF -1.1909572 Non-glycosylated

383 **N**TM -1.3285687 Non-glycosylated

***********************************

>OAA65787Niveomyces_insect Length = 415

**Potential N-Linked Glycosylated Sites:**

MFSQFLLTSSVFMALTQVATPFPHSS**N**SDKGCQ**N**GRAIYMIS**NN**AD**N**AVVAVPIASDGTLLVDRGTTTATGGVGETGITKAT**N**QSAGPDGLFSQSSLTIA

G**NN**IFAV**N**PGS**N**TVSMFAIDQRDPTKLTLVG**N**PAALLGDFPVTVAASDQ**N**RLVCVGASGARAGVSCASFSAQTGIGAMDGLRSFAL**N**QTTPPEGPL**N**TV

SEVFFSGDESTLFTTVKGDPTV**NN**TGFLASFPVQ**N**ARTTCRAASVGKQATR**N**SP**N**GTAVLFGSKPIPGSS**N**LFVTDASFGAAVLGISSAADIAAGEPGV

VTLGRGVIDGQSATCWATISPAT**N**TAFVTDVGV**N**RLVEMSLTDASIQGQPIDLSA**N**GDPGLIDLVAAGSFIYALSPG**N**GTTEAAVTVL**N**ALTRAQVQHA

QLTSLGL**N**K**N**AQGLAVLV

------------------------------------------------

Position Residue Score Prediction

------------------------------------------------

27 **N**SD -0.65001372 Non-glycosylated

34 **N**GR -1.1792941 Non-glycosylated

43 **NN**A -1.0165124 Non-glycosylated

44 **N**AD -1.0292767 Non-glycosylated

47 **N**AV -0.87628382 Non-glycosylated

**83 NQS 0.87038926 Potential Glycosylated**

102 **NN**I -0.63750248 Non-glycosylated

103 **N**IF -0.86943732 Non-glycosylated

108 **N**PG -1.5698605 Non-glycosylated

112 **N**TV -1.1712552 Non-glycosylated

132 **N**PA -1.6640458 Non-glycosylated

150 **N**RL -0.97063715 Non-glycosylated

**187 NQT 1.1450942 Potential Glycosylated**

197 **N**TV -0.91796478 Non-glycosylated

**222 NNT 0.70223303 Potential Glycosylated**

223 **N**TG -1.1319446 Non-glycosylated

234 **N**AR -0.79926119 Non-glycosylated

251 **N**SP -0.99344527 Non-glycosylated

**254 NGT 0.86423208 Potential Glycosylated**

270 **N**LF -1.3445899 Non-glycosylated

322 **N**TA -0.89186269 Non-glycosylated

332 **N**RL -0.87170323 Non-glycosylated

354 **N**GD -1.1335154 Non-glycosylated

**376 NGT 1.2874284 Potential Glycosylated**

387 **N**AL -0.69974871 Non-glycosylated

405 **N**K**N** -1.1186197 Non-glycosylated

407 **N**AQ -1.1224605 Non-glycosylated

***********************************

>OAA65787Niveomyces_insect Length = 415

**Potential N-Linked Glycosylated Sites:**

MFSQFLLTSSVFMALTQVATPFPHSS**N**SDKGCQ**N**GRAIYMIS**NN**AD**N**AVVAVPIASDGTLLVDRGTTTATGGVGETGITKAT**N**QSAGPDGLFSQSSLTIA

G**NN**IFAV**N**PGS**N**TVSMFAIDQRDPTKLTLVG**N**PAALLGDFPVTVAASDQ**N**RLVCVGASGARAGVSCASFSAQTGIGAMDGLRSFAL**N**QTTPPEGPL**N**TV

SEVFFSGDESTLFTTVKGDPTV**NN**TGFLASFPVQ**N**ARTTCRAASVGKQATR**N**SP**N**GTAVLFGSKPIPGSS**N**LFVTDASFGAAVLGISSAADIAAGEPGV

VTLGRGVIDGQSATCWATISPAT**N**TAFVTDVGV**N**RLVEMSLTDASIQGQPIDLSA**N**GDPGLIDLVAAGSFIYALSPG**N**GTTEAAVTVL**N**ALTRAQVQHA

QLTSLGL**N**K**N**AQGLAVLV

------------------------------------------------

Position Residue Score Prediction

------------------------------------------------

27 **N**SD -0.65001372 Non-glycosylated

34 **N**GR -1.1792941 Non-glycosylated

43 **NN**A -1.0165124 Non-glycosylated

44 **N**AD -1.0292767 Non-glycosylated

47 **N**AV -0.87628382 Non-glycosylated

**83 NQS 0.87038926 Potential Glycosylated**

102 **NN**I -0.63750248 Non-glycosylated

103 **N**IF -0.86943732 Non-glycosylated

108 **N**PG -1.5698605 Non-glycosylated

112 **N**TV -1.1712552 Non-glycosylated

132 **N**PA -1.6640458 Non-glycosylated

150 **N**RL -0.97063715 Non-glycosylated

**187 NQT 1.1450942 Potential Glycosylated**

197 **N**TV -0.91796478 Non-glycosylated

**222 NNT 0.70223303 Potential Glycosylated**

223 **N**TG -1.1319446 Non-glycosylated

234 **N**AR -0.79926119 Non-glycosylated

251 **N**SP -0.99344527 Non-glycosylated

**254 NGT 0.86423208 Potential Glycosylated**

270 **N**LF -1.3445899 Non-glycosylated

322 **N**TA -0.89186269 Non-glycosylated

332 **N**RL -0.87170323 Non-glycosylated

354 **N**GD -1.1335154 Non-glycosylated

**376 NGT 1.2874284 Potential Glycosylated**

387 **N**AL -0.69974871 Non-glycosylated

405 **N**K**N** -1.1186197 Non-glycosylated

407 **N**AQ -1.1224605 Non-glycosylated

***********************************

>OAA65787Niveomyces_insect Length = 415

**Potential N-Linked Glycosylated Sites:**

MFSQFLLTSSVFMALTQVATPFPHSS**N**SDKGCQ**N**GRAIYMIS**NN**AD**N**AVVAVPIASDGTLLVDRGTTTATGGVGETGITKAT**N**QSAGPDGLFSQSSLTIA

G**NN**IFAV**N**PGS**N**TVSMFAIDQRDPTKLTLVG**N**PAALLGDFPVTVAASDQ**N**RLVCVGASGARAGVSCASFSAQTGIGAMDGLRSFAL**N**QTTPPEGPL**N**TV

SEVFFSGDESTLFTTVKGDPTV**NN**TGFLASFPVQ**N**ARTTCRAASVGKQATR**N**SP**N**GTAVLFGSKPIPGSS**N**LFVTDASFGAAVLGISSAADIAAGEPGV

VTLGRGVIDGQSATCWATISPAT**N**TAFVTDVGV**N**RLVEMSLTDASIQGQPIDLSA**N**GDPGLIDLVAAGSFIYALSPG**N**GTTEAAVTVL**N**ALTRAQVQHA

QLTSLGL**N**K**N**AQGLAVLV

------------------------------------------------

Position Residue Score Prediction

------------------------------------------------

27 **N**SD -0.65001372 Non-glycosylated

34 **N**GR -1.1792941 Non-glycosylated

43 **NN**A -1.0165124 Non-glycosylated

44 **N**AD -1.0292767 Non-glycosylated

47 **N**AV -0.87628382 Non-glycosylated

**83 NQS 0.87038926 Potential Glycosylated**

102 **NN**I -0.63750248 Non-glycosylated

103 **N**IF -0.86943732 Non-glycosylated

108 **N**PG -1.5698605 Non-glycosylated

112 **N**TV -1.1712552 Non-glycosylated

132 **N**PA -1.6640458 Non-glycosylated

150 **N**RL -0.97063715 Non-glycosylated

**187 NQT 1.1450942 Potential Glycosylated**

197 **N**TV -0.91796478 Non-glycosylated

**222 NNT 0.70223303 Potential Glycosylated**

223 **N**TG -1.1319446 Non-glycosylated

234 **N**AR -0.79926119 Non-glycosylated

251 **N**SP -0.99344527 Non-glycosylated

**254 NGT 0.86423208 Potential Glycosylated**

270 **N**LF -1.3445899 Non-glycosylated

322 **N**TA -0.89186269 Non-glycosylated

332 **N**RL -0.87170323 Non-glycosylated

354 **N**GD -1.1335154 Non-glycosylated

**376 NGT 1.2874284 Potential Glycosylated**

387 **N**AL -0.69974871 Non-glycosylated

405 **N**K**N** -1.1186197 Non-glycosylated

407 **N**AQ -1.1224605 Non-glycosylated

***********************************

>XP_045959548Truncatella_a Length = 358

**Potential N-Linked Glycosylated Sites:**

MT**N**EQD**N**SIIALPVQIDGTVSDCGASSTPTDGSGSSGMLAT**N**ETAAPDALFSQSALTTAG**N**HLFAI**N**AGS**N**SVTMFAIDK**N**DPT**N**LTMVGRPVPVPGEFP

**N**TVAASQT**N**KQVCVGMTGAQAGISCASFSSQGIGAMDTLRVFDIGQSTPPVGPE**N**TVSQVFYSGDGSTLFATVKG**N**PAV**N**KTGFLAAFPVQGGIVSQQG

VQSSPDGTAVLFGSTPIPGSS**N**IFVTDASFGAAVLSIDKAGASTVAGKGAVDGQAATCWATISPAT**N**TAFVTDVGK**N**RLVEVSTTSAMVMGQIDLSA**N**G

DPGLIDLKAAGKFIYALSPG**N**GTTQAAMTVV**N**AVSKQQVQHLQLDMLGARKTAQGVAILL

------------------------------------------------

Position Residue Score Prediction

------------------------------------------------

3 **N**EQ -0.64348466 Non-glycosylated

7 **N**SI -0.59781808 Non-glycosylated

**42 NET 0.88102674 Potential Glycosylated**

61 **N**HL -0.67244454 Non-glycosylated

67 **N**AG -1.062141 Non-glycosylated

71 **N**SV -0.68368238 Non-glycosylated

81 **N**DP -0.88155506 Non-glycosylated

**85 NLT 1.0333239 Potential Glycosylated**

101 **N**TV -1.0523166 Non-glycosylated

109 **N**KQ -0.76907402 Non-glycosylated

155 **N**TV -1.0765496 Non-glycosylated

176 **N**PA -1.4723444 Non-glycosylated

**180 NKT 0.78080772 Potential Glycosylated**

221 **N**IF -1.1030747 Non-glycosylated

266 **N**TA -0.93227008 Non-glycosylated

276 **N**RL -0.80984313 Non-glycosylated

297 **N**GD -1.2063029 Non-glycosylated

**319 NGT 1.146959 Potential Glycosylated**

330 **N**AV -1.0473152 Non-glycosylated

***********************************

>KAJ9132390Coniochaeta_hof Length = 400

**Potential N-Linked Glycosylated Sites:**

MHAAFCLLFAGPAVHQVLARPAASCGA**N**K**N**GKAIYMIT**N**DKV**N**AVIAIPIDR**N**GLLQGSGTSTPTGGGGA**N**GIDGST**N**QPAAPDALFSQSSLTLAG**N**SLF

AV**N**PGS**N**TLSMFAIDPKDPTKLVMTGQPAAIPGEFPVTVAASMK**N**KLACVGTTGAKAGISCASFSAKGLGPMDGLREFDIGQTTPPVGPT**N**TVSQVFFS

**N**DGSTLFSTVKGDPAK**NN**TGFLAAFPVQASAG**N**KFAALGQQGVRSSPAGTAVLFGSSTIPGSS**N**LFVTDASFGAAVLSVDGQGVGSVGGKAAVGGQAAT

CWVAVSPAT**N**TAFVTDVGV**N**RLVEMSLVDASIQSVTDLGE**N**GDPGLIDLRAAGEFVYALSPG**N**GTTQAAVTVVDAVSKKQVQHFQLQCLGVGK**N**AQGMA

LLE

------------------------------------------------

Position Residue Score Prediction

------------------------------------------------

28 **N**K**N** -0.92807516 Non-glycosylated

30 **N**GK -0.99536721 Non-glycosylated

39 **N**DK -1.0367841 Non-glycosylated

43 **N**AV -0.98224824 Non-glycosylated

53 **N**GL -0.85802411 Non-glycosylated

71 **N**GI -1.0389134 Non-glycosylated

78 **N**QP -1.2153663 Non-glycosylated

97 **N**SL -0.54584676 Non-glycosylated

103 **N**PG -1.5093776 Non-glycosylated

107 **N**TL -0.77246426 Non-glycosylated

145 **N**KL -1.0491319 Non-glycosylated

191 **N**TV -0.93341528 Non-glycosylated

200 **N**DG -0.94254741 Non-glycosylated

**216 NNT 0.81737572 Potential Glycosylated**

217 **N**TG -1.0314671 Non-glycosylated

232 **N**KF -1.1430697 Non-glycosylated

263 **N**LF -1.3268063 Non-glycosylated

308 **N**TA -0.82637853 Non-glycosylated

318 **N**RL -0.98827243 Non-glycosylated

339 **N**GD -1.1499331 Non-glycosylated

**361 NGT 0.89535851 Potential Glycosylated**

392 **N**AQ -1.2294761 Non-glycosylated

***********************************

>KAI5926236Camillea_tincto Length = 404

**Potential N-Linked Glycosylated Sites:**

MYGLTLLSLSLATIMDQVLARPTSCGMGATQ**N**K**N**GKAIYLIT**N**D**N**T**N**AVVSIPIGEDGLLQTTGTSTATAGAGS**N**SFDGLT**N**QTAAPDALFSQSSLTIAG

**NN**LFAV**N**AGC**N**SLSMFAIDPQDPTKLTMVGKPVAVPGEFPTTVAASAEHQIACVGSTGAKAGVSCASFSAKGLGAMDDLREFDLGQTTPPVGPM**N**TVSQ

VAFS**N**DGKTLFTTVKGDPTK**NN**TGFLASFHVQACTKTKAVSVSQKAVMSSPAGTAVLFGFSTMKGS**NN**LFVTDASFGGAVLSFDQGILATVQGKEVVYG

QTATCWVAISPAT**N**TAFVTDVGV**N**RMVEMSLTDASIQGQVDLSA**N**GDPGLIDLRAAGDFVYALSPG**N**GTTQAAVTVV**N**AMTKEQVQHFQLQDLGVGK**N**A

QGMALLE

------------------------------------------------

Position Residue Score Prediction

------------------------------------------------

32 **N**K**N** -0.87454305 Non-glycosylated

34 **N**GK -0.94426187 Non-glycosylated

43 **N**D**N** -0.98853158 Non-glycosylated

45 **N**T**N** -1.1061957 Non-glycosylated

47 **N**AV -1.1544469 Non-glycosylated

75 **N**SF -0.68554188 Non-glycosylated

**82 NQT 0.91828626 Potential Glycosylated**

101 **NN**L -0.4102008 Non-glycosylated

102 **N**LF -1.038853 Non-glycosylated

107 **N**AG -0.92402752 Non-glycosylated

111 **N**SL -0.85331822 Non-glycosylated

195 **N**TV -1.001504 Non-glycosylated

204 **N**DG -0.90605206 Non-glycosylated

**220 NNT 0.74737876 Potential Glycosylated**

221 **N**TG -1.1459772 Non-glycosylated

266 **NN**L -0.65707978 Non-glycosylated

267 **N**LF -1.2797267 Non-glycosylated

312 **N**TA -0.86395599 Non-glycosylated

322 **N**RM -0.83191386 Non-glycosylated

343 **N**GD -1.1599067 Non-glycosylated

**365 NGT 0.95173133 Potential Glycosylated**

376 **N**AM -0.7584592 Non-glycosylated

396 **N**AQ -1.331451 Non-glycosylated

***********************************

>KAH6877052Thelonectria_ol Length = 401

**Potential N-Linked Glycosylated Sites:**

MHTRIFSFLLSLAPLVLDVSAHPSRCDTGYKSTKAIYMLT**N**DAK**N**AVIALPIGSDGMLSKGTSTEAGGAGS**N**SIMGSTGQPAGPDALVSQSSLTVAG**N**HL

FAV**N**AGS**N**TITMFAIDRHDPTKLTMIGQPVAVPGEFP**N**TVAASKK**N**KLVCAGSTGAKAGVSCSSFSSKGMSAMDELRPFALKQTTPPVGPT**N**TVSQVFF

SADESTLFTTVKGDPTK**NN**TGFLSAFPVRESRRGSAYVSHEGAQSSPEGTAVLFGSSTIPGS**N**DLFVTDASFGAAVLSVDSRTDMATIKGKGAIP**N**QKA

TCWVAVSAATKTAFVTDVAT**N**RLVEMSLTDASIQGTVDLSA**N**GDPGLIDLRAAG**N**LVYALSPG**N**GTTQAAVTVVDAFKKVQVQHFELGSMGAGK**N**AQGM

AVKL

------------------------------------------------

Position Residue Score Prediction

------------------------------------------------

41 **N**DA -1.0157446 Non-glycosylated

45 **N**AV -0.91402604 Non-glycosylated

72 **N**SI -0.79542919 Non-glycosylated

98 **N**HL -0.67816938 Non-glycosylated

104 **N**AG -0.99639877 Non-glycosylated

108 **N**TI -0.68992154 Non-glycosylated

138 **N**TV -1.0516953 Non-glycosylated

146 **N**KL -1.0104156 Non-glycosylated

192 **N**TV -0.78935642 Non-glycosylated

**217 NNT 0.73289828 Potential Glycosylated**

218 **N**TG -1.1994178 Non-glycosylated

262 **N**DL -0.95099544 Non-glycosylated

295 **N**QK -1.1735024 Non-glycosylated

319 **N**RL -0.60149121 Non-glycosylated

340 **N**GD -1.0992751 Non-glycosylated

353 **N**LV -1.1538701 Non-glycosylated

**362 NGT 0.95257204 Potential Glycosylated**

393 **N**AQ -1.1467764 Non-glycosylated

***********************************

>KAH6877052Thelonectria_ol Length = 401

**Potential N-Linked Glycosylated Sites:**

MHTRIFSFLLSLAPLVLDVSAHPSRCDTGYKSTKAIYMLT**N**DAK**N**AVIALPIGSDGMLSKGTSTEAGGAGS**N**SIMGSTGQPAGPDALVSQSSLTVAG**N**HL

FAV**N**AGS**N**TITMFAIDRHDPTKLTMIGQPVAVPGEFP**N**TVAASKK**N**KLVCAGSTGAKAGVSCSSFSSKGMSAMDELRPFALKQTTPPVGPT**N**TVSQVFF

SADESTLFTTVKGDPTK**NN**TGFLSAFPVRESRRGSAYVSHEGAQSSPEGTAVLFGSSTIPGS**N**DLFVTDASFGAAVLSVDSRTDMATIKGKGAIP**N**QKA

TCWVAVSAATKTAFVTDVAT**N**RLVEMSLTDASIQGTVDLSA**N**GDPGLIDLRAAG**N**LVYALSPG**N**GTTQAAVTVVDAFKKVQVQHFELGSMGAGK**N**AQGM

AVKL

------------------------------------------------

Position Residue Score Prediction

------------------------------------------------

41 **N**DA -1.0157446 Non-glycosylated

45 **N**AV -0.91402604 Non-glycosylated

72 **N**SI -0.79542919 Non-glycosylated

98 **N**HL -0.67816938 Non-glycosylated

104 **N**AG -0.99639877 Non-glycosylated

108 **N**TI -0.68992154 Non-glycosylated

138 **N**TV -1.0516953 Non-glycosylated

146 **N**KL -1.0104156 Non-glycosylated

192 **N**TV -0.78935642 Non-glycosylated

**217 NNT 0.73289828 Potential Glycosylated**

218 **N**TG -1.1994178 Non-glycosylated

262 **N**DL -0.95099544 Non-glycosylated

295 **N**QK -1.1735024 Non-glycosylated

319 **N**RL -0.60149121 Non-glycosylated

340 **N**GD -1.0992751 Non-glycosylated

353 **N**LV -1.1538701 Non-glycosylated

**362 NGT 0.95257204 Potential Glycosylated**

393 **N**AQ -1.1467764 Non-glycosylated

***********************************

>KAJ9148577Pleurostoma_ric Length = 409

**Potential N-Linked Glycosylated Sites:**

MFSRLSIFLLALAPILVHVSGRPAACKATPKAAKAVYVMS**N**AQA**N**CVAAIPIGADGMLS**N**GTCTVTGGAGS**N**SIDGTT**N**QPAAPDALVSQSSLTIAG**NN**L

FVV**N**AGS**N**TVSMLSIDSADPTKLTMIGQPVAVPGEFP**N**TVAASKK**NN**LVCVGMTGAKAGVACSSFSAQGIAAMDTLRPI**N**L**N**QTTPPVGPTDTVSQVFF

S**N**DE**N**TLFTTVKGDPTK**NN**TGFLASFPVQRACGS**N**SKGARSAGPASVSQMGVMSSPSGTAVLFGSSTIPGSTDLFVTDASFGGTVLAIDPEADTATLKG

KATVDGQKATCWVTISPAT**N**TAFVTDVSV**N**RLVEMSVS**N**ASIQGQIDLSA**N**GDPGLIDLKAAG**N**FIYALSPG**N**GTTQPAVTVVDAVGKKQLQHFGLDAL

GLDK**N**AQGMAIL

------------------------------------------------

Position Residue Score Prediction

------------------------------------------------

41 **N**AQ -1.0517161 Non-glycosylated

45 **N**CV -0.75210145 Non-glycosylated

**60 NGT 0.99060574 Potential Glycosylated**

72 **N**SI -0.86069677 Non-glycosylated

79 **N**QP -1.1544547 Non-glycosylated

98 **NN**L -0.60166129 Non-glycosylated

99 **N**LF -0.84799687 Non-glycosylated

104 **N**AG -0.99047919 Non-glycosylated

108 **N**TV -0.91810851 Non-glycosylated

138 **N**TV -1.1414638 Non-glycosylated

146 **NN**L -0.87310646 Non-glycosylated

147 **N**LV -1.0730262 Non-glycosylated

180 **N**L**N** -1.1419823 Non-glycosylated

**182 NQT 0.80611793 Potential Glycosylated**

201 **N**DE -0.73503116 Non-glycosylated

204 **N**TL -0.62116936 Non-glycosylated

**217 NNT 0.72642308 Potential Glycosylated**

218 **N**TG -1.1813681 Non-glycosylated

234 **N**SK -1.10423 Non-glycosylated

318 **N**TA -0.89531776 Non-glycosylated

328 **N**RL -0.98937736 Non-glycosylated

**337 NAS 1.2026755 Potential Glycosylated**

349 **N**GD -1.0653485 Non-glycosylated

362 **N**FI -1.1231864 Non-glycosylated

**371 NGT 0.95769094 Potential Glycosylated**

402 **N**AQ -1.1755253 Non-glycosylated

***********************************

>KAJ9148577Pleurostoma_ric Length = 409

**Potential N-Linked Glycosylated Sites:**

MFSRLSIFLLALAPILVHVSGRPAACKATPKAAKAVYVMS**N**AQA**N**CVAAIPIGADGMLS**N**GTCTVTGGAGS**N**SIDGTT**N**QPAAPDALVSQSSLTIAG**NN**L

FVV**N**AGS**N**TVSMLSIDSADPTKLTMIGQPVAVPGEFP**N**TVAASKK**NN**LVCVGMTGAKAGVACSSFSAQGIAAMDTLRPI**N**L**N**QTTPPVGPTDTVSQVFF

S**N**DE**N**TLFTTVKGDPTK**NN**TGFLASFPVQRACGS**N**SKGARSAGPASVSQMGVMSSPSGTAVLFGSSTIPGSTDLFVTDASFGGTVLAIDPEADTATLKG

KATVDGQKATCWVTISPAT**N**TAFVTDVSV**N**RLVEMSVS**N**ASIQGQIDLSA**N**GDPGLIDLKAAG**N**FIYALSPG**N**GTTQPAVTVVDAVGKKQLQHFGLDAL

GLDK**N**AQGMAIL

------------------------------------------------

Position Residue Score Prediction

------------------------------------------------

41 **N**AQ -1.0517161 Non-glycosylated

45 **N**CV -0.75210145 Non-glycosylated

**60 NGT 0.99060574 Potential Glycosylated**

72 **N**SI -0.86069677 Non-glycosylated

79 **N**QP -1.1544547 Non-glycosylated

98 **NN**L -0.60166129 Non-glycosylated

99 **N**LF -0.84799687 Non-glycosylated

104 **N**AG -0.99047919 Non-glycosylated

108 **N**TV -0.91810851 Non-glycosylated

138 **N**TV -1.1414638 Non-glycosylated

146 **NN**L -0.87310646 Non-glycosylated

147 **N**LV -1.0730262 Non-glycosylated

180 **N**L**N** -1.1419823 Non-glycosylated

**182 NQT 0.80611793 Potential Glycosylated**

201 **N**DE -0.73503116 Non-glycosylated

204 **N**TL -0.62116936 Non-glycosylated

**217 NNT 0.72642308 Potential Glycosylated**

218 **N**TG -1.1813681 Non-glycosylated

234 **N**SK -1.10423 Non-glycosylated

318 **N**TA -0.89531776 Non-glycosylated

328 **N**RL -0.98937736 Non-glycosylated

**337 NAS 1.2026755 Potential Glycosylated**

349 **N**GD -1.0653485 Non-glycosylated

362 **N**FI -1.1231864 Non-glycosylated

**371 NGT 0.95769094 Potential Glycosylated**

402 **N**AQ -1.1755253 Non-glycosylated

***********************************

>KAI9158084Paramyrothecium Length = 404

**Potential N-Linked Glycosylated Sites:**

MLHSLLSLVLVVGSSVVLSSPMPCGAPKKAVYIIS**N**EMQ**N**EVVALPIGMDGKLSAGTRTATGGMGS**N**AIDGMTMQPAVPDALVSQSALTVAG**N**SLFAV**N**A

GS**N**TITMF**N**IDEADPTKLTMVGMPVAVPGEFP**N**TVAASESKMLVCVGMTGAMAGVSCASFSPEKGIGMMDALRPFDLKQSTPPVGPT**N**TVSQVFFSDDQ

**N**TLFATVKGDPPK**NN**TGFLASFAVEGDSSCESDMAAVSVQEMQSSPEGTAVLFGSAAIPDT**N**DIFVTDASFGAAVLSIDATTGAAMVKGRGAVEGQVAT

CWAAISPATDTAFVTDVAM**N**RLVEMSVEDASVISSIDLSA**N**GDPGLIDLRASGSFVYALSPG**N**GETEAAVTVVDVMMKKEVQHLRLAEFGLGK**N**AMGMA

VFEMMMM

------------------------------------------------

Position Residue Score Prediction

------------------------------------------------

36 **N**EM -1.0529201 Non-glycosylated

40 **N**EV -0.74254654 Non-glycosylated

67 **N**AI -1.108617 Non-glycosylated

93 **N**SL -0.6523754 Non-glycosylated

99 **N**AG -0.78975801 Non-glycosylated

103 **N**TI -0.67146497 Non-glycosylated

109 **N**ID -1.2119956 Non-glycosylated

133 **N**TV -1.1018056 Non-glycosylated

188 **N**TV -0.8528998 Non-glycosylated

200 **N**TL -0.68388484 Non-glycosylated

**213 NNT 0.81064334 Potential Glycosylated**

214 **N**TG -1.1969138 Non-glycosylated

261 **N**DI -0.98258527 Non-glycosylated

318 **N**RL -0.70513897 Non-glycosylated

339 **N**GD -1.3045474 Non-glycosylated

361 **N**GE -0.81974832 Non-glycosylated

392 **N**AM -1.1530406 Non-glycosylated

***********************************

>KAI9158084Paramyrothecium Length = 404

**Potential N-Linked Glycosylated Sites:**

MLHSLLSLVLVVGSSVVLSSPMPCGAPKKAVYIIS**N**EMQ**N**EVVALPIGMDGKLSAGTRTATGGMGS**N**AIDGMTMQPAVPDALVSQSALTVAG**N**SLFAV**N**A

GS**N**TITMF**N**IDEADPTKLTMVGMPVAVPGEFP**N**TVAASESKMLVCVGMTGAMAGVSCASFSPEKGIGMMDALRPFDLKQSTPPVGPT**N**TVSQVFFSDDQ

**N**TLFATVKGDPPK**NN**TGFLASFAVEGDSSCESDMAAVSVQEMQSSPEGTAVLFGSAAIPDT**N**DIFVTDASFGAAVLSIDATTGAAMVKGRGAVEGQVAT

CWAAISPATDTAFVTDVAM**N**RLVEMSVEDASVISSIDLSA**N**GDPGLIDLRASGSFVYALSPG**N**GETEAAVTVVDVMMKKEVQHLRLAEFGLGK**N**AMGMA

VFEMMMM

------------------------------------------------

Position Residue Score Prediction

------------------------------------------------

36 **N**EM -1.0529201 Non-glycosylated

40 **N**EV -0.74254654 Non-glycosylated

67 **N**AI -1.108617 Non-glycosylated

93 **N**SL -0.6523754 Non-glycosylated

99 **N**AG -0.78975801 Non-glycosylated

103 **N**TI -0.67146497 Non-glycosylated

109 **N**ID -1.2119956 Non-glycosylated

133 **N**TV -1.1018056 Non-glycosylated

188 **N**TV -0.8528998 Non-glycosylated

200 **N**TL -0.68388484 Non-glycosylated

**213 NNT 0.81064334 Potential Glycosylated**

214 **N**TG -1.1969138 Non-glycosylated

261 **N**DI -0.98258527 Non-glycosylated

318 **N**RL -0.70513897 Non-glycosylated

339 **N**GD -1.3045474 Non-glycosylated

361 **N**GE -0.81974832 Non-glycosylated

392 **N**AM -1.1530406 Non-glycosylated

***********************************

>KAG7131979Verticillium_lo Length = 406

**Potential N-Linked Glycosylated Sites:**

MHFQTSHLILALAPSIVGVSARPSGSSSCMAASQVEKAVYLLS**N**EVK**N**SVIALPIGVDGLLKVGQQTATGGAGS**N**SVDGSTRQPAAPDALVSQSALTVAG

HSLFAV**N**AGS**N**TITMLLIDKKDPTKLQVVGEPVAVPGEFP**N**TVAASSK**N**SIVCVGTSGAVAGVACAPFTK**N**GIGAMD**N**LRPFDLKQTTPPVGPT**N**TVSQ

VFFSDDQ**N**TLFTTVKGDPTK**NN**TGFLSSFAVQAS**N**AGGCAASVSQEDRRTSPEGTAVLFGSSAIPGTTDIFVTDASFGGAVLSVDAKSG**N**ATLKGKGLV

DGQAATCWATVSPATGTAFVTDVGR**N**RLVEMSTADA**N**IISEIDLSA**N**GDPGLIDLRASGQFIYALSPG**N**GTTQAAVTVVDAKRKKQVQHFQLG**N**MGAGK

**N**AMGMAVLT

------------------------------------------------

Position Residue Score Prediction

------------------------------------------------

44 **N**EV -1.1031316 Non-glycosylated

48 **N**SV -0.5977332 Non-glycosylated

75 **N**SV -0.87836329 Non-glycosylated

107 **N**AG -1.0544174 Non-glycosylated

111 **N**TI -0.68434982 Non-glycosylated

141 **N**TV -1.1360759 Non-glycosylated

149 **N**SI -1.0468666 Non-glycosylated

171 **N**GI -0.93992514 Non-glycosylated

178 **N**LR -1.0393126 Non-glycosylated

195 **N**TV -0.85407078 Non-glycosylated

207 **N**TL -0.67381783 Non-glycosylated

**220 NNT 0.71230349 Potential Glycosylated**

221 **N**TG -1.2351912 Non-glycosylated

234 **N**AG -1.2467745 Non-glycosylated

**289 NAT 1.2929027 Potential Glycosylated**

324 **N**RL -0.91946854 Non-glycosylated

335 **N**II -0.90540287 Non-glycosylated

345 **N**GD -1.4272302 Non-glycosylated

**367 NGT 1.0567846 Potential Glycosylated**

392 **N**MG -1.3426462 Non-glycosylated

398 **N**AM -1.0823906 Non-glycosylated

***********************************

>XP_003007212Verticillium_ Length = 406

**Potential N-Linked Glycosylated Sites:**

MHFKISHLILALTHSIVGASAQPSGSSSCKAASPADKAVYLLS**N**EVK**N**SVIALPIGVDGLLQAGQQTATGGAGS**N**AVDSSTGQPAAPDALVSQSALTVAG

HSLFAV**N**AGS**N**TITMFRIDKK**N**STKLQVVGRPVAVPGEFP**N**TVAASSK**N**GIVCVGTSGAVAGVSCAPFTK**N**GIGAMD**N**LRPFDLKQTTPPVGPT**N**TVSQ

VFFSDDQ**N**TLFTTVKGDPTK**NN**TGFLSSFAVQASDEGECAASVSQE**N**RRTSPEGTSVLFGSSVIPGTTDIFVTDASFGGAVLSVDA**NN**G**N**TTVKGKGPV

DGQAATCWATVSPATGTAFVTDVGR**N**RLVEMSTADAEIISEIDLSS**N**GDPGLIDLRASGQFIYALSPG**N**GTTQAAVTVVDAKSKKQVQHLQLG**N**MGAGK

**N**AMGMAVLA

------------------------------------------------

Position Residue Score Prediction

------------------------------------------------

44 **N**EV -1.1097516 Non-glycosylated

48 **N**SV -0.5977332 Non-glycosylated

75 **N**AV -0.87569667 Non-glycosylated

107 **N**AG -1.0647862 Non-glycosylated

111 **N**TI -0.67754833 Non-glycosylated

**122 NST 0.78632454 Potential Glycosylated**

141 **N**TV -1.0465541 Non-glycosylated

149 **N**GI -1.0732029 Non-glycosylated

171 **N**GI -0.95046636 Non-glycosylated

178 **N**LR -1.0393126 Non-glycosylated

195 **N**TV -0.85407078 Non-glycosylated

207 **N**TL -0.67381783 Non-glycosylated

**220 NNT 0.71230349 Potential Glycosylated**

221 **N**TG -1.2351912 Non-glycosylated

246 **N**RR -0.92807383 Non-glycosylated

286 **NN**G -0.95597327 Non-glycosylated

287 **N**G**N** -0.90144465 Non-glycosylated

**289 NTT 1.3705097 Potential Glycosylated**

324 **N**RL -0.91946854 Non-glycosylated

345 **N**GD -1.2639937 Non-glycosylated

**367 NGT 1.0567846 Potential Glycosylated**

392 **N**MG -1.4929137 Non-glycosylated

398 **N**AM -1.1814177 Non-glycosylated

***********************************

>XP_008099976Colletotrichu Length = 370

**Potential N-Linked Glycosylated Sites:**

MKVGKTIYTIT**N**EVE**N**SVVALPISRTGLL**N**SG**N**SVKTGGAGS**N**FFDSAT**N**GPAAPDALASQSALTIAG**N**SLFAV**N**AGS**N**TLSMFAI**N**QSDPTRLTMVGQP

VAIPAQFP**N**TVAASDK**N**KIVCVGSSGAVSGISCAGFSETGVGSMDILRPVEL**N**QTTPPAGPT**N**TISHAFFS**N**DE**N**TLFATVKGDPAK**NN**TGFLAAYRVQ

F**N**EGCKSVSHYGVRSSP**N**GTAVLFGSSTIP**N**S**N**DLFVTDASFGGVILSLDAQSKAAVKGSAAVDGQKATCWATVSPAT**N**TAFVTDVAS**N**RLVEMSLK**N**A

SVMSVIDLSA**N**GDPGLTDLKAAGRFVYALSPG**N**GTTQAAITVVDAVGKKQVQRFELQQFGVGK**N**AQGMAVLL

------------------------------------------------

Position Residue Score Prediction

------------------------------------------------

12 **N**EV -1.2255848 Non-glycosylated

16 **N**SV -0.77432579 Non-glycosylated

30 **N**SG -1.4368872 Non-glycosylated

33 **N**SV -0.66332682 Non-glycosylated

43 **N**FF -0.92152801 Non-glycosylated

50 **N**GP -1.1774273 Non-glycosylated

69 **N**SL -0.58993147 Non-glycosylated

75 **N**AG -0.82882885 Non-glycosylated

79 **N**TL -0.74517637 Non-glycosylated

**87 NQS 1.2690764 Potential Glycosylated**

109 **N**TV -0.94275844 Non-glycosylated

117 **N**KI -1.1431329 Non-glycosylated

**153 NQT 0.89351519 Potential Glycosylated**

163 **N**TI -0.87980049 Non-glycosylated

172 **N**DE -0.78951389 Non-glycosylated

175 **N**TL -0.80124508 Non-glycosylated

**188 NNT 0.92373767 Potential Glycosylated**

189 **N**TG -1.0498284 Non-glycosylated

201 **N**EG -1.0756057 Non-glycosylated

**217 NGT 0.8713785 Potential Glycosylated**

230 **N**S**N** -0.66155296 Non-glycosylated

232 **N**DL -1.0048247 Non-glycosylated

278 **N**TA -1.0102426 Non-glycosylated

288 **N**RL -0.78190458 Non-glycosylated

**297 NAS 0.99364626 Potential Glycosylated**

309 **N**GD -1.1094742 Non-glycosylated

**331 NGT 0.93454764 Potential Glycosylated**

362 **N**AQ -1.4222357 Non-glycosylated

***********************************

>KAK2032527Colletotrichum_ Length = 370

**Potential N-Linked Glycosylated Sites:**

MKVGKTIYTIT**N**DAV**N**SVVALPISQSGLLAGGSSIKTGGAGS**N**FFDSAT**N**GPAAPDALASQSALTIAG**N**SLFAV**N**AGS**N**TLTMFAIDKSDPTKLTMVGKP

VAVPAQFP**N**TVAAS**N**K**N**KLVCVGSSGAVSGISCASFSAAGVGAMDMLRPVEL**N**QTTPPAGPT**N**TISHAFFSDDE**N**TLFATVKGDPTK**NN**TGFFSAYRVK

A**N**KDCKSVSHHDVRSSP**N**GTAVLFGSSTIP**N**T**N**DLFVTDASFGGAILSLDAQ**NN**ATVKGSAAVEGQKATCWVTVSPAT**N**TAFVTDVGS**N**RLVEMSLKDA

SIM**N**IVDLSA**N**GDPGLTDLKAAGRFIYALSPG**N**GTTQPAITVVDAVGKKQVQHFELQKFGVGK**N**AQGMAVLL

------------------------------------------------

Position Residue Score Prediction

------------------------------------------------

12 **N**DA -1.1777879 Non-glycosylated

16 **N**SV -0.95573288 Non-glycosylated

43 **N**FF -0.91628711 Non-glycosylated

50 **N**GP -1.1774273 Non-glycosylated

69 **N**SL -0.58993147 Non-glycosylated

75 **N**AG -0.89856752 Non-glycosylated

79 **N**TL -0.52924586 Non-glycosylated

109 **N**TV -0.92464361 Non-glycosylated

115 **N**K**N** -0.72062349 Non-glycosylated

117 **N**KL -1.1651639 Non-glycosylated

**153 NQT 0.78238643 Potential Glycosylated**

163 **N**TI -0.85187445 Non-glycosylated

175 **N**TL -0.87436149 Non-glycosylated

**188 NNT 0.96572021 Potential Glycosylated**

189 **N**TG -1.2218458 Non-glycosylated

201 **N**KD -0.99069656 Non-glycosylated

**217 NGT 0.68571441 Potential Glycosylated**

230 **N**T**N** -0.67499803 Non-glycosylated

232 **N**DL -1.0855502 Non-glycosylated

252 **NN**A -0.84354341 Non-glycosylated

**253 NAT 1.1762171 Potential Glycosylated**

278 **N**TA -0.96723898 Non-glycosylated

288 **N**RL -0.86420419 Non-glycosylated

302 **N**IV -1.4803002 Non-glycosylated

309 **N**GD -1.1584607 Non-glycosylated

**331 NGT 0.96598528 Potential Glycosylated**

362 **N**AQ -1.4545061 Non-glycosylated

***********************************

>KAK2040685Colletotrichum_ Length = 370

**Potential N-Linked Glycosylated Sites:**

MKVGKTIYTIT**N**DAV**N**SVVALPIS**N**TGLLLGGSSIKTGGAGS**N**FFDSAT**N**GPAAPDALASQSALTIAG**N**SLFAV**N**AGS**N**TLTMFTIDQSDPTKLTMVGKP

VAVPAQFP**N**TVAAS**N**K**N**KLVCVGSSGAVSGISCASFSAAGVDAMDMLRPVKL**N**QTTPPAGPT**N**TISHAFFSDDE**N**TLFATVKGDPTK**NN**TGFFSAYRVQ

A**N**RDCKSVSHHDVRSSP**N**GTAVLFGSSTIP**N**T**NN**LFVTDASFGGAILSLDAQ**NN**ATVKGSAAVEGQKATCWVTVSPAT**N**TAFVTDVGS**N**RLVEISLKDA

SVM**N**IVDLSA**N**GDPGLTDLKAAGRFIYALSPG**N**GTTQPAITVVDAVGKKQVQHFELQKFGLGK**N**AQGMAVLM

------------------------------------------------

Position Residue Score Prediction

------------------------------------------------

12 **N**DA -1.1777879 Non-glycosylated

16 **N**SV -0.92683776 Non-glycosylated

25 **N**TG -1.1535474 Non-glycosylated

43 **N**FF -0.91628711 Non-glycosylated

50 **N**GP -1.1774273 Non-glycosylated

69 **N**SL -0.58993147 Non-glycosylated

75 **N**AG -0.87450808 Non-glycosylated

79 **N**TL -0.49669434 Non-glycosylated

109 **N**TV -0.92464361 Non-glycosylated

115 **N**K**N** -0.72062349 Non-glycosylated

117 **N**KL -1.1651639 Non-glycosylated

**153 NQT 0.76418413 Potential Glycosylated**

163 **N**TI -0.85187445 Non-glycosylated

175 **N**TL -0.87436149 Non-glycosylated

**188 NNT 0.96572021 Potential Glycosylated**

189 **N**TG -1.2577539 Non-glycosylated

201 **N**RD -0.90803251 Non-glycosylated

**217 NGT 0.68571441 Potential Glycosylated**

230 **N**T**N** -0.67541893 Non-glycosylated

232 **NN**L -1.0002236 Non-glycosylated

233 **N**LF -1.4316254 Non-glycosylated

252 **NN**A -0.84354341 Non-glycosylated

**253 NAT 1.1762171 Potential Glycosylated**

278 **N**TA -0.96723898 Non-glycosylated

288 **N**RL -0.90064728 Non-glycosylated

302 **N**IV -1.2830581 Non-glycosylated

309 **N**GD -1.0981807 Non-glycosylated

**331 NGT 0.96598528 Potential Glycosylated**

362 **N**AQ -1.3436131 Non-glycosylated

***********************************

>KDN65785Colletotrichum_su Length = 397

**Potential N-Linked Glycosylated Sites:**

MRFSTTFLVLTSWSTAITAKPTGCGAAKK**N**GMTLYTTT**N**DKV**N**SVVALPISG**N**GRLTGATSFKTGGAGS**N**FFDPAA**N**ASAAPDALASQSALTVAG**NN**LFA

V**N**AGS**N**TLTMFAIDEADPTKLTMVGKPVAVPAEFP**N**TVAASDK**N**KLVCVGSSGAVSGISCASFSGAGIAAMDKLRPIEL**N**QTTPPAGPT**N**TISQAFFS**N**

DE**N**TLFTTVKGDPTK**NN**TGFLAAYKVQAAKGCKSVSQDDVRSSPDGTAVLFGSSTIP**N**S**N**DLFVTDASFGAAVLSLDGG**NN**AAVKGKAAVDGQRATCWA

TVSSAT**N**SAFVTDVGS**N**RLVEMSLK**N**ASIM**N**IVDLSA**N**GDPGLTDLKAAGKFVYALSPG**N**GTTPAAITVVDAAAKKQVQHFELDKFGVGK**N**AQGMAVLQ

------------------------------------------------

Position Residue Score Prediction

------------------------------------------------

30 **N**GM -0.73446976 Non-glycosylated

39 **N**DK -1.0720495 Non-glycosylated

43 **N**SV -1.0450352 Non-glycosylated

53 **N**GR -0.91518768 Non-glycosylated

70 **N**FF -1.1826768 Non-glycosylated

**77 NAS 0.88973487 Potential Glycosylated**

96 **NN**L -0.54277698 Non-glycosylated

97 **N**LF -0.81501348 Non-glycosylated

102 **N**AG -0.85509154 Non-glycosylated

106 **N**TL -0.62513784 Non-glycosylated

136 **N**TV -0.93526732 Non-glycosylated

144 **N**KL -1.0773617 Non-glycosylated

**180 NQT 0.77691548 Potential Glycosylated**

190 **N**TI -0.8944828 Non-glycosylated

199 **N**DE -0.7024845 Non-glycosylated

202 **N**TL -0.56535924 Non-glycosylated

**215 NNT 0.82166147 Potential Glycosylated**

216 **N**TG -1.2559659 Non-glycosylated

257 **N**S**N** -0.66155296 Non-glycosylated

259 **N**DL -1.0048247 Non-glycosylated

279 **NN**A -1.1629963 Non-glycosylated

280 **N**AA -0.9521699 Non-glycosylated

305 **N**SA -0.89998388 Non-glycosylated

315 **N**RL -0.87100003 Non-glycosylated

**324 NAS 0.91905774 Potential Glycosylated**

329 **N**IV -1.4801026 Non-glycosylated

336 **N**GD -1.1584607 Non-glycosylated

**358 NGT 1.1413277 Potential Glycosylated**

389 **N**AQ -1.3553991 Non-glycosylated

***********************************

>KAK1994904Colletotrichum_ Length = 397

**Potential N-Linked Glycosylated Sites:**

MRFLTAVSVLLSWSSTIKAKPVKC**NN**TKKVGRAIYTIT**N**DAS**N**AVVVVPIVRTGLLSGGTSIKTGGTGS**N**FFD**N**AA**N**GPAAPDALASQSALTIAG**N**SLFA

V**N**AGS**N**TLTMFSIDQFDPTKLEMVGQPTAVPAEFP**N**TVAASDK**N**KLVCVGSSGAVSGISCAPFSGAGIGTMDKLRPVGL**N**QTTPPAGPV**N**TISQAFFS**N**

DE**N**TLFATVKGDPAK**N**KTGFLAAYKVQSDKGCKSVSQHYVRSSP**N**GTAVLFGSSTIP**N**T**N**SLFVTDASFGGAVLALDAQ**NN**AVVKGAGIV**N**GQGATCWA

TVSPAT**N**TAFVTDVAS**N**RLVEMSLKDASIMGILDLSA**N**GDPGLTDLKAAGRFIYALSPG**N**GTSQAAITVVDAVEKRQVQHFGLQQLGAGK**N**AQGMAVSL

------------------------------------------------

Position Residue Score Prediction

------------------------------------------------

**25 NNT 0.96908089 Potential Glycosylated**

26 **N**TK -1.0236657 Non-glycosylated

39 **N**DA -1.0342419 Non-glycosylated

43 **N**AV -0.7619235 Non-glycosylated

70 **N**FF -1.2726784 Non-glycosylated

74 **N**AA -1.1112263 Non-glycosylated

77 **N**GP -1.0564664 Non-glycosylated

96 **N**SL -0.58993147 Non-glycosylated

102 **N**AG -0.94897089 Non-glycosylated

106 **N**TL -0.60989225 Non-glycosylated

136 **N**TV -1.0320238 Non-glycosylated

144 **N**KL -1.0773617 Non-glycosylated

**180 NQT 0.74619801 Potential Glycosylated**

190 **N**TI -0.94220186 Non-glycosylated

199 **N**DE -0.7248635 Non-glycosylated

202 **N**TL -0.57208312 Non-glycosylated

**215 NKT 0.8910155 Potential Glycosylated**

**244 NGT 0.95355463 Potential Glycosylated**

257 **N**T**N** -0.7118711 Non-glycosylated

259 **N**SL -1.0588846 Non-glycosylated

279 **NN**A -1.1276747 Non-glycosylated

280 **N**AV -0.85741433 Non-glycosylated

290 **N**GQ -1.1510388 Non-glycosylated

305 **N**TA -1.0102426 Non-glycosylated

315 **N**RL -0.7164868 Non-glycosylated

336 **N**GD -1.077948 Non-glycosylated

**358 NGT 0.99316928 Potential Glycosylated**

389 **N**AQ -1.3446784 Non-glycosylated

***********************************

>XP_060406759Colletotrichu Length = 397

**Potential N-Linked Glycosylated Sites:**

MRFSVAILIFASWP**N**AITATPVKCK**N**AQKVGKAIYTIT**N**DAS**N**AVVAVPISP**N**GLLSGGTSVKTGGAGS**N**FFDGA**NN**GPAAPDALASQSALTVAG**NN**LFA

V**N**AGS**N**TLTMFAIDKADPTKLTMVGKPVAVPAEFP**N**TVAAS**N**K**N**RIVCVGSSGAVAGISCASFSGAGISAMDELRPIDLKQTTPPAGPT**N**TISQAFFS**N**

DERTLFATVKGDPTK**NN**TGFLASYQVQA**N**KGAKSVSQQDARSSPQGTAVLFGSSTIPGSKDLFVTDASFGAAVLSLDAK**NN**AAVKGKGAVDGQKATCWA

TVSPAT**N**TAFVTDVGT**N**RLVEMSV**N**DASIKGVLDLSK**N**GDPGLTDLKAAGRFVYALSPG**N**GTT**N**AAITVVDAVEKKQVQHFDLSKLGAGK**N**TQGMAVML

------------------------------------------------

Position Residue Score Prediction

------------------------------------------------

15 **N**AI -1.0605892 Non-glycosylated

26 **N**AQ -1.0594836 Non-glycosylated

39 **N**DA -1.1136446 Non-glycosylated

43 **N**AV -0.727403 Non-glycosylated

53 **N**GL -0.90771837 Non-glycosylated

70 **N**FF -0.98041262 Non-glycosylated

76 **NN**G -1.1568659 Non-glycosylated

77 **N**GP -1.0890387 Non-glycosylated

96 **NN**L -0.54277698 Non-glycosylated

97 **N**LF -0.81501348 Non-glycosylated

102 **N**AG -0.85509154 Non-glycosylated

106 **N**TL -0.57036631 Non-glycosylated

136 **N**TV -0.87728236 Non-glycosylated

142 **N**K**N** -0.80983035 Non-glycosylated

144 **N**RI -1.2285376 Non-glycosylated

190 **N**TI -0.82788353 Non-glycosylated

199 **N**DE -0.76541181 Non-glycosylated

**215 NNT 0.83312382 Potential Glycosylated**

216 **N**TG -1.2266448 Non-glycosylated

228 **N**KG -1.2079257 Non-glycosylated

279 **NN**A -0.995545 Non-glycosylated

280 **N**AA -0.78027195 Non-glycosylated

305 **N**TA -0.79248763 Non-glycosylated

315 **N**RL -0.98753509 Non-glycosylated

323 **N**DA -1.288746 Non-glycosylated

336 **N**GD -1.0287095 Non-glycosylated

**358 NGT 0.98624958 Potential Glycosylated**

362 **N**AA -0.89310365 Non-glycosylated

389 **N**TQ -1.2358531 Non-glycosylated

***********************************

>KZL66164Colletotrichum_to Length = 399

**Potential N-Linked Glycosylated Sites:**

MAPVLRLLFLLVTSSVAVSARPARCHGT**N**RVGRVIYTIT**N**DKA**N**SVVAVPISS**N**GMLSGGTSISTGGAGS**N**FFDGTT**N**GPAAPDALASQSALTIAGT**N**LF

AV**N**AGS**N**TMTMFAIDQSDPTKLTMVGQPVAVPGEFP**N**TVAASQK**NN**LVCVGSSGAKSGISCAKFSE**N**GVGAMDMLRSVDL**N**QTTPPAGPT**N**TISQAFFS

**N**DESTLLATVKGDPDKMKTGFLAAYQVESGGGGTSVSQSGMRSSP**N**GTAVLFGSSTIP**N**T**NN**IFATDASFGGAVLSLDKQT**N**VASVKGMAAVDGQKATC

WVAISPAT**N**TAFVTDVGT**N**RLVEMSLTDASIKSTLDLTA**N**GDPGLTDLKAAGKFVYALSPG**N**GTTQPAITVVDAVGKKQVQHFGLQQLGVGK**N**AQGMAL

LL

------------------------------------------------

Position Residue Score Prediction

------------------------------------------------

29 **N**RV -0.82104071 Non-glycosylated

40 **N**DK -1.0164861 Non-glycosylated

44 **N**SV -0.94672005 Non-glycosylated

54 **N**GM -0.87805753 Non-glycosylated

71 **N**FF -0.98721208 Non-glycosylated

78 **N**GP -1.2157662 Non-glycosylated

98 **N**LF -0.80367686 Non-glycosylated

103 **N**AG -1.115981 Non-glycosylated

107 **N**TM -0.6755122 Non-glycosylated

137 **N**TV -1.1144365 Non-glycosylated

145 **NN**L -0.96803452 Non-glycosylated

146 **N**LV -1.0231597 Non-glycosylated

167 **N**GV -1.0117712 Non-glycosylated

**181 NQT 0.81217503 Potential Glycosylated**

191 **N**TI -0.8944828 Non-glycosylated

200 **N**DE -0.65660576 Non-glycosylated

**245 NGT 1.0224789 Potential Glycosylated**

258 **N**T**N** -0.77703521 Non-glycosylated

260 **NN**I -1.0474354 Non-glycosylated

261 **N**IF -1.4789206 Non-glycosylated

281 **N**VA -1.1028238 Non-glycosylated

307 **N**TA -0.79686035 Non-glycosylated

317 **N**RL -0.83207098 Non-glycosylated

338 **N**GD -1.0745077 Non-glycosylated

**360 NGT 0.97629564 Potential Glycosylated**

391 **N**AQ -1.4227198 Non-glycosylated

***********************************

>GJC89458Colletotrichum_li Length = 399

**Potential N-Linked Glycosylated Sites:**

MAPVLRLLFLLVTSSVAVSARPAKCHAT**N**RVGRVIYTIT**N**DKA**N**SVVAVPISS**N**GMLSGGTSISTGGAGS**N**FFDGTT**N**GPAAPDALASQSALTIAGT**N**LF

AV**N**AGS**N**TMTMFAIDQSDPTKLTMVGQPVAVPGEFP**N**TVAASQK**NN**LVCVGSSGAKSGISCAKFSD**N**GVGAMDMLRSVDL**N**QTTPPAGPT**N**TISQAFFS

**N**DESTLLATVKGDP**N**KMKTGFLAAYQVEAGGGGTSVSQSGMRSSP**N**GTAVLFGSSTIP**N**T**N**DIFATDASFGGAVLSLDKQT**N**VASVKGMAAVDGQKATC

WVAISPAT**N**TAFVTDVGT**N**RLVEMSLTDASIKSTLDLSA**N**GDPGLTDLKAAGKFVYALSPG**N**GTTQPAITVVDAVGKKQVQHFGLQQLGVGK**N**AQGMAL

LL

------------------------------------------------

Position Residue Score Prediction

------------------------------------------------

29 **N**RV -0.57488169 Non-glycosylated

40 **N**DK -1.0164861 Non-glycosylated

44 **N**SV -0.94672005 Non-glycosylated

54 **N**GM -0.87805753 Non-glycosylated

71 **N**FF -0.98721208 Non-glycosylated

78 **N**GP -1.2157662 Non-glycosylated

98 **N**LF -0.80367686 Non-glycosylated

103 **N**AG -1.115981 Non-glycosylated

107 **N**TM -0.6755122 Non-glycosylated

137 **N**TV -1.1144365 Non-glycosylated

145 **NN**L -0.96803452 Non-glycosylated

146 **N**LV -1.0231597 Non-glycosylated

167 **N**GV -1.0752243 Non-glycosylated

**181 NQT 0.81217503 Potential Glycosylated**

191 **N**TI -0.8944828 Non-glycosylated

200 **N**DE -0.65660576 Non-glycosylated

214 **N**KM -0.90765882 Non-glycosylated

**245 NGT 1.0224789 Potential Glycosylated**

258 **N**T**N** -0.77536462 Non-glycosylated

260 **N**DI -1.1092872 Non-glycosylated

281 **N**VA -1.1028238 Non-glycosylated

307 **N**TA -0.79686035 Non-glycosylated

317 **N**RL -0.83207098 Non-glycosylated

338 **N**GD -1.0423696 Non-glycosylated

**360 NGT 0.97629564 Potential Glycosylated**

391 **N**AQ -1.4227198 Non-glycosylated

***********************************

>KAK1978899Colletotrichum_ Length = 405

**Potential N-Linked Glycosylated Sites:**

MRPSTSLLICLQALSAPITARPARCGSGKQVGAA**N**KVGKVIYTTT**N**DKD**N**AVVAIPISS**N**GMLSKG**N**KIATGGAGS**N**FFDPAAKGPAAPDALASQSALTV

AGK**N**LFAV**N**AGS**N**TVTMFAI**N**QADPTKLTMVGQPVAVGGEFP**N**TVAASAK**N**KLVCVGSSGAKSGISCASFSA**N**GIGAMDGLR**N**VDL**N**QTTPPAGPT**N**TI

SQVFFS**N**DEKTLFTTVKGDPSIRKAGFFAAYQVQAG**N**AGASVCQEGVQSSP**N**GTAVLFGSSTIP**N**S**N**DIFATDASFGGAVLSVDPKT**N**VASIKGMAAVD

GQKATCWVAVSPAT**N**TAFVTDVGA**N**RLVEMSLKDASIK**N**TIDLSA**NN**DPGLTDLKAAGQFVYALSPG**N**GTTPAAITVVDAVKK**N**QVQHFDLQQLGAGK**N**

AQGMAVLL

------------------------------------------------

Position Residue Score Prediction

------------------------------------------------

35 **N**KV -0.99622023 Non-glycosylated

46 **N**DK -0.93771565 Non-glycosylated

50 **N**AV -1.1523959 Non-glycosylated

60 **N**GM -0.90332195 Non-glycosylated

67 **N**KI -0.90704345 Non-glycosylated

77 **N**FF -1.0327799 Non-glycosylated

104 **N**LF -0.87230361 Non-glycosylated

109 **N**AG -1.0085748 Non-glycosylated

113 **N**TV -0.90888982 Non-glycosylated

121 **N**QA -0.8421434 Non-glycosylated

143 **N**TV -1.1419745 Non-glycosylated

151 **N**KL -1.0288536 Non-glycosylated

173 **N**GI -0.88583408 Non-glycosylated

183 **N**VD -1.0798312 Non-glycosylated

**187 NQT 0.92730721 Potential Glycosylated**

197 **N**TI -0.89445798 Non-glycosylated

206 **N**DE -0.68038232 Non-glycosylated

236 **N**AG -1.0807188 Non-glycosylated

**251 NGT 0.96697474 Potential Glycosylated**

264 **N**S**N** -0.76744431 Non-glycosylated

266 **N**DI -0.98373713 Non-glycosylated

287 **N**VA -1.2270002 Non-glycosylated

313 **N**TA -0.8615295 Non-glycosylated

323 **N**RL -0.95533368 Non-glycosylated

337 **N**TI -1.3909479 Non-glycosylated

344 **NN**D -0.97186728 Non-glycosylated

345 **N**DP -0.85238226 Non-glycosylated

**366 NGT 1.0846247 Potential Glycosylated**

382 **N**QV -1.2619285 Non-glycosylated

397 **N**AQ -1.2226079 Non-glycosylated

***********************************

>XP_018163326Colletotrichu Length = 399

**Potential N-Linked Glycosylated Sites:**

MHLCSALLAYLVTIHVSAAAFPARCKSTTQFGKAIYTIT**N**E**N**A**N**AVVAVPISRTGMLSKGTSIMTGGMGS**N**FIDGIT**N**KPAAPDALASQSALTVAG**NN**LF

AV**N**AGS**N**TITMFEIDQS**N**PTKLTMVGQPIAVPGQFP**N**TVAASQK**N**GIVCVGSSGAVSGISCAKFSA**N**GVAAMDTLRSIEL**N**QTTPPAGPT**N**TISHAFFS

**N**DEKTLFVTVKGDPEKKKTGFIAAYQVESGSGSPLVCEQGVRSSP**N**GTAVLFGSSTIP**N**TTDLFTTDASFGAAVLSIEAST**N**AATVKGMAAVDGQKATC

WVAVSPAT**N**TAFVTDVGT**N**RLVEMSVTDASIKSVIDLGA**NN**DPGLIDLKASGKFIYALSPG**N**GTTQPAITVV**N**AVSKKQVQHFPLEQLGVGK**N**AQGMAL

LV

------------------------------------------------

Position Residue Score Prediction

------------------------------------------------

40 **N**E**N** -1.2623165 Non-glycosylated

42 **N**A**N** -1.0290222 Non-glycosylated

44 **N**AV -0.8972045 Non-glycosylated

71 **N**FI -1.2658499 Non-glycosylated

78 **N**KP -1.3165751 Non-glycosylated

97 **NN**L -0.54277698 Non-glycosylated

98 **N**LF -0.81501348 Non-glycosylated

103 **N**AG -0.82545527 Non-glycosylated

107 **N**TI -0.72458366 Non-glycosylated

**118 NPT 0.22741438 Potential Glycosylated**

137 **N**TV -1.1640778 Non-glycosylated

145 **N**GI -1.0696811 Non-glycosylated

167 **N**GV -1.1138902 Non-glycosylated

**181 NQT 0.93659355 Potential Glycosylated**

191 **N**TI -0.87980049 Non-glycosylated

200 **N**DE -0.78221175 Non-glycosylated

**245 NGT 0.74852828 Potential Glycosylated**

**258 NTT 1.3378587 Potential Glycosylated**

281 **N**AA -0.99022147 Non-glycosylated

307 **N**TA -0.76526123 Non-glycosylated

317 **N**RL -0.82104062 Non-glycosylated

338 **NN**D -1.1728766 Non-glycosylated

339 **N**DP -1.1212101 Non-glycosylated

**360 NGT 1.0769286 Potential Glycosylated**

371 **N**AV -1.0652438 Non-glycosylated

391 **N**AQ -1.399927 Non-glycosylated

***********************************

>KXH68255Colletotrichum_sa Length = 396

**Potential N-Linked Glycosylated Sites:**

MRQHIVLSLCFALSFAIVSGRTVGCKA**N**SQTGKAIYITT**N**DQA**N**SVVAIPISG**N**GMLSKGAVFPTGGSGS**N**VV**N**GTSKQPAAPDALASQSALTIAG**NN**LF

VV**N**AGS**N**TVTMFAIDPSDPTKLTMIGQPVAVPGQFP**N**TIAASMKH**N**VICVGCSGAQSGVSCATFSKDGLECMDSLRPVGLSQTTPPAGPT**N**TISQVFFS

**N**DQGTLFTTVKGDPSQ**N**KTGFLGAYKVSSKGSVEH**N**GVQSSP**N**GTAVLFGSSTIPKS**NN**LFVTDPSFGGAVLSVDAKS**N**AASVKGMGVISGQKATCWAA

VSSATGSAFVADVGT**N**RLIEMSLSDASIKSILDLSP**N**GDPGLTDLMAAGQFVYALSPG**N**GTTQSAITVVDAVAKKQVQHFVVGQLGVGR**N**AQGMAAML

------------------------------------------------

Position Residue Score Prediction

------------------------------------------------

28 **N**SQ -0.6282579 Non-glycosylated

40 **N**DQ -1.0216909 Non-glycosylated

44 **N**SV -0.96742651 Non-glycosylated

54 **N**GM -1.013978 Non-glycosylated

71 **N**VV -0.91650754 Non-glycosylated

**74 NGT 1.1261958 Potential Glycosylated**

97 **NN**L -0.58245899 Non-glycosylated

98 **N**LF -0.82846716 Non-glycosylated

103 **N**AG -1.1108075 Non-glycosylated

107 **N**TV -0.73574019 Non-glycosylated

137 **N**TI -1.0552206 Non-glycosylated

146 **N**VI -0.91803688 Non-glycosylated

191 **N**TI -0.88859306 Non-glycosylated

200 **N**DQ -0.59191498 Non-glycosylated

**216 NKT 0.85016844 Potential Glycosylated**

235 **N**GV -0.77825133 Non-glycosylated

**242 NGT 0.76386655 Potential Glycosylated**

257 **NN**L -0.93943828 Non-glycosylated

258 **N**LF -1.2752344 Non-glycosylated

278 **N**AA -1.0413415 Non-glycosylated

314 **N**RL -0.84346735 Non-glycosylated

335 **N**GD -1.0805853 Non-glycosylated

**357 NGT 0.98725261 Potential Glycosylated**

388 **N**AQ -1.2539273 Non-glycosylated

***********************************

>XP_060439001Colletotrichu Length = 396

**Potential N-Linked Glycosylated Sites:**

MRQHIVLSLCFASSFAIVSGRTVGCKA**N**SQTGKAIYTIT**N**DQV**N**SVVAIPISG**N**GMLSKGAVFPTGGSGS**N**FLDGTS**N**KPAAPDALPSQSALTIAG**NN**LF

AV**N**AGS**N**TVTMFSIDPLDPTKLTIVGQPVAVPGQFP**N**TIAASIKH**N**VVCVGCSGAQSGVSCTTFSKDGLESMDSLRPVGLGQTTPPAGPT**N**TISQVFFS

**N**DQGTLFTTVKGDPSQ**N**RTGLLGAYKVSSKGSVQQ**N**GVQSSP**N**GTAVLFGSSTIPKS**NN**LFVTDASFGGAVLSVDPKS**N**AASVKGMGVVSGQKATCWVA

VSLATGSAFVTDVGT**N**RLIEMSLSDASIKSILDLSP**N**GDPGLTDLRAAGQFVYALSPG**N**GTTQSTITVVDAVAKKQVQHFVLGQLGVGR**N**AQGMAVML

------------------------------------------------

Position Residue Score Prediction

------------------------------------------------

28 **N**SQ -0.683527 Non-glycosylated

40 **N**DQ -0.99466108 Non-glycosylated

44 **N**SV -0.97485761 Non-glycosylated

54 **N**GM -1.013978 Non-glycosylated

71 **N**FL -0.96383356 Non-glycosylated

78 **N**KP -1.1336313 Non-glycosylated

97 **NN**L -0.5859518 Non-glycosylated

98 **N**LF -0.84308842 Non-glycosylated

103 **N**AG -1.0278821 Non-glycosylated

107 **N**TV -0.81624269 Non-glycosylated

137 **N**TI -1.0204174 Non-glycosylated

146 **N**VV -0.92705209 Non-glycosylated

191 **N**TI -0.91118083 Non-glycosylated

200 **N**DQ -0.59191498 Non-glycosylated

**216 NRT 0.92620788 Potential Glycosylated**

235 **N**GV -0.75802719 Non-glycosylated

**242 NGT 1.0796191 Potential Glycosylated**

257 **NN**L -0.86368847 Non-glycosylated

258 **N**LF -1.398149 Non-glycosylated

278 **N**AA -1.181843 Non-glycosylated

314 **N**RL -0.92712797 Non-glycosylated

335 **N**GD -1.0506657 Non-glycosylated

**357 NGT 1.0984424 Potential Glycosylated**

388 **N**AQ -1.3554636 Non-glycosylated

***********************************

>XP_060430279Colletotrichu Length = 375

**Potential N-Linked Glycosylated Sites:**

MVGCKAKPQTGKAIYTIT**N**DQA**N**SVVAIPISD**N**GMLSKGAVFPTGGSGS**N**FLDGTS**N**KPAAPDALASQSALAVVG**NN**LFAV**N**AGS**N**TVTMFSIDPSDPTK

LTMIGQPVAVPGQFP**N**TIAASMKH**N**VVCVGCSGAQSGVSCATFSKDGLESMDSLRPVELSQTTPPAGPT**N**TISQVFFS**N**DQGTLFTTVKGDPSQ**N**KTGF

LGAYRVS**N**KGSVQQYRVQSSP**N**GTAVLFGSSTIPKS**NN**LFVTDASFGGAILYVDPKSDAASVKGMGVVGGQKATCWVAVSSATGSAFVTDVGT**N**RLIEM

SL**N**DASIKSILDLSS**N**GDPGLTDLKAAGQFVYALSPG**N**GTTQSAVTVVDAIAKKQLQHFVLGRLGVGR**N**AQGMTVML

------------------------------------------------

Position Residue Score Prediction

------------------------------------------------

19 **N**DQ -1.0606011 Non-glycosylated

23 **N**SV -0.89249116 Non-glycosylated

33 **N**GM -1.1054661 Non-glycosylated

50 **N**FL -0.96383356 Non-glycosylated

57 **N**KP -1.13119 Non-glycosylated

76 **NN**L -0.57191851 Non-glycosylated

77 **N**LF -0.94140273 Non-glycosylated

82 **N**AG -0.92830095 Non-glycosylated

86 **N**TV -0.76873243 Non-glycosylated

116 **N**TI -1.0552206 Non-glycosylated

125 **N**VV -0.97198865 Non-glycosylated

170 **N**TI -0.88859306 Non-glycosylated

179 **N**DQ -0.59191498 Non-glycosylated

**195 NKT 0.82310436 Potential Glycosylated**

207 **N**KG -1.0125557 Non-glycosylated

**221 NGT 0.9565765 Potential Glycosylated**

236 **NN**L -0.86368847 Non-glycosylated

237 **N**LF -1.398149 Non-glycosylated

293 **N**RL -0.98363591 Non-glycosylated

301 **N**DA -1.2696387 Non-glycosylated

314 **N**GD -0.93548409 Non-glycosylated

**336 NGT 0.94584336 Potential Glycosylated**

367 **N**AQ -1.3517312 Non-glycosylated

***********************************

>XP_036576679Colletotrichu Length = 400

**Potential N-Linked Glycosylated Sites:**

MHLIFLVFVCYLQTLLPVVVACPTPG**N**KKIQPGKAVYFIT**N**EKD**N**AVVAMKIAE**N**GMLT**N**GATASTGGAGS**N**FIDGDTKQPAAPDALASQSAVTIAG**N**YL

FAV**N**AGS**N**SLTMFAI**N**SADPTKLAMVGQPVAVPGEFP**N**TVAASKK**N**KLVCVGSSGAKAGVSCASFSAKGVSTMDGLRPVDLKQTTPPAGPT**N**TISQAFF

SGDESALLLTVKGDPTKM**N**TGFLGVYQVEQA**N**GVASLSQ**N**EIRSSP**N**GTAVLFGSSTIP**N**SKDIFVTDASFGGAVLSMDPKT**N**QATVKGMAKVDGQKAT

CWVAISSAT**N**SAFVTDVGR**N**IMVEMSLKDASIKGKIDLGA**N**GDPGLIDLKAAGKFIYALSPG**N**GTTQAAITVVDAMEKKQVQHL**N**VQGLGAGK**N**SQGMA

VLL

------------------------------------------------

Position Residue Score Prediction

------------------------------------------------

27 **N**KK -1.0433072 Non-glycosylated

41 **N**EK -1.1573119 Non-glycosylated

45 **N**AV -0.99849376 Non-glycosylated

55 **N**GM -0.62540098 Non-glycosylated

60 **N**GA -0.91369719 Non-glycosylated

72 **N**FI -1.1043722 Non-glycosylated

98 **N**YL -0.76193684 Non-glycosylated

104 **N**AG -0.91487537 Non-glycosylated

108 **N**SL -0.89099457 Non-glycosylated

116 **N**SA -0.89996354 Non-glycosylated

138 **N**TV -1.0516953 Non-glycosylated

146 **N**KL -1.1506488 Non-glycosylated

192 **N**TI -0.83014805 Non-glycosylated

218 **N**TG -1.2145413 Non-glycosylated

231 **N**GV -1.3946407 Non-glycosylated

239 **N**EI -0.76604863 Non-glycosylated

**246 NGT 1.0265952 Potential Glycosylated**

259 **N**SK -0.77704382 Non-glycosylated

282 **N**QA -1.0760129 Non-glycosylated

308 **N**SA -0.75507897 Non-glycosylated

318 **N**IM -0.78989705 Non-glycosylated

339 **N**GD -1.2506818 Non-glycosylated

**361 NGT 1.1476872 Potential Glycosylated**

383 **N**VQ -1.2548954 Non-glycosylated

392 **N**SQ -1.0883649 Non-glycosylated

***********************************

>XP_060282260Phialemonium_ Length = 409

**Potential N-Linked Glycosylated Sites:**

MYAHVFTTSLLFLALIVPEASACRTGVRP**N**PATGKAIYFIT**N**DAV**N**AVVALPIGADGLLSAAGTFTATGGAGA**N**SIDGAT**N**QPAAPDALVSQSALTIAGS

**N**LFAV**N**AGS**N**TVSMFAI**N**PADPTRLSMVGQPVAVLGEFP**N**TVAASDK**N**RLVCVGSSGAKAGISCTSFSARGMAAMDGLRVIDLQQTTPPVGPT**N**TVSQV

FFS**N**DE**N**TLFSTVKGDPAQ**NN**TGFLASFQVQAKA**N**GLRFGAAAAAVSAQGTASSPDGTAVLFGSSTIPGSTDLFVTDASFGAAVLSVDLATGAATVKGK

TALDGQKATCWAAVSLAT**N**TAFVTDVGV**N**RVVEMSLADASVVATVDLSA**N**GDPGLIDLKVAG**N**FLYALSPGDGTTQPAVAVLDVISKQQVQHAELQALG

VGK**N**AQGMAVLL

------------------------------------------------

Position Residue Score Prediction

------------------------------------------------

30 **N**PA -1.8115469 Non-glycosylated

42 **N**DA -1.1127791 Non-glycosylated

46 **N**AV -0.73751816 Non-glycosylated

74 **N**SI -0.96516003 Non-glycosylated

81 **N**QP -1.0747356 Non-glycosylated

101 **N**LF -0.75874608 Non-glycosylated

106 **N**AG -1.0240843 Non-glycosylated

110 **N**TV -0.90832051 Non-glycosylated

118 **N**PA -1.323946 Non-glycosylated

140 **N**TV -1.0065515 Non-glycosylated

148 **N**RL -1.077075 Non-glycosylated

194 **N**TV -0.89842091 Non-glycosylated

203 **N**DE -0.83123256 Non-glycosylated

206 **N**TL -0.68400256 Non-glycosylated

**219 NNT 0.83724371 Potential Glycosylated**

220 **N**TG -0.93120196 Non-glycosylated

234 **N**GL -0.98902248 Non-glycosylated

317 **N**TA -0.74168196 Non-glycosylated

327 **N**RV -1.0563045 Non-glycosylated

348 **N**GD -1.1307666 Non-glycosylated

361 **N**FL -1.1006993 Non-glycosylated

401 **N**AQ -1.4454934 Non-glycosylated

***********************************

>XP_030989089Thyridium_cur Length = 422

**Potential N-Linked Glycosylated Sites:**

MHLTRSFPFLLSLGLVPTALARP**N**ARAEAGRS**N**GRAIYILS**N**EQQ**N**SVVALPMAG**N**GMLSAGS**N**TPTGGAGS**N**AIDGMTKQPAGPDALLSQSALTVAG**N**S

LFAV**N**AGS**N**TVTMFAIDRADPTKLKMVG**N**PMSVKGEFP**N**TVAASEKHKIVCVASTGAVAGVSCGSFSSCGIGAMDDLRPIDLKQTTPPVGPT**N**TVSQVF

FS**N**DQDTLYVTVKGDPSK**N**STGFLGAFPVQGKSACMAKASVSREGVMSSPEGTVVLFGSSPIPKSTDLFVTDASFGAVVLAIDPKTEVATAKGKGVIEG

QKATCWSTISPAS**N**TAFVTDVAL**N**RLVEMSLTDARIVSIIDLAA**N**GDPGLIDLEAAGKFVYALSPG**N**GTTPAAVTVVDVTSKAQVQHLQLKDAGLSK**N**A

QGMALLFERSGDRTD**N**LGDMCRDTR

------------------------------------------------

Position Residue Score Prediction

------------------------------------------------

24 **N**AR -1.1119601 Non-glycosylated

33 **N**GR -1.1718648 Non-glycosylated

42 **N**EQ -0.73708749 Non-glycosylated

46 **N**SV -0.63726056 Non-glycosylated

56 **N**GM -0.95811885 Non-glycosylated

64 **N**TP -1.0092986 Non-glycosylated

73 **N**AI -0.76964103 Non-glycosylated

99 **N**SL -0.54806489 Non-glycosylated

105 **N**AG -0.87860475 Non-glycosylated

109 **N**TV -0.68119717 Non-glycosylated

129 **N**PM -1.3402041 Non-glycosylated

139 **N**TV -0.95403829 Non-glycosylated

193 **N**TV -0.86769492 Non-glycosylated

202 **N**DQ -0.64212416 Non-glycosylated

**218 NST 0.72678433 Potential Glycosylated**

312 **N**TA -0.87979419 Non-glycosylated

322 **N**RL -0.75077759 Non-glycosylated

343 **N**GD -1.1944433 Non-glycosylated

**365 NGT 1.1134497 Potential Glycosylated**

396 **N**AQ -1.0100119 Non-glycosylated

413 **N**LG -1.2706252 Non-glycosylated

***********************************

>XP_009227230Gaeumannomyce Length = 413

**Potential N-Linked Glycosylated Sites:**

MHVSALLLLALGQAS**N**VLALPA**NNNN**SGCGAPSQPGAAAR**N**AKAVYTIT**N**EKE**N**AVVAIRVGQDGMLQAQGSSSTPTGGAGATGVDGDGKPAVPDALFSQ

SALTVAGS**N**LFAV**N**AGS**N**TLTMFAIDAADPTKLTMVGKPVAIPGEFPVTVAASKR**N**KLACVATTGAKAGVSCASFSAQAGLAAMDALRPFDLGQTTPPK

GPT**N**TVSQVFFARDGKTLFATVKGDPPA**N**KTGFLASFPVQAAAQ**N**KAASL**N**AQGAQSSPQGTAVLFGSATIPGSRELFVTDASFGAAVLSVDAATGAAT

VKGKGAVDGQKATCWATI**N**PATGTAFVTDVAT**N**RLVEMSVKDASVMGQIDLSA**N**GDPGLIDLQSAGKFVYALSPG**N**GTTQAAITVVDATTKKQVQHMQL

QAMGVGK**N**SMGLALRK

------------------------------------------------

Position Residue Score Prediction

------------------------------------------------

16 **N**VL -0.90977134 Non-glycosylated

23 **NNN** -0.74989909 Non-glycosylated

24 **NNN** -0.93185765 Non-glycosylated

**25 NNS 1.0470103 Potential Glycosylated**

26 **N**SG -0.91286769 Non-glycosylated

41 **N**AK -0.860162 Non-glycosylated

50 **N**EK -1.1144375 Non-glycosylated

54 **N**AV -0.81563468 Non-glycosylated

109 **N**LF -0.73908074 Non-glycosylated

114 **N**AG -1.0032601 Non-glycosylated

118 **N**TL -0.53239762 Non-glycosylated

156 **N**KL -1.0700324 Non-glycosylated

203 **N**TV -0.81904918 Non-glycosylated

**228 NKT 0.73256674 Potential Glycosylated**

244 **N**KA -0.9861811 Non-glycosylated

250 **N**AQ -0.87480328 Non-glycosylated

317 **N**PA -1.4878553 Non-glycosylated

331 **N**RL -0.77885224 Non-glycosylated

352 **N**GD -1.2687714 Non-glycosylated

**374 NGT 1.0418792 Potential Glycosylated**

405 **N**SM -1.2589779 Non-glycosylated

***********************************

>KLU91551Magnaporthiopsis_ Length = 384

**Potential N-Linked Glycosylated Sites:**

MHASALLLLSLGQVASVLARPAT**NN**KSCAK**N**ARVVYTI**NN**EKE**N**AVVAIRVGADGMLQAAGASKTATGGAGA**N**GVDDKGQPAAPDPLFSQSALTVAGK**N**L

FAV**N**AGS**N**TLTMFAIDATDPTKLTMVGKPVAIPGEFPVTVAAS**N**K**N**KLACVGTTGAKAGVSCASFSAQGLAAMDALRPFDLGQTTPPKGPT**N**TVSQVFF

ARDGKTLFTTVKGDPPAKK**N**GFLASFSSSPQGTAVLFGSTTIPGSKDLFVTDASFGAAVLSVDAAGG**N**ATVKGKGTVDGQKATCWVTI**N**PTTKTAFVTD

VGT**N**RLVEMSTQDAKILGQVDLSA**N**GDPGLIDLQAAGKFVYALSPG**N**GTTQAAITVVDATTKKQVQHMQLQAMGVGK**N**SMGLALLK

------------------------------------------------

Position Residue Score Prediction

------------------------------------------------

24 **NN**K -0.77669441 Non-glycosylated

**25 NKS 1.0911539 Potential Glycosylated**

31 **N**AR -0.901176 Non-glycosylated

39 **NN**E -0.76058395 Non-glycosylated

40 **N**EK -0.97477292 Non-glycosylated

44 **N**AV -0.78197199 Non-glycosylated

73 **N**GV -1.0833783 Non-glycosylated

99 **N**LF -0.87230361 Non-glycosylated

104 **N**AG -0.99801908 Non-glycosylated

108 **N**TL -0.50360819 Non-glycosylated

144 **N**K**N** -0.56422638 Non-glycosylated

146 **N**KL -1.0381242 Non-glycosylated

192 **N**TV -0.81904918 Non-glycosylated

219 **N**GF -0.90955208 Non-glycosylated

**267 NAT 1.4692178 Potential Glycosylated**

**288 NPT 0.40470612 Potential Glycosylated**

302 **N**RL -0.80791885 Non-glycosylated

323 **N**GD -1.2522494 Non-glycosylated

**345 NGT 1.0418792 Potential Glycosylated**

376 **N**SM -1.3145439 Non-glycosylated

***********************************

>KLU91551Magnaporthiopsis_ Length = 384

**Potential N-Linked Glycosylated Sites:**

MHASALLLLSLGQVASVLARPAT**NN**KSCAK**N**ARVVYTI**NN**EKE**N**AVVAIRVGADGMLQAAGASKTATGGAGA**N**GVDDKGQPAAPDPLFSQSALTVAGK**N**L

FAV**N**AGS**N**TLTMFAIDATDPTKLTMVGKPVAIPGEFPVTVAAS**N**K**N**KLACVGTTGAKAGVSCASFSAQGLAAMDALRPFDLGQTTPPKGPT**N**TVSQVFF

ARDGKTLFTTVKGDPPAKK**N**GFLASFSSSPQGTAVLFGSTTIPGSKDLFVTDASFGAAVLSVDAAGG**N**ATVKGKGTVDGQKATCWVTI**N**PTTKTAFVTD

VGT**N**RLVEMSTQDAKILGQVDLSA**N**GDPGLIDLQAAGKFVYALSPG**N**GTTQAAITVVDATTKKQVQHMQLQAMGVGK**N**SMGLALLK

------------------------------------------------

Position Residue Score Prediction

------------------------------------------------

24 **NN**K -0.77669441 Non-glycosylated

**25 NKS 1.0911539 Potential Glycosylated**

31 **N**AR -0.901176 Non-glycosylated

39 **NN**E -0.76058395 Non-glycosylated

40 **N**EK -0.97477292 Non-glycosylated

44 **N**AV -0.78197199 Non-glycosylated

73 **N**GV -1.0833783 Non-glycosylated

99 **N**LF -0.87230361 Non-glycosylated

104 **N**AG -0.99801908 Non-glycosylated

108 **N**TL -0.50360819 Non-glycosylated

144 **N**K**N** -0.56422638 Non-glycosylated

146 **N**KL -1.0381242 Non-glycosylated

192 **N**TV -0.81904918 Non-glycosylated

219 **N**GF -0.90955208 Non-glycosylated

**267 NAT 1.4692178 Potential Glycosylated**

**288 NPT 0.40470612 Potential Glycosylated**

302 **N**RL -0.80791885 Non-glycosylated

323 **N**GD -1.2522494 Non-glycosylated

**345 NGT 1.0418792 Potential Glycosylated**

376 **N**SM -1.3145439 Non-glycosylated

***********************************

>KAH8841654Pyricularia_ory Length = 404

**Potential N-Linked Glycosylated Sites:**

MRASTTLLLALVAQAIASPCPKGCGK**N**KAKAAK**N**AKAIYVLS**N**EKA**N**AVVAMPIGKDGMVGKPVSTATGGEGATGVDDKGQPAVPDALFSQSAVTVAGK**N**

LFAV**N**PGS**N**TLTMFAIDPKDPTKLTMVGKPAAIPGEFPVTVGASSK**N**KMACVGTSGAKAGISCASFSDKGLGKMDALRPIDLGQTTPPKGPT**N**TVSQVF

FSRDQKTLFSTVKGDPPT**N**KDGFIASFPVAAAAAAGQAAKVDSKM**N**KATPDGTKVLFGATTIPGSKDLFVTDAAFGAVVLAMDQKTQSASTVKGKGEVD

GQKATCWATVSPATGTAFVTDVGS**N**RLVEMSTKDAKVL**N**TVELT**N**GDPGLIDLQAAGSFVYALSPG**N**GTTPAAVTVVDAKSRKQVQHVQMDAKLAGK**N**S

MGLAMLK

------------------------------------------------

Position Residue Score Prediction

------------------------------------------------

27 **N**KA -1.1698367 Non-glycosylated

34 **N**AK -0.80296845 Non-glycosylated

43 **N**EK -1.0897692 Non-glycosylated

47 **N**AV -0.82908293 Non-glycosylated

100 **N**LF -0.85287122 Non-glycosylated

105 **N**PG -1.689744 Non-glycosylated

109 **N**TL -0.74782444 Non-glycosylated

147 **N**KM -1.0972592 Non-glycosylated

193 **N**TV -0.83670315 Non-glycosylated

218 **N**KD -1.3076009 Non-glycosylated

245 **N**KA -1.0165162 Non-glycosylated

323 **N**RL -0.88065152 Non-glycosylated

337 **N**TV -1.0616727 Non-glycosylated

343 **N**GD -1.1283801 Non-glycosylated

**365 NGT 1.1488858 Potential Glycosylated**

396 **N**SM -1.2024176 Non-glycosylated

***********************************

>OIW33070Coniochaeta_ligni Length = 394

**Potential N-Linked Glycosylated Sites:**

MRAETLASVVLAALVLPVSALPQTTRSSSGKAIYIIT**N**DAK**N**AVVAVRIGRDGQLSRGTTTETGGAGGSGVDSAT**N**QTAAPDSLFSQSSLT**N**IFAV**N**PGS

**N**TVSMLSIDPRDPTKLSLVGTPATLPGEFPVTVAASVK**N**RLVCVGMTGAKAGVSCASFSA**N**GLGAMDGLRTFDLGQTTPPAGPL**N**TVSQVLFSSDESVL

FATVKGDPTV**NN**TGFLAAFPI**N**KACSGKGASLAQQGTKSSP**N**GTAVLFGSSIIPGSS**N**LFVTDASFGGAVLAIDGQDVASTVGKGVIDGQGATCWATVS

PAT**N**TAFVSDVAV**N**RLVQMSLTDATPLGQIDLSS**N**GDPGLIDLRAAG**N**FVYALSPG**N**GTTSPAVTVVDAIGARQVQHFQLQGLGVSKSAMGMAVLV

------------------------------------------------

Position Residue Score Prediction

------------------------------------------------

38 **N**DA -0.89723528 Non-glycosylated

42 **N**AV -0.71259312 Non-glycosylated

**76 NQT 0.89050884 Potential Glycosylated**

92 **N**IF -1.1883523 Non-glycosylated

97 **N**PG -1.6281545 Non-glycosylated

101 **N**TV -1.0711628 Non-glycosylated

139 **N**RL -0.79823267 Non-glycosylated

161 **N**GL -0.78253298 Non-glycosylated

185 **N**TV -0.98016589 Non-glycosylated

**210 NNT 0.72619355 Potential Glycosylated**

211 **N**TG -1.0361968 Non-glycosylated

221 **N**KA -1.1265489 Non-glycosylated

**241 NGT 1.004214 Potential Glycosylated**

257 **N**LF -1.3232481 Non-glycosylated

302 **N**TA -0.94963563 Non-glycosylated

312 **N**RL -0.78827873 Non-glycosylated

333 **N**GD -1.0992855 Non-glycosylated

346 **N**FV -1.2136071 Non-glycosylated

**355 NGT 0.88460538 Potential Glycosylated**

***********************************

>XP_014174329Grosmannia_cl Length = 394

**Potential N-Linked Glycosylated Sites:**

MRYAPALPLLLAPLVLQVLARPAAKGIGAVYLIT**N**EKD**N**AVLAMPIQRDGLLSAGKFVSTGGSGA**N**GIDGST**N**KSAAPDALFSQSSLTVSGQ**N**LFAV**N**AG

S**N**TVSMFAIDARDPTKLKMVGKPAAVPGEFPSTVASSAKH**N**LVCVGTTGAKAGVSCATFSAKGLGTMDALRPFKLGQSTPPVGPT**N**TVSQVFFSDDGAT

LFSTVKGDPTK**NN**TGFLAAFSVTAGK**N**SSAAAVSKQGKMTSPPGTAVLFGSSTIPGSKSLFVTDASFGAAVLSVSKAGSVSMAGKGVVAGQKATCWAAV

S**N**ATKTAFVTDVAT**N**RLVEMSLT**N**AKVISEI**N**LK**N**GDPGLIDLKAAG**N**FIYALSPG**N**GTIPAAVTVVSAL**N**KTQVQHFRLRHQVGK**N**AQGMAVMLS

------------------------------------------------

Position Residue Score Prediction

------------------------------------------------

35 **N**EK -1.2026398 Non-glycosylated

39 **N**AV -0.83929 Non-glycosylated

66 **N**GI -1.202185 Non-glycosylated

**73 NKS 0.95228775 Potential Glycosylated**

93 **N**LF -1.0341101 Non-glycosylated

98 **N**AG -0.95985185 Non-glycosylated

102 **N**TV -0.85397696 Non-glycosylated

141 **N**LV -0.92013356 Non-glycosylated

186 **N**TV -0.93856276 Non-glycosylated

**211 NNT 0.62117371 Potential Glycosylated**

212 **N**TG -1.1085525 Non-glycosylated

**226 NSS 0.96424016 Potential Glycosylated**

**300 NAT 1.2559864 Potential Glycosylated**

313 **N**RL -0.66580651 Non-glycosylated

322 **N**AK -0.88955535 Non-glycosylated

330 **N**LK -1.2634167 Non-glycosylated

333 **N**GD -1.1430762 Non-glycosylated

346 **N**FI -1.1231864 Non-glycosylated

**355 NGT 1.0323184 Potential Glycosylated**

**369 NKT 0.96401721 Potential Glycosylated**

**385** **N**AQ -1.4599989 Non-glycosylated

***********************************

>KAH8770811Diaporthaceae_s Length = 420

**Potential N-Linked Glycosylated Sites:**

MYFSTLTFLFAVAPSAIQVSARPAKSCGSKGGAAKVA**N**GKAVYMLS**N**QAS**N**AVVAVPIAQDGTL**N**EAGGSST**N**TGGSGAAGIDGST**N**APS**N**PDALFSQSA

LTVAG**NN**LFAV**N**AGS**N**TLSMFAIDAQDPTKLTMVGQPAQLPGEFPVTVGASAKH**N**VACVGMTGSTAGISCASFDGKQGLGAMDDLRPFDLGQSTPPVGP

T**N**TVSQVFFSDDQATMFATVKGDPTK**NN**TGFLASFPV**N**AAGGGCQAASVGAQGEQTSPEGTAVLFGSAAIPGSSDLFVTDASFGGAVLSTQQASAGGSS

QASGAAQVVGKGVVDGQKATCWAAISPAT**N**TAFVTDVGV**N**HLVEMSLKDASIQSQTDLSA**N**GDPGLIDLKAAG**N**MVYALSPG**N**GTT**N**AAVTVMDATSKK

QVQHLDMKALGLDG**N**AMGMAVLE

------------------------------------------------

Position Residue Score Prediction

------------------------------------------------

38 **N**GK -1.0196401 Non-glycosylated

47 **N**QA -1.2807797 Non-glycosylated

51 **N**AV -0.65173931 Non-glycosylated

65 **N**EA -1.1389741 Non-glycosylated

73 **N**TG -1.2988835 Non-glycosylated

87 **N**AP -1.1115153 Non-glycosylated

91 **N**PD -1.6410707 Non-glycosylated

106 **NN**L -0.54434225 Non-glycosylated

107 **N**LF -0.81501348 Non-glycosylated

112 **N**AG -0.79500126 Non-glycosylated

116 **N**TL -0.63319559 Non-glycosylated

155 **N**VA -0.87674161 Non-glycosylated

201 **N**TV -0.93856276 Non-glycosylated

**226 NNT 0.78360316 Potential Glycosylated**

227 **N**TG -1.1142199 Non-glycosylated

237 **N**AA -0.94287228 Non-glycosylated

328 **N**TA -0.88408248 Non-glycosylated

338 **N**HL -0.93478845 Non-glycosylated

359 **N**GD -1.1537116 Non-glycosylated

372 **N**MV -1.1592829 Non-glycosylated

**381 NGT 0.91571808 Potential Glycosylated**

**385** **N**AA -0.98713449 Non-glycosylated

412 **N**AM -1.373735 Non-glycosylated

***********************************

>POS79352Diaporthe_heliant Length = 424

**Potential N-Linked Glycosylated Sites:**

MYISTFVSLLALASSAIQVSARPARSCGSKGCAAKVT**N**GKAVYMLS**N**QAS**N**TVVAVPIAQDGTL**N**EAGGSSTTTGGAGATGIDGST**N**APS**N**PDALFSQSA

LTVAG**NN**LFAV**N**AGS**N**TLSMFTIDAQDPTKLTMVGQPAQLPGEFPVTVGASAKH**N**IACAGMTGSTAGISCASF**N**AEQGLGAMDDLRPFDLGQSTPPVGP

T**N**TVSQVFFSDDQATMFATVKGDPTK**NN**TGFLASFPV**N**AASRCQSASVAAQGEQSSPEGTAVLFGSAAIPGTSDLFVTDASFGGAVLSMQQAAAAATG**N**

GSSQAAGAAPQVVGKGVVEGQKATCWAAISPAT**N**TAFVTDVGV**N**HLVEMSLEDASIQSQTDLSA**N**GDPGLIDLKAAG**N**MVYALSPG**N**GTT**N**AAVTVVDA

TSKKQVQHLDMKALGFDA**N**AMGMAVLE

------------------------------------------------

Position Residue Score Prediction

------------------------------------------------

38 **N**GK -1.0162533 Non-glycosylated

47 **N**QA -1.202658 Non-glycosylated

51 **N**TV -0.66063264 Non-glycosylated

65 **N**EA -1.0389364 Non-glycosylated

87 **N**AP -1.0404753 Non-glycosylated

91 **N**PD -1.6410707 Non-glycosylated

106 **NN**L -0.54434225 Non-glycosylated

107 **N**LF -0.81501348 Non-glycosylated

112 **N**AG -0.79992271 Non-glycosylated

116 **N**TL -0.58164945 Non-glycosylated

155 **N**IA -0.81876382 Non-glycosylated

174 **N**AE -1.0015998 Non-glycosylated

201 **N**TV -0.93856276 Non-glycosylated

**226 NNT 0.78360316 Potential Glycosylated**

227 **N**TG -1.1142199 Non-glycosylated

237 **N**AA -1.0689713 Non-glycosylated

**298 NGS 1.3151107 Potential Glycosylated**

332 **N**TA -0.88408248 Non-glycosylated

342 **N**HL -0.91444838 Non-glycosylated

363 **N**GD -1.1537116 Non-glycosylated

376 **N**MV -1.1592829 Non-glycosylated

**385 NGT 0.92103107 Potential Glycosylated**

389 **N**AA -0.98055998 Non-glycosylated

416 **N**AM -1.3751118 Non-glycosylated

***********************************

>XP_043018833Diaporthe_cit Length = 402

**Potential N-Linked Glycosylated Sites:**

MQVAARPAKPCGSKGGAAKAA**N**GKAIYMLS**N**QAS**N**SVVAVPIAEDGTL**N**EAGGSST**N**TGGSGA**N**GVDGST**N**QPAMPDPLFSQSALTVAG**NN**LFAV**N**AGS**N**

TLSMFAIDAQDPTKLTMVG**N**PADLPGEFPVTVGASAKH**N**LACVGMSGSTAGVSCASFDAAQGLGAMDALRPFDLGQTTPPVGPT**N**TVSHVFFSDDQSTL

FSTVKGDPTK**NN**TGFLASFPV**N**AAGQCQAASVGAEGAQDSP**N**GTAVLFGSSPIAGSSDLFVTDASFGAAVLSTQQAAGGSAQASGAAEVVGKGAIDGQK

ATCWVAISPATKTAFVTDVGV**N**HLVEMSLADASILGETDLSA**N**GDPGLIDLKAAG**N**MVYALSPG**N**GTT**N**AAVTVMDA**N**TKQQVQHLDMTSLGLDA**N**AQG

MAVLE

------------------------------------------------

Position Residue Score Prediction

------------------------------------------------

22 **N**GK -0.89397931 Non-glycosylated

31 **N**QA -1.2489954 Non-glycosylated

35 **N**SV -0.6909026 Non-glycosylated

49 **N**EA -1.1586541 Non-glycosylated

57 **N**TG -1.2256923 Non-glycosylated

64 **N**GV -0.99888187 Non-glycosylated

71 **N**QP -1.323051 Non-glycosylated

90 **NN**L -0.54434225 Non-glycosylated

91 **N**LF -0.81501348 Non-glycosylated

96 **N**AG -0.79500126 Non-glycosylated

100 **N**TL -0.63319559 Non-glycosylated

120 **N**PA -1.7178078 Non-glycosylated

139 **N**LA -0.99863356 Non-glycosylated

185 **N**TV -0.91087519 Non-glycosylated

**210 NNT 0.81988158 Potential Glycosylated**

211 **N**TG -1.0455218 Non-glycosylated

221 **N**AA -1.1768378 Non-glycosylated

**241 NGT 0.81512571 Potential Glycosylated**

320 **N**HL -0.86795588 Non-glycosylated

341 **N**GD -1.2268679 Non-glycosylated

354 **N**MV -1.1592829 Non-glycosylated

**363 NGT 0.91571808 Potential Glycosylated**

367 **N**AA -1.0185327 Non-glycosylated

376 **N**TK -1.0636289 Non-glycosylated

394 **N**AQ -1.126936 Non-glycosylated

***********************************

>KAI7782878Diaporthe_eres Length = 402

**Potential N-Linked Glycosylated Sites:**

MQVAARPAKPCGSKGGAAKAA**N**GKAIYMLS**N**QAS**N**SVVAVPIAADGTL**N**EAGGSST**N**TGGSGA**N**GVDGTT**N**QPAAPDPLFSQSSLTVAG**NN**LFAV**N**AGS**N**

TLSMFAIDAQDPTKLTMVG**N**PADLPGEFPVTVGASAKH**N**LACVGMSGSTAGVSCASFDATQGLGAMDALRPFDLGQTTPPVGPT**N**TVSHVFFSDDQSTL

LSTVKGDPTK**NN**TGFLASFPV**N**AAGQCQAASVGAEGAQDSP**N**GTAVLFGSSPIAGSSDLFVTDASFGAAVLSTQQAAG**N**SAQASGAAEVVGKGAID**N**QK

ATCWVAISPATKTAFVTDVGV**N**HVVEMSLADASILGETDLSA**N**GDPGLIDLKAAG**N**MVYALSPG**N**GTT**N**AAVTVMDA**N**TKQQVQHLDMKSLGLDK**N**AQG

MAVLE

------------------------------------------------

Position Residue Score Prediction

------------------------------------------------

22 **N**GK -0.89397931 Non-glycosylated

31 **N**QA -1.2489954 Non-glycosylated

35 **N**SV -0.59539621 Non-glycosylated

49 **N**EA -1.0550335 Non-glycosylated

57 **N**TG -1.2256923 Non-glycosylated

64 **N**GV -0.91789112 Non-glycosylated

71 **N**QP -1.2339861 Non-glycosylated

90 **NN**L -0.52376216 Non-glycosylated

91 **N**LF -0.84203977 Non-glycosylated

96 **N**AG -0.79500126 Non-glycosylated

100 **N**TL -0.63319559 Non-glycosylated

120 **N**PA -1.7178078 Non-glycosylated

139 **N**LA -0.99863356 Non-glycosylated

185 **N**TV -0.91087519 Non-glycosylated

**210 NNT 0.84194828 Potential Glycosylated**

211 **N**TG -1.0455218 Non-glycosylated

221 **N**AA -1.1768378 Non-glycosylated

**241 NGT 0.81512571 Potential Glycosylated**

278 **N**SA -0.85920828 Non-glycosylated

296 **N**QK -1.1213395 Non-glycosylated

320 **N**HV -0.95537215 Non-glycosylated

341 **N**GD -1.2268679 Non-glycosylated

354 **N**MV -1.1592829 Non-glycosylated

**363 NGT 0.91571808 Potential Glycosylated**

367 **N**AA -1.0185327 Non-glycosylated

376 **N**TK -1.0636289 Non-glycosylated

394 **N**AQ -1.2640088 Non-glycosylated

***********************************

>XP_053000237Diaporthe_amy Length = 419

**Potential N-Linked Glycosylated Sites:**

MHISTFASIMMLAPSTIQVSARPARSCGKSMGGQAQPA**N**GKAIYMLS**N**QAS**N**SVVAVPIA**N**DGSLDAAGSSSTSTGGCGA**N**GVDGET**N**QPAAPDALFSQS

ALTVAG**NN**LFAV**N**AGS**N**TLSMFAIDAQDPTKLTMVG**N**PVDVPGEFPVTVGASAKH**N**LACVGMTGSTAGMACASFDAAQGLGEMDDLRPFDLGQTTPPVG

PT**N**TVSQVFFSDDQSMVFSTVKGDPTK**NN**TGFLASFPV**N**AACQGQAASVAAEGAQTSPSGTAVLFGSSPIAGSS**N**LFVTDASFGAAVLSMQQAA**N**GSAP

GSMVAEVAGKGVID**N**QKATCWAAISPATKTAFVTDVGV**N**HLVEMSLEDASIQSQTDLSA**N**GDPGLIDLKAAGSMVYALSPG**N**GTT**N**AAVTVLGAQSKEQ

VQHLDMSTLGLDK**N**AQGMAVLE

------------------------------------------------

Position Residue Score Prediction

------------------------------------------------

39 **N**GK -1.2074088 Non-glycosylated

48 **N**QA -1.2489954 Non-glycosylated

52 **N**SV -0.66477674 Non-glycosylated

61 **N**DG -1.250413 Non-glycosylated

81 **N**GV -0.98116297 Non-glycosylated

88 **N**QP -1.1470215 Non-glycosylated

107 **NN**L -0.54434225 Non-glycosylated

108 **N**LF -0.81501348 Non-glycosylated

113 **N**AG -0.79500126 Non-glycosylated

117 **N**TL -0.63319559 Non-glycosylated

137 **N**PV -1.680441 Non-glycosylated

156 **N**LA -0.92823483 Non-glycosylated

202 **N**TV -0.93379338 Non-glycosylated

**227 NNT 0.81988158 Potential Glycosylated**

228 **N**TG -1.0455218 Non-glycosylated

238 **N**AA -1.1369747 Non-glycosylated

274 **N**LF -1.2482558 Non-glycosylated

**294 NGS 1.0745497 Potential Glycosylated**

313 **N**QK -1.1530562 Non-glycosylated

337 **N**HL -0.86996823 Non-glycosylated

358 **N**GD -1.1537116 Non-glycosylated

**380 NGT 1.1274917 Potential Glycosylated**

384 **N**AA -0.94460677 Non-glycosylated

411 **N**AQ -1.2334824 Non-glycosylated

***********************************

>TGJ81826Xylaria_hypoxylon Length = 406

**Potential N-Linked Glycosylated Sites:**

MHTKSIISFTLVALAQQALARPHCKSPA**N**GKAIYMIS**N**EQK**N**AVLALPIGK**N**GLFTPGTATLTETGGAGMSGVDGDAGKPAGPDALFSQSSVTIAG**NN**LF

AV**N**PGS**N**TVSMFAISRQDPTKLTLVGKPVAVPAEFPVTVAASEQ**N**KLVCVGSSGAVAGVSCSKFSAQGIEEMDDLRVFDIGQTTPPVGPKGTVSHVFFS

EDESTVFTTVKGDPAK**N**KTGFLAAFPVGPSDCDSGASVSQDGMRSTPAGTVVLFGSSTIPGSD**N**LFVTDASFGGAVLT**N**GERSDEAASKAFSVKAKSAV

DGQSATCWVAISPATKTAFVTDVGV**N**RLVEMSLEDASIKKVIDLSA**N**GDPGLIDLRAAGHLVYALSPG**N**GTTPPAVTVVSAVTKKQVQHLQLEGI**N**FGK

**N**AQGMALLM

------------------------------------------------

Position Residue Score Prediction

------------------------------------------------

29 **N**GK -1.27899 Non-glycosylated

38 **N**EQ -0.98208786 Non-glycosylated

42 **N**AV -0.70593672 Non-glycosylated

52 **N**GL -1.0307464 Non-glycosylated

97 **NN**L -0.63405694 Non-glycosylated

98 **N**LF -0.87045635 Non-glycosylated

103 **N**PG -1.5879063 Non-glycosylated

107 **N**TV -1.062787 Non-glycosylated

145 **N**KL -1.0582753 Non-glycosylated

**216 NKT 0.75664214 Potential Glycosylated**

263 **N**LF -1.5030077 Non-glycosylated

278 **N**GE -1.2310651 Non-glycosylated

324 **N**RL -0.89652333 Non-glycosylated

345 **N**GD -1.3110842 Non-glycosylated

**367 NGT 0.93640428 Potential Glycosylated**

394 **N**FG -0.98069067 Non-glycosylated

398 **N**AQ -1.0262072 Non-glycosylated

***********************************

>KAF2970651Xylaria_multipl Length = 405

**Potential N-Linked Glycosylated Sites:**

MHSKSIIPLALTTLGLQALARPHC**N**TQA**N**GKAIYMIS**N**EQK**N**AVLALPIGK**N**GLFTPGTGRLTETGGAGM**N**GLDA**NN**QPAVPDPLFSQSSVTIAG**NN**LFA

V**N**PGS**N**SVSMFSISRQDPTKLTLVGKPVAVPAEFPVTVAASLQ**N**KLVCVGSSGAIAGVSCAKFSSQGIEEMDDLRPFDIGQTTPPVGPTGTVSHVFFSD

DELTVFTTVKGDPAK**N**KTGFMAAFPVEPSDCEAGVSV**N**QDGVRSTPDGTAVLFGSSTIPGS**N**DLFVTDASFGGAVLS**N**GG**N**SDDAAKKAFAVKGKGVVD

GQSATCWVAISPATKTAFVTDVGV**N**RLVEMSLEDASIKKVIDLSA**N**GDPGLIDLRAAGHLVYALSPG**N**GTTPAAVTVVDAVTKKQVQHFQLQGISFGK**N**

AQGMALLA

------------------------------------------------

Position Residue Score Prediction

------------------------------------------------

25 **N**TQ -1.0238786 Non-glycosylated

29 **N**GK -1.0632955 Non-glycosylated

38 **N**EQ -0.98208786 Non-glycosylated

42 **N**AV -0.70593672 Non-glycosylated

52 **N**GL -1.032108 Non-glycosylated

71 **N**GL -0.97968399 Non-glycosylated

76 **NN**Q -0.93767637 Non-glycosylated

77 **N**QP -1.511418 Non-glycosylated

96 **NN**L -0.63405694 Non-glycosylated

97 **N**LF -0.99744493 Non-glycosylated

102 **N**PG -1.6110382 Non-glycosylated

106 **N**SV -1.0667037 Non-glycosylated

144 **N**KL -1.0950094 Non-glycosylated

**215 NKT 0.83391672 Potential Glycosylated**

237 **N**QD -1.4499941 Non-glycosylated

261 **N**DL -0.95099544 Non-glycosylated

277 **N**GG -1.1192794 Non-glycosylated

280 **N**SD -1.024445 Non-glycosylated

323 **N**RL -0.89652333 Non-glycosylated

344 **N**GD -1.3110842 Non-glycosylated

**366 NGT 0.99996559 Potential Glycosylated**

397 **N**AQ -1.125675 Non-glycosylated

***********************************

>KAI0532188Xylaria_digitat Length = 405

**Potential N-Linked Glycosylated Sites:**

MHSKSIIPFAFTTLGLQALARPHC**N**SPA**N**GKAIYMIS**N**EQK**N**AVLALPIGK**N**GLFTPGTGTLTETGGAGM**N**GLDAS**N**QPAAPDPLFSQSSVTIAG**N**HLFA

V**N**PGS**N**SVSMFAISRQDPTKLTLVGKPVAVPAEFPVTIAASLQ**N**KLVCVGSSGATAGVSCSKFSGQGIEEMDDLRVFDIGQTTPPVGPTGTVSHVFFSE

DELTVFATVKGDPAK**N**KTGFMAAFPVEPSDCEAGVSV**N**QDGMRSTPEGTAVLFGSSTIPGSSDIFVTDASFGGAVLS**N**GGSSDDAARKAFIVKGKGVVD

GQSATCWVAISPTTKTAFVTDVGV**N**RLVEMSLEDASIKKVIDLSA**N**GDPGLIDLRAAGHLVYALSPG**N**GTAPAAVTVVDAVTKKQVQHFQLQGI**N**FGK**N**

AQGMALLA

------------------------------------------------

Position Residue Score Prediction

------------------------------------------------

25 **N**SP -1.2322051 Non-glycosylated

29 **N**GK -1.2609787 Non-glycosylated

38 **N**EQ -0.98208786 Non-glycosylated

42 **N**AV -0.70593672 Non-glycosylated

52 **N**GL -1.0330842 Non-glycosylated

71 **N**GL -0.95973396 Non-glycosylated

77 **N**QP -1.3175313 Non-glycosylated

96 **N**HL -0.7175121 Non-glycosylated

102 **N**PG -1.6831268 Non-glycosylated

106 **N**SV -1.1056615 Non-glycosylated

144 **N**KL -1.1001905 Non-glycosylated

**215 NKT 0.85057499 Potential Glycosylated**

237 **N**QD -1.5039888 Non-glycosylated

277 **N**GG -1.0622402 Non-glycosylated

323 **N**RL -0.89652333 Non-glycosylated

344 **N**GD -1.3110842 Non-glycosylated

**366 NGT 0.8560385 Potential Glycosylated**

393 **N**FG -1.0516047 Non-glycosylated

397 **N**AQ -1.027961 Non-glycosylated

***********************************

>XP_047826610Xylaria_bambu Length = 405

**Potential N-Linked Glycosylated Sites:**

MHSKQILPLALATLALQAQAHPRCKSPP**N**GKAIYMIS**N**EKD**N**AVLALPIGKDGLLSPGTATLTATGGAGMTGVDSEGQPAVPDALFSQSSITIAG**NN**LFA

V**N**AGS**N**SVSMFAISHQDPTKLTLVGKPVAVPAEFPVTVAASLK**N**KLVCVGSSGAVAGVSCSQFSSQGIEEMDDLRVFDIGQTTPPVGPTGTVSHAFFSE

DESTLFTTVKGRPDQ**N**RTGFLAAFAVGTSDCEASVSV**N**QEGMRSTPAGTAVLFGSSTIPGSDDLFVTDASFGGAVLA**N**GGRSEDAALKAFSVRGKGVVD

GQSATCWVAVSPATKTAFVTDVGV**N**RLVEMSLQDASIKKVIDLSA**N**GDPGLIDLRAAGRLVYALSPG**N**GSTPAAITVVDAVTKKQVQHFQIDAMPFGK**N**

AQGMAILL

------------------------------------------------

Position Residue Score Prediction

------------------------------------------------

29 **N**GK -1.2984009 Non-glycosylated

38 **N**EK -1.0416427 Non-glycosylated

42 **N**AV -0.92001417 Non-glycosylated

96 **NN**L -0.54617218 Non-glycosylated

97 **N**LF -1.0268116 Non-glycosylated

102 **N**AG -0.90333119 Non-glycosylated

106 **N**SV -0.78788149 Non-glycosylated

144 **N**KL -1.0776765 Non-glycosylated

**215 NRT 0.62872774 Potential Glycosylated**

237 **N**QE -1.3403078 Non-glycosylated

277 **N**GG -1.2018401 Non-glycosylated

323 **N**RL -0.87490051 Non-glycosylated

344 **N**GD -1.3110842 Non-glycosylated

**366 NGS 1.0309931 Potential Glycosylated**

397 **N**AQ -1.2128093 Non-glycosylated

***********************************

>XP_047826610Xylaria_bambu Length = 405

**Potential N-Linked Glycosylated Sites:**

MHSKQILPLALATLALQAQAHPRCKSPP**N**GKAIYMIS**N**EKD**N**AVLALPIGKDGLLSPGTATLTATGGAGMTGVDSEGQPAVPDALFSQSSITIAG**NN**LFA

V**N**AGS**N**SVSMFAISHQDPTKLTLVGKPVAVPAEFPVTVAASLK**N**KLVCVGSSGAVAGVSCSQFSSQGIEEMDDLRVFDIGQTTPPVGPTGTVSHAFFSE

DESTLFTTVKGRPDQ**N**RTGFLAAFAVGTSDCEASVSV**N**QEGMRSTPAGTAVLFGSSTIPGSDDLFVTDASFGGAVLA**N**GGRSEDAALKAFSVRGKGVVD

GQSATCWVAVSPATKTAFVTDVGV**N**RLVEMSLQDASIKKVIDLSA**N**GDPGLIDLRAAGRLVYALSPG**N**GSTPAAITVVDAVTKKQVQHFQIDAMPFGK**N**

AQGMAILL

------------------------------------------------

Position Residue Score Prediction

------------------------------------------------

29 **N**GK -1.2984009 Non-glycosylated

38 **N**EK -1.0416427 Non-glycosylated

42 **N**AV -0.92001417 Non-glycosylated

96 **NN**L -0.54617218 Non-glycosylated

97 **N**LF -1.0268116 Non-glycosylated

102 **N**AG -0.90333119 Non-glycosylated

106 **N**SV -0.78788149 Non-glycosylated

144 **N**KL -1.0776765 Non-glycosylated

**215 NRT 0.62872774 Potential Glycosylated**

237 **N**QE -1.3403078 Non-glycosylated

277 **N**GG -1.2018401 Non-glycosylated

323 **N**RL -0.87490051 Non-glycosylated

344 **N**GD -1.3110842 Non-glycosylated

**366 NGS 1.0309931 Potential Glycosylated**

397 **N**AQ -1.2128093 Non-glycosylated

***********************************

>XP_047826610Xylaria_bambu Length = 405

**Potential N-Linked Glycosylated Sites:**

MHSKQILPLALATLALQAQAHPRCKSPP**N**GKAIYMIS**N**EKD**N**AVLALPIGKDGLLSPGTATLTATGGAGMTGVDSEGQPAVPDALFSQSSITIAG**NN**LFA

V**N**AGS**N**SVSMFAISHQDPTKLTLVGKPVAVPAEFPVTVAASLK**N**KLVCVGSSGAVAGVSCSQFSSQGIEEMDDLRVFDIGQTTPPVGPTGTVSHAFFSE

DESTLFTTVKGRPDQ**N**RTGFLAAFAVGTSDCEASVSV**N**QEGMRSTPAGTAVLFGSSTIPGSDDLFVTDASFGGAVLA**N**GGRSEDAALKAFSVRGKGVVD

GQSATCWVAVSPATKTAFVTDVGV**N**RLVEMSLQDASIKKVIDLSA**N**GDPGLIDLRAAGRLVYALSPG**N**GSTPAAITVVDAVTKKQVQHFQIDAMPFGK**N**

AQGMAILL

------------------------------------------------

Position Residue Score Prediction

------------------------------------------------

29 **N**GK -1.2984009 Non-glycosylated

38 **N**EK -1.0416427 Non-glycosylated

42 **N**AV -0.92001417 Non-glycosylated

96 **NN**L -0.54617218 Non-glycosylated

97 **N**LF -1.0268116 Non-glycosylated

102 **N**AG -0.90333119 Non-glycosylated

106 **N**SV -0.78788149 Non-glycosylated

144 **N**KL -1.0776765 Non-glycosylated

**215 NRT 0.62872774 Potential Glycosylated**

237 **N**QE -1.3403078 Non-glycosylated

277 **N**GG -1.2018401 Non-glycosylated

323 **N**RL -0.87490051 Non-glycosylated

344 **N**GD -1.3110842 Non-glycosylated

**366 NGS 1.0309931 Potential Glycosylated**

397 **N**AQ -1.2128093 Non-glycosylated

***********************************

>KAI3334604Ustulina_deusta Length = 407

**Potential N-Linked Glycosylated Sites:**

MHSKPIIPLVLATLALQTLAYPRCKSPP**N**GRAIYMIS**N**EKE**N**AVLALPIGK**N**GLFTPGTATLTATGGAGM**N**GVDGDLGGQPAGPDALFSQSSITIAG**NN**L

FAV**N**PGS**N**SVSMFTIS**N**QDPTKLTLIGKPVAVPAEFPVTVAASER**N**KLVCVGSSGAVAGVSCSRFSAGGIEAMDDLRVFDIGQTTPPVGPKGTVSHAFF

SEDESTLFTTVKGLPAQ**N**RTGFLAAFAVGTSDCEAGVSV**N**QEGIRSTPAGTLVLFGSSTIPGSD**N**LFVTDASFGGAVLT**N**GGRGEDAAIKAFSVRGKGV

VEGQSATCWVAISPATKTAFVTDVGV**N**RLVEMSLEDASIKKVIDLSA**N**GDPGLIDLRAAGRLVYALSPG**N**GSTPAAITVVDAVTKKQVQHFQVKGLEFG

KSAQGMAMFL

------------------------------------------------

Position Residue Score Prediction

------------------------------------------------

29 **N**GR -1.1683935 Non-glycosylated

38 **N**EK -1.0089139 Non-glycosylated

42 **N**AV -0.77333602 Non-glycosylated

52 **N**GL -1.0307464 Non-glycosylated

71 **N**GV -1.2625281 Non-glycosylated

98 **NN**L -0.58445802 Non-glycosylated

99 **N**LF -0.98836805 Non-glycosylated

104 **N**PG -1.5610978 Non-glycosylated

108 **N**SV -1.0266451 Non-glycosylated

117 **N**QD -1.3810945 Non-glycosylated

146 **N**KL -1.0604253 Non-glycosylated

**217 NRT 0.68773417 Potential Glycosylated**

239 **N**QE -1.3285106 Non-glycosylated

264 **N**LF -1.5030077 Non-glycosylated

279 **N**GG -1.1367142 Non-glycosylated

325 **N**RL -0.89652333 Non-glycosylated

346 **N**GD -1.3110842 Non-glycosylated

**368 NGS 1.0309931 Potential Glycosylated**

***********************************

>KAI3334604Ustulina_deusta Length = 407

**Potential N-Linked Glycosylated Sites:**

MHSKPIIPLVLATLALQTLAYPRCKSPP**N**GRAIYMIS**N**EKE**N**AVLALPIGK**N**GLFTPGTATLTATGGAGM**N**GVDGDLGGQPAGPDALFSQSSITIAG**NN**L

FAV**N**PGS**N**SVSMFTIS**N**QDPTKLTLIGKPVAVPAEFPVTVAASER**N**KLVCVGSSGAVAGVSCSRFSAGGIEAMDDLRVFDIGQTTPPVGPKGTVSHAFF

SEDESTLFTTVKGLPAQ**N**RTGFLAAFAVGTSDCEAGVSV**N**QEGIRSTPAGTLVLFGSSTIPGSD**N**LFVTDASFGGAVLT**N**GGRGEDAAIKAFSVRGKGV

VEGQSATCWVAISPATKTAFVTDVGV**N**RLVEMSLEDASIKKVIDLSA**N**GDPGLIDLRAAGRLVYALSPG**N**GSTPAAITVVDAVTKKQVQHFQVKGLEFG

KSAQGMAMFL

------------------------------------------------

Position Residue Score Prediction

------------------------------------------------

29 **N**GR -1.1683935 Non-glycosylated

38 **N**EK -1.0089139 Non-glycosylated

42 **N**AV -0.77333602 Non-glycosylated

52 **N**GL -1.0307464 Non-glycosylated

71 **N**GV -1.2625281 Non-glycosylated

98 **NN**L -0.58445802 Non-glycosylated

99 **N**LF -0.98836805 Non-glycosylated

104 **N**PG -1.5610978 Non-glycosylated

108 **N**SV -1.0266451 Non-glycosylated

117 **N**QD -1.3810945 Non-glycosylated

146 **N**KL -1.0604253 Non-glycosylated

**217 NRT 0.68773417 Potential Glycosylated**

239 **N**QE -1.3285106 Non-glycosylated

264 **N**LF -1.5030077 Non-glycosylated

279 **N**GG -1.1367142 Non-glycosylated

325 **N**RL -0.89652333 Non-glycosylated

346 **N**GD -1.3110842 Non-glycosylated

**368 NGS 1.0309931 Potential Glycosylated**

***********************************

>KAI1357184Xylaria_arbuscu Length = 405

**Potential N-Linked Glycosylated Sites:**

MFSKPILPFALAALALQSQAHPRCRSPP**N**GKAIYMIS**N**EKD**N**TVLALPIGKDGLLSPETMTLTATGGVGMAGVESDGQPAGPDSLFSQSSVTIAG**N**YLFA

V**N**PGS**N**SVSMFTISGEDPTKLTLVGKPATVPAEFPVTVAASAK**NN**VVCVASSGAVAGVSCSKFSSQGIEEMDGLRAFDIGQTTPPVGPTGTVSHAFFSE

DESTLFTTVKGLPEQ**N**RTGFLAAFAVESSDCETGAFV**N**QEGMRSTPAGTAVLFGSSTIPGSDDLFVTDASFGGAVLA**N**GGTSADAASRAFSVKGKGVV**N**

GQSATCWVAISPAT**N**TAFVTDVGV**N**RLVEMSLKDASVKKIIDLSA**N**GDPGLIDLKAAGRLVYALSPG**N**GSTPAAITIV**N**AITKQQVQHFQIDGVPFGKS

AQGMAILV

------------------------------------------------

Position Residue Score Prediction

------------------------------------------------

29 **N**GK -1.3573935 Non-glycosylated

38 **N**EK -0.98940972 Non-glycosylated

42 **N**TV -0.91753194 Non-glycosylated

96 **N**YL -0.790995 Non-glycosylated

102 **N**PG -1.5436024 Non-glycosylated

106 **N**SV -1.0124095 Non-glycosylated

144 **NN**V -0.96450098 Non-glycosylated

145 **N**VV -0.82997035 Non-glycosylated

**215 NRT 0.57451608 Potential Glycosylated**

237 **N**QE -1.295492 Non-glycosylated

277 **N**GG -1.290857 Non-glycosylated

298 **N**GQ -1.0188105 Non-glycosylated

313 **N**TA -0.86395599 Non-glycosylated

323 **N**RL -0.96325755 Non-glycosylated

344 **N**GD -1.2169005 Non-glycosylated

**366 NGS 1.002859 Potential Glycosylated**

377 **N**AI -0.95673313 Non-glycosylated

***********************************

>GAP83551Rosellinia_necatr Length = 403

**Potential N-Linked Glycosylated Sites:**

MHRSPLISLVLAPLVLQALARPHC**N**TLA**N**GKAIYMLS**N**EQD**N**AVVALPIQK**N**GLLAAGRLTGTGGAGM**N**GMDADGKPAAPDPLFSQSSLTIAG**N**HLFAV**N**

PGS**N**TVSMFAIDPRDPTRLTLVGQPVAVPGEFPVTVAASER**N**KLVCVGSSGAVAGVACSTFSADGIGEMDDLRVFDIGQTTPPVGPT**N**TVSHVFFSRDE

STVFTTVKGDPAT**N**KTGFLAAFPVDRSDCEAGASVAREGRRSSPAGTAVLFGSSTIPGSDDLFVTDASFGGAVLT**N**GGGRGGDAAAFTVRGKGVVDGQS

ATCWAAISPAT**N**TAFVTDVGV**N**RLVEMSLGDASIRSVVDLGA**N**GDPGLIDLRAAGRLVYALSPG**N**GTTPAAVTVVDAVTKKQVQHLQF**N**GGMRFGK**N**AQ

GMALLM

------------------------------------------------

Position Residue Score Prediction

------------------------------------------------

25 **N**TL -1.1037031 Non-glycosylated

29 **N**GK -1.1033786 Non-glycosylated

38 **N**EQ -0.97104182 Non-glycosylated

42 **N**AV -0.8568228 Non-glycosylated

52 **N**GL -0.93648341 Non-glycosylated

69 **N**GM -1.0446524 Non-glycosylated

94 **N**HL -0.64879328 Non-glycosylated

100 **N**PG -1.6748903 Non-glycosylated

104 **N**TV -1.0275837 Non-glycosylated

142 **N**KL -1.0604253 Non-glycosylated

188 **N**TV -0.8094592 Non-glycosylated

**213 NKT 0.77553402 Potential Glycosylated**

275 **N**GG -1.1048137 Non-glycosylated

310 **N**TA -0.88408248 Non-glycosylated

320 **N**RL -0.91744214 Non-glycosylated

341 **N**GD -1.3260416 Non-glycosylated

**363 NGT 1.0367459 Potential Glycosylated**

387 **N**GG -0.87245781 Non-glycosylated

395 **N**AQ -1.1732519 Non-glycosylated

***********************************

>KAI0443997Xylaria_telfair Length = 404

**Potential N-Linked Glycosylated Sites:**

MHCKPIIPFVLAPLVLQAQARPQCRSPA**N**GKAIYMIS**N**EDK**N**AVLALPIGRHGLFAAGTGTLTATGGSGMTGVQAD**N**SPAIPDGLFSQSSVTIAG**N**HLFA

V**N**PGS**N**TVSMFAI**N**PYDPTKLTLVGAPVAVPGEFPVTVAASKR**N**KVVCVGTSGAVAGLSCATFSAQGIGAMDSLRTFDIGQTTPPVGPT**N**TVSHAFFSE

DESTIFTTVKGDPAT**N**KTGFLAAFPI**N**APSDCEARAAVDQQGMRSSPVGTAVLFGSSPISGSD**N**LFVTDASFGAAVLT**N**GGVDVAKATFTVQGKGVIDG

QSATCWAAISPATKTAFVTDVGV**N**RLVEMSLDDASIK**N**IIDLST**N**GDPGLIDLRAAG**N**LVYALSPG**N**GSTPAAITVVDAVTKKQVQHFQLEGIDFGKRA

QGMAILV

------------------------------------------------

Position Residue Score Prediction

------------------------------------------------

29 **N**GK -1.346575 Non-glycosylated

38 **N**ED -1.1983748 Non-glycosylated

42 **N**AV -0.74348786 Non-glycosylated

77 **N**SP -1.3662748 Non-glycosylated

96 **N**HL -0.7175121 Non-glycosylated

102 **N**PG -1.6748903 Non-glycosylated

106 **N**TV -1.0662565 Non-glycosylated

114 **N**PY -1.2148386 Non-glycosylated

144 **N**KV -1.2066208 Non-glycosylated

190 **N**TV -0.88969332 Non-glycosylated

**215 NKT 0.80784073 Potential Glycosylated**

**226** **N**AP -0.82679583 Non-glycosylated

263 **N**LF -1.3487077 Non-glycosylated

278 **N**GG -0.80431931 Non-glycosylated

322 **N**RL -0.85859887 Non-glycosylated

336 **N**II -1.3342417 Non-glycosylated

343 **N**GD -1.2011874 Non-glycosylated

356 **N**LV -1.1538701 Non-glycosylated

**365 NGS 1.0849471 Potential Glycosylated**

***********************************

>KAI0454232Xylaria_acuta Length = 404

**Potential N-Linked Glycosylated Sites:**

MHCKPIIPFVLAPLALQVLARPHCQGQA**N**GKAIYMIS**N**EPR**N**AVLALPIGKHGLFAAGTGTLTATGGSGMTGL**N**AD**N**QSAVPDGLFSQSSVTIAG**NN**LFA

V**N**PGS**N**TVSMFAI**N**RHDPTKLTLVGQPVAVPGEFPVTVAASER**N**KVVCVATSGAVAGMSCSTFSAQGIGAMDDLRAFDIGQTTPPVGPT**N**TVSHVFFSE

DESTVFTTVKGDPAT**N**KTGFLAAFPVIAQSDCEARASVDQQGMRSSPAGTAVLFGTSIISGTD**N**LFATDASFGAAVLTSGGVDVARAAFTVKGKGVIDG

QSATCWSVISPATKTAFVTDVGV**N**RLVEMSLEDASIK**N**TIDLTK**N**GDPGLIDLRAAG**N**LVYALSPG**N**GTTLAAITVVDAVTKKQVQHFQLEGIAFGK**N**A

QGMAMLL
[truncated: 29,305 more chars]
